# Association of urbanization-related factors with tuberculosis incidence among 1992 counties in China from 2005 to 2019: a nationwide observational study

**DOI:** 10.1186/s40249-025-01299-4

**Published:** 2025-04-25

**Authors:** Yaping Wang, Xiaoqiu Liu, Yuhong Li, Min Liu, Yiheng Wang, Hongliang Zhang, Jue Liu, Yanlin Zhao

**Affiliations:** 1 Department of Epidemiology and Biostatistics, School of Public Health, Peking University, No.38 Xueyuan Road, Haidian District, Beijing, 100191 China; 2 Key Laboratory of Epidemiology of Major Diseases (Peking University), Ministry of Education, No.38 Xueyuan Road, Haidian District, Beijing, 100191 China; 3 National Center for Tuberculosis Control and Prevention, Chinese Center for Disease Control and Prevention, No.155 Changbai Road, Changping District, Beijing, 102211 China; 4 Department of Environmental Science and Engineering, Fudan University, 220 Handan Road, Shanghai, 200433 China; 5 Global Center for Infectious Disease and Policy Research & Global Health and Infectious Diseases Group, Peking University, No.38 Xueyuan Road, Haidian District, Beijing, China

**Keywords:** Tuberculosis, Urbanization, County, China

## Abstract

**Background:**

Most high tuberculosis (TB) burden countries are in low- and middle-income regions undergoing rapid urbanization. We aimed to assess the association between urbanization factors and TB incidence in China.

**Methods:**

We evaluated urbanization at the county level in China from 2005 to 2019 using a composite index integrating population density, gross domestic product (GDP, per capita), hospital beds per 1000 population, nighttime light (NTL), and normalized difference vegetation index (NDVI). The annual incidence rate and number of TB cases were obtained from the national Tuberculosis Information Management System, maintained by the Chinese Center for Disease Control and Prevention. Fixed-effects models were used to examine the association between urbanization factors and TB incidence. A subgroup analysis was performed by dividing counties into four regions: northeast, eastern, central, and western.

**Results:**

A total of 1992 counties in China were included in this study. Overall, urbanization scores were associated with reduced TB incidence (β = − 0.0114, *P* < 0.001), corresponding to a 1.1% reduction in TB incidence per unit score. Quadratic models presented a U-shaped relationship between urbanization and TB incidence with an inflection point at 52.94 urbanization units. For each indicator of urbanization, population density and the number of hospital beds were positively associated with TB incidence, with incidence rate ratios of 11.384 [95% confidence interval (*CI*): 9.337 to 13.881], and 1.015 (95%* CI*: 1.011 to 1.019), respectively, while GDP, NTL, and NDVI exhibited protective effects. Central China displayed an increase trend that urbanization score was linked to a 1.8% rise in TB incidence.

**Conclusions:**

Urbanization-related factors, including GDP, NTL and NDVI, were inversely associated with TB incidence. Central China’s contrasting results highlighted region-specific challenges. Therefore, governments in developing countries should adopt integrated approaches that promote both economic growth and sustainable development of environment during urbanization to optimize TB control efforts.

**Graphical abstract:**

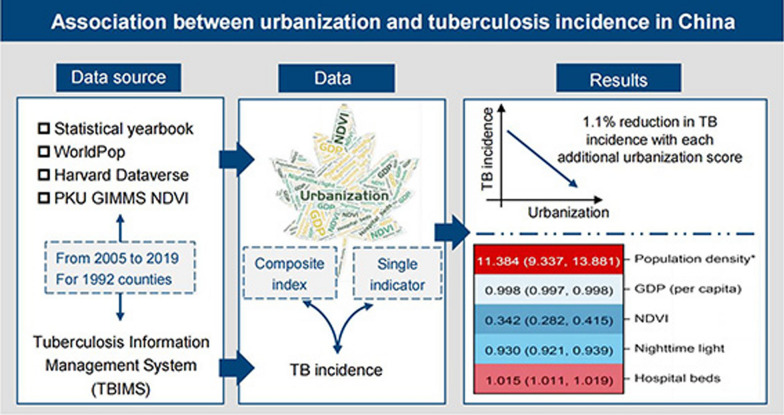

**Supplementary Information:**

The online version contains supplementary material available at 10.1186/s40249-025-01299-4.

## Background

Although tuberculosis (TB) is both preventable and curable, it remains the leading cause of death from a single infectious agent worldwide, second only to coronavirus disease (COVID-19) [1]. In 2022, the World Health Organization (WHO) estimated 7.5 million new TB cases and 1.3 million TB-related deaths globally [1]. TB is a disease of poverty. The 30 high TB burden countries—accounting for 87% of the world’s new cases in 2022—are predominantly low- and middle-income countries (LMICs) [1]. Despite decades of progress in TB prevention, LMICs continue to face significant challenges in achieving the 2030 End TB strategy targets. For instance, in China, older adults aged 60–84 experienced higher TB incidence than other age groups, with the peak occurring in those aged 70–74 [2]. Given the rapidly aging population, the TB burden in China remains substantial. Adding to these challenges, drug-resistant TB—particularly multi-drug resistant TB (MDR-TB)—poses a major obstacle to global TB prevention and control efforts [3]. A systematic review and meta-analysis conducted in China found that among TB new cases, the resistance rate to any drug was 20.1%, which was 49.8% among TB retreatment cases [4]. Furthermore, although preventive measures against transmission of COVID-19 restricted the spread of TB to some extent, pandemic-related delays in TB diagnosis and treatment deflected globe from the track to realize the End TB Strategy targets [1, 5]. In response, the UN General Assembly held its second high-level meeting on TB in 2023 and agreed upon new commitments and targets for 2023–2027 to ensure effective, high-quality, and people-centered TB care [6].

Urbanization, characterized by rural-to-urban migration and socioeconomic transformation, has continued to accelerate, even during the COVID-19 pandemic [7–9]. In 2021, an estimated 56% of the world’s population resided in urban areas, a figure projected to rise to 68% by 2050 [9]. Notably, low-income countries are experiencing much higher absolute and relative urban population growth compared to high-income countries, with city population expected to exceed 700 million by 2070, mainly driven by the nature growth of population [9, 10]. Rapid urban expansion in these regions, often marked by unsustainable land development, can exacerbate poverty, inequality, and health disparities. While well-planned urbanization has been shown to benefit TB prevention and control globally, unsustainable and unequitable urbanization can have adverse effects, including excessive population density, environmental degradation (e.g. climate change, air pollution, greenhouse gas emissions, and loss of green space), an increase in urban slum populations, and inadequate health services [11, 12].

Urbanization is a complex socioeconomic process, entailing not only population concentration but also systemic transformations in land use, governance, and economic activity [13]. Consequently, urbanization can be measured using various indicators depending on the research discipline, such as population density (demography), land use (geography), changes in production modes (economics), and the formation of large, diverse permanent settlements (sociology) [14]. Previous studies have primarily used indicators such as gross domestic product (GDP), the proportion of urban or agricultural populations and population density—focusing solely on the physical aspects of urbanization [15–17]. However, newer indicators, such as the normalized difference vegetation index (NDVI) and nighttime light (NTL), which reflect additional dimensions of urbanization, remain underexplored [18–20]. To address this gap, our study aims to develop a composite index incorporating multiple urbanization aspects to provide a more comprehensive assessment. Current studies on the relationship between urbanization and TB have predominantly relied on theoretical reviews or have been conducted in developed countries [21, 22]. Most researches have focused on comparing TB epidemiology between urban and rural areas, with limited reliable and quantified evidence from China or other developing countries, particularly regarding varying degrees of urbanization, such as towns [12, 21, 22]. China’s “Key Tasks for New Urbanization and Urban-Rural Integration Development in 2021” emphasized the importance of county-level urbanization in promoting urban-rural integration and balanced development [23]. Understanding how urbanization affects TB incidence at the county level is crucial for designing targeted interventions that address urban-rural disparities in China.

Identifying the association between urbanization factors and TB can provide valuable insights for developing sustainable human habitats and accelerating progress toward the End TB Strategy targets. In this study, we conducted an observational study, using national reported data on TB incidence and data from multiple sources on environmental, demographic, and socioeconomic indicators across county-level regions in China from 2005 to 2019, to examine the relationship between urbanization factors and TB incidence.

## Methods

### Selected counties and data sources

As of the end of 2005, China had 2862 county-level administrative divisions, including 2010 counties and county-level cities (hereinafter collectively referred to as “counties”). In this study, we excluded counties that were merged or split between 2006 and 2019 (*n* = 2) and those with missing data on urbanization indicators (*n* = 16). Finally, 1992 counties were included in the analysis.

We utilized data from multiple sources to examine the association between urbanization factors and TB incidence in China. For each of the 1992 counties, annual TB incidence rate and cases number were obtained from the Tuberculosis Information Management System (TBIMS), maintained by the Chinese Center for Disease Control and Prevention, covering the period from January 1, 2005 to December 31, 2019 [24]. TBIMS users comprise all TB healthcare facilities, such as TB dispensaries and designated hospitals at province, prefectural, and county levels. All TB-reporting facilities in China are required to report confirmed TB cases via TBIMS within 48 hours [24]. TB case confirmation is based on epidemiological history, clinical symptoms and signs, chest imaging, and laboratory tests.

Urbanization indicators for each county from 2005 to 2019 were collected from multiple sources, including the *China Statistical Yearbook* (national and provincial levels), the *China County Statistical Yearbook* (GDP and hospital beds), WorldPop (population density) [25], Harvard Dataverse (NTL), [26] and PKU GIMMS NDVI (NDVI) [27]. Raw raster data for population density, NTL, and NDVI were downloaded at spatial resolution of 30 arcseconds (~1 km), 15 arcseconds (~500 m), and 1/12°, respectively. We used the “terra” package in R 4.3.0 (Lucent Technologies, Jasmine Mountain, USA) to extract annual data and calculate county-level values.

Meteorological factors, including annual average atmospheric temperature, wind speed, and relative humidity, were derived from simulations using the Weather Research and Forecasting (WRF) model (version 4.1.2). The model applied Final (FNL) Operational Global Analysis data from the National Centers for Environmental Prediction (NCEP; https://rda.ucar.edu/datasets/ds083.3/) as meteorological input, with a spatial resolution of 1.0° × 1.0°. Fine particulate matter (PM_2.5_) data were simulated using the Community Multiscale Air Quality (CMAQ) model version 5.0.2 with inputs of meteorological factors, anthropogenic emissions and biogenic emissions. Meteorological factors were generated using WRF version 4.1.2. The anthropogenic emissions were sourced from the Multi-resolution Emission Inventory for China (MEIC; http://www.meicmodel.org/) and the Emissions Database for Global Atmospheric Research data (EDGAR; https://edgar.jrc.ec.europa.eu/). Biogenic emissions were sourced from the Model of Emissions of Gases and Aerosols from Nature (MEGAN) version 2.1 [28]. Both simulations from WRF version 4.1.2 and CMAQ version 5.0.2 spanned from 2005 to 2019, configured a 197 × 127 grid with a horizontal resolution of 36 km × 36 km. Outputs of meteorological and PM_2.5_ data for each county based on their annual latitude and longitude coordinates from the original grid. Details for the sources and methods in meteorological factors and PM_2.5_ were shown in Online Appendix.

### Assessment of the urbanization factors

To comprehensively assess urbanization, we constructed an index integrating multiple indicators, including the population density (per square kilometers) representing population migration, GDP (per capita) indicating economic development, the number of hospital beds (per 1000 population) reflecting health-related development, NTL intensity representing the urban construction, and NDVI capturing environment changes.

Urbanization factors for each county were assessed using weighted cumulative scores across these five indicators [23, 29]. The entropy evaluation method was used to determine the weight for each indicator annually (Table S1) [23]. The mean weights of each indicator from 2005 to 2019 were as follows: population density (0.20), GDP (0.14), number of hospital beds (0.06), NTL (0.57), and NDVI (0.03). The weights used to calculate the annual urbanization score were similar (Table S2).

### Changes in urbanization factors and TB incidence

We used the median and interquartile range (IQR) to describe the distribution of urbanization indicators and TB incidence. To analyze temporal trends in urbanization scores and TB incidence from 2005 to 2019 across 1992 counties, we calculated the estimated annual percentage change (EAPC), a widely used measure for assessing temporal trends [30]. EAPC was derived by fitting the following regression model:

1 $$y = \alpha + \beta x + \varepsilon$$

where y represents the natural logarithm of the urbanization score or TB incidence, x denotes the calendar year, and ε is an independent error term following a normal distribution. EAPC was computed as $100\times ({e}^{\upbeta }-1)$ with the 95% confidence interval (*CI*) derived from the estimation of β. A positive EAPC with a lower 95% *CI* boundary above zero represents indicates an upward trend, whereas a negative EAPC with an upper 95% *CI* boundary below zero indicates a downward trend. Otherwise, the trend is considered stable.

### Relationship between urbanization factors and TB incidence

To investigate the association between urbanization factors and TB incidence, we applied a two-way fixed-effects linear model:

2 $$Ln\left( {Y_{it} } \right) = \beta_{i} + \beta_{t} + \beta_{1} \times score_{it} + \beta_{2} \times temp_{it} + \beta_{3} \times ws_{it} + \beta_{4} \times rh_{it} + \beta_{5} \times PM_{2.5it} + \varepsilon_{it}$$

where *i* signifies counties (*i* = 1, 2, …, *N* = 1992) and* t* indicates year in the analysis (*t* = 2005, 2006, …, *T* = 2019). ${\text{Y}}_{it}$ represents the natural logarithm of TB incidence, as the data were very skewed. ${\upbeta }_{i}$ captures county-specific fixed effects, which remain constant over time, while ${\upbeta }_{t}$ is county-invariant but varies between years. ${\upbeta }_{1}$ measures the effect of urbanization on TB incidence. ${\upbeta }_{2}$ to ${\upbeta }_{5}$ represent the coefficients of covariates. ${\text{score}}_{it}$ represents the urbanization score of county *i* in year *t*. ${\text{temp}}_{it}$, ${\text{ws}}_{it}$, and ${\text{rh}}_{it}$ refer to the mean temperature, wind speed, and relative humidity of county *i* in year *t*. ${\upvarepsilon }_{it}$ is the error term. The effect size of urbanization on TB incidence was interpreted as $\text{exp}\left({\upbeta }_{1}\right)$, representing the percentage change in TB incidence per unit increase in the urbanization score. Furthermore, we incorporated a quadratic term for urbanization to capture potential nonlinear patterns.

Subgroup analyses were performed by stratifying counties into four regions (northeast, eastern, central, and western) according to China’s income policies (Table S3). A sensitivity analysis was also conducted using the same model by establishing a new composite index without the number of hospital beds.

All statistical analyses were performed in the R 4.3.0 (Lucent Technologies, Jasmine Mountain, USA). Data visualization was conducted using Origin2020b (OriginLab Corporation, Northampton, USA). A two-sided *P*-value < 0.05 was considered statistically significant.

## Results

### Descriptive statistics of TB and urbanization

Table 1 presents descriptive statistics on the annual incidence rate and the number of TB cases, along with urbanization indicators from 2005 to 2019. Over the study period, a total of 14,621,814 TB cases were reported in China, with an annual average of 974,788 TB cases. Nationally, the median TB incidence decreased from 95.61 per 100,000 population in 2005 to 54.51 per 100,000 population in 2019. However, trends in reported TB incidence varied across the 1992 counties. From 2005 to 2019, a downward trend in TB incidence was observed in 1283 counties, accounting for 64.41% of the total counties included in this study (Table S4). Conversely, 123 counties (6.17%) exhibited an upward trend, primarily concentrated in western China (Table S4). The most significant decline in TB incidence was recorded in Aksay Kazakh Autonomous County, Gansu Province, with an average annual decrease of 15.16% (EAPC = − 15.16, 95% *CI*: − 21.91 to − 7.84).

**Table 1:** Trends of urbanization factors and incidence of tuberculosis among 1992 counties in China from 2005 to 2019

Year	Urbanization factors						TB	
	Composite index (Urbanization score)	Single indicator						
		Population density (per square kilometers)	NDVI	Nighttime light	GDP (per capita)	Number of hospital beds (per 1000 population)	Number of incident cases (people)	Incidence (per 100,000 population)
2005	4.44 (3.46, 6.11)	172.88 (75.82, 401.52)	0.61 (0.52, 0.69)	0.02 (0.01, 0.07)	7196.00 (4799.50, 11,108.00)	1.67 (1.25, 2.30)	341 (178, 610)	95.61 (68.89, 131.04)
2006	4.41 (3.47, 5.92)	173.44 (76.33, 404.11)	0.60 (0.51, 0.69)	0.02 (0.01, 0.08)	8291.50 (5550.25, 12,909.50)	1.74 (1.30, 2.44)	303 (166, 556)	82.89 (61.15, 113.36)
2007	4.45 (3.51, 5.87)	174.68 (77.14, 408.45)	0.62 (0.52, 0.70)	0.03 (0.01, 0.09)	9994.00 (6757.75, 15,816.50)	1.86 (1.38, 2.52)	314 (165, 568)	84.54 (61.80, 120.08)
2008	4.32 (3.34, 5.76)	175.37 (77.25, 408.62)	0.62 (0.53, 0.70)	0.03 (0.01, 0.09)	12,329.50 (8086.00, 19,334.25)	1.99 (1.50, 2.65)	316 (168, 574)	84.04 (63.29, 113.90)
2009	4.78 (3.82, 6.39)	176.18 (77.26, 411.80)	0.62 (0.52, 0.70)	0.03 (0.01, 0.10)	13,517.00 (9141.75, 21,817.00)	2.12 (1.64, 2.80)	291 (160, 501)	79.57 (61.83, 105.00)
2010	4.71 (3.69, 6.36)	176.97 (77.88, 413.37)	0.62 (0.52, 0.71)	0.03 (0.01, 0.12)	16,769.00 (11,141.50, 26,382.25)	2.29 (1.78, 3.02)	270 (151, 454)	73.80 (55.74, 96.98)
2011	4.63 (3.65, 6.13)	177.39 (78.44, 412.73)	0.62 (0.52, 0.70)	0.05 (0.02, 0.17)	20,357.00 (13,230.92, 31,507.25)	2.50 (1.93, 3.20)	262 (144, 442)	73.84 (55.91, 98.55)
2012	4.62 (3.62, 6.34)	178.77 (78.87, 415.60)	0.63 (0.54, 0.72)	0.04 (0.01, 0.15)	23,262.66 (15,588.27, 36,169.25)	2.75 (2.15, 3.51)	261 (142, 457)	73.07 (55.91, 98.01)
2013	5.30 (4.10, 7.13)	180.41 (79.14, 417.72)	0.62 (0.53, 0.72)	0.10 (0.03, 0.30)	25,968.91 (17,483.00, 40,508.75)	3.12 (2.40, 3.92)	246 (134, 432)	69.04 (52.46, 95.42)
2014	5.37 (4.23, 7.22)	181.04 (79.34, 420.11)	0.63 (0.52, 0.72)	0.10 (0.03, 0.30)	27,635.78 (19,073.60, 43,116.50)	3.37 (2.64, 4.20)	237 (126, 415)	67.19 (49.05, 92.20)
2015	5.77 (4.61, 7.64)	182.73 (79.62, 421.07)	0.63 (0.53, 0.73)	0.10 (0.03, 0.30)	28,909.00 (20,256.54, 44,720.17)	3.57 (2.87, 4.44)	226 (125, 400)	64.12 (46.28, 89.47)
2016	5.80 (4.59, 7.76)	182.90 (80.12, 421.64)	0.64 (0.54, 0.73)	0.11 (0.03, 0.32)	30,852.12 (21684.00, 47,184.25)	3.89 (3.10, 4.85)	220 (119, 383)	61.52 (44.13, 87.81)
2017	6.51 (5.05, 8.68)	184.38 (79.76, 423.38)	0.64 (0.54, 0.73)	0.15 (0.04, 0.43)	32,799.00 (23,295.94, 50,041.50)	4.22 (3.40, 5.19)	220 (121, 378)	60.35 (42.93, 85.37)
2018	5.83 (4.54, 7.92)	185.58 (79.78, 423.91)	0.63 (0.54, 0.74)	0.16 (0.05, 0.49)	35,094.00 (25,120.50, 53,296.54)	4.42 (3.54, 5.47)	209 (116, 363)	56.78 (39.79, 81.48)
2019	6.13 (4.71, 8.37)	186.75 (79.86, 426.02)	0.63 (0.53, 0.74)	0.18 (0.06, 0.56)	36,542.33 (26,671.91, 55,562.12)	4.63 (3.75, 5.76)	201 (113, 344)	54.51 (38.97, 78.68)
EAPC (95% *CI*)	2.89 (2.19, 3.59) *	0.56 (0.53, 0.59) *	0.29 (0.17, 0.42) *	18.95 (16.18, 21.78) *	12.57 (10.69, 14.49) *	8.21 (7.81, 8.62) *	-3.59 (-3.94, -3.24) *	-3.52 (-3.85, -3.19) *

*EAPC* estimated annual percentage change; *NDVI* normalized difference vegetation index; *GDP* gross domestic product; *TB* tuberculosis. Data were shown as median (IQR). Trends from 2005 to 2019 were indicated by EAPC. *P < 0.05.

During the study period, the median (IQR) urbanization score of the 1992 counties was 5.13 (3.87–6.97). The highest urbanization score, 88.10, was recorded in Shishi, a county-level city in Fujian, in 2017, while the lowest score, 0.61, was observed in Sajia county, Xizang, in 2010. A total of 1709 counties (85.79%) demonstrated an increase in urbanization factors from 2005 to 2019 (Table S5). The largest growth in urbanization was observed in Jimsar county, Xinjiang, with an average annual increase of 11.08% (EAPC = 11.08, 95% *CI*: 8.65 to 13.55).

### Association between urbanization factors and TB incidence

Urbanization, assessed by a composite index incorporating population density, GDP per capita, the number of hospital beds (per 1000 population), NTL and NDVI, was negatively associated with TB incidence (β = − 0.0114,* P* < 0.001). Specifically, for each additional urbanization score, TB incidence was reduced by 1.1% [incidence rate ratio (IRR) = 0.989, 95% *CI*: 0.986 to 0.992, Table 2]. Notably, we identified a nonlinear pattern through quadratic modeling. Both linear (β = − 1.9734 × 10^-2^,* P* < 0.001) and quadratic (β = 1.8637×10^-4^,* P* < 0.001) terms of urbanization score were statistically significant, revealing a U-shaped relationship with an inflection point at 52.94 urbanization units (Table S6).

**Table 2:** Association of urbanization factors with tuberculosis incidence by fixed-effect models

Urbanization factors	Coefficients	Standard error	*t*-value	IRR (95% *CI*)	*P*-value
Main analysis					
Urbanization score	− 0.0114	0.0016	− 7.1081	0.989 (0.986, 0.992)	< 0.001
PM_2.5_	0.0078	0.0004	21.0779	1.008 (1.007, 1.009)	< 0.001
Temperature	− 0.0014	0.0036	− 0.3729	0.999 (0.992, 1.006)	0.709
Wind speed	0.1165	0.0132	8.8333	1.124 (1.095, 1.153)	< 0.001
Relative humidity	− 0.6419	0.1280	− 5.0133	0.526 (0.409, 0.676)	< 0.001
Sensitivity analysis					
Urbanization score	− 0.0109	0.0015	− 7.0289	0.989 (0.986, 0.992)	< 0.001
PM_2.5_	0.0079	0.0004	21.3566	1.008 (1.007, 1.009)	< 0.001
Temperature	− 0.0011	0.0036	− 0.3108	0.999 (0.992, 1.006)	0.756
Wind speed	0.1175	0.0132	8.9100	1.125 (1.096, 1.154)	< 0.001
Relative humidity	− 0.6456	0.1280	− 5.0424	0.524 (0.408, 0.674)	< 0.001

*PM*_*2.5*_ fine particulate matter, *IRR* incidence rate ratio

**Fig. 1:**
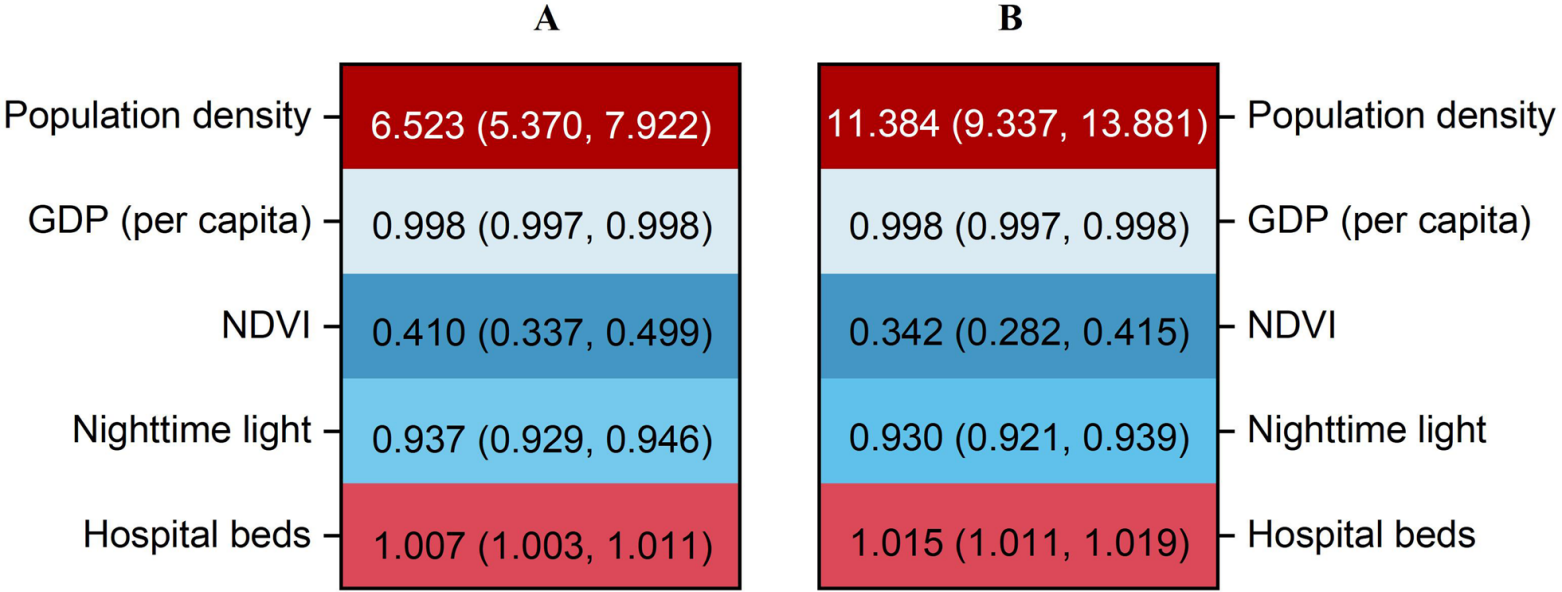
Association of single urbanization indicator with tuberculosis incidence by fixed-effect models. **A** shows the associations between single indicator of urbanization factors with tuberculosis using univariate analysis adjusted by meteorological factors (temperature, wind speed, and relative humidity) and fine particulate matter; **B** shows the associations between single indicator of urbanization factors with tuberculosis using multivariate analysis adjusted by meteorological factors (temperature, wind speed, and relative humidity), fine particulate matter and other urbanization factors

The effects of each single urbanization indicator on TB incidence varied (Figure 1). Both univariate and multivariate analyses revealed that population density and the number of hospital beds were positively associated with TB incidence. In the multivariate analysis, the adjusted IRR for population density was 11.384 (95%* CI*: 9.337 to 13.881), and for the number of hospital beds, it was 1.015 (1.011 to 1.019). In contrast, GDP per capita, NTL, and NDVI were significantly negatively associated with TB incidence. The adjusted IRR were 0.998 (0.997 to 0.998) for GDP, 0.937 (0.929 to 0.946) for NTL, and 0.342 (0.282 to 0.415) for NDVI in the multivariate analysis.

In sensitivity analysis, we constructed a new composite index excluding the number of hospital beds. The results confirmed the robustness of the inverse association between urbanization and TB incidence (IRR = 0.989, 95% *CI*: 0.986 to 0.992). Subgroup analyses by region, categorized according to China’s income policies, further support the role of urbanization factors in reducing TB incidence in eastern and western China (Table 3). However, among counties in central China, each additional urbanization score was connected to a 1.5% increase of TB incidence (IRR = 1.015, 95%* CI*: 1.008 to 1.021).

**Table 3:** Subgroup analysis of the association between urbanization factors and incidence of tuberculosis

Income group	Number of counties	IRR (95% *CI*)	*P*-value
Northeast Region	150	0.986 (0.971, 1.001)	0.064
Eastern Region	477	0.994 (0.991, 0.997)	0.001*
Central Region	492	1.015 (1.008, 1.021)	< 0.001*
Western Region	873	0.976 (0.970, 0.982)	< 0.001*

IRR incidence rate ratio. *P < 0.05

## Discussion

Thirty high TB burden countries are mostly in low- and middle-income regions [1]. Researches have shown that poorly planned urbanization can lead to various environmental and economic challenges, ultimately affecting public health, particularly in LMICs experiencing rapid urban sprawl [9–12]. To our knowledge, this is the first study to evaluate the impact of urbanization on TB risk using data from1992 counties in Chinese mainland. We found that TB incidence increased in 6.17% of counties, primarily in western China, between 2005 and 2019. Urbanization factors were generally associated with a decreased risk of TB incidence, although the associations varied among subgroups of counties with different economic levels.

Consistent with our results, several studies revealed that the reported TB incidence continued to decline in China over the past two decades [2, 30, 31]. The implement of neonatal Bacillus Calmette Guerin vaccination programs, the coordinated efforts of disease control and prevention centers (prevention), hospitals (diagnosis, treatment, reporting), and primary health institutions (management), as well as innovations in TB detection, treatment and care, have all contributed to this decline [2, 30, 31]. However, we also observed increasing TB incidence in certain counties in western China, particularly in Xizang, Qinghai and Sichuan, despite urbanization progress. This suggests that the benefits of urbanization on TB incidence seem not be fully realized in these counties. One possible explanation is the improved ability for TB diagnosis and reporting due to advancements in local healthcare systems, enhanced training for health workers, the implement of active surveillance, and the standardized management of TB patients. A previous study showed that countries with low-level human health resources had a 43% increased risk of mortality from respiratory infections and TB compared to those with the highest levels [30]. Additionally, since the implement of China’s reform and opening-up policies, many individuals living in western China have migrated to eastern coastal cities for work and returned home in middle and old age, potentially increasing TB incidence due to the progression of latent infections into active disease [2].

Numerous studies have indicated that urbanization is related to population health [14, 32]. In high-income countries such as the United States and European countries, research has shown that TB incidence tends to be higher in big cities than in other regions—contrasting with our findings [21, 22]. Several factors could explain this discrepancy. First, urbanization was measured differently. Studies conducted in the United States and European countries defined urbanization using the concept “big cities”, depending on population density [21, 22], whereas our study used a composite index encompassing multiple indicators. Second, the analytical method differed. Those studies calculated rate ratios using TB incidence in big cities as the numerator and the national TB incidence (excluding big city TB cases and population) as a denominator [21, 22], while our study employed fixed-effects models. Third, the impact of urbanization on TB incidence is complex and may differ between high-income countries and LMICs.

Previous studies in China have found that urbanization is linked to lower mortality from hepatitis A and other infectious diseases [17, 33], however, the effects of urbanization on TB incidence follow a non-linear pattern in our study. China, with its rapidly evolving economy, provides an ideal setting to examine the multifaceted effects of urbanization. Some studies suggest a U-shaped relationship between urbanization and health outcomes in China [34, 35]. Specifically, urbanization initially improves health by enhanced access to high-quality health services, abundant knowledge about health, increased individual income, and advanced infrastructure. However, once beyond a certain threshold, urbanization may have detrimental effects due to factors such as air pollution, climate change, and overcrowded, which may undermine its beneficial effects. In our study, most counties in China have not yet reached the optimal threshold, which may explain the observed positive effects of urbanization on TB incidence. Meanwhile, poorly planned urbanization before the inflection point could reverse this U-shaped correlation among LMICs—characterized by inadequate and unprofessional healthcare services with population explosion, leading to an increased TB burden, which is different from our results [36–38].

Interestingly, we found that in central China, urbanization was associated with a slight increase in TB incidence. This could be interpreted by the dual effects of urbanization. On one hand, rapid and unstructured urbanization may have adverse health consequences. “Three bases, one hub” (i.e. grain production base, energy and raw materials base, modern equipment manufacturing and high-tech industrial base and comprehensive transportation hub) are the main development orientation of central region according to Chinese policies. Economic growth in many central counties relies on resources extraction, which may contribute to environmental degradation and health risk. On the other hand, the positive effects of urbanization on TB incidence may be weaker in this region. According to the *2020 China Population Census Yearbook*, four of the six provincial-level administrative divisions (PLADs) in central China had a higher proportion of older adults (aged 60 and above) than the national average (excluding central PLADs) [39]. Since older adults in this region have a higher relative risk of TB than the general population [2], the effect of urbanization on primary health care, especially interventions of TB prevention and control might be less pronounced. Furthermore, during rapid urbanization, the migration of individuals from high-prevalence western regions to central China may contribute to the increased TB incidence.

We also found that population density and the number of hospital beds were positively associated with TB incidence, while GDP, NTL and NDVI were negatively associated. According to the epidemiologic characteristics of TB, rapid growth of population density could make the transmission of pathogen easier. On one hand, reduced personal space and frequent contacts among people and between people and things could facilitate the spread of TB from person to person [40]. On the other hand, population migrating from rural to urban could bring new pathogens into urban area and even increase the potential risk for outbreaks [41]. The positive association between hospital beds and TB incidence may reflect improved detection in counties with well-developed healthcare system [42]. In counties with higher income level, people infected with TB can be detected earlier and more accurately due to advanced healthcare resources, including more professional and higher educated health workers, high-level health facilities, and comprehensive detecting techniques [43]. Another reason for the negative association between hospital beds and TB incidence is that the hospital beds density measurement did not account for spatial distribution [44]. Lower-income counties with lower population density and vast areas of land may have a higher hospital beds density when the number of total hospital beds is similar. NTL serves as a proxy for urban infrastructure, such as traffic roads, buildings, and electricity supply, which can improve living condition and subsequently reduce TB risk [45]. Similarly, greenspace—measured using NDVI— has been linked to better air quality and mental well-being, potentially lowering TB risk [18]. These findings for the relationships between single urbanization indicators and TB incidence underscore the importance of balancing economic growth with environmental sustainability in urban planning.

Urbanization presents an opportunity to address multiple public health challenges and realize the Sustainable Development Goals (SDGs). However, its effects on health are complex and multifaceted. Governments must prioritize well-planned, managed, and financed urban development to maximize the benefits and mitigate the adverse effects [8]. In LMICs, urbanization efforts should go beyond economic growth and infrastructure expansion to incorporate environmental protection. Local governments could integrate NDVI or other greening indicators into urban planning and collaborate with environmental and health authorities to evaluate the construction of new urban areas. Additionally, government should manage urban land expansion carefully and maintain appropriate population densities. In regions with high population density—such as central China and emerging industrial area—regular active TB screening could be organized to mitigate TB transmission.

In this study, we established a comprehensive dataset by collecting accurate data from nation-, province-, and county-level statistical books, constructed a composite index using five indicators representing various domains of urbanization, and used reported TB incidence from TBIMS to develop the analysis. However, this study has several limitations. First, as an ecological study, we cannot ignore the existence of ecological fallacy, where associations observed at the county level may not reflect individual-level relationships. Second, due to the multiple risk factors associated with TB incidence, the models applied in our study was not be adjusted by all potential confounders. Third, as the evaluation of urbanization is still heterogeneous and there are plentiful domains of urbanization, the indicators used to construct our composite urbanization index not fully capture all relevant aspects of urbanization. Forth, we did not test a spatial lag model or spatial error model to account for geographic clustering and regional transmission patterns of TB, which need be examined in future studies.

## Conclusions

In this county-level analysis using national data from 1992 counties between 2005 and 2019, we found that urbanization was associated with a reduction in TB incidence. However, this association was reversed in central China. Among single urbanization factors, population density and hospital beds density were positively associated with TB incidence, while GDP, NTL and NDVI were negatively associated. In the process of urbanization, governments should adopt integrated strategies to promote well-planned, managed, and financed cities to realize the targets of End TB Strategy.

## Supplementary Information

Additional file 1.

## Data Availability

The data and code used to generate the results in this study are available from the authors upon reasonable request.

## Abbreviations

TB: Tuberculosis
COVID-19: Coronavirus disease 2019
WHO: World health organization
LMICs: Low-and middle-income countries
NDVI: Normalized difference vegetation index
GDP: Gross domestic product
NTL: Nighttime light
*CI*: Confidence interval
MDR-TB: Multi-drug resistant TB
TBIMS: Tuberculosis information management system
WRF: Weather research and forecasting
CMAQ: Community multiscale air quality
EDGAR: Emissions database for global atmospheric research data
MEGAN: Model of emissions of gases and aerosols from nature
EAPC: Estimated annual percentage change
PLADs: Provincial-level administrative divisions
SDGs: Sustainable development goals
