# Supplementary material for: Association of urbanization-related factors with tuberculosis incidence among 1992 counties in China from 2005 to 2019: a nationwide observational study
Source: Infect Dis Poverty. 2025 Apr 25;14:30. doi: 10.1186/s40249-025-01299-4 (PMC12023368; doi:10.1186/s40249-025-01299-4)
Supplement: Supplementary file 1 — Additional file 1. [file 40249_2025_1299_MOESM1_ESM.docx]

Supplementary materials

**Association of urbanization-related factors with tuberculosis incidence among 1992 counties in China from 2005 to 2019: a nationwide observational study**

**Authors:** Yaping Wang, Xiaoqiu Liu, Yuhong Li, Min Liu, Yiheng Wang, Hongliang Zhang, Jue Liu, Yanlin Zhao

[**Content**](#_Toc174108434)

[Table S1. Steps of Entropy evaluation method 1](#_Toc174108435)

[Table S2. Annual weights of indicators for the composite index of urbanization factors using entropy evaluation method 2](#_Toc174108436)

[Methods for data of meteorological factors and PM_2.5_ 3](#_Toc174108437)

[Table S3. Counties grouped by economic development at provincial level^16^ 6](#_Toc174108438)

[Table S4. The estimated annual percentage change (EAPC) of tuberculosis incidence from 2005 to 2019 9](#_Toc174108439)

[Table S5. The estimated annual percentage change (EAPC) of urbanization score from 2005 to 2019 56](#_Toc174108440)

# Table S1. Steps of Entropy evaluation method

| **Step** | **Function** | **Description** |
| --- | --- | --- |
| Step 1 | $X_{ij}^{'}=\frac{X_{ij}-min(X_{j})}{\max\left( X_{j} \right)- min(X_{j})}$ | $X_{ij}$ is the original value of the *j*-th indicator of the *i*-th study county, $X_{ij}^{'}$ is the standardized value of the indicator, and min ($X_{j}$) and max ($X_{j}$) is the minimum and maximum values of the *j*-th indicator. |
| Step 2 | $p_{ij}=\frac{X_{ij}^{'}}{\sum_{i=1}^{n} X_{ij}^{'}}$ | $p_{ij}$ is the proportion of the *i*-th study county under the *j*-th indicator in the indicator. n is the total number of counties. |
| Step 3 | $e_{j}=-k\sum_{i=1}^{n} p_{ij}\ln\left( p_{ij} \right)$ $k=\frac{1}{ln(n)}$ | $e_{j}$is the entropy value of the *j*-th indicator. |
| Step 4 | $d_{j}=1-e_{j}$ | $d_{j}$is the diversity factor or information utility value of the *j*-th indicator. |
| Step 5 | $w_{j}=\frac{d_{j}}{\sum_{j=1}^{m} d_{j}}$ | $w_{j}$is the weight value of the *j*-th indicator. |
| Step 6 | Repeat steps 1 to 5 for all indicators in each year during 2005–2019. | |

# Table S2. Annual weights of indicators for the composite index of urbanization factors using entropy evaluation method

| **Year** | **Population density (per square kilometers)** | **GDP (per capita)** | **Number of hospital beds (per 1000 population)** | **Nighttime light** | **NDVI** |
| --- | --- | --- | --- | --- | --- |
| 2005 | 0.18 | 0.14 | 0.08 | 0.57 | 0.03 |
| 2006 | 0.17 | 0.14 | 0.08 | 0.58 | 0.03 |
| 2007 | 0.17 | 0.15 | 0.07 | 0.59 | 0.03 |
| 2008 | 0.17 | 0.14 | 0.06 | 0.60 | 0.03 |
| 2009 | 0.20 | 0.17 | 0.05 | 0.55 | 0.03 |
| 2010 | 0.19 | 0.15 | 0.06 | 0.58 | 0.03 |
| 2011 | 0.19 | 0.14 | 0.05 | 0.59 | 0.03 |
| 2012 | 0.20 | 0.15 | 0.07 | 0.56 | 0.03 |
| 2013 | 0.22 | 0.16 | 0.05 | 0.55 | 0.03 |
| 2014 | 0.21 | 0.15 | 0.07 | 0.54 | 0.03 |
| 2015 | 0.21 | 0.14 | 0.06 | 0.55 | 0.03 |
| 2016 | 0.22 | 0.14 | 0.04 | 0.56 | 0.03 |
| 2017 | 0.24 | 0.13 | 0.04 | 0.56 | 0.03 |
| 2018 | 0.24 | 0.14 | 0.04 | 0.55 | 0.03 |
| 2019 | 0.24 | 0.13 | 0.04 | 0.55 | 0.03 |

Note: GDP, gross domestic product; NDVI, normalized difference vegetation index.

# Methods for data of meteorological factors and PM_2.5_

1. **Meteorological data sources**

The meteorological data utilized in this study originates from simulations performed using the Weather Research and Forecasting model (WRF) version 4.1.2. The model applied the Final (FNL) Operational Global Analysis data from the National Centers for Environmental Prediction (NCEP) as meteorological input, with a spatial resolution of 1.0°×1.0° (<https://rda.ucar.edu/datasets/ds083.3/>, last accessed on March 2, 2023). The simulation period spans 15 years from 2005 to 2019, configured on a 197×127 grid with a horizontal resolution of 36 km × 36 km. Table A presents the detailed parameterization schemes of the WRF model.

Preprocessing of the meteorological data involved extracting WRF model output for each county-level administrative center in China based on their annual latitude and longitude coordinates from the original grid. Each county-level region corresponds to one grid cell's data. Subsequently, hourly data for each county were averaged to weekly data. For weeks with fewer than seven days of data, averages were computed based on the actual number of days available. Then, we generated annual average data using weekly average data for each county.

1. **Pollutant concentration data sources**

To enhance the simulation performance, this study employed Community Multiscale Air Quality (CMAQ) version 5.0.2 with an improved SAPRC-11 photochemical mechanism,^1-3^ simulating the concentration changes of PM_2.5_ in China from 2005 to 2019. The aforementioned WRF v4.1.2 was utilized to generate meteorology inputs. Anthropogenic emissions from 2008 to 2019 were sourced from the Multi-resolution Emission Inventory for China (MEIC) (<http://www.meicmodel.org/>, last accessed on July 2, 2023).^4^ Given the absence of emissions data in the previous MEIC v1.3 inventory before 2008, estimates for the years 2005–2007 were derived in this study based on emission ratios using the Emissions Database for Global Atmospheric Research data (EDGAR; <https://edgar.jrc.ec.europa.eu/;> last accessed in Jul. 2023).^5^ Both MEIC and EDGAR anthropogenic emissions encompass multiple sectors including power, industry, residential transportation and agriculture. Biogenic emissions were sourced from the Model of Emissions of Gases and Aerosols from Nature (MEGAN) v2.1.^6^ The processing of emission data in the model followed the approach by Wang et al..^7^ The Lambert projection was used to convert the original latitude and longitude data into emissions for each grid point (36 km) to fit the model input. Consistent with the WRF model, CMAQ also utilized a 197×127 grid configuration with a horizontal resolution of 36 km × 36 km, and the vertical grid was divided into 18 sigma levels, extending from the surface to ~20 km altitude. The first three days of model simulations were excluded to avoid the influence of initial conditions on subsequent predictions. Table B presents the detailed configuration of the CMAQ model.

Similarly, the preprocessing of pollutant concentration data was consistent with the method used for meteorological data. However, before obtaining the weekly county-level data, the hourly results from the CMAQ model were first averaged to daily values.

1. **Observational Data and Model Validation**

We validated the simulation results using observational data of meteorological conditions and pollutant concentrations. The meteorological observations including temperature at 2 m (T2), relative humidity (RH), wind speed (WS), and wind direction (WD) at 10 m were from the National Climatic Data Center (NCDC; <https://www.ncdc.noaa.gov/>, last accessed April 17, 2022). This dataset provided the meteorological observations at around 1,200 stations for the period from 2000 to 2020. The hourly concentrations of pollutants were validated using datasets from the China National Environmental Monitoring Center (CNEMC; <http://www.cnemc.cn/>, last accessed August 15, 2023). This dataset provided measurements from 1600 sites across the country from 2014 to 2020.

Our previous article have detailed the validation results of the WRF-CMAQ model.^8^ The results indicate that the WRF simulation results show slight deviations from the standard, primarily due to the relatively coarse 36 km horizontal resolution. Nonetheless, the model's performance is very close to that of previous WRF models and can provide accurate meteorological inputs for CMAQ.^9-12^ The performance of the CMAQ model in simulating pollutant concentrations was evaluated using the criteria proposed by Emery et al. and EPA.^13-15^ Comparison with observations shows that CMAQ effectively simulates the variations in pollutant concentrations. Overall, the model's simulation results are satisfactory.

**Table A. WRF scheme set-up**

| Microphysics | Thompson |
| --- | --- |
| Longwave Radiation | RRTM |
| Shortwave Radiation | Goddard |
| Surface Layer | Monin-Obukhov |
| Land Surface | Noah |
| Cumulus Parameterization | Grell-Devenyi |
| Planetary Boundary Layer | YSU |

**Table B. CMAQ configurations and inventories input**

| Simulation time | 2005–2019 |
| --- | --- |
| Chemical transport model | CMAQ v5.0.2 |
| meteorological model | WRF v4.1.2 |
| resolution | 36km × 36 km |
| chemical mechanism | SAPRC11 |
| aerosol mechanism | AERO6 |
| Meteorological inputs | FNL, 1.0°× 1.0° |
| Anthropogenic emissions | MEIC v1.3 (2008–2019)  EDGAR v5.1 (2005–2007) |
| Biogenic emissions | MEGAN 2.1 |

# Table S3. Counties grouped by economic development at provincial level^16^

| **Subgroup** | **Provincial-level administrative division** |
| --- | --- |
| Northeast region | Liaoning, Jilin, and Heilongjiang |
| Eastern region | Beijing, Tianjin, Hebei, Shanghai, Jiangsu, Zhejiang, Fujian, Shandong, Guangdong, and Hainan. |
| Central region | Shanxi, Anhui, Jiangxi, Henan, Hubei, and Hunan |
| Western region | Inner Mongolia, Guangxi, Chongqing, Sichuan, Guizhou, Yunnan, Xizang, Shaanxi, Gansu, Qinghai, Ningxia, and Xinjiang |

**Reference:**

1. Ying, Q.; Li, J.; Kota, S. H., Significant contributions of isoprene to summertime secondary organic aerosol in eastern United States. Environmental science & technology 2015, 49 (13), 7834-7842.
2. Hu, J.; Wu, L.; Zheng, B.; Zhang, Q.; He, K.; Chang, Q.; Li, X.; Yang, F.; Ying, Q.; Zhang, H., Source contributions and regional transport of primary particulate matter in China. Environmental pollution 2015, 207, 31-42.
3. Carter, W. P. L.; Heo, G., Development of revised SAPRC aromatics mechanisms. Atmospheric environment 2013, 77, 404-414.
4. Zheng, B.; Tong, D.; Li, M.; Liu, F.; Hong, C.; Geng, G.; Li, H.; Li, X.; Peng, L.; Qi, J., Trends in China's anthropogenic emissions since 2010 as the consequence of clean air actions. Atmospheric Chemistry and Physics 2018, 18 (19), 14095-14111.
5. Crippa, M.; Solazzo, E.; Huang, G.; Guizzardi, D.; Koffi, E.; Muntean, M.; Schieberle, C.; Friedrich, R.; Janssens-Maenhout, G., High resolution temporal profiles in the Emissions Database for Global Atmospheric Research. Scientific data 2020, 7 (1), 121.
6. Guenther, A. B.; Jiang, X.; Heald, C. L.; Sakulyanontvittaya, T.; Duhl, T. a.; Emmons, L. K.; Wang, X., The Model of Emissions of Gases and Aerosols from Nature version 2.1 (MEGAN2. 1): an extended and updated framework for modeling biogenic emissions. Geoscientific Model Development 2012, 5 (6), 1471-1492.
7. Wang, D.; Hu, J.; Xu, Y.; Lv, D.; Xie, X.; Kleeman, M.; Xing, J.; Zhang, H.; Ying, Q., Source contributions to primary and secondary inorganic particulate matter during a severe wintertime PM2. 5 pollution episode in Xi'an, China. Atmospheric environment 2014, 97, 182-194.
8. Zhang, R.; Zhu, S.; Zhang, Z.; Zhang, H.; Tian, C.; Wang, S.; Wang, P.; Zhang, H., Long-term variations of air pollutants and public exposure in China during 2000–2020. Science of The Total Environment 2024, 930, 172606.
9. Shao, T.; Wang, P.; Yu, W.; Gao, Y.; Zhu, S.; Zhang, Y.; Hu, D.; Zhang, B.; Zhang, H., Drivers of alleviated PM2. 5 and O3 concentrations in China from 2013 to 2020. Resources, Conservation and Recycling 2023, 197, 107110.
10. Mao, J.; Li, L.; Li, J.; Sulaymon, I. D.; Xiong, K.; Wang, K.; Zhu, J.; Chen, G.; Ye, F.; Zhang, N., Evaluation of long-term modeling fine particulate matter and ozone in China during 2013–2019. Frontiers in Environmental Science 2022, 10, 872249.
11. Hu, J.; Chen, J.; Ying, Q.; Zhang, H., One-year simulation of ozone and particulate matter in China using WRF/CMAQ modeling system. Atmospheric Chemistry and Physics 2016, 16 (16), 10333-10350.
12. Qiao, X.; Guo, H.; Tang, Y.; Wang, P.; Deng, W.; Zhao, X.; Hu, J.; Ying, Q.; Zhang, H., Local and regional contributions to fine particulate matter in the 18 cities of Sichuan Basin, southwestern China. Atmospheric Chemistry and Physics 2019, 19 (9), 5791-5803.
13. Emery, C.; Tai, E.; Yarwood, G., Enhanced meteorological modeling and performance evaluation for two Texas ozone episodes. Prepared for the Texas natural resource conservation commission, by ENVIRON International Corporation 2001, 161.
14. Epa, U. S. Guidance on the Use of Models and Other Analyses in Attainment Demonstrations for the 8-hour Ozone NAAQS; EPA-454/R-05-002: 2005.
15. Epa, U., Guidance on the use of models and other analyses for demonstrating attainment of air quality goals for ozone, PM2. 5, and regional haze. US Environmental Protection Agency, Office of Air Quality Planning and Standards 2007.
16. National Bureau of Statistics of China. Methods of division of eastern, western, central, and northeast regions. 2011. https://www.stats.gov.cn/zt_18555/zthd/sjtjr/dejtjkfr/tjkp/202302/t20230216_1909741.htm

# Table S4. The estimated annual percentage change (EAPC) of tuberculosis incidence from 2005 to 2019

| **Name** | **Province** | **EAPC (%, 95% CI)** | | | |
| --- | --- | --- | --- | --- | --- |
|  |  | **2005-2019** | **2005-2009** | **2010-2014** | **2015-2019** |
| Changfeng | Anhui | -0.60 (-2.08, 0.91) | 3.88 (-11.29, 21.64) | 0.19 (-2.43, 2.88) | -2.56 (-6.96, 2.06) |
| Chaohu | Anhui | -3.52 (-5.32, -1.68) | -0.93 (-15.73, 16.47) | 2.08 (-14.23, 21.48) | -3.11 (-7.76, 1.77) |
| Dangshan | Anhui | -2.43 (-4.48, -0.34) | -6.37 (-17.88, 6.76) | 6.49 (-11.05, 27.49) | -7.89 (-20.51, 6.73) |
| Dangtu | Anhui | -3.54 (-4.75, -2.33) | -1.09 (-7.48, 5.76) | 3.16 (-2.65, 9.33) | -8.11 (-14.98, -0.69) |
| Dingyuan | Anhui | -5.46 (-6.80, -4.09) | -4.67 (-18.02, 10.86) | -4.76 (-16.42, 8.53) | -2.71 (-11.99, 7.55) |
| Dongzhi | Anhui | -3.80 (-7.20, -0.28) | -6.79 (-19.18, 7.50) | -4.85 (-13.57, 4.74) | 5.99 (-23.28, 46.43) |
| Fanchang | Anhui | -8.84 (-10.77, -6.88) | -3.70 (-18.81, 14.22) | -4.44 (-16.04, 8.76) | 2.09 (-4.02, 8.60) |
| Feidong | Anhui | -5.58 (-7.46, -3.66) | -8.68 (-26.15, 12.92) | 3.99 (-8.72, 18.47) | -9.21 (-14.14, -3.98) |
| Feixi | Anhui | -5.81 (-7.23, -4.37) | -14.89 (-21.24, -8.02) | -1.28 (-4.98, 2.56) | -9.85 (-13.88, -5.64) |
| Fengtai | Anhui | -3.49 (-4.45, -2.51) | -4.79 (-14.47, 5.98) | -2.47 (-8.94, 4.45) | 0.49 (-4.89, 6.17) |
| Fengyang | Anhui | -2.22 (-3.52, -0.91) | 2.75 (-2.43, 8.22) | -8.21 (-15.37, -0.43) | -7.09 (-14.08, 0.47) |
| Funan | Anhui | -4.53 (-6.53, -2.49) | -6.14 (-25.04, 17.53) | 4.18 (1.15, 7.30) | -6.16 (-20.06, 10.15) |
| Guangde | Anhui | -5.42 (-7.68, -3.11) | -12.51 (-27.74, 5.93) | 3.96 (-4.09, 12.69) | 0.33 (-5.92, 6.99) |
| Guzhen | Anhui | -4.02 (-6.14, -1.85) | 5.55 (-16.75, 33.82) | -10.51 (-14.8, -6.01) | 3.18 (-4.60, 11.59) |
| Hanshan | Anhui | -6.51 (-8.69, -4.28) | -2.33 (-13.44, 10.21) | -11.87 (-24.35, 2.66) | 5.01 (-11.00, 23.90) |
| He | Anhui | -5.10 (-6.68, -3.48) | -4.63 (-19.41, 12.85) | -6.78 (-16.66, 4.28) | -1.90 (-12.11, 9.50) |
| Huaining | Anhui | -2.97 (-4.93, -0.97) | -8.35 (-21.06, 6.41) | 9.23 (0.20, 19.08) | -1.27 (-10.70, 9.15) |
| Huaiyuan | Anhui | -3.99 (-5.92, -2.02) | -9.01 (-18.73, 1.88) | -2.75 (-18.17, 15.59) | -4.14 (-23.84, 20.67) |
| Huoqiu | Anhui | -2.52 (-3.77, -1.24) | -1.41 (-11.73, 10.13) | 2.81 (-9.86, 17.25) | -0.79 (-5.35, 3.99) |
| Huoshan | Anhui | -4.72 (-6.91, -2.47) | -8.08 (-21.11, 7.12) | 2.75 (-14.78, 23.90) | -4.31 (-18.13, 11.85) |
| Jieshou | Anhui | -3.17 (-5.19, -1.11) | -7.56 (-24.15, 12.65) | 0.38 (-5.68, 6.84) | -14.49 (-21.29, -7.11) |
| Jing | Anhui | -6.39 (-8.67, -4.05) | -12.97 (-31.74, 10.96) | -3.25 (-20.95, 18.42) | -0.25 (-14.68, 16.63) |
| Jingde | Anhui | -1.02 (-3.64, 1.67) | -14.15 (-20.57, -7.21) | 12.53 (-3.83, 31.67) | 2.82 (-12.60, 20.95) |
| Jinzhai | Anhui | 0.32 (-2.17, 2.89) | 12.76 (-14.74, 49.12) | 7.35 (-2.17, 17.79) | -2.24 (-11.93, 8.51) |
| Jixi | Anhui | -1.25 (-3.67, 1.23) | -11.67 (-28.42, 8.98) | 6.68 (-4.13, 18.7) | 2.94 (-13.64, 22.72) |
| Laian | Anhui | -5.06 (-6.55, -3.55) | -5.55 (-18.97, 10.10) | -5.80 (-11.21, -0.07) | -11.42 (-21.43, -0.13) |
| Langxi | Anhui | -6.00 (-7.71, -4.26) | -10.06 (-26.13, 9.51) | -4.84 (-12.99, 4.08) | -1.07 (-9.08, 7.65) |
| Lingbi | Anhui | -2.68 (-4.60, -0.72) | 1.81 (-19.21, 28.30) | 1.57 (-4.07, 7.55) | -9.93 (-19.53, 0.82) |
| Linquan | Anhui | -4.39 (-6.42, -2.32) | -12.63 (-21.66, -2.57) | 3.44 (-3.52, 10.91) | -5.08 (-12.88, 3.41) |
| Lixin | Anhui | -2.92 (-3.94, -1.89) | -3.71 (-8.73, 1.58) | -0.90 (-14.09, 14.31) | -3.42 (-7.04, 0.34) |
| Lujiang | Anhui | -3.29 (-4.87, -1.69) | -3.14 (-17.80, 14.13) | 4.11 (-3.35, 12.14) | -0.66 (-12.50, 12.8) |
| Mengcheng | Anhui | -1.65 (-2.88, -0.42) | -2.33 (-16.17, 13.81) | 1.63 (-4.82, 8.52) | -2.02 (-11.14, 8.03) |
| Mingguang | Anhui | -2.63 (-4.65, -0.56) | -10.06 (-26.59, 10.21) | 6.15 (4.07, 8.27) | -4.85 (-14.50, 5.90) |
| Nanling | Anhui | -6.47 (-7.69, -5.24) | -9.62 (-17.88, -0.53) | -3.25 (-12.79, 7.33) | -0.93 (-7.88, 6.56) |
| Ningguo | Anhui | -4.11 (-6.16, -2.02) | -9.37 (-17.91, 0.07) | -0.47 (-6.78, 6.27) | 0.13 (-5.73, 6.35) |
| Qianshan | Anhui | -4.51 (-6.27, -2.72) | -0.14 (-12.25, 13.65) | -3.07 (-14.82, 10.30) | -12.16 (-17.23, -6.78) |
| Qimen | Anhui | -2.70 (-4.22, -1.15) | -0.33 (-13.48, 14.82) | 1.02 (-4.91, 7.32) | 3.10 (-6.17, 13.28) |
| Qingyang | Anhui | -2.99 (-5.40, -0.51) | 3.20 (-0.82, 7.38) | -5.13 (-21.29, 14.35) | -13.64 (-32.32, 10.20) |
| Quanjiao | Anhui | -4.00 (-6.21, -1.73) | 14.50 (5.98, 23.72) | -4.73 (-9.67, 0.48) | -7.02 (-14.82, 1.50) |
| She | Anhui | -5.87 (-7.71, -4.00) | -6.51 (-21.24, 10.98) | 4.91 (-5.71, 16.74) | -5.18 (-11.93, 2.09) |
| Shitai | Anhui | -5.59 (-8.51, -2.58) | -2.27 (-15.85, 13.52) | -3.14 (-14.57, 9.83) | 14.50 (-11.20, 47.64) |
| Shou | Anhui | -3.28 (-5.17, -1.36) | -8.08 (-16.57, 1.29) | 10.86 (0.64, 22.12) | -2.20 (-9.68, 5.90) |
| Shucheng | Anhui | -2.89 (-4.26, -1.50) | -7.89 (-14.05, -1.30) | 4.08 (-6.02, 15.26) | 0.04 (-7.24, 7.89) |
| Si | Anhui | -4.27 (-5.71, -2.82) | -8.61 (-18.18, 2.07) | 2.47 (-4.85, 10.36) | -4.53 (-10.74, 2.12) |
| Suixi | Anhui | -2.70 (-4.99, -0.35) | -10.48 (-35.51, 24.26) | -2.65 (-13.2, 9.17) | -3.59 (-7.71, 0.72) |
| Taihe | Anhui | -5.06 (-6.23, -3.88) | -3.77 (-12.34, 5.65) | -3.61 (-7.00, -0.11) | -11.27 (-19.44, -2.28) |
| Taihu | Anhui | -1.18 (-5.11, 2.92) | 0.68 (-14.53, 18.61) | 20.89 (6.01, 37.85) | -21.50 (-40.12, 2.91) |
| Tianchang | Anhui | -2.91 (-4.17, -1.63) | -2.89 (-14.70, 10.56) | -1.90 (-7.22, 3.74) | -0.04 (-7.82, 8.39) |
| Tongcheng | Anhui | -4.29 (-5.96, -2.60) | -3.39 (-23.23, 21.58) | -8.04 (-15.74, 0.37) | -8.44 (-11.69, -5.08) |
| Wangjiang | Anhui | -3.71 (-6.56, -0.76) | -6.63 (-16.15, 3.96) | 8.33 (-16.69, 40.86) | -17.95 (-28.94, -5.25) |
| Woyang | Anhui | -5.70 (-7.89, -3.45) | -8.46 (-26.34, 13.75) | 0.77 (-9.45, 12.13) | -3.05 (-10.98, 5.59) |
| Wuhe | Anhui | -2.37 (-4.59, -0.10) | 12.70 (-8.69, 39.10) | -5.87 (-12.35, 1.07) | -0.21 (-10.17, 10.87) |
| Wuhu | Anhui | -4.52 (-6.24, -2.77) | -7.84 (-26.07, 14.89) | -9.94 (-19.02, 0.16) | -3.32 (-9.17, 2.91) |
| Wuwei | Anhui | -2.10 (-4.54, 0.41) | -9.35 (-20.25, 3.04) | 3.53 (-3.19, 10.72) | -8.96 (-22.33, 6.71) |
| Xiao | Anhui | -3.63 (-6.11, -1.09) | -12.59 (-29.94, 9.07) | 8.43 (-4.74, 23.42) | -8.50 (-14.65, -1.91) |
| Xiuning | Anhui | -3.23 (-5.15, -1.26) | -3.42 (-17.78, 13.45) | 4.09 (-9.46, 19.65) | -0.81 (-8.60, 7.63) |
| Xiusong | Anhui | -6.42 (-7.44, -5.39) | -1.67 (-10.29, 7.78) | -7.22 (-12.77, -1.31) | -10.12 (-15.23, -4.70) |
| Yi | Anhui | -4.32 (-6.99, -1.56) | -8.80 (-31.35, 21.16) | 2.75 (-9.98, 17.29) | -5.94 (-20.60, 11.43) |
| Yian | Anhui | -3.48 (-5.83, -1.07) | -0.82 (-13.41, 13.60) | 0.53 (-4.46, 5.78) | -10.28 (-15.95, -4.23) |
| Yingshang | Anhui | -4.78 (-6.79, -2.73) | 0.24 (-18.91, 23.91) | -1.50 (-17.12, 17.06) | -10.09 (-19.17, 0.01) |
| Yuexi | Anhui | -0.90 (-3.11, 1.36) | -12.75 (-23.22, -0.86) | 6.87 (-4.15, 19.15) | -4.29 (-18.81, 12.84) |
| Zongyang | Anhui | -5.76 (-8.65, -2.78) | -6.87 (-27.54, 19.69) | 8.17 (-19.24, 44.89) | -11.96 (-15.56, -8.21) |
| Miyun | Beijing | -1.71 (-2.92, -0.49) | -6.39 (-13.76, 1.62) | 1.13 (-7.63, 10.71) | -0.94 (-10.05, 9.09) |
| Yanqing | Beijing | -1.81 (-3.44, -0.16) | -1.06 (-11.85, 11.05) | 2.59 (-13.22, 21.28) | -2.42 (-13.39, 9.95) |
| Bishan | Chongqing | -5.88 (-8.28, -3.41) | -13.60 (-30.89, 8.02) | 6.28 (-5.55, 19.60) | -2.02 (-16.85, 15.46) |
| Chengkou | Chongqing | 0.71 (-0.42, 1.85) | -1.84 (-13.96, 11.98) | 0.48 (-7.42, 9.06) | 3.76 (0.49, 7.12) |
| Dazu | Chongqing | -5.36 (-7.77, -2.88) | -11.02 (-26.90, 8.30) | 6.30 (-10.02, 25.59) | -12.43 (-26.22, 3.95) |
| Dianjiang | Chongqing | -4.06 (-6.10, -1.97) | -13.83 (-18.24, -9.18) | 4.23 (-12.35, 23.95) | -1.28 (-12.08, 10.83) |
| Fengdou | Chongqing | -4.70 (-6.92, -2.42) | -9.19 (-25.17, 10.20) | -3.07 (-16.10, 11.98) | 6.27 (-1.53, 14.68) |
| Fengjie | Chongqing | -3.65 (-6.67, -0.52) | -19.17 (-29.82, -6.91) | -1.09 (-10.15, 8.88) | 3.16 (-6.31, 13.58) |
| Hechuan | Chongqing | -5.80 (-7.69, -3.86) | -8.98 (-21.98, 6.20) | -1.75 (-6.53, 3.28) | -0.70 (-8.68, 7.98) |
| Jiangjin | Chongqing | -1.57 (-3.33, 0.21) | -5.93 (-12.05, 0.61) | -9.17 (-12.36, -5.87) | 4.20 (-13.97, 26.20) |
| Kaizhou | Chongqing | -1.62 (-2.91, -0.31) | -2.16 (-18.89, 18.03) | -0.57 (-8.25, 7.77) | -0.40 (-5.97, 5.49) |
| Liangping | Chongqing | -5.48 (-6.88, -4.06) | -13.59 (-24.15, -1.55) | -4.59 (-13.13, 4.78) | -6.89 (-12.13, -1.34) |
| Nanchuan | Chongqing | -6.52 (-7.74, -5.28) | -3.56 (-18.08, 13.54) | -6.74 (-10.95, -2.33) | -3.69 (-12.74, 6.30) |
| Pengshui | Chongqing | 0.03 (-3.79, 4.00) | 5.10 (-21.77, 41.20) | -10.84 (-21.44, 1.18) | 14.72 (10.43, 19.17) |
| Qijiang | Chongqing | -1.57 (-2.46, -0.67) | -1.52 (-10.44, 8.29) | 1.88 (-0.98, 4.82) | -3.65 (-10.60, 3.84) |
| Rongchang | Chongqing | -7.42 (-8.94, -5.88) | -13.49 (-24.87, -0.39) | -6.43 (-12.50, 0.07) | -6.72 (-18.13, 6.28) |
| Shizhu | Chongqing | -4.85 (-6.20, -3.47) | -6.61 (-12.36, -0.49) | -1.08 (-13.43, 13.03) | -8.91 (-16.33, -0.82) |
| Tongliang | Chongqing | -5.29 (-6.70, -3.86) | -10.97 (-20.91, 0.22) | -2.85 (-14.68, 10.62) | -0.63 (-5.08, 4.02) |
| Tongnan | Chongqing | -5.56 (-7.48, -3.61) | -11.37 (-21.99, 0.69) | 4.22 (-13.98, 26.27) | -4.86 (-9.71, 0.25) |
| Wulong | Chongqing | -1.56 (-3.90, 0.83) | -3.99 (-20.94, 16.58) | -4.12 (-8.74, 0.74) | 10.13 (2.71, 18.09) |
| Wushan | Chongqing | -5.36 (-6.78, -3.91) | -4.59 (-12.67, 4.24) | -7.55 (-12.12, -2.75) | -5.80 (-17.89, 8.08) |
| Wuxi | Chongqing | -3.27 (-4.80, -1.71) | -8.19 (-19.46, 4.66) | -2.29 (-8.11, 3.90) | 2.51 (-9.53, 16.15) |
| Xiushan | Chongqing | -1.29 (-3.67, 1.16) | 4.87 (-5.20, 16.02) | -5.67 (-12.04, 1.16) | 8.14 (-15.24, 37.97) |
| Yongchuan | Chongqing | -4.02 (-5.54, -2.47) | -9.13 (-20.85, 4.33) | -3.61 (-11.62, 5.14) | 1.61 (-6.58, 10.52) |
| Youyang | Chongqing | -2.48 (-3.97, -0.97) | -3.68 (-13.44, 7.19) | 4.50 (-2.77, 12.31) | -5.79 (-15.09, 4.53) |
| Yunyang | Chongqing | -5.31 (-6.97, -3.63) | -12.18 (-22.89, 0.02) | 2.28 (-9.28, 15.31) | -9.26 (-15.42, -2.64) |
| Zhong | Chongqing | -3.20 (-5.41, -0.93) | -6.97 (-24.24, 14.25) | 1.80 (-5.64, 9.83) | -13.20 (-26.86, 3.01) |
| Anxi | Fujian | -3.80 (-5.49, -2.09) | -9.83 (-21.51, 3.59) | 1.42 (-5.86, 9.27) | -1.53 (-10.04, 7.78) |
| Changle | Fujian | -7.24 (-8.45, -6.02) | -8.90 (-18.53, 1.86) | -10.53 (-18.52, -1.76) | -2.21 (-9.73, 5.95) |
| Changtai | Fujian | -4.91 (-6.44, -3.35) | 3.27 (-6.88, 14.52) | 0.52 (-6.12, 7.64) | -8.19 (-13.05, -3.05) |
| Changting | Fujian | -5.92 (-7.81, -3.99) | -7.58 (-18.11, 4.32) | -3.83 (-14.54, 8.22) | 1.18 (-1.97, 4.44) |
| Datian | Fujian | -3.80 (-5.24, -2.34) | -8.56 (-16.65, 0.31) | 4.66 (-4.57, 14.78) | -3.56 (-12.69, 6.52) |
| Dehua | Fujian | -3.57 (-6.81, -0.22) | 1.14 (-2.10, 4.47) | 0.01 (-33.06, 49.42) | 3.67 (-9.76, 19.10) |
| Dongshan | Fujian | -6.48 (-9.62, -3.23) | -8.13 (-34.04, 27.94) | -5.79 (-13.41, 2.50) | 10.21 (3.13, 17.79) |
| Fuan | Fujian | -10.63 (-13.02, -8.18) | -9.35 (-27.50, 13.36) | -10.71 (-18.57, -2.10) | 3.61 (-9.16, 18.17) |
| Fuding | Fujian | -7.47 (-9.75, -5.14) | -7.14 (-23.90, 13.31) | -9.09 (-12.13, -5.95) | 5.49 (-0.60, 11.95) |
| Fuqing | Fujian | -3.42 (-5.22, -1.59) | 0.62 (-6.24, 7.98) | -8.23 (-10.09, -6.34) | 5.97 (-3.46, 16.32) |
| Guangze | Fujian | -2.81 (-4.86, -0.72) | -12.42 (-25.42, 2.84) | 2.57 (-9.95, 16.83) | -2.04 (-6.42, 2.55) |
| Gutian | Fujian | -6.94 (-9.20, -4.62) | -13.80 (-25.47, -0.31) | -4.58 (-21.66, 16.22) | -0.77 (-21.75, 25.83) |
| Huaan | Fujian | -5.83 (-9.59, -1.91) | -13.16 (-29.34, 6.74) | -5.66 (-15.46, 5.28) | 17.50 (4.83, 31.70) |
| Huian | Fujian | -8.02 (-10.04, -5.97) | 0.78 (-9.80, 12.60) | -2.81 (-6.73, 1.27) | -11.66 (-20.20, -2.21) |
| Jiangle | Fujian | -3.15 (-5.78, -0.45) | -9.28 (-18.99, 1.59) | -6.55 (-18.67, 7.37) | 7.12 (-14.64, 34.42) |
| Jianning | Fujian | -6.12 (-9.05, -3.10) | -19.28 (-26.16, -11.77) | 0.09 (-11.66, 13.41) | -3.17 (-17.11, 13.12) |
| Jianou | Fujian | -4.48 (-6.33, -2.59) | -10.13 (-23.25, 5.22) | -4.02 (-12.44, 5.21) | 3.39 (-3.79, 11.11) |
| Jianyang | Fujian | -2.51 (-4.62, -0.34) | -9.91 (-22.10, 4.19) | 4.72 (-4.71, 15.09) | -2.63 (-10.58, 6.03) |
| Jinjiang | Fujian | -9.06 (-11.37, -6.69) | -1.00 (-18.63, 20.45) | -14.21 (-31.50, 7.44) | -5.62 (-16.61, 6.81) |
| Liancheng | Fujian | -3.02 (-5.56, -0.41) | -15.01 (-30.22, 3.52) | -5.39 (-10.89, 0.46) | 0.97 (-18.53, 25.13) |
| Lianjiang | Fujian | -6.06 (-7.45, -4.64) | -9.18 (-24.84, 9.75) | -8.43 (-11.92, -4.80) | -1.70 (-6.59, 3.44) |
| Luoyuan | Fujian | -4.92 (-6.83, -2.97) | -6.82 (-25.32, 16.26) | 1.51 (-8.44, 12.55) | -7.15 (-21.64, 10.01) |
| Mingxi | Fujian | -6.92 (-9.25, -4.54) | -4.74 (-20.64, 14.34) | -2.70 (-22.40, 22.00) | -17.1 (-30.11, -1.66) |
| Minhou | Fujian | -5.66 (-7.21, -4.09) | -3.74 (-14.43, 8.28) | -7.10 (-14.57, 1.01) | 1.94 (-7.13, 11.89) |
| Minqing | Fujian | -3.01 (-4.71, -1.27) | -5.99 (-13.42, 2.09) | 2.83 (-11.57, 19.57) | -1.66 (-16.16, 15.34) |
| Nanan | Fujian | -7.40 (-10.06, -4.66) | -13.68 (-28.76, 4.60) | 4.19 (0.57, 7.94) | -3.02 (-10.69, 5.30) |
| Nanjing | Fujian | -4.90 (-7.80, -1.91) | 4.29 (-23.68, 42.51) | -2.02 (-14.71, 12.56) | 3.21 (-3.66, 10.56) |
| Ninghua | Fujian | -4.23 (-5.71, -2.74) | -4.65 (-12.47, 3.86) | 2.72 (-11.32, 18.99) | -6.25 (-14.99, 3.38) |
| Pinghe | Fujian | -3.50 (-6.21, -0.71) | -4.74 (-13.14, 4.48) | -5.11 (-9.71, -0.29) | 11.56 (-17.49, 50.83) |
| Pingnan | Fujian | -3.25 (-6.26, -0.14) | -10.63 (-22.30, 2.80) | 3.17 (-31.81, 56.09) | 4.12 (0.08, 8.32) |
| Pingtan | Fujian | -5.93 (-7.32, -4.52) | -3.46 (-10.07, 3.63) | 0.82 (-12.52, 16.19) | -9.93 (-16.52, -2.82) |
| Pucheng | Fujian | -5.39 (-6.65, -4.11) | -3.75 (-15.54, 9.68) | -6.07 (-7.78, -4.34) | -7.42 (-21.50, 9.19) |
| Qingliu | Fujian | -9.11 (-12.89, -5.17) | -6.02 (-20.90, 11.67) | -12.72 (-19.50, -5.38) | 21.24 (4.77, 40.30) |
| Sha | Fujian | -4.49 (-6.24, -2.71) | -6.68 (-13.77, 0.98) | -2.65 (-16.33, 13.27) | 0.48 (-18.08, 23.23) |
| Shanghang | Fujian | -5.90 (-7.53, -4.25) | -12.07 (-17.09, -6.74) | 0.26 (-6.33, 7.31) | -2.28 (-5.95, 1.53) |
| Shaowu | Fujian | -2.88 (-3.80, -1.95) | -2.62 (-7.88, 2.94) | 0.92 (-5.75, 8.07) | -2.34 (-8.70, 4.46) |
| Shishi | Fujian | -9.42 (-13.06, -5.62) | -21.66 (-35.42, -4.95) | -4.29 (-23.36, 19.53) | -4.06 (-6.85, -1.19) |
| Shouning | Fujian | -6.56 (-8.93, -4.13) | -11.31 (-17.01, -5.22) | 5.23 (-7.22, 19.35) | 2.11 (-5.19, 9.98) |
| Shunchang | Fujian | -1.26 (-2.94, 0.46) | -9.05 (-14.98, -2.71) | 2.11 (-10.17, 16.08) | -0.08 (-9.12, 9.87) |
| Songxi | Fujian | -1.73 (-4.14, 0.74) | -3.42 (-33.13, 39.5) | -3.42 (-13.23, 7.50) | 2.73 (-2.25, 7.96) |
| Taining | Fujian | -3.47 (-5.35, -1.55) | -8.80 (-24.68, 10.44) | -2.29 (-12.06, 8.56) | 4.93 (2.35, 7.58) |
| Wuping | Fujian | -4.63 (-6.42, -2.81) | -9.61 (-19.50, 1.50) | -3.62 (-19.68, 15.64) | -0.33 (-4.98, 4.55) |
| Wuyishan | Fujian | -0.70 (-2.65, 1.30) | -11.92 (-24.21, 2.36) | 3.50 (-2.75, 10.14) | 0.53 (-8.32, 10.23) |
| Xianyou | Fujian | -2.64 (-6.38, 1.24) | -5.10 (-23.81, 18.21) | 1.34 (-15.65, 21.77) | 2.60 (-24.57, 39.56) |
| Xiapu | Fujian | -8.82 (-12.58, -4.89) | -19.71 (-28.83, -9.43) | -9.80 (-12.55, -6.97) | 10.72 (-5.21, 29.32) |
| Yongan | Fujian | -4.27 (-6.97, -1.50) | -11.06 (-34.16, 20.16) | -10.46 (-25.18, 7.16) | 0.52 (-12.79, 15.85) |
| Yongchun | Fujian | -1.17 (-3.40, 1.11) | -12.01 (-25.12, 3.38) | 1.34 (-4.21, 7.21) | 2.67 (-12.16, 20.00) |
| Yongding | Fujian | -5.53 (-7.27, -3.75) | -3.92 (-22.12, 18.53) | -1.66 (-12.11, 10.02) | -8.76 (-21.64, 6.24) |
| Yongtai | Fujian | -4.35 (-6.73, -1.91) | -3.29 (-26.41, 27.09) | -4.32 (-24.45, 21.16) | 1.24 (-13.10, 17.96) |
| Youxi | Fujian | -2.35 (-4.39, -0.28) | 3.24 (-16.25, 27.26) | -1.49 (-12.67, 11.12) | -8.98 (-22.29, 6.61) |
| Yunxiao | Fujian | -7.66 (-10.94, -4.26) | -14.52 (-23.08, -5.01) | -11.83 (-14.31, -9.28) | 10.63 (-17.92, 49.12) |
| Zhangping | Fujian | -4.59 (-6.66, -2.48) | -8.54 (-20.21, 4.83) | 2.53 (-10.19, 17.06) | 0.19 (-21.1, 27.22) |
| Zhangpu | Fujian | -6.09 (-8.28, -3.85) | -5.64 (-23.65, 16.62) | -9.24 (-20.50, 3.60) | 1.57 (-12.67, 18.14) |
| Zhaoan | Fujian | -6.34 (-7.68, -4.98) | -6.01 (-7.81, -4.18) | -8.87 (-17.41, 0.56) | -0.30 (-15.73, 17.94) |
| Zhenghe | Fujian | -5.31 (-7.42, -3.16) | -11.20 (-13.95, -8.35) | 9.12 (2.63, 16.02) | 2.36 (-4.74, 10.0) |
| Zherong | Fujian | -7.91 (-11.14, -4.56) | 0.41 (-15.08, 18.71) | -14.85 (-31.41, 5.72) | -0.57 (-10.35, 10.27) |
| Zhouning | Fujian | -5.12 (-7.94, -2.22) | -7.67 (-22.64, 10.19) | 7.64 (-16.49, 38.75) | -12.01 (-25.67, 4.17) |
| Aksay | Gansu | -15.16 (-21.91, -7.84) | 12.96 (-33.73, 92.56) | -12.09 (-30.09, 10.53) | -42.61 (-69.70, 8.70) |
| Cheng | Gansu | -7.77 (-12.59, -2.69) | 13.22 (-19.61, 59.45) | -3.74 (-15.47, 9.61) | -32.56 (-43.48, -19.54) |
| Chongxin | Gansu | -5.78 (-10.07, -1.28) | 13.03 (-12.84, 46.58) | -3.66 (-13.56, 7.38) | -22.02 (-39.62, 0.70) |
| Dangchang | Gansu | -5.96 (-11.10, -0.52) | 17.14 (-6.36, 46.52) | -5.51 (-18.77, 9.92) | -30.78 (-38.47, -22.13) |
| Diebu | Gansu | -7.96 (-10.39, -5.47) | -9.20 (-22.96, 7.02) | -10.33 (-24.88, 7.04) | -23.01 (-28.79, -16.77) |
| Dongxiangzu | Gansu | -11.03 (-14.93, -6.95) | 9.63 (-4.66, 26.05) | -10.13 (-18.31, -1.12) | -32.92 (-44.04, -19.60) |
| Dunhuang | Gansu | -1.03 (-4.83, 2.92) | 28.41 (15.77, 42.43) | -3.09 (-10.30, 4.70) | -12.33 (-22.88, -0.34) |
| Gangu | Gansu | -9.38 (-12.32, -6.35) | 6.60 (0.27, 13.34) | -12.50 (-28.69, 7.38) | -0.35 (-26.47, 35.05) |
| Gaolan | Gansu | -6.84 (-9.52, -4.09) | 0.45 (-14.08, 17.43) | 2.34 (-13.32, 20.82) | -18.41 (-30.79, -3.82) |
| Gaotai | Gansu | -8.45 (-13.03, -3.63) | 14.21 (-10.16, 45.17) | -8.53 (-13.5, -3.27) | -27.46 (-57.45, 23.66) |
| Guanghe | Gansu | -12.99 (-15.6, -10.30) | 3.90 (-20.71, 36.14) | -12.35 (-17.37, -7.03) | -16.70 (-33.66, 4.61) |
| Guazhou | Gansu | -10.21 (-13.53, -6.76) | -21.26 (-30.77, -10.44) | -13.06 (-22.72, -2.19) | 2.61 (-21.67, 34.42) |
| Gulang | Gansu | -10.35 (-13.62, -6.96) | 5.82 (-13.47, 29.41) | -14.49 (-25.32, -2.08) | -22.02 (-44.71, 9.97) |
| Heshui | Gansu | -9.03 (-13.94, -3.85) | 21.62 (-4.35, 54.63) | -24.97 (-41.15, -4.36) | -2.93 (-24.90, 25.47) |
| Hezheng | Gansu | -7.52 (-10.54, -4.39) | 10.01 (-17.47, 46.65) | -11.29 (-16.37, -5.90) | -12.43 (-15.61, -9.12) |
| Hezuo | Gansu | -5.03 (-8.69, -1.23) | 11.94 (2.17, 22.65) | -24.59 (-36.00, -11.14) | -0.89 (-24.39, 29.93) |
| Huachi | Gansu | -11.78 (-16.14, -7.19) | -6.96 (-33.69, 30.55) | 2.28 (-33.27, 56.79) | -11.35 (-36.30, 23.37) |
| Huan | Gansu | -6.80 (-8.91, -4.64) | -1.27 (-13.59, 12.80) | -6.40 (-15.88, 4.15) | -13.45 (-32.99, 11.78) |
| Huating | Gansu | -14.97 (-18.56, -11.22) | -0.41 (-19.55, 23.27) | -0.26 (-4.30, 3.94) | -21.38 (-34.61, -5.48) |
| Hui | Gansu | -4.18 (-7.15, -1.12) | 6.40 (-12.11, 28.81) | -6.56 (-17.77, 6.18) | -15.80 (-28.72, -0.55) |
| Huining | Gansu | -5.87 (-8.97, -2.67) | 18.39 (-1.62, 42.48) | -10.40 (-17.84, -2.29) | 2.64 (-7.34, 13.69) |
| Jingchuan | Gansu | -7.29 (-11.09, -3.32) | 10.65 (-11.62, 38.53) | -8.38 (-20.08, 5.03) | -17.15 (-43.47, 21.40) |
| Jingning | Gansu | -9.97 (-13.48, -6.31) | 2.60 (-7.51, 13.82) | -5.38 (-13.54, 3.56) | -31.42 (-48.41, -8.82) |
| Jingtai | Gansu | -4.94 (-7.85, -1.94) | 10.88 (3.17, 19.17) | -2.66 (-7.04, 1.93) | -4.18 (-15.43, 8.56) |
| Jingyuan | Gansu | -0.59 (-3.05, 1.94) | 12.10 (-4.98, 32.25) | -0.65 (-14.24, 15.09) | -7.29 (-22.02, 10.23) |
| Jinta | Gansu | -9.94 (-12.23, -7.58) | -11.57 (-29.57, 11.03) | -11.48 (-19.99, -2.07) | -12.52 (-36.31, 20.16) |
| Jishishan | Gansu | -5.51 (-9.91, -0.90) | 21.94 (-10.58, 66.29) | -7.93 (-13.73, -1.74) | -18.94 (-42.70, 14.67) |
| Kang | Gansu | -4.35 (-8.30, -0.24) | 16.08 (-3.98, 40.33) | -4.05 (-18.57, 13.06) | -16.54 (-27.56, -3.85) |
| Kangle | Gansu | -8.10 (-12.31, -3.69) | 12.70 (-23.90, 66.90) | -4.12 (-17.88, 11.96) | -27.51 (-39.14, -13.66) |
| Li | Gansu | -9.20 (-15.34, -2.61) | 23.29 (-6.90, 63.28) | -3.23 (-13.01, 7.65) | -28.03 (-46.20, -3.74) |
| Liangdang | Gansu | -4.66 (-9.10, -0.01) | 7.24 (-11.11, 29.38) | -6.30 (-40.36, 47.20) | 2.95 (-29.06, 49.40) |
| Lingtai | Gansu | -1.00 (-3.46, 1.53) | 10.08 (-13.72, 40.45) | 4.31 (-13.53, 25.84) | 0.70 (-11.51, 14.60) |
| Lintan | Gansu | -7.87 (-11.82, -3.75) | 9.22 (-10.73, 33.62) | -3.64 (-22.36, 19.61) | -21.16 (-39.32, 2.42) |
| Lintao | Gansu | -2.53 (-4.69, -0.32) | 2.76 (-11.81, 19.73) | -1.78 (-4.41, 0.93) | -13.46 (-23.01, -2.73) |
| Linxia | Gansu | -12.19 (-15.09, -9.20) | -1.42 (-29.89, 38.60) | -11.76 (-21.16, -1.25) | -24.20 (-39.26, -5.40) |
| Linxia | Gansu | -8.46 (-12.16, -4.62) | 16.91 (-18.43, 67.57) | -5.26 (-21.56, 14.43) | -13.65 (-23.97, -1.94) |
| Linze | Gansu | -13.72 (-17.37, -9.90) | 8.93 (-10.82, 33.06) | -9.70 (-12.67, -6.63) | -21.40 (-46.51, 15.47) |
| Longxi | Gansu | -3.45 (-5.42, -1.44) | 0.32 (-9.80, 11.58) | -9.85 (-23.01, 5.55) | 4.85 (-8.24, 19.81) |
| Luqu | Gansu | -6.99 (-8.76, -5.18) | -0.67 (-7.14, 6.25) | -10.52 (-28.53, 12.03) | -4.13 (-9.85, 1.95) |
| Maqu | Gansu | 0.06 (-5.16, 5.57) | 8.24 (-9.89, 30.02) | -21.17 (-33.62, -6.38) | 10.21 (-33.53, 82.72) |
| Min | Gansu | -5.23 (-8.78, -1.54) | 10.81 (-10.15, 36.66) | -3.16 (-16.96, 12.94) | -21.09 (-35.30, -3.75) |
| Minqin | Gansu | -7.40 (-9.79, -4.94) | -7.48 (-23.95, 12.57) | -2.29 (-18.20, 16.70) | -15.70 (-33.84, 7.42) |
| Minyue | Gansu | -10.07 (-12.59, -7.48) | -1.73 (-28.25, 34.58) | -1.11 (-10.98, 9.86) | -11.14 (-23.83, 3.68) |
| Qinan | Gansu | -10.59 (-14.26, -6.76) | 3.86 (-18.45, 32.28) | -12.31 (-32.89, 14.58) | -17.18 (-45.18, 25.14) |
| Qingcheng | Gansu | -4.48 (-6.56, -2.35) | 3.58 (-5.70, 13.78) | 3.37 (-7.69, 15.76) | -10.18 (-17.19, -2.59) |
| Qingshui | Gansu | -4.20 (-7.18, -1.12) | 15.34 (5.27, 26.37) | -8.02 (-17.96, 3.12) | -18.51 (-31.65, -2.85) |
| Shandan | Gansu | -9.23 (-12.73, -5.6) | 14.37 (-7.87, 41.97) | -11.11 (-22.93, 2.52) | -13.31 (-25.63, 1.04) |
| Subei | Gansu | -13.07 (-16.62, -9.37) | -0.73 (-19.11, 21.83) | -17.23 (-27.26, -5.82) | -22.89 (-54.65, 31.11) |
| Sunan | Gansu | -2.34 (-6.98, 2.54) | 22.47 (-2.71, 54.15) | -13.49 (-25.43, 0.37) | -16.72 (-48.88, 35.67) |
| Tianzhu | Gansu | -6.36 (-11.69, -0.71) | 3.84 (-8.49, 17.82) | 5.22 (-11.64, 25.31) | -30.42 (-45.51, -11.14) |
| Tongwei | Gansu | -3.21 (-7.09, 0.83) | 2.63 (-22.91, 36.63) | -2.33 (-25.56, 28.16) | -4.30 (-16.36, 9.49) |
| Weiyuan | Gansu | -5.26 (-7.76, -2.69) | 2.26 (-5.17, 10.27) | -15.44 (-25.53, -3.99) | -9.96 (-30.91, 17.35) |
| Wen | Gansu | -2.74 (-5.74, 0.37) | 12.92 (-7.64, 38.06) | -11.69 (-20.36, -2.08) | -9.65 (-26.66, 11.29) |
| Wushan | Gansu | -10.55 (-13.88, -7.09) | 12.11 (-17.82, 52.94) | -15.74 (-23.77, -6.85) | -16.47 (-31.37, 1.67) |
| Xiahe | Gansu | -10.65 (-13.75, -7.44) | 0.34 (-13.57, 16.48) | -3.19 (-11.70, 6.15) | -26.99 (-45.62, -1.97) |
| Xihe | Gansu | -8.13 (-13.22, -2.74) | 9.11 (-2.69, 22.34) | -3.20 (-8.01, 1.87) | -22.59 (-37.19, -4.61) |
| Yongchang | Gansu | -12.54 (-17.01, -7.84) | 20.34 (-14.23, 68.86) | -12.26 (-21.99, -1.33) | -21.97 (-50.06, 21.90) |
| Yongdeng | Gansu | -4.76 (-7.24, -2.22) | 3.84 (-19.36, 33.71) | -4.21 (-13.15, 5.66) | 7.83 (-3.64, 20.67) |
| Yongjing | Gansu | -3.01 (-5.70, -0.25) | 1.39 (-10.61, 15.00) | -4.43 (-15.59, 8.20) | -13.55 (-38.00, 20.55) |
| Yumen | Gansu | -7.29 (-9.33, -5.20) | -4.52 (-17.37, 10.32) | -3.64 (-18.02, 13.26) | -3.09 (-23.95, 23.50) |
| Yuzhong | Gansu | -10.71 (-13.80, -7.50) | 5.73 (-6.67, 19.78) | -17.66 (-28.91, -4.64) | 1.75 (-23.63, 35.57) |
| Zhang | Gansu | -0.17 (-3.61, 3.39) | 7.93 (-18.95, 43.72) | -2.29 (-15.89, 13.51) | -9.93 (-24.01, 6.76) |
| Zhangjiachuan | Gansu | -9.34 (-12.17, -6.43) | 6.36 (-12.28, 28.97) | -6.00 (-21.27, 12.23) | -12.22 (-35.73, 19.90) |
| Zhengning | Gansu | -5.49 (-9.49, -1.30) | 12.48 (-16.99, 52.42) | -4.46 (-9.59, 0.95) | -16.61 (-33.62, 4.75) |
| Zhenyuan | Gansu | -5.76 (-8.70, -2.72) | 2.19 (-21.71, 33.39) | 2.48 (-10.67, 17.56) | -19.64 (-32.97, -3.65) |
| Zhouqu | Gansu | -5.52 (-8.23, -2.73) | 3.37 (-4.62, 12.04) | -5.38 (-15.01, 5.35) | -18.73 (-37.97, 6.48) |
| Zhuanglang | Gansu | -3.32 (-5.74, -0.83) | -0.49 (-6.55, 5.95) | 1.77 (-5.06, 9.08) | -17.45 (-33.15, 1.95) |
| Zhuoni | Gansu | -7.30 (-10.64, -3.83) | 3.80 (-16.14, 28.49) | -7.24 (-11.06, -3.26) | -19.05 (-40.91, 10.89) |
| Boluo | Guangdong | -5.88 (-8.36, -3.32) | 11.76 (1.54, 23.01) | -4.49 (-13.31, 5.22) | -13.78 (-20.01, -7.07) |
| Chaoan | Guangdong | -2.61 (-5.54, 0.41) | 20.37 (0.25, 44.52) | -7.36 (-15.56, 1.64) | 0.66 (-7.46, 9.49) |
| Conghua | Guangdong | -7.86 (-9.50, -6.19) | 1.87 (-10.99, 16.60) | -13.35 (-19.15, -7.13) | -3.59 (-10.74, 4.12) |
| Dapu | Guangdong | -8.75 (-10.66, -6.79) | -1.42 (-3.04, 0.23) | -3.72 (-7.88, 0.62) | -12.24 (-24.27, 1.70) |
| Deqing | Guangdong | -6.22 (-8.45, -3.93) | -5.85 (-20.9, 12.06) | -8.57 (-18.92, 3.09) | -17.51 (-29.51, -3.46) |
| Dongyuan | Guangdong | -5.32 (-8.07, -2.49) | -2.97 (-14.29, 9.83) | -17.03 (-31.91, 1.09) | -3.14 (-17.51, 13.72) |
| Enping | Guangdong | 1.17 (-0.44, 2.81) | 4.76 (-12.34, 25.21) | -5.83 (-10.74, -0.66) | -2.10 (-10.31, 6.85) |
| Fengkai | Guangdong | -1.70 (-3.45, 0.09) | -5.77 (-13.85, 3.05) | 3.68 (-5.10, 13.28) | -11.17 (-17.83, -3.97) |
| Fengshun | Guangdong | -7.37 (-9.46, -5.23) | -5.66 (-20.73, 12.26) | 5.00 (-1.28, 11.69) | -7.17 (-14.20, 0.44) |
| Fogang | Guangdong | -5.92 (-7.69, -4.12) | 0.73 (-10.62, 13.51) | -14.43 (-18.64, -10.00) | 0.36 (-11.58, 13.91) |
| Gaoyao | Guangdong | -6.63 (-8.01, -5.23) | -2.20 (-18.03, 16.68) | -5.54 (-16.12, 6.37) | -6.56 (-8.37, -4.71) |
| Gaozhou | Guangdong | -6.11 (-7.81, -4.39) | -3.88 (-15.38, 9.18) | -3.41 (-9.79, 3.43) | -14.71 (-17.71, -11.61) |
| Guangning | Guangdong | -1.49 (-4.26, 1.37) | 10.55 (0.31, 21.84) | 6.32 (-11.88, 28.28) | -16.91 (-22.12, -11.36) |
| Haifeng | Guangdong | -5.29 (-8.00, -2.51) | 1.84 (-10.60, 16.01) | -18.95 (-23.47, -14.17) | 6.98 (-9.90, 27.02) |
| Heping | Guangdong | -3.78 (-5.77, -1.75) | -5.57 (-12.9, 2.39) | -9.57 (-19.98, 2.18) | -8.03 (-28.33, 18.02) |
| Heshan | Guangdong | -5.83 (-7.56, -4.05) | -1.87 (-22.65, 24.49) | -3.89 (-11.24, 4.06) | -6.25 (-14.17, 2.41) |
| Huaiji | Guangdong | -6.85 (-8.62, -5.05) | -10.76 (-18.82, -1.89) | 1.44 (-7.66, 11.43) | -14.64 (-24.53, -3.46) |
| Huazhou | Guangdong | -1.55 (-3.68, 0.63) | 2.47 (-6.77, 12.63) | -1.06 (-13.34, 12.95) | -4.82 (-29.23, 28.01) |
| Huidong | Guangdong | -5.32 (-6.76, -3.85) | -3.56 (-13.41, 7.42) | -9.25 (-12.81, -5.55) | -11.82 (-18.71, -4.34) |
| Huilai | Guangdong | -3.10 (-4.61, -1.56) | 2.04 (-10.30, 16.08) | -8.63 (-15.56, -1.13) | -4.92 (-16.96, 8.87) |
| Jiaoling | Guangdong | -9.76 (-12.73, -6.69) | -0.71 (-11.00, 10.77) | 6.83 (-7.82, 23.82) | -16.93 (-22.84, -10.55) |
| Jiedong | Guangdong | -5.52 (-7.27, -3.73) | 2.98 (-8.37, 15.74) | -10.95 (-19.85, -1.05) | -8.49 (-17.25, 1.19) |
| Jiexi | Guangdong | -7.16 (-9.00, -5.29) | 5.96 (-1.43, 13.89) | -4.65 (-8.38, -0.78) | -10.29 (-23.35, 5.00) |
| Kaiping | Guangdong | 0.89 (-0.95, 2.77) | 14.31 (3.39, 26.39) | -1.51 (-8.97, 6.56) | 0.50 (-10.21, 12.50) |
| Lechang | Guangdong | -2.27 (-3.79, -0.71) | 3.67 (-7.37, 16.03) | 2.76 (-7.50, 14.16) | 1.05 (-10.05, 13.53) |
| Leizhou | Guangdong | -0.04 (-1.22, 1.15) | -3.64 (-10.25, 3.45) | 1.96 (-9.44, 14.78) | -4.58 (-8.81, -0.16) |
| Lianjiang | Guangdong | 0.14 (-1.21, 1.50) | 3.65 (-7.26, 15.85) | -3.50 (-13.68, 7.89) | 0.96 (-7.28, 9.93) |
| Liannan | Guangdong | -1.93 (-4.91, 1.14) | 9.97 (0.18, 20.72) | 5.21 (-14.52, 29.49) | -17.71 (-29.20, -4.37) |
| Lianping | Guangdong | -10.65 (-13.20, -8.03) | 3.23 (-3.86, 10.85) | -8.11 (-14.70, -1.01) | -6.99 (-23.73, 13.42) |
| Lianshan | Guangdong | 0.23 (-2.08, 2.60) | -1.87 (-20.85, 21.67) | 0.12 (-20.47, 26.04) | -4.99 (-14.18, 5.19) |
| Lianzhou | Guangdong | -1.83 (-3.92, 0.30) | 6.98 (-0.30, 14.78) | 12.85 (5.16, 21.10) | -8.36 (-11.42, -5.19) |
| Longchuan | Guangdong | -4.79 (-7.46, -2.04) | 5.69 (3.04, 8.41) | -1.67 (-12.98, 11.10) | -7.03 (-27.31, 18.92) |
| Longmen | Guangdong | -4.70 (-6.66, -2.70) | 2.09 (-4.96, 9.67) | -7.11 (-10.60, -3.48) | -9.61 (-13.01, -6.07) |
| Lufeng | Guangdong | 0.29 (-1.39, 2.00) | 7.28 (-3.22, 18.91) | -3.01 (-7.37, 1.55) | -4.70 (-18.31, 11.17) |
| Luhe | Guangdong | -2.55 (-4.91, -0.13) | 2.17 (-4.70, 9.53) | -7.93 (-33.47, 27.41) | 6.01 (-0.92, 13.43) |
| Luoding | Guangdong | -1.84 (-3.87, 0.23) | 3.33 (-4.91, 12.28) | 3.57 (-7.23, 15.63) | -11.79 (-24.45, 2.98) |
| Mei | Guangdong | -8.06 (-9.46, -6.63) | -9.22 (-17.11, -0.57) | -8.83 (-17.99, 1.37) | -4.41 (-11.79, 3.60) |
| Nanao | Guangdong | -2.14 (-3.70, -0.55) | -1.42 (-11.69, 10.04) | 0.78 (-10.99, 14.10) | -5.43 (-21.08, 13.32) |
| Nanxiong | Guangdong | 0 (-2.59, 2.65) | -5.28 (-13.90, 4.20) | 9.64 (-12.12, 36.79) | -9.40 (-19.38, 1.80) |
| Pingyuan | Guangdong | -6.84 (-9.14, -4.49) | -3.45 (-4.57, -2.32) | 4.39 (1.14, 7.75) | -9.61 (-14.82, -4.07) |
| Puning | Guangdong | -3.27 (-4.38, -2.15) | 1.40 (-2.73, 5.70) | -6.26 (-9.84, -2.55) | -8.80 (-16.43, -0.47) |
| Qingxin | Guangdong | -7.46 (-10.88, -3.92) | -1.66 (-4.76, 1.55) | -3.90 (-21.31, 17.35) | -7.00 (-12.51, -1.14) |
| Raoping | Guangdong | 1.75 (-0.24, 3.78) | 6.55 (-1.00, 14.67) | -6.78 (-20.05, 8.69) | 3.83 (-14.44, 26.00) |
| Renhua | Guangdong | -8.78 (-12.41, -4.99) | -17.09 (-40.39, 15.31) | -2.08 (-8.54, 4.84) | -4.14 (-9.28, 1.30) |
| Ruyuan | Guangdong | -4.29 (-6.29, -2.24) | -13.07 (-23.27, -1.50) | 1.17 (-11.33, 15.44) | 0.61 (-11.54, 14.44) |
| Shixing | Guangdong | -3.29 (-4.91, -1.63) | -7.05 (-22.96, 12.14) | -0.05 (-11.79, 13.26) | -4.71 (-15.49, 7.45) |
| Sihui | Guangdong | -6.98 (-8.92, -4.99) | -6.17 (-18.64, 8.20) | -2.30 (-18.47, 17.07) | -9.93 (-18.44, -0.52) |
| Suixi | Guangdong | 0.48 (-1.12, 2.11) | 1.16 (-9.73, 13.36) | 0.02 (-8.67, 9.54) | -8.24 (-13.12, -3.08) |
| Taishan | Guangdong | 1.14 (-1.25, 3.59) | 10.42 (-5.18, 28.59) | -11.2 (-18.04, -3.80) | 5.32 (-7.53, 19.96) |
| Wengyuan | Guangdong | -4.01 (-6.37, -1.59) | -15.84 (-20.73, -10.64) | 5.59 (4.06, 7.14) | -11.09 (-15.39, -6.56) |
| Wuchuan | Guangdong | 0.35 (-1.25, 1.97) | 4.31 (0.20, 8.59) | 7.44 (-3.32, 19.38) | -3.55 (-15.32, 9.86) |
| Wuhua | Guangdong | -6.96 (-8.89, -4.99) | 4.94 (-0.85, 11.08) | -8.24 (-19.33, 4.38) | -12.82 (-23.01, -1.28) |
| Xinfeng | Guangdong | -3.89 (-5.93, -1.81) | 3.41 (-9.36, 17.98) | -7.91 (-16.14, 1.14) | -6.39 (-17.68, 6.45) |
| Xingning | Guangdong | -9.07 (-10.01, -8.11) | -6.04 (-16.71, 6.00) | -6.70 (-11.92, -1.17) | -8.67 (-14.48, -2.46) |
| Xinxing | Guangdong | -1.51 (-3.27, 0.28) | 5.19 (-11.83, 25.50) | -3.75 (-10.20, 3.16) | -7.92 (-19.16, 4.89) |
| Xinyi | Guangdong | -4.48 (-5.94, -3.00) | -0.48 (-5.42, 4.72) | -3.17 (-9.23, 3.29) | 4.49 (-7.07, 17.50) |
| Xuwen | Guangdong | -1.71 (-3.62, 0.25) | 6.73 (-10.77, 27.66) | -3.08 (-11.28, 5.88) | -7.83 (-18.88, 4.72) |
| Yangchun | Guangdong | -6.50 (-8.20, -4.76) | -1.13 (-14.51, 14.35) | -12.79 (-22.14, -2.33) | -12.25 (-19.54, -4.31) |
| Yangdong | Guangdong | -3.67 (-6.03, -1.25) | 9.20 (-0.79, 20.21) | -10.65 (-19.06, -1.37) | -15.32 (-24.65, -4.83) |
| Yangshan | Guangdong | -2.57 (-4.26, -0.84) | 2.16 (-3.97, 8.69) | 0.65 (-12.65, 15.97) | -10.46 (-21.77, 2.49) |
| Yangxi | Guangdong | -6.01 (-7.73, -4.26) | 2.81 (-5.57, 11.93) | -1.94 (-13.96, 11.76) | -12.54 (-21.32, -2.78) |
| Yingde | Guangdong | -3.02 (-4.85, -1.16) | 2.25 (-14.97, 22.96) | -2.77 (-9.51, 4.47) | -11.38 (-18.45, -3.70) |
| Yunan | Guangdong | -6.02 (-7.78, -4.21) | 1.40 (-15.91, 22.27) | -9.88 (-18.22, -0.70) | -7.37 (-17.38, 3.86) |
| Yunan | Guangdong | -1.23 (-2.66, 0.22) | 3.34 (-3.43, 10.59) | 1.55 (-3.49, 6.86) | -7.16 (-14.20, 0.46) |
| Zengcheng | Guangdong | -6.29 (-8.62, -3.91) | 13.88 (8.05, 20.03) | -9.33 (-17.50, -0.36) | -6.90 (-16.96, 4.38) |
| Zijin | Guangdong | -7.27 (-8.94, -5.58) | -2.55 (-11.34, 7.12) | -2.55 (-11.67, 7.50) | -17.25 (-22.48, -11.66) |
| Bama | Guangxi | -3.17 (-4.75, -1.57) | 1.47 (-11.54, 16.39) | 2.02 (-6.58, 11.41) | -5.09 (-14.95, 5.90) |
| Beiliu | Guangxi | -6.91 (-8.49, -5.30) | -9.33 (-24.70, 9.17) | 2.46 (-1.62, 6.72) | -11.16 (-12.39, -9.90) |
| Binyang | Guangxi | -2.43 (-4.41, -0.42) | -12.55 (-21.00, -3.21) | 1.80 (-10.83, 16.21) | -9.06 (-19.39, 2.61) |
| Bobai | Guangxi | -4.93 (-7.34, -2.45) | -7.68 (-24.27, 12.55) | 3.63 (-9.33, 18.45) | 4.42 (-8.57, 19.24) |
| Cangwu | Guangxi | -1.10 (-3.56, 1.42) | -13.54 (-17.67, -9.20) | 4.55 (-6.13, 16.45) | 4.25 (-6.04, 15.66) |
| Cenxi | Guangxi | -3.73 (-5.92, -1.48) | -17.41 (-30.68, -1.60) | -3.68 (-13.24, 6.92) | -5.81 (-10.67, -0.69) |
| Dahua | Guangxi | 1.20 (-1.30, 3.76) | -4.09 (-13.57, 6.44) | 7.16 (-5.60, 21.64) | -3.77 (-20.73, 16.81) |
| Daxin | Guangxi | -5.50 (-8.14, -2.78) | -5.37 (-24.87, 19.19) | -7.57 (-22.20, 9.82) | -8.21 (-27.70, 16.53) |
| Debao | Guangxi | -5.88 (-7.84, -3.87) | -0.90 (-21.52, 25.13) | -5.81 (-14.1, 3.27) | -3.49 (-18.44, 14.20) |
| Donglan | Guangxi | 0.85 (-0.99, 2.73) | -0.34 (-14.18, 15.73) | 6.32 (-9.08, 24.33) | -6.77 (-14.81, 2.03) |
| Dongxing | Guangxi | -10.00 (-12.4, -7.53) | -5.91 (-13.79, 2.68) | -18.14 (-31.44, -2.25) | -3.18 (-24.41, 24.02) |
| Douan | Guangxi | -1.44 (-3.72, 0.89) | 2.36 (-9.89, 16.27) | 8.94 (-6.12, 26.42) | -11.36 (-21.59, 0.21) |
| Fengshan | Guangxi | -0.13 (-2.87, 2.68) | 1.90 (-24.71, 37.92) | 0.35 (-2.49, 3.28) | -14.78 (-24.05, -4.39) |
| Fuchuan | Guangxi | -3.22 (-4.47, -1.94) | -3.33 (-10.74, 4.69) | -1.30 (-6.06, 3.70) | 0.70 (-14.95, 19.23) |
| Fusui | Guangxi | -6.93 (-9.53, -4.25) | -12.94 (-26.40, 2.97) | -7.86 (-13.34, -2.04) | 10.35 (-13.68, 41.07) |
| Gongcheng | Guangxi | -4.53 (-7.51, -1.44) | -21.51 (-28.45, -13.9) | -0.90 (-25.76, 32.27) | -9.92 (-22.23, 4.34) |
| Guanyang | Guangxi | -6.68 (-9.47, -3.80) | -20.15 (-29.60, -9.42) | -15.73 (-28.88, -0.16) | 3.54 (-4.05, 11.73) |
| Guiping | Guangxi | -0.72 (-1.98, 0.55) | -1.05 (-6.94, 5.21) | 1.35 (-7.80, 11.41) | 0.41 (-8.36, 10.02) |
| Heng | Guangxi | -8.43 (-10.91, -5.87) | -8.35 (-25.08, 12.10) | -2.95 (-16.52, 12.82) | -9.91 (-35.62, 26.06) |
| Hepu | Guangxi | -4.55 (-6.63, -2.43) | -4.72 (-24.44, 20.14) | 6.86 (-4.52, 19.58) | -5.63 (-14.93, 4.70) |
| Heshan | Guangxi | -1.85 (-4.49, 0.87) | -0.54 (-16.96, 19.13) | 10.02 (0.65, 20.27) | -4.64 (-19.29, 12.66) |
| Huanjiang | Guangxi | -0.91 (-2.75, 0.97) | -7.34 (-11.85, -2.60) | 10.83 (-2.41, 25.85) | -4.87 (-13.91, 5.13) |
| Jingxi | Guangxi | -3.20 (-4.84, -1.53) | 2.54 (-8.15, 14.47) | -1.24 (-20.03, 21.98) | -1.60 (-5.56, 2.51) |
| Jinxiu | Guangxi | -3.69 (-6.09, -1.22) | 2.29 (-19.01, 29.19) | 2.15 (-17.21, 26.06) | -1.83 (-8.86, 5.73) |
| Leye | Guangxi | -1.66 (-4.67, 1.46) | -6.52 (-29.74, 24.36) | 4.74 (-20.41, 37.82) | -5.36 (-26.79, 22.34) |
| Lingchuan | Guangxi | -4.91 (-6.94, -2.84) | -8.79 (-19.83, 3.76) | -1.54 (-20.00, 21.17) | -0.52 (-9.81, 9.73) |
| Lingshan | Guangxi | 1.43 (-0.83, 3.74) | -9.73 (-13.01, -6.33) | 14.45 (5.28, 24.41) | -6.67 (-13.47, 0.66) |
| Lingui | Guangxi | -3.32 (-4.67, -1.95) | -7.23 (-18.81, 6.02) | 1.13 (-7.17, 10.18) | -0.69 (-7.06, 6.12) |
| Lingyun | Guangxi | -0.78 (-3.88, 2.42) | 3.22 (-6.13, 13.51) | 6.22 (-10.44, 25.97) | -19.64 (-35.17, -0.40) |
| Lipu | Guangxi | -4.34 (-5.91, -2.74) | -7.44 (-16.10, 2.12) | 2.39 (-9.13, 15.37) | -11.27 (-18.25, -3.69) |
| Liucheng | Guangxi | -0.92 (-2.95, 1.16) | -11.40 (-27.65, 8.49) | 2.46 (-4.10, 9.46) | -6.16 (-13.06, 1.30) |
| Liujiang | Guangxi | -4.42 (-9.60, 1.05) | -6.42 (-17.12, 5.67) | 38.48 (16.40, 64.74) | -14.76 (-47.09, 37.32) |
| Longan | Guangxi | -6.32 (-8.26, -4.34) | -14.75 (-27.35, 0.02) | 0.21 (-6.02, 6.86) | 0.82 (-12.59, 16.28) |
| Longlin | Guangxi | 0.76 (-1.88, 3.48) | -3.58 (-12.92, 6.77) | 10.27 (-13.12, 39.96) | 2.89 (-19.58, 31.64) |
| Longsheng | Guangxi | -3.81 (-7.11, -0.39) | -16.39 (-32.22, 3.13) | 16.13 (4.51, 29.03) | 5.06 (-12.25, 25.77) |
| Long州 | Guangxi | -1.51 (-3.89, 0.93) | -7.71 (-24.32, 12.55) | 0.08 (-22.39, 29.05) | 2.36 (-12.74, 20.09) |
| Luchuan | Guangxi | -5.20 (-6.99, -3.37) | -5.15 (-24.25, 18.76) | -4.76 (-8.42, -0.96) | 3.13 (-5.73, 12.81) |
| Luocheng | Guangxi | -3.22 (-5.92, -0.43) | 1.04 (-28.11, 42.00) | 1.88 (-20.49, 30.56) | -7.42 (-12.41, -2.14) |
| Luzhai | Guangxi | -2.86 (-5.19, -0.48) | -14.04 (-24.27, -2.43) | -3.55 (-20.48, 17.00) | 5.39 (-9.86, 23.23) |
| Mashan | Guangxi | -5.34 (-7.67, -2.96) | -8.81 (-25.64, 11.84) | 8.04 (2.54, 13.83) | -14.57 (-23.96, -4.03) |
| Mengshan | Guangxi | -3.25 (-5.89, -0.54) | 2.89 (-13.21, 21.98) | -9.07 (-22.39, 6.53) | 10.97 (-1.21, 24.65) |
| Nandan | Guangxi | -1.18 (-4.20, 1.94) | 0.50 (-9.86, 12.04) | -6.72 (-29.29, 23.07) | 17.84 (0.72, 37.87) |
| Napo | Guangxi | -3.07 (-5.50, -0.57) | -6.59 (-24.88, 16.15) | 8.00 (-8.80, 27.89) | 1.70 (-14.55, 21.05) |
| Ningming | Guangxi | -3.91 (-6.03, -1.74) | -2.75 (-21.01, 19.74) | -0.47 (-4.30, 3.52) | -14.33 (-27.49, 1.23) |
| Pingle | Guangxi | -2.67 (-4.28, -1.04) | 0.02 (-5.12, 5.43) | -7.76 (-10.08, -5.38) | 5.19 (-4.79, 16.23) |
| Pingnan | Guangxi | -0.91 (-1.80, -0.02) | -1.20 (-9.61, 7.98) | -4.83 (-11.83, 2.73) | -1.74 (-5.00, 1.64) |
| Pingxiang | Guangxi | -4.38 (-6.17, -2.55) | 2.44 (-15.17, 23.71) | -6.49 (-12.98, 0.48) | -8.50 (-22.20, 7.62) |
| Pingguo | Guangxi | -6.22 (-7.74, -4.67) | -13.77 (-24.44, -1.59) | -7.43 (-12.81, -1.71) | 1.65 (-1.79, 5.21) |
| Pubei | Guangxi | -5.40 (-7.08, -3.68) | -2.89 (-17.94, 14.92) | 1.21 (-7.29, 10.49) | -1.74 (-12.86, 10.80) |
| Quanzhou | Guangxi | -4.53 (-6.74, -2.26) | -12.33 (-28.14, 6.95) | -2.25 (-10.63, 6.91) | -6.30 (-14.42, 2.58) |
| Rong | Guangxi | -6.40 (-7.39, -5.41) | -5.15 (-15.88, 6.95) | -4.18 (-11.24, 3.45) | -6.14 (-12.63, 0.83) |
| Rongan | Guangxi | -7.60 (-9.22, -5.96) | -6.07 (-21.23, 12.02) | -3.78 (-10.13, 3.02) | -11.40 (-20.18, -1.66) |
| Rongshui | Guangxi | -3.98 (-7.44, -0.40) | -12.54 (-28.09, 6.37) | 17.69 (-8.61, 51.57) | -9.19 (-11.13, -7.20) |
| Sanjiang | Guangxi | -4.23 (-6.56, -1.84) | -7.86 (-22.63, 9.73) | 7.10 (-2.91, 18.14) | -12.07 (-29.29, 9.35) |
| Shanglin | Guangxi | -1.51 (-4.86, 1.97) | -20.10 (-29.52, -9.43) | 3.15 (-10.32, 18.63) | 10.12 (5.59, 14.84) |
| Shangsi | Guangxi | -1.97 (-3.93, 0.04) | -0.69 (-23.16, 28.35) | -2.48 (-16.21, 13.51) | 1.60 (-9.61, 14.18) |
| Teng | Guangxi | -4.13 (-6.09, -2.12) | -10.98 (-15.40, -6.33) | -6.05 (-14.89, 3.71) | 5.08 (-14.93, 29.80) |
| Tiandeng | Guangxi | -5.74 (-7.86, -3.57) | -12.44 (-31.45, 11.83) | -1.62 (-7.64, 4.79) | -2.32 (-7.60, 3.27) |
| Tiandong | Guangxi | -5.69 (-7.45, -3.91) | -1.09 (-5.82, 3.88) | -3.17 (-19.39, 16.32) | -14.51 (-23.64, -4.29) |
| Tiane | Guangxi | 4.17 (1.46, 6.96) | -5.60 (-26.40, 21.09) | -3.64 (-14.7, 8.87) | -0.07 (-16.31, 19.33) |
| Tianlin | Guangxi | 1.95 (-0.22, 4.17) | 2.69 (-5.53, 11.63) | 12.82 (2.05, 24.73) | -7.46 (-16.85, 2.99) |
| Tianyang | Guangxi | -3.61 (-6.56, -0.57) | 7.83 (-7.03, 25.06) | -12.48 (-23.04, -0.46) | -15.40 (-36.27, 12.29) |
| Wuming | Guangxi | -0.69 (-2.49, 1.15) | -9.33 (-17.51, -0.33) | 5.97 (-2.03, 14.63) | 2.25 (-6.49, 11.80) |
| Wuxuan | Guangxi | -5.79 (-8.35, -3.16) | -10.91 (-28.96, 11.72) | 5.22 (-17.35, 33.95) | -1.54 (-16.92, 16.68) |
| Xiangzhou | Guangxi | -3.25 (-5.16, -1.30) | -10.99 (-14.54, -7.29) | 1.17 (-2.36, 4.83) | 7.69 (-6.08, 23.49) |
| Xilin | Guangxi | 1.45 (-1.56, 4.56) | -0.54 (-23.42, 29.18) | -5.55 (-10.19, -0.67) | -8.18 (-28.05, 17.17) |
| Xincheng | Guangxi | -2.22 (-4.93, 0.56) | -0.39 (-10.38, 10.72) | 5.85 (-15.53, 32.64) | 9.04 (-3.26, 22.90) |
| Xingan | Guangxi | -2.74 (-4.26, -1.20) | -6.32 (-17.13, 5.90) | 5.35 (-3.19, 14.63) | -2.17 (-6.53, 2.38) |
| Xingye | Guangxi | -7.00 (-9.22, -4.72) | -3.28 (-14.60, 9.54) | -18.05 (-28.37, -6.24) | 2.80 (-10.24, 17.74) |
| Yangshuo | Guangxi | -5.77 (-8.29, -3.19) | -12.84 (-33.74, 14.66) | -0.13 (-14.53, 16.69) | -5.77 (-16.40, 6.20) |
| Yizhou | Guangxi | -1.42 (-3.13, 0.32) | -2.27 (-10.46, 6.66) | -1.59 (-13.67, 12.19) | -12.77 (-16.65, -8.72) |
| Yongfu | Guangxi | -2.91 (-5.18, -0.58) | -8.68 (-33.36, 25.13) | -2.34 (-8.84, 4.63) | -9.52 (-14.09, -4.70) |
| Zhaoping | Guangxi | -8.12 (-9.60, -6.61) | -15.57 (-20.94, -9.84) | 1.92 (-3.24, 7.36) | -7.41 (-11.43, -3.21) |
| Zhongshan | Guangxi | -2.95 (-4.87, -0.99) | -12.14 (-20.91, -2.40) | 4.16 (-11.60, 22.74) | -6.30 (-12.69, 0.56) |
| Ziyuan | Guangxi | -1.25 (-3.38, 0.93) | -11.14 (-11.59, -10.68) | 9.77 (-0.95, 21.65) | 1.27 (-11.95, 16.48) |
| Anlong | Guizhou | -0.60 (-2.28, 1.10) | -1.72 (-13.24, 11.33) | 3.95 (-5.85, 14.76) | -3.50 (-11.19, 4.87) |
| Bozhou | Guizhou | -5.28 (-7.41, -3.10) | 4.43 (-1.99, 11.28) | -1.87 (-9.76, 6.71) | -14.38 (-23.43, -4.25) |
| Ceheng | Guizhou | 3.48 (2.21, 4.77) | 0.86 (-7.78, 10.30) | 4.02 (-8.50, 18.25) | 0.17 (-9.17, 10.48) |
| Cen巩 | Guizhou | -6.26 (-7.99, -4.50) | -13.00 (-22.32, -2.55) | -1.33 (-12.67, 11.48) | -9.06 (-20.27, 3.74) |
| Changshun | Guizhou | -0.56 (-3.25, 2.20) | 3.25 (-4.06, 11.11) | 0.09 (-6.46, 7.09) | -3.68 (-13.36, 7.10) |
| Chishui | Guizhou | -6.05 (-8.21, -3.84) | -16.05 (-26.55, -4.05) | -9.66 (-17.50, -1.08) | 2.20 (-4.24, 9.08) |
| Congjiang | Guizhou | -0.22 (-1.61, 1.19) | -2.58 (-19.56, 17.97) | -0.08 (-5.36, 5.49) | -0.81 (-9.11, 8.25) |
| Dafang | Guizhou | 1.13 (-0.70, 3.00) | 11.56 (8.37, 14.85) | -6.66 (-21.07, 10.38) | 0.32 (-6.38, 7.49) |
| Danzhai | Guizhou | -2.20 (-4.28, -0.08) | 5.44 (1.97, 9.03) | 2.87 (-16.24, 26.34) | -6.97 (-21.01, 9.57) |
| Daozhen | Guizhou | -1.58 (-3.38, 0.26) | 10.46 (-4.23, 27.40) | 0.33 (-6.65, 7.83) | -5.76 (-11.64, 0.50) |
| Dejiang | Guizhou | -4.11 (-6.94, -1.18) | 14.18 (10.65, 17.82) | -0.39 (-8.15, 8.03) | -19.74 (-23.94, -15.30) |
| Douyun | Guizhou | -0.59 (-2.45, 1.31) | -5.81 (-20.66, 11.82) | 7.91 (0.49, 15.89) | -8.33 (-13.87, -2.42) |
| Dushan | Guizhou | -3.39 (-6.00, -0.72) | -3.14 (-12.78, 7.58) | 3.54 (-13.43, 23.84) | -15.59 (-34.11, 8.14) |
| Fenggang | Guizhou | -2.82 (-5.62, 0.07) | 6.81 (-13.29, 31.57) | -13.26 (-19.22, -6.86) | 0.16 (-16.40, 19.99) |
| Fuquan | Guizhou | -3.87 (-5.32, -2.39) | -7.48 (-12.23, -2.46) | 0.26 (-4.19, 4.92) | -7.46 (-15.53, 1.37) |
| Guanling | Guizhou | -2.40 (-4.61, -0.14) | 12.75 (-2.81, 30.80) | 0.05 (-8.66, 9.59) | -6.99 (-19.37, 7.30) |
| Guiding | Guizhou | 0.86 (-1.46, 3.23) | -4.60 (-24.98, 21.31) | 9.14 (3.72, 14.83) | -9.80 (-15.68, -3.51) |
| Hezhang | Guizhou | 6.37 (4.07, 8.72) | 8.78 (-16.06, 40.98) | 11.74 (2.89, 21.36) | -4.20 (-8.24, 0.02) |
| Huangping | Guizhou | -3.60 (-6.38, -0.74) | -0.70 (-19.63, 22.69) | 4.00 (-14.69, 26.79) | -11.45 (-14.91, -7.84) |
| Huishui | Guizhou | 1.53 (0.49, 2.59) | 4.32 (-3.28, 12.52) | 2.72 (-7.78, 14.42) | -0.69 (-4.80, 3.59) |
| Jiangkou | Guizhou | -4.30 (-7.78, -0.70) | 9.70 (-33.29, 80.39) | 2.91 (-0.35, 6.28) | -2.10 (-11.51, 8.31) |
| Jianhe | Guizhou | -3.89 (-5.74, -2.00) | -3.21 (-14.66, 9.78) | -0.80 (-18.14, 20.22) | 1.15 (-4.51, 7.14) |
| Jinping | Guizhou | -6.96 (-9.06, -4.81) | -4.85 (-22.08, 16.19) | -5.60 (-22.75, 15.35) | -0.80 (-18.70, 21.04) |
| Jinsha | Guizhou | -3.90 (-5.71, -2.05) | 1.06 (-19.14, 26.31) | -2.55 (-11.29, 7.06) | -10.44 (-14.39, -6.30) |
| Kaili | Guizhou | -4.94 (-6.70, -3.15) | -1.16 (-9.23, 7.63) | -4.27 (-9.65, 1.44) | 0.99 (-9.91, 13.22) |
| Kaiyang | Guizhou | -1.44 (-2.67, -0.21) | -0.28 (-8.64, 8.86) | 1.91 (-6.48, 11.06) | -7.94 (-13.48, -2.04) |
| Leishan | Guizhou | -2.27 (-5.92, 1.52) | -24.09 (-43.52, 2.01) | 1.25 (-8.16, 11.62) | -0.82 (-21.70, 25.64) |
| Libo | Guizhou | 2.86 (-0.49, 6.33) | -5.99 (-25.94, 19.34) | 16.77 (0.92, 35.11) | -5.34 (-22.9, 16.22) |
| Liping | Guizhou | -3.96 (-6.87, -0.95) | -2.93 (-29.09, 32.89) | -6.39 (-21.86, 12.13) | -0.95 (-18.09, 19.78) |
| Liuzhite | Guizhou | -3.40 (-5.16, -1.60) | -1.60 (-15.63, 14.76) | -10.17 (-20.72, 1.79) | -4.54 (-16.56, 9.22) |
| Longli | Guizhou | -1.46 (-3.69, 0.82) | -5.54 (-29.97, 27.41) | 3.11 (-4.00, 10.75) | -1.49 (-10.82, 8.83) |
| Luodian | Guizhou | 0.55 (-2.61, 3.81) | 21.31 (8.56, 35.56) | -2.00 (-18.57, 17.95) | -12.64 (-23.04, -0.85) |
| Majiang | Guizhou | -2.09 (-4.07, -0.07) | -2.99 (-16.22, 12.34) | 7.22 (-8.58, 25.75) | -8.09 (-16.59, 1.26) |
| Meitan | Guizhou | -2.42 (-4.17, -0.65) | -5.00 (-17.84, 9.83) | -0.58 (-15.40, 16.84) | -10.32 (-14.99, -5.40) |
| Nayong | Guizhou | 2.22 (-1.05, 5.59) | 12.55 (-21.76, 61.91) | 17.04 (6.53, 28.59) | -2.62 (-17.28, 14.65) |
| Panzhou | Guizhou | -2.86 (-5.05, -0.62) | -0.34 (-9.87, 10.18) | 0.39 (-19.07, 24.54) | -14.71 (-24.98, -3.04) |
| Pingba | Guizhou | -8.97 (-11.7, -6.15) | 0.61 (-16.93, 21.85) | -13.67 (-31.27, 8.44) | 3.78 (-11.35, 21.49) |
| Pingtang | Guizhou | -3.13 (-5.02, -1.20) | 5.36 (-13.02, 27.63) | -6.65 (-18.96, 7.53) | -1.03 (-11.26, 10.38) |
| Puan | Guizhou | -0.53 (-2.52, 1.50) | 1.64 (-20.02, 29.16) | 3.61 (-0.26, 7.62) | -8.46 (-9.25, -7.67) |
| Puding | Guizhou | -3.19 (-4.96, -1.40) | 5.21 (-0.13, 10.84) | -5.33 (-20.40, 12.59) | -8.43 (-20.60, 5.59) |
| Qianxi | Guizhou | -0.04 (-2.50, 2.48) | 5.77 (-23.59, 46.42) | -1.14 (-10.30, 8.96) | -2.40 (-11.14, 7.21) |
| Qinglong | Guizhou | -3.19 (-4.72, -1.64) | -2.16 (-16.23, 14.26) | -3.30 (-8.89, 2.64) | -5.54 (-18.65, 9.68) |
| Qingzhen | Guizhou | -2.77 (-5.17, -0.31) | 8.62 (-1.82, 20.18) | -1.07 (-10.94, 9.89) | -15.49 (-19.61, -11.16) |
| Qixingguan | Guizhou | -1.39 (-3.75, 1.03) | -6.94 (-16.31, 3.49) | -5.98 (-16.77, 6.20) | 14.54 (6.69, 22.98) |
| Renhuai | Guizhou | -3.79 (-7.67, 0.25) | -0.25 (-9.90, 10.43) | 11.74 (-32.05, 83.76) | -2.70 (-16.27, 13.06) |
| Rongjiang | Guizhou | -2.42 (-4.15, -0.67) | 0.86 (-12.66, 16.48) | 1.54 (-10.4, 15.08) | 0.19 (-11.78, 13.79) |
| Sandou | Guizhou | 3.75 (1.28, 6.27) | 21.88 (12.42, 32.14) | 5.53 (0.14, 11.21) | -2.46 (-20.08, 19.03) |
| Sansui | Guizhou | -6.22 (-8.45, -3.93) | -7.27 (-16.71, 3.24) | 4.37 (-5.90, 15.76) | -10.14 (-22.08, 3.64) |
| Shibing | Guizhou | -4.39 (-6.81, -1.89) | -7.32 (-19.13, 6.21) | -6.25 (-13.54, 1.66) | -11.21 (-25.38, 5.65) |
| Shiqian | Guizhou | -4.29 (-6.33, -2.21) | 5.97 (-3.73, 16.65) | 3.62 (-2.95, 10.63) | -12.59 (-21.05, -3.22) |
| Shuicheng | Guizhou | 1.27 (-1.50, 4.11) | 3.45 (-21.87, 36.97) | 16.80 (0.20, 36.15) | -3.52 (-5.71, -1.27) |
| Sinan | Guizhou | -4.99 (-7.95, -1.93) | 13.05 (-11.67, 44.70) | -6.19 (-20.69, 10.97) | 4.24 (-9.49, 20.06) |
| Songtao | Guizhou | -3.43 (-5.65, -1.16) | 6.15 (-13.80, 30.73) | 6.34 (0.98, 11.97) | -8.06 (-15.11, -0.43) |
| Suiyang | Guizhou | -2.02 (-3.91, -0.08) | 5.60 (-5.40, 17.89) | -0.04 (-17.20, 20.67) | -9.58 (-16.10, -2.54) |
| Taijiang | Guizhou | 2.56 (-0.50, 5.73) | 6.44 (-7.01, 21.84) | 7.26 (-17.05, 38.69) | -8.47 (-20.29, 5.11) |
| Tianzhu | Guizhou | -2.68 (-4.07, -1.26) | 2.06 (-0.90, 5.10) | -1.22 (-17.23, 17.89) | -6.25 (-14.41, 2.70) |
| Tongzi | Guizhou | -2.43 (-4.93, 0.13) | 5.04 (-1.45, 11.96) | -2.04 (-12.53, 9.71) | -15.28 (-24.34, -5.15) |
| Wangmo | Guizhou | 5.82 (-0.20, 12.2) | -0.25 (-30.96, 44.12) | 54.27 (23.99, 91.95) | -2.79 (-7.05, 1.67) |
| Weining | Guizhou | 3.00 (0.39, 5.67) | 15.75 (-8.61, 46.61) | 10.57 (-0.55, 22.93) | -6.30 (-12.92, 0.82) |
| Wengan | Guizhou | -2.07 (-5.28, 1.24) | 12.02 (-10.95, 40.91) | 0.28 (-14.51, 17.62) | -14.54 (-20.01, -8.70) |
| Wuchuan | Guizhou | -0.08 (-3.12, 3.06) | 2.89 (-12.65, 21.19) | 11.00 (2.21, 20.54) | -19.62 (-27.58, -10.79) |
| Xifeng | Guizhou | -5.53 (-6.75, -4.30) | -3.13 (-4.36, -1.89) | -0.91 (-6.13, 4.60) | -12.54 (-19.75, -4.69) |
| Xingren | Guizhou | -2.39 (-4.01, -0.74) | 9.52 (8.61, 10.43) | 0.76 (-7.29, 9.50) | -1.85 (-13.34, 11.16) |
| Xingyi | Guizhou | -8.53 (-10.87, -6.14) | 1.79 (-10.69, 16.02) | -3.86 (-23.26, 20.45) | -0.83 (-5.94, 4.56) |
| Xishui | Guizhou | -4.11 (-6.23, -1.93) | 0.77 (-18.36, 24.39) | -9.15 (-23.45, 7.82) | -10.21 (-16.36, -3.62) |
| Xiuwen | Guizhou | -3.40 (-5.72, -1.02) | 9.18 (-1.95, 21.56) | -3.19 (-16.00, 11.56) | -15.54 (-24.08, -6.04) |
| Yanhe | Guizhou | -2.61 (-4.91, -0.26) | 5.04 (1.42, 8.80) | 2.75 (-5.40, 11.61) | -8.16 (-14.89, -0.89) |
| Yinjiang | Guizhou | -2.49 (-4.42, -0.52) | 12.25 (1.97, 23.56) | 1.90 (-4.21, 8.41) | -7.49 (-14.31, -0.12) |
| Yuping | Guizhou | -8.91 (-12.22, -5.48) | -7.56 (-18.33, 4.63) | -6.78 (-14.48, 1.62) | -3.98 (-11.73, 4.45) |
| Yuqing | Guizhou | -7.82 (-9.17, -6.46) | -5.74 (-20.15, 11.27) | -3.23 (-12.28, 6.75) | -5.65 (-9.83, -1.27) |
| Zhenfeng | Guizhou | -0.96 (-3.40, 1.54) | 6.87 (-12.37, 30.34) | 14.89 (0.80, 30.95) | 0.11 (-6.72, 7.43) |
| Zhengan | Guizhou | -0.16 (-1.56, 1.27) | -2.49 (-12.43, 8.57) | -2.46 (-11.80, 7.87) | -6.94 (-10.57, -3.17) |
| Zhenning | Guizhou | 0.39 (-0.82, 1.62) | 1.89 (-6.93, 11.56) | 3.05 (-2.85, 9.31) | -3.63 (-11.03, 4.38) |
| Zhenyuan | Guizhou | -6.81 (-8.17, -5.44) | -9.16 (-13.66, -4.44) | -3.37 (-19.18, 15.54) | -7.80 (-13.24, -2.02) |
| Zhijin | Guizhou | 4.42 (1.27, 7.67) | 18.51 (0.75, 39.40) | 2.31 (-4.47, 9.58) | -8.78 (-17.56, 0.92) |
| Ziyun | Guizhou | -3.11 (-5.15, -1.03) | 7.18 (-5.87, 22.03) | 0.43 (-8.40, 10.11) | -7.93 (-14.12, -1.30) |
| Baisha | Hainan | -2.79 (-5.68, 0.19) | -14.05 (-31.82, 8.36) | -5.55 (-21.00, 12.92) | 12.24 (-4.44, 31.82) |
| Baoting | Hainan | -0.85 (-2.05, 0.36) | 2.72 (-9.99, 17.23) | 0.64 (-5.56, 7.24) | 2.27 (-6.58, 11.95) |
| Changjiang | Hainan | -3.36 (-6.04, -0.59) | -14.65 (-25.28, -2.49) | -3.59 (-28.83, 30.59) | -7.20 (-17.76, 4.71) |
| Chengmai | Hainan | -6.89 (-9.02, -4.72) | -5.69 (-13.06, 2.32) | -2.94 (-17.79, 14.58) | -16.55 (-30.59, 0.33) |
| Dingan | Hainan | -5.06 (-6.65, -3.45) | 0.54 (-2.14, 3.30) | -1.91 (-7.58, 4.09) | 0.37 (-11.20, 13.43) |
| Dongfang | Hainan | -2.65 (-5.98, 0.80) | -3.13 (-18.68, 15.40) | 10.79 (1.28, 21.20) | -23.98 (-32.74, -14.08) |
| Ledong | Hainan | -1.29 (-2.49, -0.08) | -8.06 (-14.52, -1.12) | 0.59 (-2.20, 3.46) | 0.31 (-9.35, 10.99) |
| Lingao | Hainan | -2.10 (-4.44, 0.29) | -8.08 (-24.53, 11.95) | -6.26 (-20.63, 10.70) | 3.98 (-8.06, 17.60) |
| Lingshui | Hainan | 3.70 (2.07, 5.37) | 9.95 (-3.25, 24.95) | 3.79 (0.07, 7.65) | 7.83 (-8.91, 27.65) |
| Qionghai | Hainan | -6.71 (-8.66, -4.71) | -14.75 (-29.00, 2.35) | -9.42 (-15.78, -2.58) | -2.08 (-8.99, 5.34) |
| Qiongzhong | Hainan | 2.17 (-0.23, 4.64) | 14.78 (-2.68, 35.38) | -0.09 (-18.63, 22.67) | -2.17 (-11.63, 8.31) |
| Tunchang | Hainan | -5.83 (-8.26, -3.33) | -10.67 (-21.09, 1.13) | -4.06 (-27.71, 27.32) | 8.50 (1.99, 15.43) |
| Wanning | Hainan | -4.81 (-8.39, -1.08) | 9.18 (-5.95, 26.73) | 0.50 (-8.88, 10.84) | -23.75 (-34.25, -11.57) |
| Wenchang | Hainan | -6.78 (-9.07, -4.42) | -5.77 (-17.89, 8.14) | -13.99 (-23.11, -3.78) | 5.63 (-2.25, 14.16) |
| Wuzhishan | Hainan | 0.95 (-1.61, 3.57) | -2.83 (-11.84, 7.10) | 1.42 (-9.65, 13.84) | 13.70 (-5.54, 36.86) |
| Anguo | Hebei | -3.82 (-5.51, -2.09) | 0.33 (-12.48, 15.01) | -8.55 (-17.49, 1.35) | -2.69 (-17.42, 14.66) |
| Anping | Hebei | -0.12 (-2.74, 2.56) | 10.72 (3.01, 19.01) | 4.43 (-10.69, 22.10) | -14.98 (-23.39, -5.66) |
| Anxin | Hebei | -5.50 (-6.98, -4.01) | 3.69 (-8.61, 17.65) | -4.85 (-7.93, -1.66) | -9.41 (-17.99, 0.07) |
| Baixiang | Hebei | -1.12 (-3.28, 1.09) | 5.69 (-2.57, 14.66) | -1.09 (-7.87, 6.19) | -10.08 (-18.49, -0.79) |
| Bazhou | Hebei | -7.31 (-10.04, -4.50) | -0.09 (-12.81, 14.50) | -6.52 (-12.09, -0.61) | -22.08 (-36.78, -3.96) |
| Botou | Hebei | -5.56 (-8.32, -2.71) | 2.75 (-10.09, 17.43) | -3.63 (-6.12, -1.08) | -11.20 (-23.04, 2.46) |
| Boye | Hebei | -3.98 (-7.88, 0.09) | 3.35 (-12.05, 21.44) | -2.15 (-27.78, 32.58) | -5.24 (-24.18, 18.42) |
| Cang | Hebei | -5.69 (-8.02, -3.30) | -1.99 (-5.24, 1.37) | -4.80 (-16.82, 8.95) | -1.01 (-11.39, 10.58) |
| Caofeidian | Hebei | -5.41 (-9.56, -1.08) | -2.88 (-10.26, 5.11) | 9.60 (-30.40, 72.61) | 12.09 (-8.93, 37.97) |
| Changli | Hebei | -0.48 (-2.15, 1.22) | 10.58 (3.60, 18.02) | -6.45 (-14.57, 2.44) | 2.73 (-4.92, 11.00) |
| Chengan | Hebei | -3.76 (-5.80, -1.67) | 11.71 (0.83, 23.76) | -10.53 (-18.68, -1.56) | -4.23 (-7.65, -0.68) |
| Chengde | Hebei | -3.78 (-5.27, -2.27) | -8.72 (-20.75, 5.13) | 2.81 (-5.26, 11.56) | -4.89 (-14.54, 5.85) |
| Chicheng | Hebei | -1.54 (-3.19, 0.13) | 3.33 (-4.26, 11.53) | -7.70 (-13.79, -1.19) | -2.74 (-15.54, 12.01) |
| Chongli | Hebei | -4.93 (-7.77, -2.01) | 7.83 (-1.02, 17.49) | -1.14 (-10.18, 8.81) | 0.17 (-21.40, 27.65) |
| Ci | Hebei | -4.42 (-7.21, -1.54) | 5.01 (-8.37, 20.34) | 9.02 (-9.02, 30.65) | -7.89 (-29.81, 20.89) |
| Dachang | Hebei | -2.74 (-5.17, -0.25) | -2.86 (-26.87, 29.03) | -5.71 (-25.15, 18.79) | -1.03 (-16.73, 17.63) |
| Dacheng | Hebei | -4.66 (-6.95, -2.32) | 2.59 (-11.73, 19.24) | -1.87 (-7.50, 4.11) | -16.19 (-27.53, -3.07) |
| Daming | Hebei | -3.28 (-5.79, -0.70) | -2.36 (-22.98, 23.79) | -7.11 (-10.63, -3.44) | 0.12 (-7.71, 8.61) |
| Dingxing | Hebei | -3.27 (-6.29, -0.15) | 14.48 (-13.70, 51.86) | -2.06 (-8.10, 4.37) | -6.97 (-21.21, 9.84) |
| Dingzhou | Hebei | -1.75 (-2.86, -0.62) | -1.42 (-11.94, 10.35) | -2.10 (-4.97, 0.86) | -1.70 (-9.71, 7.02) |
| Dongguang | Hebei | -4.22 (-5.59, -2.84) | 3.52 (-7.06, 15.29) | -3.65 (-8.16, 1.07) | -5.49 (-13.66, 3.45) |
| Feixiang | Hebei | -3.70 (-5.99, -1.35) | 8.67 (1.99, 15.78) | -0.45 (-6.48, 5.98) | -5.28 (-10.08, -0.22) |
| Fengning | Hebei | -6.85 (-8.26, -5.43) | -4.47 (-14.25, 6.44) | -12.02 (-19.11, -4.31) | -12.3 (-22.44, -0.83) |
| Fucheng | Hebei | -1.72 (-4.59, 1.23) | -10.39 (-19.16, -0.66) | -0.85 (-20.64, 23.89) | 3.79 (-11.84, 22.19) |
| Funing | Hebei | -3.09 (-6.95, 0.93) | 7.47 (-13.98, 34.28) | -11.67 (-26.84, 6.63) | 20.46 (-5.47, 53.51) |
| Fuping | Hebei | 0.78 (-0.82, 2.40) | 3.55 (-6.50, 14.68) | -2.60 (-8.49, 3.68) | 8.53 (-4.01, 22.71) |
| Gaobeidian | Hebei | -4.04 (-6.30, -1.72) | 0.86 (-19.92, 27.03) | -12.34 (-20.53, -3.31) | 0.95 (-14.97, 19.86) |
| Gaocheng | Hebei | -4.93 (-7.26, -2.55) | 3.90 (-1.90, 10.05) | -9.07 (-12.77, -5.21) | 4.33 (-8.87, 19.45) |
| Gaoyang | Hebei | -6.66 (-8.72, -4.54) | -1.39 (-12.02, 10.52) | -15.41 (-26.39, -2.79) | -9.64 (-21.68, 4.25) |
| Gaoyi | Hebei | -8.10 (-10.34, -5.81) | 1.91 (-7.26, 11.99) | -15.24 (-25.75, -3.24) | -6.48 (-24.11, 15.24) |
| Guan | Hebei | -2.99 (-4.53, -1.42) | 0.88 (-5.03, 7.17) | -4.75 (-8.95, -0.36) | -12.93 (-22.34, -2.37) |
| Guangping | Hebei | 0.10 (-1.40, 1.63) | 4.38 (-7.36, 17.61) | 4.22 (1.57, 6.93) | -1.58 (-11.29, 9.19) |
| Guangzong | Hebei | -3.19 (-5.89, -0.41) | 9.47 (-6.95, 28.78) | -9.87 (-25.94, 9.68) | -10.82 (-25.29, 6.45) |
| Guantao | Hebei | -7.37 (-9.84, -4.83) | 0.08 (-7.86, 8.71) | -6.59 (-24.48, 15.55) | 8.64 (-7.10, 27.04) |
| Gucheng | Hebei | -4.58 (-6.96, -2.14) | 5.01 (-7.94, 19.77) | -2.17 (-16.61, 14.79) | -5.94 (-10.99, -0.59) |
| Guyuan | Hebei | -4.60 (-5.84, -3.35) | -2.61 (-10.19, 5.61) | -6.51 (-13.48, 1.01) | -7.70 (-12.90, -2.18) |
| Haixing | Hebei | -2.42 (-4.05, -0.76) | -6.02 (-18.5, 8.36) | 2.49 (-5.13, 10.72) | -7.90 (-20.68, 6.94) |
| Hejian | Hebei | -5.71 (-8.18, -3.17) | 2.11 (-16.76, 25.25) | -2.32 (-5.06, 0.51) | -9.60 (-31.27, 18.91) |
| Huaian | Hebei | -0.40 (-2.29, 1.53) | 4.06 (-4.56, 13.47) | 2.34 (-3.03, 8.01) | -4.83 (-22.45, 16.79) |
| Huailai | Hebei | -3.72 (-5.87, -1.53) | 1.12 (-20.40, 28.47) | -1.74 (-7.36, 4.21) | -5.55 (-18.87, 9.96) |
| Huanghua | Hebei | -1.86 (-3.35, -0.35) | -1.66 (-13.19, 11.41) | 3.16 (-4.78, 11.76) | -6.03 (-19.63, 9.87) |
| Jing | Hebei | -3.86 (-4.91, -2.79) | -0.45 (-11.43, 11.91) | 0.50 (-5.05, 6.38) | -5.33 (-10.49, 0.13) |
| Jingxing | Hebei | -5.23 (-7.20, -3.23) | 2.24 (-3.84, 8.70) | -3.62 (-7.02, -0.10) | -15.80 (-19.48, -11.96) |
| Jinzhou | Hebei | -9.69 (-11.87, -7.45) | 2.03 (-7.97, 13.11) | -19.98 (-27.09, -12.17) | -2.82 (-14.11, 9.95) |
| Jize | Hebei | -1.94 (-3.84, 0.00) | 6.33 (1.23, 11.67) | -7.81 (-24.87, 13.14) | 2.41 (-1.60, 6.59) |
| Jizhou | Hebei | -2.14 (-4.29, 0.06) | -0.39 (-10.37, 10.69) | 5.69 (-9.28, 23.14) | -11.99 (-24.49, 2.59) |
| Julu | Hebei | 0.23 (-1.99, 2.51) | 8.37 (-3.05, 21.13) | -1.93 (-6.91, 3.32) | -12.29 (-20.12, -3.70) |
| Kangbao | Hebei | -3.00 (-6.60, 0.75) | 15.71 (-9.44, 47.86) | -5.26 (-12.51, 2.59) | 2.95 (-6.10, 12.87) |
| Kuanchen | Hebei | -2.09 (-4.30, 0.17) | 11.54 (3.42, 20.30) | -12.13 (-21.88, -1.17) | -4.08 (-16.37, 10.01) |
| Laishui | Hebei | 0.97 (-0.78, 2.75) | 3.71 (-8.19, 17.16) | 0.24 (-3.47, 4.09) | -0.54 (-22.10, 26.97) |
| Laiyuan | Hebei | -1.04 (-3.68, 1.67) | -1.58 (-15.35, 14.42) | 7.89 (-12.06, 32.38) | -0.51 (-13.04, 13.81) |
| Leting | Hebei | -5.84 (-9.21, -2.35) | 8.43 (-18.96, 45.08) | -2.55 (-20.49, 19.45) | -11.11 (-31.02, 14.54) |
| Li | Hebei | -1.30 (-5.15, 2.70) | -10.61 (-19.12, -1.20) | -3.95 (-19.66, 14.83) | -15.55 (-18.01, -13.02) |
| Lincheng | Hebei | -4.34 (-6.75, -1.86) | 8.72 (-7.62, 27.95) | -0.53 (-4.70, 3.82) | -13.44 (-22.27, -3.61) |
| Lingshou | Hebei | -5.62 (-7.58, -3.61) | 0.05 (-12.46, 14.35) | -7.69 (-19.56, 5.93) | -1.18 (-9.76, 8.22) |
| Linxi | Hebei | -1.86 (-4.34, 0.69) | -0.38 (-19.57, 23.39) | -10.18 (-14.98, -5.11) | 12.24 (-6.85, 35.24) |
| Linzhang | Hebei | -5.72 (-8.70, -2.64) | 9.43 (-3.53, 24.12) | -15.51 (-26.40, -3.01) | 11.51 (0.46, 23.79) |
| Longhua | Hebei | -4.98 (-7.05, -2.85) | -1.14 (-8.90, 7.28) | -16.97 (-28.69, -3.32) | 2.50 (-5.71, 11.43) |
| Longyao | Hebei | -2.29 (-4.78, 0.26) | 3.95 (-9.82, 19.82) | 3.48 (1.39, 5.61) | -15.83 (-23.02, -7.96) |
| Luancheng | Hebei | -4.04 (-6.83, -1.17) | 5.31 (-5.00, 16.73) | -4.31 (-8.97, 0.58) | -18.57 (-27.76, -8.21) |
| Luannan | Hebei | -5.74 (-7.52, -3.91) | -1.94 (-8.24, 4.80) | 4.50 (-12.73, 25.15) | -6.08 (-16.48, 5.61) |
| Luanping | Hebei | -6.77 (-8.49, -5.02) | -5.09 (-16.81, 8.29) | -8.54 (-19.9, 4.42) | 1.04 (-14.79, 19.81) |
| Luanzhou | Hebei | -8.04 (-10.04, -6.00) | -3.59 (-15.97, 10.61) | -1.91 (-10.99, 8.10) | 2.77 (-10.69, 18.27) |
| Lulong | Hebei | -0.24 (-2.05, 1.61) | -0.86 (-13.67, 13.84) | 5.38 (-6.36, 18.60) | -0.06 (-14.78, 17.21) |
| Luquan | Hebei | -5.44 (-7.00, -3.84) | 1.84 (-4.15, 8.21) | -6.32 (-11.43, -0.91) | 4.96 (-3.35, 13.98) |
| Mancheng | Hebei | -1.60 (-4.72, 1.61) | 8.25 (0.02, 17.16) | -9.83 (-29.69, 15.64) | -0.34 (-15.91, 18.10) |
| Mengcun | Hebei | -1.80 (-3.92, 0.37) | 1.03 (-20.42, 28.27) | -11.33 (-18.49, -3.55) | 1.07 (-8.35, 11.46) |
| Nangong | Hebei | -3.07 (-5.38, -0.70) | 6.35 (-0.03, 13.13) | -6.99 (-21.37, 10.03) | -8.94 (-14.99, -2.45) |
| Nanhe | Hebei | -0.30 (-3.42, 2.92) | 7.56 (-4.55, 21.20) | 7.03 (2.68, 11.56) | -20.57 (-25.47, -15.35) |
| Nanpi | Hebei | -2.38 (-4.87, 0.18) | 6.42 (-3.17, 16.97) | -2.58 (-7.15, 2.22) | -11.74 (-20.57, -1.92) |
| Neiqiu | Hebei | -4.90 (-6.67, -3.10) | -2.69 (-15.2, 11.66) | -2.20 (-9.21, 5.34) | -14.73 (-26.69, -0.83) |
| Ningjin | Hebei | -4.75 (-7.18, -2.25) | 5.71 (-4.34, 16.80) | -1.88 (-3.32, -0.42) | -18.55 (-26.04, -10.30) |
| Pingquan | Hebei | -3.36 (-5.57, -1.10) | -8.89 (-27.93, 15.17) | -4.99 (-22.06, 15.8) | 1.96 (-8.37, 13.45) |
| Pingshan | Hebei | -5.34 (-8.15, -2.45) | 0.06 (-9.63, 10.78) | -22.47 (-37.97, -3.10) | -2.04 (-10.72, 7.48) |
| Pingxiang | Hebei | -0.05 (-2.37, 2.33) | 1.11 (-13.64, 18.39) | 5.46 (-8.90, 22.09) | -0.38 (-7.02, 6.74) |
| Qianan | Hebei | -3.35 (-4.90, -1.77) | 2.72 (-10.64, 18.08) | -1.51 (-11.50, 9.61) | 2.30 (-4.21, 9.24) |
| Qianxi | Hebei | -7.20 (-8.98, -5.39) | -9.45 (-24.39, 8.45) | -6.46 (-20.50, 10.06) | -13.08 (-20.14, -5.41) |
| Qing | Hebei | -5.46 (-8.17, -2.67) | 0.54 (-16.15, 20.56) | -6.16 (-13.97, 2.36) | 1.27 (-7.84, 11.28) |
| Qinghe | Hebei | -7.74 (-9.94, -5.50) | 1.17 (-22.46, 32.00) | -13.59 (-20.79, -5.74) | -2.44 (-14.70, 11.59) |
| Qinglong | Hebei | -2.28 (-3.71, -0.83) | -7.40 (-12.17, -2.36) | -1.64 (-8.90, 6.20) | 5.22 (-1.15, 12.00) |
| Qingyuan | Hebei | -1.52 (-5.39, 2.51) | -11.05 (-30.15, 13.28) | -1.97 (-10.22, 7.04) | 16.01 (-14.35, 57.14) |
| Qiu | Hebei | -0.91 (-3.47, 1.72) | 5.57 (-21.47, 41.92) | 3.92 (-4.21, 12.73) | -6.36 (-19.88, 9.43) |
| Quyang | Hebei | -4.32 (-7.35, -1.18) | -5.43 (-17.44, 8.33) | -3.84 (-11.32, 4.26) | 12.3 (-23.64, 65.16) |
| Quzhou | Hebei | -4.41 (-7.39, -1.33) | 8.31 (-4.42, 22.74) | -11.52 (-30.87, 13.25) | -1.12 (-7.48, 5.69) |
| Raoyang | Hebei | -1.93 (-3.28, -0.57) | -2.16 (-9.95, 6.31) | 4.38 (-2.62, 11.87) | 1.10 (-8.49, 11.7) |
| Ren | Hebei | -4.77 (-8.02, -1.41) | 11.44 (6.20, 16.93) | -4.79 (-8.22, -1.22) | -20.75 (-34.81, -3.67) |
| Renqiu | Hebei | -4.98 (-7.04, -2.87) | 5.65 (-7.79, 21.06) | -3.70 (-10.45, 3.56) | -12.37 (-18.34, -5.96) |
| Rongcheng | Hebei | -2.80 (-5.30, -0.22) | -9.41 (-19.7, 2.20) | -8.54 (-20.02, 4.59) | -3.13 (-11.09, 5.54) |
| Sanhe | Hebei | -2.53 (-3.81, -1.23) | -0.87 (-5.90, 4.43) | -7.97 (-22.11, 8.74) | -0.99 (-5.44, 3.68) |
| Shahe | Hebei | -4.86 (-7.40, -2.25) | 6.21 (2.06, 10.52) | 1.79 (-4.85, 8.88) | -16.50 (-27.64, -3.64) |
| Shangyi | Hebei | 2.39 (-3.34, 8.47) | 43.35 (14.47, 79.52) | 2.07 (-9.30, 14.87) | -14.42 (-31.31, 6.62) |
| She | Hebei | -4.21 (-6.70, -1.66) | -4.60 (-19.31, 12.78) | -15.42 (-22.34, -7.89) | 5.77 (-12.34, 27.63) |
| Shenze | Hebei | -8.62 (-12.19, -4.90) | 9.26 (1.59, 17.51) | -23.67 (-49.73, 15.90) | -4.52 (-15.43, 7.80) |
| Shenzhou | Hebei | -1.17 (-2.25, -0.08) | 1.21 (-10.45, 14.39) | -4.30 (-11.40, 3.36) | -1.30 (-6.89, 4.63) |
| Shunping | Hebei | -3.45 (-5.93, -0.90) | -2.91 (-9.66, 4.34) | -4.79 (-17.81, 10.3) | 16.49 (2.81, 32.00) |
| Suning | Hebei | -4.85 (-8.01, -1.59) | 3.98 (-17.36, 30.82) | 1.05 (-4.77, 7.22) | -20.15 (-32.18, -5.99) |
| Tang | Hebei | -5.61 (-7.67, -3.50) | -5.62 (-17.76, 8.33) | -9.12 (-15.82, -1.89) | 7.15 (-3.82, 19.38) |
| Tangxian | Hebei | -1.20 (-3.09, 0.72) | 2.15 (-13.10, 20.07) | 2.33 (-3.33, 8.31) | -6.97 (-22.03, 11.00) |
| Wangdou | Hebei | -5.57 (-8.35, -2.70) | -1.51 (-12.00, 10.24) | -12.64 (-28.91, 7.37) | 13.83 (-4.73, 35.99) |
| Wanquan | Hebei | -1.88 (-4.29, 0.60) | 6.31 (-7.04, 21.58) | -3.97 (-16.57, 10.53) | -13.17 (-19.39, -6.48) |
| Wei | Hebei | -3.12 (-5.47, -0.72) | 4.69 (-10.34, 22.23) | -1.30 (-17.68, 18.34) | -3.87 (-9.12, 1.67) |
| Wei | Hebei | -2.07 (-4.23, 0.14) | -9.67 (-21.08, 3.40) | -2.83 (-12.72, 8.18) | 8.48 (-4.60, 23.36) |
| Wei | Hebei | -0.71 (-2.14, 0.74) | 0.18 (-12.81, 15.10) | -6.48 (-8.91, -3.98) | 5.69 (2.38, 9.12) |
| Weichang | Hebei | -4.24 (-6.64, -1.78) | -0.11 (-9.80, 10.62) | -4.22 (-11.12, 3.20) | -7.80 (-24.65, 12.82) |
| Wenan | Hebei | -6.75 (-10.16, -3.21) | -1.25 (-11.41, 10.08) | -2.50 (-6.88, 2.09) | -27.70 (-41.01, -11.39) |
| Wuan | Hebei | -4.25 (-5.88, -2.59) | -2.54 (-13.95, 10.38) | -9.02 (-15.40, -2.15) | 2.42 (-9.57, 16.01) |
| Wuji | Hebei | -6.12 (-9.18, -2.95) | 9.88 (-4.23, 26.07) | -8.52 (-12.27, -4.62) | -7.79 (-18.45, 4.27) |
| Wuqiang | Hebei | -1.21 (-3.31, 0.94) | 0.65 (-3.67, 5.16) | 15.22 (8.85, 21.97) | -9.89 (-15.86, -3.49) |
| Wuqiao | Hebei | -4.79 (-7.31, -2.19) | -2.76 (-8.90, 3.79) | -0.41 (-2.21, 1.43) | -20.38 (-25.56, -14.84) |
| Wuyi | Hebei | -1.61 (-3.57, 0.38) | 3.26 (-3.72, 10.74) | -9.75 (-23.25, 6.12) | -2.32 (-16.14, 13.79) |
| Xian | Hebei | -2.79 (-5.11, -0.41) | 4.51 (-12.00, 24.10) | -1.01 (-2.26, 0.25) | -14.01 (-23.89, -2.84) |
| Xianghe | Hebei | -5.39 (-6.97, -3.78) | -3.81 (-19.24, 14.58) | -6.86 (-14.83, 1.85) | -12.01 (-20.41, -2.71) |
| Xinglong | Hebei | -4.71 (-5.89, -3.52) | -1.88 (-12.75, 10.34) | -6.39 (-15.11, 3.24) | -4.32 (-10.11, 1.85) |
| Xingtai | Hebei | -4.71 (-6.35, -3.05) | 5.12 (-1.83, 12.56) | -8.30 (-14.17, -2.02) | 0.52 (-12.26, 15.15) |
| Xinhe | Hebei | -3.01 (-5.89, -0.05) | 3.30 (-2.97, 9.97) | -2.61 (-13.78, 10.00) | -15.83 (-34.57, 8.28) |
| Xinji | Hebei | -5.31 (-8.59, -1.92) | 2.48 (-12.77, 20.39) | -5.91 (-40.03, 47.61) | -14.90 (-25.10, -3.31) |
| Xinle | Hebei | -5.88 (-7.13, -4.61) | -4.91 (-17.69, 9.85) | -4.00 (-7.48, -0.40) | -9.40 (-14.06, -4.49) |
| Xiong | Hebei | -4.64 (-6.92, -2.31) | 7.68 (1.53, 14.19) | -0.48 (-23.3, 29.13) | -8.02 (-12.88, -2.88) |
| Xushui | Hebei | -3.53 (-5.09, -1.94) | -4.81 (-9.16, -0.26) | 0.78 (-8.06, 10.48) | -12.98 (-22.13, -2.75) |
| Yangyuan | Hebei | -3.19 (-4.74, -1.61) | 5.87 (1.17, 10.79) | -7.79 (-10.99, -4.48) | -2.11 (-11.78, 8.63) |
| Yanshan | Hebei | -5.59 (-7.10, -4.06) | -7.39 (-12.58, -1.88) | -0.16 (-4.08, 3.92) | -10.50 (-22.62, 3.51) |
| Yi | Hebei | -3.58 (-4.62, -2.53) | 2.77 (-1.22, 6.92) | -3.66 (-5.34, -1.95) | -5.49 (-14.11, 4.01) |
| Yongnian | Hebei | -2.66 (-4.11, -1.19) | 6.22 (-4.07, 17.62) | -8.11 (-13.63, -2.24) | 1.46 (-1.77, 4.79) |
| Yongqing | Hebei | -3.59 (-5.42, -1.73) | 1.86 (-6.74, 11.26) | -1.51 (-8.35, 5.83) | -12.34 (-25.23, 2.77) |
| Yuanshi | Hebei | -7.34 (-9.93, -4.68) | 5.69 (-4.80, 17.33) | -14.97 (-22.79, -6.35) | -8.57 (-25.53, 12.26) |
| Yutian | Hebei | -4.71 (-6.20, -3.19) | 2.19 (-1.58, 6.10) | -2.11 (-19.50, 19.03) | -4.36 (-10.51, 2.21) |
| Zanhuang | Hebei | -6.26 (-7.57, -4.92) | 0.74 (-6.93, 9.04) | -10.65 (-12.92, -8.32) | -10.52 (-14.81, -6.01) |
| Zaoqiang | Hebei | -3.58 (-5.52, -1.61) | 5.97 (-2.43, 15.10) | -3.17 (-5.27, -1.02) | -5.15 (-14.92, 5.75) |
| Zhangbei | Hebei | -3.13 (-5.44, -0.77) | 7.69 (-12.73, 32.90) | -7.54 (-13.12, -1.60) | -3.45 (-9.46, 2.96) |
| Zhao | Hebei | -8.09 (-11.10, -4.97) | 11.01 (-16.94, 48.38) | -14.71 (-27.40, 0.20) | -12.58 (-17.92, -6.90) |
| Zhengding | Hebei | -3.81 (-6.21, -1.35) | 3.87 (-2.59, 10.75) | -16.29 (-28.80, -1.60) | 0.37 (-4.03, 4.98) |
| Zhuolu | Hebei | -3.42 (-5.16, -1.63) | 5.56 (-7.56, 20.54) | -3.54 (-6.86, -0.10) | -6.99 (-21.60, 10.35) |
| Zhuozhou | Hebei | -3.48 (-4.92, -2.01) | 1.39 (-8.30, 12.10) | -4.86 (-20.55, 13.93) | 0.23 (-3.27, 3.85) |
| Zunhua | Hebei | -8.37 (-11.67, -4.94) | -13.22 (-27.47, 3.84) | -4.33 (-27.45, 26.16) | 7.61 (-5.18, 22.12) |
| Acheng | Heilongjiang | -4.30 (-5.31, -3.28) | -7.45 (-18.40, 4.96) | -6.32 (-9.65, -2.87) | -6.20 (-11.10, -1.04) |
| Anda | Heilongjiang | -7.60 (-10.34, -4.78) | 6.13 (-13.74, 30.58) | -0.69 (-4.17, 2.93) | -14.18 (-23.25, -4.03) |
| Baiquan | Heilongjiang | -2.74 (-4.89, -0.54) | -6.37 (-14.53, 2.58) | 7.54 (-12.83, 32.66) | -8.30 (-17.91, 2.44) |
| Baoqing | Heilongjiang | -2.27 (-4.90, 0.44) | 0.94 (-19.37, 26.38) | -4.91 (-11.8, 2.53) | -8.33 (-14.66, -1.54) |
| Bayan | Heilongjiang | -5.71 (-7.36, -4.03) | -6.64 (-13.04, 0.23) | -6.43 (-17.52, 6.15) | -7.03 (-25.39, 15.83) |
| Beian | Heilongjiang | -4.73 (-7.18, -2.20) | -8.08 (-21.64, 7.84) | 13.93 (9.35, 18.70) | -9.30 (-20.04, 2.88) |
| Bin | Heilongjiang | -8.22 (-10.31, -6.09) | -7.94 (-15.92, 0.81) | -19.50 (-27.75, -10.30) | 2.83 (-7.77, 14.64) |
| Boli | Heilongjiang | -5.77 (-8.06, -3.42) | -8.73 (-15.11, -1.88) | 4.32 (-10.16, 21.14) | -0.68 (-15.28, 16.44) |
| Dongning | Heilongjiang | -1.58 (-4.32, 1.25) | -2.61 (-12.73, 8.69) | 2.54 (-1.16, 6.37) | -20.31 (-25.93, -14.26) |
| Dorbod | Heilongjiang | -4.06 (-5.22, -2.88) | -3.83 (-10.01, 2.78) | 2.87 (-3.91, 10.14) | -5.95 (-14.61, 3.60) |
| Fangzheng | Heilongjiang | -5.05 (-7.39, -2.64) | -14.34 (-30.55, 5.67) | -1.68 (-12.47, 10.44) | -11.48 (-23.33, 2.21) |
| Fujin | Heilongjiang | -1.05 (-2.80, 0.74) | -7.35 (-19.81, 7.03) | 1.73 (-10.71, 15.90) | 2.27 (-8.25, 13.99) |
| Fuyu | Heilongjiang | -2.46 (-3.92, -0.97) | -4.76 (-18.51, 11.30) | -2.19 (-4.85, 0.54) | -10.71 (-14.29, -6.98) |
| Fuyuan | Heilongjiang | -4.73 (-9.15, -0.09) | -18.1 (-32.45, -0.70) | 2.16 (-33.91, 57.92) | -1.67 (-18.23, 18.25) |
| Gannan | Heilongjiang | -3.23 (-5.32, -1.1) | -5.14 (-15.17, 6.08) | 4.58 (-4.64, 14.68) | -15.94 (-18.16, -13.65) |
| Hailin | Heilongjiang | -6.11 (-9.33, -2.78) | -3.34 (-7.75, 1.29) | -2.77 (-9.78, 4.79) | -26.40 (-30.43, -22.14) |
| Hailun | Heilongjiang | -2.26 (-4.16, -0.31) | 3.54 (-7.51, 15.90) | 2.18 (-4.01, 8.77) | -6.58 (-12.75, 0.02) |
| Huachuan | Heilongjiang | -2.22 (-4.51, 0.13) | -8.56 (-16.34, -0.06) | -0.40 (-16.89, 19.36) | -0.98 (-21.78, 25.35) |
| Huanan | Heilongjiang | -2.98 (-5.38, -0.53) | -15.65 (-29.32, 0.67) | -8.63 (-20.51, 5.03) | 1.16 (-4.36, 7.01) |
| Hulin | Heilongjiang | -2.67 (-5.25, -0.02) | -1.18 (-16.64, 17.14) | 0.93 (-10.26, 13.52) | -12.17 (-22.50, -0.47) |
| Huma | Heilongjiang | -4.20 (-6.52, -1.82) | -1.71 (-18.08, 17.92) | 3.42 (-3.54, 10.87) | -10.20 (-30.77, 16.49) |
| Jiayin | Heilongjiang | -1.12 (-2.90, 0.68) | 3.78 (-1.57, 9.42) | -2.77 (-13.41, 9.17) | -10.66 (-22.10, 2.46) |
| Jidong | Heilongjiang | -4.98 (-6.46, -3.47) | -5.10 (-10.83, 1.00) | -9.87 (-18.89, 0.15) | -9.31 (-23.88, 8.04) |
| Jixian | Heilongjiang | -7.84 (-10.01, -5.61) | -5.46 (-17.87, 8.82) | -8.37 (-16.88, 1.01) | -4.21 (-25.16, 22.60) |
| Kedong | Heilongjiang | -0.54 (-2.45, 1.40) | -6.31 (-23.36, 14.53) | 8.77 (6.54, 11.04) | 2.33 (-1.79, 6.63) |
| Keshan | Heilongjiang | 0.03 (-1.97, 2.06) | -0.09 (-18.93, 23.13) | 6.46 (-7.20, 22.13) | -3.84 (-9.56, 2.24) |
| Lanxi | Heilongjiang | -2.63 (-3.95, -1.29) | -6.14 (-14.93, 3.56) | -4.33 (-7.93, -0.59) | 5.92 (-3.35, 16.08) |
| Lindian | Heilongjiang | -4.92 (-7.31, -2.47) | -14.56 (-25.17, -2.44) | 4.90 (-2.84, 13.25) | -9.08 (-13.58, -4.35) |
| Linkou | Heilongjiang | -3.17 (-5.02, -1.27) | -2.30 (-10.04, 6.09) | 1.37 (-5.31, 8.53) | -14.95 (-24.66, -3.99) |
| Longjiang | Heilongjiang | -0.59 (-2.41, 1.27) | -10.36 (-19.00, -0.81) | -5.75 (-15.31, 4.89) | 4.45 (-3.37, 12.90) |
| Luobei | Heilongjiang | -10.33 (-11.74, -8.90) | -5.78 (-15.11, 4.59) | -12.95 (-23.32, -1.19) | -6.58 (-12.23, -0.56) |
| Mingshui | Heilongjiang | -3.22 (-5.13, -1.27) | -1.36 (-7.42, 5.10) | -5.33 (-19.84, 11.80) | -8.71 (-19.00, 2.88) |
| Mishan | Heilongjiang | -5.77 (-7.18, -4.33) | 0.02 (-11.99, 13.67) | -7.63 (-14.45, -0.26) | -9.50 (-16.43, -2.00) |
| Mohe | Heilongjiang | -8.93 (-11.14, -6.67) | -6.55 (-23.92, 14.79) | -2.82 (-13.50, 9.17) | -13.11 (-31.71, 10.56) |
| Mulan | Heilongjiang | -3.03 (-4.59, -1.45) | -4.88 (-18.88, 11.53) | -0.76 (-7.90, 6.93) | -10.18 (-10.47, -9.89) |
| Muleng | Heilongjiang | -3.57 (-7.07, 0.07) | 7.17 (-2.20, 17.43) | -0.46 (-10.90, 11.22) | -26.21 (-31.73, -20.24) |
| Nehe | Heilongjiang | -0.39 (-2.55, 1.82) | -6.61 (-10.41, -2.65) | 4.35 (-6.89, 16.94) | -4.51 (-17.27, 10.22) |
| Nenjiang | Heilongjiang | -7.76 (-9.15, -6.34) | -12.07 (-21.45, -1.57) | -3.23 (-15.77, 11.18) | -6.84 (-15.63, 2.86) |
| Ningan | Heilongjiang | -5.06 (-7.97, -2.05) | 0.09 (-5.82, 6.37) | 2.53 (-2.49, 7.80) | -18.87 (-28.59, -7.81) |
| Qingan | Heilongjiang | -2.11 (-4.43, 0.28) | 1.52 (-11.25, 16.13) | 2.10 (-11.57, 17.90) | -13.71 (-20.45, -6.38) |
| Qinggang | Heilongjiang | -4.00 (-6.54, -1.39) | -11.23 (-30.05, 12.67) | 2.28 (-13.49, 20.93) | -14.16 (-26.70, 0.52) |
| Raohe | Heilongjiang | -6.46 (-8.58, -4.30) | -8.17 (-18.67, 3.69) | -12.21 (-27.8, 6.75) | -9.24 (-26.24, 11.69) |
| Shangzhi | Heilongjiang | -6.21 (-8.41, -3.97) | -7.63 (-23.21, 11.10) | 2.23 (-12.84, 19.91) | -17.54 (-23.71, -10.87) |
| Shuangcheng | Heilongjiang | -3.28 (-5.65, -0.85) | -8.75 (-23.48, 8.82) | 1.95 (-2.07, 6.15) | -6.94 (-16.52, 3.74) |
| Suibin | Heilongjiang | -7.92 (-10.37, -5.40) | -2.26 (-23.51, 24.90) | -8.70 (-23.52, 9.00) | -17.84 (-30.95, -2.25) |
| Suifenhe | Heilongjiang | -8.71 (-12.21, -5.08) | 0.87 (-8.58, 11.30) | -17.28 (-38.32, 10.94) | -27.47 (-42.25, -8.89) |
| Suileng | Heilongjiang | -1.54 (-3.3, 0.26) | 4.29 (-0.31, 9.11) | 2.21 (-6.82, 12.12) | -1.16 (-11.81, 10.77) |
| Sunwu | Heilongjiang | -9.48 (-11.54, -7.37) | -11.51 (-35.17, 20.79) | -12.71 (-18.33, -6.70) | -7.91 (-20.94, 7.27) |
| Tahe | Heilongjiang | -3.87 (-6.75, -0.89) | -0.80 (-12.44, 12.39) | 0.82 (-16.33, 21.48) | -21.11 (-30.86, -9.98) |
| Tailai | Heilongjiang | -1.68 (-3.32, 0.00) | -11.36 (-19.81, -2.03) | 0.23 (-1.32, 1.80) | 4.01 (-5.82, 14.86) |
| Tangyuan | Heilongjiang | -2.64 (-3.98, -1.28) | -2.31 (-12.57, 9.16) | 2.16 (-6.46, 11.57) | -6.15 (-17.62, 6.92) |
| Tieli | Heilongjiang | -3.99 (-5.95, -2.00) | 0.37 (-10.76, 12.9) | 1.46 (-6.55, 10.15) | -4.65 (-15.93, 8.14) |
| Tonghe | Heilongjiang | -4.68 (-7.43, -1.85) | 3.36 (-17.32, 29.21) | -18.88 (-27.93, -8.70) | -14.55 (-20.61, -8.02) |
| Tongjiang | Heilongjiang | -1.98 (-3.81, -0.12) | -0.18 (-6.50, 6.57) | 1.37 (-13.37, 18.60) | -12.43 (-22.91, -0.51) |
| Wangkui | Heilongjiang | -1.53 (-4.24, 1.27) | 1.15 (-13.98, 18.95) | 9.34 (7.71, 11.00) | -18.02 (-30.32, -3.56) |
| Wuchang | Heilongjiang | -6.23 (-7.78, -4.65) | -4.23 (-16.74, 10.16) | -0.06 (-7.57, 8.05) | -11.15 (-22.29, 1.58) |
| Wudalianchi | Heilongjiang | -5.82 (-7.03, -4.59) | 0.06 (-2.10, 2.28) | -7.19 (-14.74, 1.02) | -8.38 (-14.99, -1.26) |
| Xunke | Heilongjiang | -3.85 (-6.43, -1.20) | -6.48 (-22.02, 12.17) | -1.51 (-21.21, 23.10) | -9.19 (-20.31, 3.48) |
| Yanshou | Heilongjiang | -5.79 (-8.04, -3.49) | -7.50 (-15.89, 1.73) | -14.52 (-23.49, -4.49) | -11.63 (-23.85, 2.54) |
| Yian | Heilongjiang | -1.62 (-3.13, -0.09) | -7.32 (-13.80, -0.35) | -0.66 (-10.48, 10.24) | 0.08 (-13.71, 16.07) |
| Yilan | Heilongjiang | -5.28 (-6.95, -3.59) | -5.56 (-11.05, 0.27) | -0.28 (-6.79, 6.70) | -14.87 (-26.11, -1.92) |
| Youyi | Heilongjiang | -6.13 (-8.67, -3.53) | 6.96 (-9.45, 26.34) | -2.41 (-5.80, 1.10) | -14.36 (-26.43, -0.31) |
| Zhaodong | Heilongjiang | -3.84 (-5.35, -2.31) | 0.42 (-14.00, 17.25) | -1.45 (-13.73, 12.59) | -4.58 (-14.87, 6.94) |
| Zhaoyuan | Heilongjiang | -5.09 (-7.04, -3.09) | -13.32 (-26.75, 2.57) | 1.17 (-11.24, 15.33) | -5.28 (-16.86, 7.91) |
| Zhao州 | Heilongjiang | -4.51 (-7.03, -1.93) | -2.13 (-22.11, 22.97) | 3.43 (-12.57, 22.36) | 1.15 (-5.84, 8.67) |
| Anyang | Henan | -8.69 (-11.49, -5.80) | 1.61 (-6.33, 10.24) | -3.42 (-11.12, 4.95) | -18.70 (-41.91, 13.79) |
| Baofeng | Henan | -5.93 (-8.06, -3.75) | -15.78 (-31.82, 4.04) | -0.58 (-15.23, 16.61) | -3.52 (-13.87, 8.07) |
| Biyang | Henan | -5.75 (-6.46, -5.02) | -8.24 (-10.15, -6.28) | -5.79 (-10.42, -0.91) | -3.35 (-11.52, 5.57) |
| Boai | Henan | -4.54 (-6.16, -2.90) | -7.98 (-15.08, -0.29) | -9.65 (-20.64, 2.87) | 1.60 (-6.57, 10.49) |
| Changge | Henan | -2.54 (-6.13, 1.19) | -24.02 (-41.90, -0.65) | 9.24 (-6.14, 27.14) | -8.10 (-12.64, -3.32) |
| Changyuan | Henan | -4.52 (-5.78, -3.25) | -6.41 (-15.39, 3.53) | 1.62 (-9.74, 14.41) | -4.21 (-8.72, 0.51) |
| Dancheng | Henan | -3.13 (-4.35, -1.88) | -0.98 (-17.58, 18.96) | -4.78 (-8.47, -0.94) | -0.14 (-3.92, 3.78) |
| Dengfeng | Henan | -4.74 (-6.72, -2.72) | -13.5 (-23.11, -2.69) | 4.16 (-8.16, 18.12) | -7.41 (-11.35, -3.29) |
| Dengzhou | Henan | -6.41 (-7.21, -5.60) | -6.70 (-14.54, 1.87) | -8.12 (-12.57, -3.44) | -3.44 (-9.79, 3.36) |
| Fan | Henan | -2.31 (-4.26, -0.31) | -9.52 (-21.68, 4.53) | 7.24 (1.65, 13.14) | -4.60 (-17.69, 10.59) |
| Fangcheng | Henan | -4.87 (-6.10, -3.62) | -3.50 (-14.13, 8.45) | -3.98 (-11.16, 3.77) | -9.72 (-19.96, 1.83) |
| Fengqiu | Henan | -3.80 (-5.05, -2.53) | -7.35 (-19.54, 6.69) | 2.76 (-4.42, 10.47) | -3.37 (-4.59, -2.13) |
| Fugou | Henan | -6.49 (-9.27, -3.63) | -10.00 (-22.98, 5.17) | -18.56 (-37.48, 6.07) | -1.05 (-5.10, 3.17) |
| Gongyi | Henan | -5.93 (-7.01, -4.83) | -10.44 (-21.29, 1.92) | -7.12 (-11.65, -2.37) | -7.78 (-11.44, -3.98) |
| Guangshan | Henan | -5.20 (-6.57, -3.81) | -12.54 (-19.19, -5.34) | -2.62 (-8.88, 4.07) | -7.15 (-12.72, -1.22) |
| Gushi | Henan | -2.86 (-4.83, -0.84) | 5.33 (-11.59, 25.48) | -0.36 (-16.73, 19.23) | 0.73 (-10.82, 13.78) |
| Hua | Henan | -5.87 (-7.07, -4.64) | -4.56 (-13.72, 5.56) | -7.29 (-17.92, 4.72) | -1.69 (-11.06, 8.67) |
| Huaibin | Henan | -0.61 (-2.21, 1.02) | -4.56 (-18.67, 12.00) | 2.33 (-4.46, 9.60) | 3.93 (-10.66, 20.90) |
| Huaiyang | Henan | -4.30 (-5.65, -2.92) | -6.36 (-15.51, 3.78) | -9.01 (-19.91, 3.37) | 1.85 (-3.31, 7.29) |
| Huangchuan | Henan | -4.92 (-6.22, -3.59) | -6.60 (-18.11, 6.53) | -3.77 (-9.8, 2.67) | -3.79 (-5.91, -1.62) |
| Hui | Henan | -2.10 (-3.77, -0.40) | -5.39 (-19.36, 11.01) | 3.05 (0.27, 5.89) | -6.74 (-14.52, 1.76) |
| Huojia | Henan | -2.93 (-4.40, -1.43) | -5.75 (-14.68, 4.12) | 3.11 (-6.40, 13.58) | -1.27 (-10.70, 9.16) |
| Jia | Henan | -4.50 (-6.47, -2.48) | -14.25 (-26.27, -0.28) | -1.87 (-13.33, 11.10) | -6.25 (-14.70, 3.02) |
| Jiyuan | Henan | -3.74 (-5.60, -1.84) | 9.30 (-8.10, 30.00) | -2.09 (-11.64, 8.49) | -9.20 (-20.57, 3.80) |
| Jun | Henan | -0.87 (-2.66, 0.95) | -5.54 (-17.79, 8.53) | 10.31 (-1.93, 24.08) | -5.14 (-7.39, -2.83) |
| Lankao | Henan | -6.36 (-8.08, -4.61) | -14.01 (-25.23, -1.09) | -4.16 (-12.88, 5.44) | -0.85 (-2.43, 0.75) |
| Lingbao | Henan | -4.03 (-5.43, -2.61) | -6.90 (-15.15, 2.17) | 4.20 (-0.92, 9.58) | -6.67 (-18.09, 6.34) |
| Linying | Henan | -12.35 (-15.19, -9.43) | -1.28 (-5.64, 3.28) | -13.3 (-21.57, -4.15) | 2.64 (-14.70, 23.51) |
| Linzhou | Henan | -1.58 (-3.08, -0.05) | -1.75 (-14.93, 13.46) | 2.52 (-9.91, 16.67) | -5.76 (-7.92, -3.55) |
| Luanchuan | Henan | -4.22 (-5.08, -3.35) | -6.27 (-9.64, -2.77) | -6.65 (-12.86, 0.00) | -0.81 (-8.19, 7.16) |
| Luoning | Henan | -1.99 (-3.37, -0.59) | -0.04 (-15.18, 17.80) | -1.67 (-6.49, 3.41) | -4.93 (-15.10, 6.45) |
| Luoshan | Henan | -4.95 (-6.58, -3.30) | -2.19 (-18.51, 17.40) | -1.48 (-9.17, 6.86) | -6.34 (-13.56, 1.49) |
| Lushan | Henan | -4.27 (-5.56, -2.96) | -8.97 (-17.75, 0.76) | 3.46 (-5.33, 13.08) | -3.82 (-6.40, -1.17) |
| Lushi | Henan | -3.86 (-4.97, -2.75) | -3.55 (-17.00, 12.09) | -3.34 (-11.01, 4.98) | -3.71 (-10.51, 3.61) |
| Luyi | Henan | -3.49 (-4.53, -2.43) | 1.07 (-4.33, 6.78) | -2.29 (-5.25, 0.77) | -4.78 (-13.52, 4.85) |
| Mengjin | Henan | -4.55 (-6.49, -2.57) | 5.40 (-15.99, 32.24) | -3.74 (-13.91, 7.64) | -5.31 (-13.47, 3.62) |
| Mengzhou | Henan | -4.85 (-7.03, -2.63) | 9.83 (-13.09, 38.81) | -0.13 (-4.96, 4.96) | -5.42 (-8.71, -2.02) |
| Mianchi | Henan | -4.92 (-6.48, -3.34) | -8.51 (-24.29, 10.57) | 0.35 (-7.87, 9.30) | -7.17 (-16.63, 3.36) |
| Minquan | Henan | -4.92 (-6.63, -3.18) | -5.33 (-14.73, 5.10) | 1.36 (-6.71, 10.13) | 0.93 (-5.54, 7.84) |
| Nanle | Henan | -4.14 (-5.43, -2.83) | -5.66 (-20.42, 11.83) | -7.34 (-14.82, 0.80) | -2.84 (-8.71, 3.41) |
| Nanzhao | Henan | -2.21 (-4.25, -0.12) | -0.91 (-28.11, 36.56) | 0.50 (-2.82, 3.94) | -0.44 (-3.92, 3.18) |
| Neihuang | Henan | -5.54 (-6.70, -4.37) | -1.64 (-8.74, 6.03) | -3.08 (-7.38, 1.42) | -7.07 (-20.30, 8.36) |
| Neixiang | Henan | -4.80 (-6.03, -3.56) | -9.97 (-16.50, -2.93) | -1.64 (-6.60, 3.59) | -1.31 (-4.57, 2.06) |
| Ningling | Henan | -3.12 (-4.71, -1.50) | 3.38 (-1.81, 8.84) | 0.02 (-6.56, 7.06) | -8.57 (-21.61, 6.65) |
| Pingyu | Henan | -5.06 (-5.96, -4.15) | -7.37 (-13.21, -1.13) | -2.22 (-10.61, 6.96) | -5.75 (-8.77, -2.63) |
| Puyang | Henan | -2.29 (-5.31, 0.83) | -10.47 (-21.90, 2.63) | 5.61 (-3.87, 16.04) | -16.94 (-38.53, 12.24) |
| Qi | Henan | -3.58 (-4.96, -2.18) | -5.03 (-12.98, 3.64) | 4.64 (-4.75, 14.95) | -7.75 (-14.81, -0.10) |
| Qi | Henan | -3.71 (-4.91, -2.49) | -6.83 (-8.32, -5.32) | 2.22 (-7.78, 13.30) | -7.30 (-11.87, -2.48) |
| Qingfeng | Henan | -5.09 (-7.61, -2.49) | -17.61 (-30.17, -2.79) | 1.67 (-5.65, 9.55) | -12.82 (-29.51, 7.81) |
| Qinyang | Henan | -2.38 (-3.59, -1.15) | -3.26 (-12.21, 6.61) | 4.12 (0.57, 7.79) | -8.36 (-13.01, -3.47) |
| Queshan | Henan | -3.39 (-4.49, -2.27) | -10.32 (-16.47, -3.72) | -1.12 (-6.86, 4.98) | -5.53 (-9.54, -1.35) |
| Runan | Henan | -4.81 (-5.96, -3.65) | -9.57 (-18.23, 0.00) | -2.32 (-6.59, 2.14) | -2.37 (-5.06, 0.39) |
| Ruyang | Henan | -4.21 (-5.62, -2.78) | -6.09 (-15.98, 4.97) | -1.22 (-9.69, 8.05) | -5.03 (-6.53, -3.50) |
| Ruzhou | Henan | -4.89 (-6.62, -3.12) | 1.76 (-9.55, 14.49) | -4.51 (-17.69, 10.78) | -3.89 (-9.39, 1.94) |
| Shangcai | Henan | -4.82 (-5.54, -4.09) | -7.14 (-10.29, -3.88) | -1.82 (-7.40, 4.09) | -6.14 (-7.10, -5.17) |
| Shangcheng | Henan | -2.91 (-4.06, -1.75) | 1.34 (-13.55, 18.8) | -3.66 (-8.92, 1.90) | -2.95 (-6.22, 0.43) |
| Shangshui | Henan | -2.70 (-4.04, -1.35) | -6.31 (-19.73, 9.36) | 1.42 (-8.99, 13.02) | -0.09 (-3.88, 3.84) |
| Shenqiu | Henan | -3.76 (-5.92, -1.54) | -14.63 (-25.63, -2.01) | 6.03 (-10.41, 25.48) | -7.16 (-10.72, -3.45) |
| Sheqi | Henan | -4.80 (-6.41, -3.17) | 2.64 (-15.15, 24.16) | -8.53 (-13.63, -3.14) | -1.16 (-8.89, 7.22) |
| Song | Henan | -4.07 (-5.94, -2.16) | -6.75 (-25.69, 17.02) | -7.68 (-24.70, 13.19) | -4.64 (-6.31, -2.94) |
| Sui | Henan | -2.77 (-4.50, -1.01) | 4.15 (-11.55, 22.63) | -2.29 (-10.32, 6.47) | -0.89 (-16.31, 17.37) |
| Suiping | Henan | -5.53 (-6.66, -4.38) | -4.44 (-15.84, 8.52) | -1.19 (-8.70, 6.95) | -6.53 (-14.48, 2.17) |
| Taikang | Henan | -0.87 (-2.08, 0.36) | 1.23 (-7.56, 10.85) | 0.63 (-9.23, 11.56) | -5.04 (-13.42, 4.15) |
| Taiqian | Henan | -2.96 (-5.45, -0.40) | -8.81 (-16.09, -0.89) | 4.96 (-20.1, 37.88) | -10.25 (-19.73, 0.35) |
| Tanghe | Henan | -8.03 (-10.48, -5.51) | 5.14 (0.25, 10.27) | -11.94 (-24.04, 2.09) | -8.22 (-18.96, 3.95) |
| Tangyin | Henan | -2.26 (-3.72, -0.77) | -2.40 (-8.69, 4.31) | 0.97 (-7.83, 10.61) | -7.62 (-22.92, 10.71) |
| Tongbai | Henan | -3.18 (-4.71, -1.63) | -3.26 (-21.48, 19.17) | -1.91 (-4.59, 0.84) | 0.85 (0.13, 1.57) |
| Tongxu | Henan | -5.23 (-6.43, -4.02) | -9.66 (-19.13, 0.91) | 0.28 (-4.52, 5.32) | -8.05 (-9.29, -6.80) |
| Weihui | Henan | -1.19 (-3.10, 0.77) | -4.41 (-19.13, 13.00) | 8.39 (-8.55, 28.45) | -5.16 (-10.92, 0.96) |
| Weishi | Henan | -3.71 (-5.05, -2.35) | -0.42 (-12.85, 13.79) | -0.31 (-11.51, 12.30) | -5.94 (-14.22, 3.13) |
| Wen | Henan | -4.12 (-5.13, -3.11) | -7.14 (-18.63, 5.98) | -1.25 (-7.27, 5.16) | -3.58 (-6.14, -0.95) |
| Wugang | Henan | -1.88 (-4.50, 0.82) | -15.56 (-26.32, -3.24) | 8.49 (-0.01, 17.70) | -10.62 (-21.73, 2.08) |
| Wuyang | Henan | -7.61 (-10.02, -5.13) | -10.94 (-20.48, -0.26) | -6.00 (-20.47, 11.10) | -6.85 (-21.01, 9.85) |
| Wuzhi | Henan | -3.09 (-4.89, -1.26) | -3.48 (-21.43, 18.58) | -0.06 (-13.22, 15.09) | -3.00 (-8.97, 3.37) |
| Xi | Henan | -3.14 (-4.69, -1.55) | -9.24 (-23.48, 7.65) | 1.61 (-4.80, 8.46) | -7.10 (-14.33, 0.73) |
| Xiangcheng | Henan | -5.21 (-7.02, -3.36) | -11.16 (-24.84, 5.02) | 3.95 (-2.18, 10.47) | -9.45 (-20.02, 2.51) |
| Xiangcheng | Henan | -5.49 (-7.47, -3.48) | -15.42 (-29.37, 1.27) | -1.29 (-6.74, 4.49) | -0.22 (-6.33, 6.29) |
| Xiayi | Henan | -1.41 (-2.64, -0.15) | -3.13 (-12.99, 7.84) | -0.01 (-10.39, 11.58) | 4.54 (-1.98, 11.50) |
| Xichuan | Henan | -5.94 (-7.80, -4.03) | -6.75 (-23.57, 13.78) | -5.67 (-17.25, 7.54) | 2.45 (-3.91, 9.22) |
| Xihua | Henan | -4.95 (-6.52, -3.34) | -10.99 (-24.34, 4.71) | -5.08 (-9.90, 0.01) | 0.95 (-5.20, 7.49) |
| Xin | Henan | -9.57 (-11.86, -7.23) | -14.54 (-33.12, 9.20) | -7.24 (-18.67, 5.79) | -11.04 (-18.95, -2.36) |
| Xinan | Henan | -4.56 (-6.22, -2.87) | -14.83 (-24.39, -4.06) | -4.11 (-13.89, 6.80) | -2.21 (-7.57, 3.47) |
| Xincai | Henan | -4.74 (-6.39, -3.07) | -6.88 (-26.44, 17.87) | -8.32 (-14.90, -1.23) | -8.15 (-14.04, -1.86) |
| Xingyang | Henan | -4.42 (-7.48, -1.26) | -7.25 (-22.85, 11.50) | 6.49 (-10.92, 27.29) | -21.11 (-35.97, -2.80) |
| Xinmi | Henan | -3.21 (-4.44, -1.96) | -6.17 (-17.95, 7.29) | 0.55 (-6.32, 7.91) | -6.66 (-13.04, 0.19) |
| Xinxiang | Henan | -0.93 (-3.14, 1.32) | -1.09 (-12.21, 11.45) | -8.15 (-27.50, 16.37) | -9.27 (-14.09, -4.19) |
| Xinye | Henan | -2.16 (-3.33, -0.97) | -2.44 (-13.19, 9.63) | -0.29 (-9.23, 9.52) | -3.11 (-13.98, 9.13) |
| Xinzheng | Henan | -6.49 (-8.77, -4.15) | -7.57 (-15.70, 1.35) | -1.25 (-18.24, 19.28) | -1.11 (-9.24, 7.74) |
| Xiping | Henan | -4.16 (-5.33, -2.97) | -7.67 (-20.66, 7.45) | -2.24 (-9.10, 5.15) | -3.11 (-6.73, 0.66) |
| Xiuwu | Henan | -2.02 (-3.48, -0.54) | -5.52 (-15.47, 5.59) | 4.63 (-7.11, 17.86) | -2.75 (-7.05, 1.74) |
| Xixia | Henan | -3.42 (-4.43, -2.41) | -2.84 (-8.96, 3.68) | -6.26 (-14.20, 2.41) | -0.07 (-4.75, 4.84) |
| Yanjin | Henan | -2.68 (-4.96, -0.35) | -9.71 (-21.09, 3.30) | 10.74 (-6.07, 30.56) | -8.01 (-12.85, -2.91) |
| Yanling | Henan | -3.45 (-6.03, -0.80) | -16.55 (-24.68, -7.54) | 13.63 (-0.34, 29.56) | -6.07 (-9.30, -2.72) |
| Yanshi | Henan | 0.34 (-0.55, 1.24) | 1.45 (-4.05, 7.26) | 4.90 (1.24, 8.68) | 2.81 (-3.85, 9.94) |
| Ye | Henan | -5.95 (-7.47, -4.40) | -12.77 (-16.93, -8.41) | 1.70 (-2.72, 6.33) | -5.20 (-15.79, 6.73) |
| Yichuan | Henan | -4.88 (-6.10, -3.64) | -3.42 (-9.03, 2.53) | 0.13 (-4.48, 4.95) | -1.68 (-5.95, 2.79) |
| Yima | Henan | -7.50 (-10.53, -4.37) | -14.53 (-26.56, -0.53) | 5.98 (-11.10, 26.35) | -0.09 (-13.3, 15.14) |
| Yiyang | Henan | -4.98 (-6.43, -3.50) | -10.66 (-18.23, -2.39) | -1.06 (-9.18, 7.78) | -0.54 (-5.35, 4.51) |
| Yongcheng | Henan | -8.54 (-10.15, -6.89) | -9.04 (-16.04, -1.45) | -2.70 (-5.72, 0.43) | 3.58 (-4.05, 11.81) |
| Yuanyang | Henan | -2.76 (-4.16, -1.33) | -5.37 (-13.20, 3.17) | 1.18 (-9.60, 13.24) | -5.74 (-16.94, 6.97) |
| Yucheng | Henan | -0.81 (-2.50, 0.90) | -5.13 (-17.94, 9.67) | 7.03 (2.34, 11.93) | -0.21 (-13.20, 14.72) |
| Yuzhou | Henan | -1.98 (-3.08, -0.87) | -3.97 (-17.68, 12.02) | -0.22 (-5.88, 5.79) | -3.20 (-7.80, 1.62) |
| Zhecheng | Henan | -3.59 (-4.80, -2.36) | -5.04 (-20.70, 13.72) | -0.86 (-4.13, 2.52) | -5.56 (-10.59, -0.24) |
| Zhengyang | Henan | -5.44 (-7.02, -3.84) | -13.61 (-25.80, 0.58) | -1.38 (-4.94, 2.31) | -4.86 (-9.14, -0.38) |
| Zhenping | Henan | -4.00 (-5.26, -2.73) | -11.33 (-20.09, -1.62) | -1.27 (-4.63, 2.21) | -1.98 (-4.22, 0.32) |
| Zhongmou | Henan | -6.59 (-8.35, -4.80) | -0.63 (-18.34, 20.92) | -6.60 (-14.95, 2.58) | -12.62 (-16.37, -8.70) |
| Anlu | Hubei | -4.09 (-6.34, -1.79) | 4.82 (-5.78, 16.61) | -3.47 (-13.57, 7.81) | -14.91 (-27.30, -0.43) |
| Badong | Hubei | -0.01 (-1.53, 1.54) | -1.45 (-9.40, 7.20) | 0.01 (-12.20, 13.92) | -9.52 (-13.12, -5.78) |
| Baokang | Hubei | -5.22 (-6.94, -3.47) | -7.70 (-23.08, 10.75) | -0.78 (-8.58, 7.69) | -7.89 (-17.72, 3.13) |
| Changyang | Hubei | -1.00 (-4.53, 2.66) | -3.88 (-22.97, 19.95) | 3.63 (-11.18, 20.9) | 0.11 (-2.45, 2.75) |
| Chibi | Hubei | -4.38 (-5.06, -3.70) | -4.37 (-11.02, 2.77) | -1.93 (-7.97, 4.51) | -5.22 (-7.82, -2.55) |
| Chongyang | Hubei | -1.17 (-2.44, 0.13) | -2.37 (-8.70, 4.39) | 0.62 (-3.58, 5.01) | -10.02 (-16.47, -3.08) |
| Dangyang | Hubei | -5.49 (-7.30, -3.65) | -2.39 (-12.76, 9.22) | -1.81 (-13.73, 11.77) | -4.92 (-17.99, 10.23) |
| Danjiangkou | Hubei | -5.68 (-6.83, -4.52) | -7.46 (-16.05, 2.02) | 0.57 (-7.80, 9.69) | -9.84 (-12.07, -7.55) |
| Dawu | Hubei | -5.55 (-7.12, -3.96) | -2.54 (-15.02, 11.77) | -3.50 (-13.57, 7.74) | -12.23 (-21.54, -1.82) |
| Daye | Hubei | -5.68 (-6.69, -4.66) | -8.42 (-15.01, -1.31) | -3.04 (-6.90, 0.97) | -5.14 (-14.99, 5.85) |
| Enshi | Hubei | -1.15 (-3.53, 1.28) | -8.62 (-12.58, -4.49) | -0.20 (-10.82, 11.69) | 11.88 (-5.59, 32.58) |
| Fang | Hubei | -4.93 (-6.80, -3.02) | -0.19 (-11.21, 12.21) | 0.20 (-7.41, 8.44) | -15.26 (-25.58, -3.52) |
| Gongan | Hubei | -3.67 (-4.78, -2.54) | -2.19 (-13.86, 11.05) | 1.08 (-1.13, 3.34) | -7.87 (-8.47, -7.26) |
| Guangshui | Hubei | -4.83 (-6.52, -3.12) | -5.24 (-9.72, -0.54) | -3.61 (-11.47, 4.96) | -12.31 (-28.30, 7.24) |
| Gucheng | Hubei | -5.14 (-6.87, -3.39) | -8.23 (-20.69, 6.19) | 0.56 (-5.38, 6.87) | -0.71 (-6.97, 5.97) |
| Hanchuan | Hubei | -9.62 (-11.77, -7.43) | -0.14 (-15.59, 18.14) | -17.87 (-31.91, -0.93) | -4.23 (-13.96, 6.59) |
| Hefeng | Hubei | -1.17 (-3.62, 1.35) | 4.50 (2.55, 6.49) | -6.49 (-17.17, 5.56) | -13.86 (-31.74, 8.72) |
| Hongan | Hubei | -4.16 (-5.95, -2.33) | 3.98 (-11.99, 22.83) | 0.02 (-6.70, 7.22) | -11.18 (-16.69, -5.31) |
| Honghu | Hubei | -2.84 (-5.46, -0.14) | 1.63 (-16.45, 23.61) | 2.35 (0.56, 4.17) | -13.82 (-31.83, 8.94) |
| Huangmei | Hubei | -6.83 (-9.21, -4.40) | -5.35 (-20.74, 13.03) | -0.42 (-5.97, 5.46) | -19.81 (-34.06, -2.50) |
| Jiangling | Hubei | -4.64 (-6.64, -2.60) | -11.67 (-29.13, 10.09) | 1.28 (-4.48, 7.39) | -10.81 (-18.50, -2.40) |
| Jianli | Hubei | -1.94 (-6.05, 2.35) | -15.88 (-45.04, 28.74) | -1.74 (-30.20, 38.30) | -7.99 (-24.14, 11.59) |
| Jianshi | Hubei | -2.98 (-4.79, -1.14) | 0.68 (-9.55, 12.06) | -4.75 (-18.56, 11.40) | 6.50 (-6.83, 21.75) |
| Jiayu | Hubei | -1.92 (-3.95, 0.15) | -2.27 (-28.05, 32.74) | 0.24 (-3.27, 3.88) | 0.31 (-8.35, 9.79) |
| Jingshan | Hubei | -4.02 (-5.61, -2.41) | -1.64 (-8.40, 5.61) | 0.59 (-1.93, 3.17) | -14.20 (-22.01, -5.61) |
| Laifeng | Hubei | 1.71 (-0.28, 3.73) | 2.99 (-13.89, 23.18) | 5.05 (-8.24, 20.26) | -3.26 (-10.32, 4.37) |
| Laohekou | Hubei | -3.26 (-4.74, -1.75) | -5.31 (-21.98, 14.91) | -0.24 (-3.71, 3.36) | -0.54 (-11.01, 11.17) |
| Lichuan | Hubei | -2.07 (-3.68, -0.43) | 4.64 (-5.20, 15.50) | -0.18 (-10.49, 11.33) | -10.15 (-18.54, -0.9) |
| Luotian | Hubei | -4.00 (-5.14, -2.84) | -3.12 (-13.70, 8.75) | -2.43 (-5.09, 0.30) | -6.41 (-14.06, 1.91) |
| Macheng | Hubei | -4.29 (-6.11, -2.44) | -9.13 (-21.68, 5.42) | 5.16 (-10.20, 23.15) | -7.74 (-12.40, -2.83) |
| Nanzhang | Hubei | -5.69 (-6.96, -4.41) | -8.84 (-21.06, 5.28) | -2.19 (-8.55, 4.61) | -7.56 (-13.35, -1.38) |
| Qianjiang | Hubei | -5.01 (-6.08, -3.94) | -0.68 (-10.60, 10.34) | -4.65 (-7.92, -1.26) | -10.16 (-13.41, -6.79) |
| Qichun | Hubei | -1.37 (-2.93, 0.21) | -9.05 (-13.96, -3.87) | 5.67 (-3.23, 15.37) | -5.73 (-14.66, 4.13) |
| Shayang | Hubei | -5.65 (-7.23, -4.05) | -7.62 (-21.16, 8.24) | 2.67 (-4.02, 9.83) | -10.11 (-15.07, -4.87) |
| Shennongjia | Hubei | -1.71 (-2.99, -0.42) | -0.46 (-8.44, 8.21) | -1.11 (-4.42, 2.31) | 0.27 (-15.95, 19.61) |
| Shishou | Hubei | -3.75 (-5.69, -1.78) | -12.54 (-25.53, 2.72) | -0.38 (-5.28, 4.77) | -9.35 (-23.20, 6.99) |
| Songzi | Hubei | -2.20 (-3.67, -0.70) | 2.03 (-17.65, 26.41) | -2.14 (-8.80, 5.01) | -3.52 (-7.94, 1.10) |
| Tianmen | Hubei | -3.67 (-4.97, -2.34) | -2.04 (-15.45, 13.51) | 0.51 (-8.36, 10.24) | -8.61 (-11.48, -5.64) |
| Tongcheng | Hubei | -1.09 (-2.66, 0.50) | -7.84 (-14.22, -0.98) | -0.83 (-6.26, 4.92) | -8.77 (-13.37, -3.94) |
| Tongshan | Hubei | -2.32 (-4.32, -0.27) | -5.48 (-14.11, 4.02) | 5.09 (-2.79, 13.62) | -10.62 (-20.62, 0.64) |
| Tuanfeng | Hubei | -3.53 (-5.49, -1.52) | -7.58 (-15.99, 1.66) | 3.50 (-5.03, 12.79) | -15.18 (-22.03, -7.73) |
| Wufeng | Hubei | -0.65 (-2.66, 1.41) | 5.21 (-10.11, 23.13) | -4.73 (-13.89, 5.42) | 7.39 (1.07, 14.11) |
| Wuxue | Hubei | -4.57 (-5.74, -3.39) | -5.99 (-18.14, 7.97) | -0.06 (-5.53, 5.73) | -7.29 (-11.81, -2.55) |
| Xianfeng | Hubei | -3.23 (-4.86, -1.56) | -1.43 (-16.62, 16.51) | 2.61 (-11.16, 18.51) | -5.48 (-15.22, 5.38) |
| Xiantao | Hubei | -2.48 (-3.84, -1.11) | 0.47 (-9.98, 12.13) | -0.11 (-10.82, 11.89) | -8.13 (-10.07, -6.14) |
| Xiaochang | Hubei | -5.66 (-6.90, -4.41) | -2.47 (-7.90, 3.28) | -3.99 (-10.96, 3.52) | -7.81 (-13.18, -2.11) |
| Xingshan | Hubei | -5.13 (-6.99, -3.22) | -11.89 (-30.56, 11.80) | -4.21 (-13.34, 5.89) | -4.22 (-15.81, 8.97) |
| Xishui | Hubei | -3.77 (-5.05, -2.48) | -6.57 (-17.13, 5.35) | -0.97 (-7.00, 5.45) | -8.47 (-18.12, 2.31) |
| Xuanen | Hubei | 0.43 (-1.94, 2.85) | 6.09 (-11.15, 26.68) | -2.64 (-19.03, 17.07) | 9.17 (-6.22, 27.09) |
| Yangxin | Hubei | -2.64 (-4.27, -0.98) | -8.02 (-19.56, 5.17) | 1.34 (-8.11, 11.75) | -7.56 (-12.86, -1.94) |
| Yicheng | Hubei | -3.96 (-5.20, -2.69) | -4.82 (-12.37, 3.39) | -0.64 (-11.22, 11.21) | -9.92 (-16.73, -2.55) |
| Yidu | Hubei | -5.31 (-6.88, -3.71) | -12.35 (-25.54, 3.18) | -4.42 (-9.83, 1.31) | -2.50 (-10.73, 6.48) |
| Yingcheng | Hubei | -7.41 (-8.72, -6.09) | -15.01 (-18.32, -11.57) | -7.31 (-18.88, 5.90) | -6.25 (-10.69, -1.59) |
| Yingshan | Hubei | -2.37 (-4.27, -0.44) | 4.84 (-3.13, 13.47) | -2.99 (-7.35, 1.57) | -12.33 (-26.51, 4.59) |
| Yuanan | Hubei | -3.61 (-5.22, -1.97) | -2.11 (-19.29, 18.72) | 2.53 (-3.11, 8.50) | -7.41 (-15.69, 1.69) |
| Yunmeng | Hubei | -4.50 (-6.17, -2.81) | -1.03 (-5.30, 3.43) | -0.38 (-8.44, 8.39) | -11.08 (-18.32, -3.19) |
| Yunxi | Hubei | -1.71 (-3.06, -0.34) | 0.11 (-18.53, 23.02) | -0.37 (-5.57, 5.12) | -0.78 (-4.44, 3.02) |
| Zaoyang | Hubei | -6.68 (-8.81, -4.51) | -4.66 (-28.00, 26.24) | -2.12 (-5.67, 1.57) | -11.90 (-24.11, 2.28) |
| Zhijiang | Hubei | -5.84 (-7.95, -3.68) | -15.20 (-22.98, -6.64) | -2.98 (-18.52, 15.53) | 3.32 (-3.00, 10.06) |
| Zhongxiang | Hubei | -4.48 (-6.43, -2.49) | -2.66 (-8.60, 3.67) | -1.53 (-4.69, 1.74) | -14.46 (-25.72, -1.49) |
| Zhushan | Hubei | -0.57 (-1.98, 0.87) | -1.44 (-16.16, 15.87) | -0.51 (-7.88, 7.46) | -4.65 (-15.86, 8.06) |
| Zhuxi | Hubei | -4.31 (-5.66, -2.95) | 0.70 (-15.20, 19.57) | -5.47 (-11.19, 0.61) | -3.77 (-12.18, 5.44) |
| Zigui | Hubei | -3.26 (-5.77, -0.68) | 1.82 (-28.98, 45.98) | 0.50 (-9.02, 11.02) | -3.95 (-5.92, -1.94) |
| Anhua | Hunan | 0.06 (-2.14, 2.30) | -13.59 (-27.33, 2.74) | 4.88 (-1.39, 11.56) | 6.00 (-0.26, 12.67) |
| Anren | Hunan | 0.67 (-1.07, 2.43) | -5.96 (-16.21, 5.55) | 3.06 (-9.27, 17.07) | 2.98 (-5.81, 12.58) |
| Anxiang | Hunan | -2.05 (-3.53, -0.54) | 1.63 (-8.64, 13.05) | 6.30 (-3.60, 17.22) | -6.64 (-12.66, -0.21) |
| Baojing | Hunan | -3.65 (-5.21, -2.05) | -9.32 (-18.55, 0.95) | -4.22 (-16.35, 9.68) | 3.70 (-6.15, 14.59) |
| Chaling | Hunan | -2.10 (-3.75, -0.43) | -4.48 (-11.99, 3.68) | -0.36 (-7.20, 6.98) | 8.86 (-0.93, 19.63) |
| Changning | Hunan | 0.56 (-0.74, 1.87) | 1.01 (-9.52, 12.76) | 6.09 (1.96, 10.39) | -3.34 (-14.38, 9.12) |
| Changsha | Hunan | -3.82 (-5.32, -2.30) | -0.64 (-9.90, 9.57) | -9.76 (-15.90, -3.18) | 1.58 (-11.82, 17.02) |
| Chengbu | Hunan | -0.08 (-1.74, 1.61) | -4.18 (-16.13, 9.47) | 4.70 (3.08, 6.35) | -3.76 (-7.10, -0.29) |
| Chenxi | Hunan | -3.64 (-5.19, -2.06) | -7.30 (-19.44, 6.66) | 4.42 (-0.80, 9.91) | -1.29 (-13.25, 12.31) |
| Cili | Hunan | -1.95 (-3.12, -0.76) | -6.54 (-16.40, 4.49) | 1.03 (-3.79, 6.10) | 2.45 (-3.34, 8.60) |
| Dao | Hunan | -1.23 (-2.89, 0.47) | -7.78 (-18.11, 3.86) | -2.39 (-12.69, 9.13) | 0.73 (-8.36, 10.71) |
| Dongan | Hunan | -2.63 (-3.25, -2.00) | -1.42 (-5.79, 3.15) | -3.08 (-7.41, 1.46) | -3.12 (-9.98, 4.26) |
| Dongkou | Hunan | -0.78 (-2.90, 1.38) | -1.03 (-28.03, 36.10) | 2.23 (-5.75, 10.88) | 3.52 (-3.41, 10.94) |
| Fenghuang | Hunan | -0.41 (-1.53, 0.72) | 1.57 (-7.15, 11.12) | 3.22 (-4.16, 11.15) | 3.49 (-3.04, 10.47) |
| Guidong | Hunan | -5.34 (-7.93, -2.68) | -5.30 (-18.54, 10.09) | 1.00 (-9.87, 13.18) | 1.05 (-7.89, 10.86) |
| Guiyang | Hunan | 0.86 (-0.3, 2.04) | -2.61 (-16.90, 14.13) | 1.89 (-1.37, 5.26) | 1.95 (-4.13, 8.41) |
| Guzhang | Hunan | -8.54 (-15.39, -1.13) | 0.77 (-16.11, 21.03) | -6.25 (-25.14, 17.41) | 63.46 (25.17, 113.47) |
| Hanshou | Hunan | 1.38 (-1.14, 3.96) | 5.62 (-12.24, 27.13) | 4.72 (-5.79, 16.39) | 8.73 (-12.61, 35.28) |
| Hengdong | Hunan | 3.25 (1.77, 4.76) | 3.43 (-7.97, 16.24) | 6.03 (-6.27, 19.94) | 9.86 (-0.22, 20.96) |
| Hengshan | Hunan | 3.58 (1.02, 6.20) | 10.27 (-3.06, 25.42) | -6.56 (-23.98, 14.84) | 1.66 (-9.25, 13.88) |
| Hengyang | Hunan | -1.72 (-3.57, 0.17) | 5.07 (-3.29, 14.16) | -6.77 (-20.01, 8.67) | 2.37 (-10.52, 17.11) |
| Hongjiang | Hunan | -1.03 (-2.90, 0.88) | -9.80 (-17.17, -1.76) | 5.65 (-8.30, 21.73) | 1.47 (-11.78, 16.71) |
| Huarong | Hunan | -3.25 (-4.61, -1.87) | 1.38 (-12.28, 17.16) | 0.09 (-1.49, 1.70) | -8.21 (-17.38, 1.99) |
| Huayuan | Hunan | -4.47 (-6.43, -2.47) | 4.85 (-6.34, 17.38) | -2.20 (-14.7, 12.13) | 1.82 (-13.81, 20.28) |
| Huitong | Hunan | 0.01 (-2.43, 2.52) | -7.24 (-28.51, 20.35) | 7.51 (-7.75, 25.30) | -3.92 (-9.23, 1.71) |
| Jiahe | Hunan | -1.74 (-3.66, 0.22) | -0.60 (-11.85, 12.09) | 7.84 (0.97, 15.17) | -9.11 (-19.95, 3.19) |
| Jianghua | Hunan | -0.78 (-2.14, 0.60) | -0.60 (-12.93, 13.48) | -0.31 (-6.90, 6.75) | -1.75 (-8.73, 5.77) |
| Jiangyong | Hunan | -1.91 (-3.53, -0.26) | -1.68 (-22.19, 24.24) | -4.49 (-14.69, 6.92) | -0.32 (-5.14, 4.75) |
| Jin | Hunan | -1.69 (-3.69, 0.35) | 5.57 (-12.50, 27.38) | 0.59 (-11.92, 14.87) | 6.08 (-6.26, 20.06) |
| Jingzhou | Hunan | -1.66 (-4.14, 0.88) | -0.70 (-23.10, 28.22) | 5.67 (-3.82, 16.10) | -5.74 (-26.51, 20.89) |
| Jishou | Hunan | -3.33 (-5.45, -1.17) | -5.35 (-17.24, 8.25) | -6.27 (-8.76, -3.70) | 8.15 (-13.98, 35.98) |
| Lanshan | Hunan | 0.09 (-3.99, 4.33) | 23.77 (0.75, 52.05) | 4.33 (-7.04, 17.10) | -16.33 (-21.08, -11.29) |
| Leiyang | Hunan | -0.71 (-2.55, 1.16) | -6.55 (-17.14, 5.39) | 5.72 (4.25, 7.22) | 6.70 (-9.44, 25.71) |
| Lengshuijiang | Hunan | -6.60 (-8.34, -4.82) | -1.11 (-3.60, 1.43) | -4.94 (-19.26, 11.92) | -9.29 (-18.87, 1.41) |
| Li | Hunan | -1.61 (-2.81, -0.38) | -1.98 (-12.64, 9.98) | -1.71 (-5.43, 2.16) | 3.89 (-6.35, 15.25) |
| Lianyuan | Hunan | -0.82 (-2.27, 0.64) | -0.30 (-16.50, 19.05) | 3.36 (-4.42, 11.77) | 0.85 (-3.28, 5.16) |
| Liling | Hunan | -0.63 (-1.92, 0.68) | -3.69 (-12.31, 5.77) | -7.31 (-14.32, 0.27) | -1.16 (-9.85, 8.36) |
| Linli | Hunan | -3.58 (-6.17, -0.92) | -13.39 (-19.95, -6.29) | -4.99 (-13.15, 3.93) | 11.85 (-1.43, 26.92) |
| Linwu | Hunan | -1.04 (-3.04, 0.99) | 0.96 (-18.13, 24.50) | -6.09 (-16.32, 5.39) | -2.19 (-9.92, 6.20) |
| Linxiang | Hunan | -4.31 (-6.13, -2.45) | 4.69 (-17.09, 32.21) | -4.07 (-11.62, 4.14) | -7.70 (-13.42, -1.61) |
| Liuyang | Hunan | -4.30 (-6.29, -2.27) | -6.43 (-23.20, 14.01) | -3.17 (-14.73, 9.95) | -2.92 (-13.56, 9.02) |
| Longhui | Hunan | -1.83 (-3.47, -0.15) | -8.85 (-19.57, 3.29) | -2.40 (-12.18, 8.48) | 6.20 (-3.02, 16.29) |
| Longshan | Hunan | -2.18 (-4.25, -0.07) | 0.06 (-11.04, 12.55) | -5.22 (-10.25, 0.09) | 5.48 (-15.02, 30.94) |
| Lukou | Hunan | -0.42 (-2.62, 1.83) | -0.88 (-18.52, 20.59) | -3.27 (-12.58, 7.04) | 7.81 (-7.76, 26.01) |
| Luxi | Hunan | -0.52 (-2.14, 1.12) | 0.11 (-16.49, 20.02) | -1.85 (-6.21, 2.71) | 8.82 (2.18, 15.90) |
| Mayang | Hunan | -3.43 (-5.54, -1.28) | -4.55 (-21.15, 15.53) | -7.89 (-19.39, 5.24) | -0.37 (-14.13, 15.59) |
| Miluo | Hunan | -3.32 (-5.32, -1.29) | 3.33 (-17.72, 29.77) | 0.27 (-2.37, 2.97) | -0.41 (-17.80, 20.66) |
| Nan | Hunan | 0.87 (-1.59, 3.39) | 7.44 (-10.74, 29.34) | -0.59 (-6.07, 5.21) | 5.06 (-14.94, 29.76) |
| Ningxiang | Hunan | -0.26 (-2.40, 1.92) | 3.76 (-13.44, 24.38) | 2.61 (-2.96, 8.50) | -7.08 (-15.01, 1.59) |
| Ningyuan | Hunan | -0.50 (-3.12, 2.20) | 18.08 (-5.22, 47.11) | 4.42 (-3.52, 13.01) | 1.63 (-10.29, 15.14) |
| Pingjiang | Hunan | -3.38 (-4.90, -1.84) | -1.43 (-14.54, 13.69) | 3.48 (-2.76, 10.11) | -9.12 (-17.43, 0.02) |
| Qidong | Hunan | 0.27 (-2.83, 3.47) | 2.45 (-18.22, 28.33) | 6.61 (-1.04, 14.84) | 12.04 (-0.17, 25.75) |
| Qiyang | Hunan | 0.94 (-0.58, 2.49) | 4.79 (-6.41, 17.32) | -1.88 (-12.30, 9.77) | 7.03 (-4.48, 19.93) |
| Rucheng | Hunan | 0.87 (-0.56, 2.32) | -5.14 (-15.64, 6.66) | 6.70 (-2.20, 16.42) | -1.69 (-8.88, 6.06) |
| Sangzhi | Hunan | -1.89 (-4.22, 0.50) | -4.30 (-10.83, 2.71) | -2.17 (-5.73, 1.52) | 9.20 (2.90, 15.88) |
| Shaodong | Hunan | 0.63 (-0.50, 1.78) | 1.19 (-6.69, 9.73) | 0.12 (-9.14, 10.33) | -0.39 (-8.93, 8.94) |
| Shaoshan | Hunan | 2.05 (-1.14, 5.34) | 2.19 (-10.13, 16.20) | 7.78 (2.46, 13.38) | -19.18 (-34.99, 0.47) |
| Shaoyang | Hunan | -0.30 (-1.91, 1.33) | -4.90 (-16.89, 8.82) | 5.39 (-4.28, 16.03) | -5.85 (-14.35, 3.51) |
| Shimen | Hunan | 1.36 (-0.17, 2.91) | 2.84 (-5.80, 12.28) | -2.82 (-11.25, 6.40) | 7.43 (-7.06, 24.19) |
| Shuangfeng | Hunan | 1.18 (-0.36, 2.74) | -3.16 (-13.47, 8.37) | 2.84 (-3.43, 9.51) | 0.67 (-13.60, 17.31) |
| Shuangpai | Hunan | -3.57 (-5.40, -1.71) | 2.26 (-19.15, 29.36) | -5.81 (-14.61, 3.90) | -0.60 (-11.78, 11.99) |
| Suining | Hunan | -0.25 (-2.13, 1.66) | -3.46 (-19.01, 15.07) | 3.32 (-0.05, 6.81) | -6.14 (-16.02, 4.90) |
| Taojiang | Hunan | -4.78 (-6.40, -3.12) | -11.56 (-20.93, -1.08) | 3.74 (-1.78, 9.58) | -4.43 (-15.49, 8.08) |
| Taoyuan | Hunan | -1.99 (-4.08, 0.14) | -3.81 (-11.80, 4.89) | -2.44 (-9.10, 4.70) | -6.00 (-12.89, 1.43) |
| Tongdao | Hunan | -0.93 (-3.10, 1.29) | -0.62 (-17.20, 19.28) | 0.81 (-9.37, 12.13) | -7.34 (-25.08, 14.61) |
| Wangcheng | Hunan | -5.16 (-6.32, -4.00) | 0.41 (-0.98, 1.81) | -11.54 (-16.6, -6.18) | -1.90 (-7.67, 4.24) |
| Wugang | Hunan | -2.19 (-3.92, -0.42) | -4.89 (-14.62, 5.94) | 0.01 (-2.82, 2.93) | 6.03 (-9.35, 24.02) |
| Xiangtan | Hunan | 0.01 (-1.70, 1.75) | -2.00 (-15.69, 13.92) | 2.65 (-9.63, 16.61) | 6.83 (-6.21, 21.67) |
| Xiangxiang | Hunan | -0.90 (-2.49, 0.72) | -1.16 (-17.61, 18.59) | -1.82 (-4.70, 1.16) | -4.51 (-16.10, 8.68) |
| Xiangyin | Hunan | -4.37 (-6.11, -2.59) | 2.06 (-13.04, 19.79) | -2.53 (-10.81, 6.53) | -2.56 (-13.11, 9.28) |
| Xinhua | Hunan | 0.89 (-2.13, 4.00) | -8.83 (-29.19, 17.39) | 17.31 (2.82, 33.83) | -5.39 (-19.10, 10.64) |
| Xinhuang | Hunan | -5.80 (-8.32, -3.21) | -9.30 (-29.37, 16.49) | 1.71 (-4.30, 8.10) | 4.24 (-3.35, 12.42) |
| Xinning | Hunan | -1.41 (-4.18, 1.45) | -15.8 (-29.73, 0.90) | 10.42 (6.94, 14.02) | 7.35 (-9.46, 27.29) |
| Xinshao | Hunan | -0.99 (-2.45, 0.48) | 0.96 (-15.33, 20.37) | 1.70 (-1.72, 5.23) | -1.73 (-14.49, 12.93) |
| Xintian | Hunan | 1.43 (-1.21, 4.13) | -1.15 (-23.96, 28.51) | 0.34 (-10.59, 12.61) | -0.93 (-19.87, 22.50) |
| Xupu | Hunan | -1.50 (-3.56, 0.60) | 0.53 (-7.56, 9.33) | 5.55 (-12.93, 27.95) | 0.48 (-16.31, 20.63) |
| Yanling | Hunan | -5.74 (-9.03, -2.33) | -10.65 (-33.83, 20.66) | -21.43 (-35.51, -4.29) | 0.89 (-5.67, 7.91) |
| Yizhang | Hunan | -0.18 (-1.69, 1.35) | 2.21 (-7.56, 13.02) | 0.06 (-6.74, 7.36) | -5.55 (-8.61, -2.38) |
| Yongshun | Hunan | -0.20 (-3.69, 3.41) | -9.34 (-23.82, 7.87) | 5.68 (-7.59, 20.85) | 20.75 (-8.21, 58.86) |
| Yongxing | Hunan | -2.82 (-4.58, -1.02) | -3.83 (-18.39, 13.32) | -0.16 (-8.21, 8.61) | 0.98 (-16.33, 21.87) |
| You | Hunan | -1.65 (-3.54, 0.29) | 2.67 (-3.89, 9.67) | -5.95 (-15.65, 4.86) | 12.41 (5.80, 19.45) |
| Yuanjiang | Hunan | 0.47 (-1.88, 2.88) | -11.19 (-30.18, 12.95) | 4.93 (-0.96, 11.16) | -2.72 (-8.11, 2.98) |
| Yuanling | Hunan | -2.45 (-4.50, -0.36) | -2.95 (-15.90, 12.01) | 6.06 (-7.34, 21.40) | -8.91 (-23.87, 9.00) |
| Yueyang | Hunan | -6.24 (-8.10, -4.35) | -0.41 (-5.67, 5.15) | 3.07 (-7.30, 14.61) | -11.75 (-25.45, 4.46) |
| Zhijiang | Hunan | 2.59 (-2.07, 7.48) | 4.72 (-45.32, 100.55) | 16.83 (11.31, 22.62) | -10.84 (-22.68, 2.81) |
| Zhongfang | Hunan | -3.15 (-6.19, -0.01) | -5.81 (-38.05, 43.20) | 5.16 (0.22, 10.34) | -13.00 (-25.33, 1.36) |
| Zixing | Hunan | -0.75 (-2.48, 1.00) | -4.99 (-12.67, 3.38) | 4.74 (-0.40, 10.15) | 7.45 (-7.12, 24.31) |
| Abaga Banner | Inner Mongolia | -1.42 (-4.05, 1.29) | -8.59 (-24.85, 11.19) | 14.72 (0.95, 30.38) | -6.47 (-13.30, 0.90) |
| Alxa Left Banner | Inner Mongolia | -5.41 (-7.98, -2.77) | 9.67 (-2.64, 23.55) | -13.41 (-20.80, -5.32) | 5.85 (-4.37, 17.15) |
| Alxa Right Banner | Inner Mongolia | 0.56 (-3.62, 4.93) | 6.13 (-16.47, 34.83) | -9.12 (-27.45, 13.85) | -3.20 (-30.54, 34.89) |
| Aohan Banner | Inner Mongolia | -3.02 (-6.00, 0.05) | 0.33 (-19.86, 25.60) | -12.08 (-28.61, 8.27) | 7.17 (-1.28, 16.36) |
| Ar Horqin Banner | Inner Mongolia | -4.37 (-7.45, -1.20) | 13.11 (-7.07, 37.68) | -5.99 (-9.65, -2.17) | -8.37 (-27.70, 16.14) |
| Arun Banner | Inner Mongolia | -1.03 (-3.26, 1.25) | 5.67 (-3.75, 16.00) | -9.54 (-27.81, 13.37) | 8.14 (-0.68, 17.75) |
| Arxan | Inner Mongolia | -9.71 (-15.71, -3.28) | 3.31 (-34.51, 62.97) | -23.94 (-51.42, 19.08) | -3.99 (-13.35, 6.39) |
| Bairin Left Banner | Inner Mongolia | -3.43 (-5.15, -1.67) | 1.82 (-4.85, 8.95) | -3.14 (-11.71, 6.25) | -11.19 (-22.18, 1.36) |
| Bairin Right Banner | Inner Mongolia | -2.91 (-4.99, -0.79) | -6.62 (-22.16, 12.03) | -3.53 (-18.41, 14.07) | 4.34 (-10.32, 21.39) |
| Bordered Yellow Banner | Inner Mongolia | -11.23 (-14.12, -8.24) | -13.59 (-19.69, -7.02) | -22.10 (-34.06, -7.97) | -7.07 (-36.76, 36.56) |
| Dalad Banner | Inner Mongolia | -5.93 (-7.31, -4.53) | -0.15 (-12.15, 13.50) | -9.44 (-16.74, -1.49) | -6.08 (-13.49, 1.95) |
| Darhan Muminggan United Banner | Inner Mongolia | -10.42 (-14.79, -5.83) | -5.64 (-12.73, 2.03) | -15.11 (-24.15, -4.99) | 21.05 (-4.43, 53.33) |
| Dengkou | Inner Mongolia | -3.90 (-7.60, -0.04) | -11.49 (-23.31, 2.15) | -19.79 (-32.82, -4.24) | 17.24 (4.74, 31.23) |
| Duolun | Inner Mongolia | -9.93 (-13.71, -5.97) | -5.62 (-31.83, 30.67) | -21.27 (-41.33, 5.66) | 6.94 (-19.60, 42.23) |
| East Ujimqin Banner | Inner Mongolia | -0.85 (-3.34, 1.71) | -9.32 (-17.17, -0.72) | -6.85 (-27.40, 19.52) | 7.02 (-9.91, 27.13) |
| Ejin Banner | Inner Mongolia | -4.93 (-7.76, -2.01) | -1.51 (-24.10, 27.81) | -11.08 (-39.18, 30.00) | -7.62 (-13.39, -1.48) |
| Ejin Horo Banner | Inner Mongolia | -9.70 (-11.91, -7.45) | -1.39 (-8.92, 6.76) | -5.15 (-29.76, 28.08) | -3.78 (-12.59, 5.91) |
| Erenhot | Inner Mongolia | -12.05 (-15.87, -8.06) | -9.16 (-18.23, 0.92) | -18.97 (-41.51, 12.25) | 13.70 (-17.29, 56.30) |
| Ergun | Inner Mongolia | -4.30 (-8.46, 0.04) | 0.25 (-13.11, 15.66) | -18.09 (-38.45, 8.99) | 25.18 (-5.30, 65.48) |
| Etuoke Banner | Inner Mongolia | -9.18 (-12.15, -6.11) | 5.39 (-12.47, 26.89) | -17.06 (-38.11, 11.14) | -9.80 (-29.95, 16.16) |
| Etuokeqian Banner | Inner Mongolia | -9.89 (-12.64, -7.06) | -8.49 (-32.41, 23.88) | -7.64 (-17.09, 2.88) | -18.50 (-41.52, 13.57) |
| Evenk Autonomous Banner | Inner Mongolia | -1.95 (-4.11, 0.26) | -4.58 (-11.05, 2.35) | -7.86 (-15.15, 0.05) | 11.34 (3.35, 19.93) |
| Fengzhen | Inner Mongolia | -2.02 (-4.13, 0.14) | -6.69 (-17.57, 5.62) | -6.59 (-27.65, 20.60) | -1.54 (-16.45, 16.02) |
| Genhe | Inner Mongolia | -8.28 (-11.21, -5.26) | -3.08 (-20.00, 17.41) | -14.51 (-36.66, 15.38) | -1.78 (-26.35, 30.99) |
| Guyang | Inner Mongolia | -8.49 (-14.20, -2.40) | 2.36 (-6.57, 12.14) | -16.06 (-23.44, -7.97) | 38.66 (-1.15, 94.50) |
| Hangjin Banner | Inner Mongolia | -5.33 (-7.08, -3.55) | 0.95 (-15.33, 20.37) | -3.26 (-10.07, 4.07) | -13.09 (-21.60, -3.65) |
| Hangjin Rear Banner | Inner Mongolia | -3.49 (-5.09, -1.86) | -7.70 (-17.31, 3.02) | -4.44 (-14.30, 6.56) | 6.04 (-0.89, 13.44) |
| Harqin Banner | Inner Mongolia | -3.60 (-5.46, -1.70) | 1.89 (-6.28, 10.77) | 3.57 (-14.05, 24.80) | -9.78 (-20.21, 2.00) |
| Hexigten Banner | Inner Mongolia | -3.94 (-6.47, -1.34) | -6.64 (-17.15, 5.20) | 8.87 (-6.21, 26.37) | -11.97 (-30.08, 10.83) |
| Horinger | Inner Mongolia | -7.77 (-10.64, -4.80) | -14.43 (-31.57, 7.01) | -3.90 (-14.28, 7.75) | 3.40 (-12.81, 22.61) |
| Horqin Left Rear Banner | Inner Mongolia | 0.76 (-1.07, 2.63) | -4.91 (-17.36, 9.42) | 5.09 (-5.40, 16.75) | 1.45 (-16.87, 23.81) |
| Horqin Left Wing Middle Banner | Inner Mongolia | 0.31 (-1.58, 2.24) | 7.14 (-9.55, 26.91) | 1.99 (-1.58, 5.70) | 0.15 (-11.15, 12.89) |
| Horqin Right Front Banner | Inner Mongolia | -1.72 (-3.60, 0.20) | -10.65 (-19.05, -1.37) | -0.54 (-8.48, 8.09) | -0.78 (-19.49, 22.27) |
| Horqin Right Middle Banner | Inner Mongolia | -5.27 (-8.31, -2.14) | -9.83 (-19.65, 1.18) | -5.88 (-12.49, 1.22) | -0.75 (-16.14, 17.46) |
| Huade | Inner Mongolia | -6.94 (-9.21, -4.61) | 4.42 (-9.87, 20.97) | -10.50 (-22.17, 2.92) | -9.00 (-17.91, 0.89) |
| Huolinguole | Inner Mongolia | -9.44 (-13.25, -5.46) | -17.15 (-33.88, 3.80) | -2.01 (-31.71, 40.59) | 13.52 (-13.99, 49.82) |
| Hure Banner | Inner Mongolia | -0.51 (-3.77, 2.86) | 7.43 (-9.60, 27.66) | -1.09 (-17.57, 18.69) | -7.11 (-24.69, 14.56) |
| Jalaid Banner | Inner Mongolia | -1.19 (-3.93, 1.63) | -9.71 (-30.14, 16.70) | 2.09 (-7.10, 12.19) | 13.89 (0.13, 29.55) |
| Jungar Banner | Inner Mongolia | -9.78 (-12.01, -7.49) | -2.20 (-15.96, 13.82) | -6.65 (-30.64, 25.63) | -10.00 (-17.23, -2.14) |
| Kailu | Inner Mongolia | -1.77 (-2.93, -0.60) | 0.58 (-13.31, 16.70) | 2.46 (-2.58, 7.76) | 0.08 (-4.45, 4.82) |
| Liangcheng | Inner Mongolia | -1.89 (-4.06, 0.32) | -11.49 (-29.75, 11.53) | -1.23 (-14.25, 13.78) | -5.04 (-16.02, 7.37) |
| Linxi | Inner Mongolia | -3.15 (-4.98, -1.28) | -8.30 (-16.60, 0.83) | 4.29 (-5.50, 15.10) | -12.25 (-21.93, -1.36) |
| Manzhouli | Inner Mongolia | -6.87 (-12.62, -0.75) | -3.87 (-29.13, 30.40) | -32.73 (-42.82, -20.85) | 6.61 (-38.71, 85.43) |
| Molidavar Daur autonomous banner | Inner Mongolia | -5.70 (-9.42, -1.83) | -18.97 (-26.79, -10.31) | -8.56 (-17.37, 1.18) | 7.96 (-6.08, 24.11) |
| Naiman Banner | Inner Mongolia | 1.98 (-0.26, 4.28) | 12.69 (0.49, 26.38) | -1.43 (-16.32, 16.11) | -4.72 (-20.97, 14.86) |
| New Barag Left Banner | Inner Mongolia | -4.13 (-7.61, -0.52) | -1.34 (-2.17, -0.50) | -19.64 (-29.89, -7.89) | 14.10 (-20.14, 63.00) |
| New Barag Righr Banner | Inner Mongolia | -4.68 (-9.19, 0.05) | -19.59 (-35.98, 1.01) | -21.22 (-32.07, -8.64) | 14.23 (-3.95, 35.83) |
| Ningcheng | Inner Mongolia | -6.24 (-8.64, -3.78) | -2.87 (-13.94, 9.62) | -18.73 (-31.54, -3.51) | 2.52 (-14.53, 22.98) |
| Old Barag Banner | Inner Mongolia | -3.47 (-6.16, -0.70) | -15.11 (-27.79, -0.21) | -2.02 (-19.49, 19.24) | 10.31 (-1.58, 23.64) |
| Ongniud Banner | Inner Mongolia | -5.15 (-6.97, -3.30) | -4.25 (-12.49, 4.77) | -6.27 (-19.22, 8.76) | -8.58 (-17.98, 1.91) |
| Oroqen Autonomous Banner | Inner Mongolia | -3.30 (-5.70, -0.83) | -1.25 (-14.44, 13.97) | -5.45 (-25.43, 19.87) | 9.33 (-7.65, 29.43) |
| Qahar Right Front Banner | Inner Mongolia | -5.90 (-9.87, -1.74) | -9.53 (-38.23, 32.51) | -19.05 (-48.63, 27.55) | 4.13 (-0.36, 8.82) |
| Qahar Right Middle Banner | Inner Mongolia | -9.12 (-11.77, -6.39) | -16.16 (-28.24, -2.04) | -9.74 (-30.80, 17.71) | 1.52 (-12.46, 17.74) |
| Qahar Right Rear Banner | Inner Mongolia | -4.84 (-7.28, -2.34) | -3.55 (-4.38, -2.72) | -12.32 (-34.86, 18.01) | -7.39 (-21.49, 9.25) |
| Qingshuihe | Inner Mongolia | -3.16 (-4.67, -1.63) | -7.17 (-20.42, 8.29) | 0.61 (-9.50, 11.85) | -2.50 (-15.02, 11.86) |
| Shangdou | Inner Mongolia | -5.87 (-8.33, -3.35) | 1.58 (-13.11, 18.75) | -15.53 (-35.98, 11.45) | -5.37 (-9.53, -1.02) |
| Siziwang Banner | Inner Mongolia | -3.74 (-7.28, -0.05) | 1.59 (-22.29, 32.82) | -9.12 (-33.90, 24.94) | 11.95 (-4.34, 31.02) |
| Sonid Left Banner | Inner Mongolia | -5.90 (-9.47, -2.20) | -16.71 (-22.38, -10.62) | -19.99 (-37.12, 1.81) | 14.60 (-5.49, 38.95) |
| Sonid Right Banner | Inner Mongolia | -5.94 (-8.45, -3.37) | -0.24 (-1.88, 1.42) | -10.72 (-26.04, 7.76) | 2.96 (-19.67, 31.96) |
| Taipusi Banner | Inner Mongolia | -6.24 (-8.85, -3.56) | 1.37 (-8.38, 12.16) | -15.78 (-19.61, -11.77) | -5.51 (-33.35, 33.98) |
| Tumed Left Banner | Inner Mongolia | -5.49 (-8.88, -1.97) | -10.05 (-18.50, -0.73) | -14.04 (-34.42, 12.68) | 2.40 (-25.56, 40.86) |
| Tumed Right Banner | Inner Mongolia | -8.78 (-11.16, -6.34) | -11.01 (-17.99, -3.44) | -10.07 (-21.27, 2.72) | 7.16 (-7.71, 24.43) |
| Tuoketuo | Inner Mongolia | -6.93 (-10.74, -2.96) | -4.71 (-28.60, 27.17) | -19.72 (-34.01, -2.34) | -10.34 (-25.11, 7.35) |
| Tuquan | Inner Mongolia | -0.54 (-3.64, 2.65) | -17.54 (-30.24, -2.52) | -0.91 (-28.72, 37.75) | 2.21 (-2.44, 7.08) |
| Ulanhot | Inner Mongolia | -5.35 (-10.46, 0.04) | -3.61 (-13.45, 7.35) | -12.06 (-15.04, -8.98) | 27.97 (-18.96, 102.06) |
| Urad Front Banner | Inner Mongolia | -2.13 (-4.77, 0.58) | -4.28 (-14.48, 7.14) | -3.42 (-21.32, 18.55) | 14.86 (0.87, 30.80) |
| Urad Rear Banner | Inner Mongolia | -5.54 (-9.69, -1.19) | -3.93 (-17.77, 12.25) | -19.45 (-27.97, -9.92) | 31.20 (10.48, 55.81) |
| Uxin Banner | Inner Mongolia | -7.87 (-9.26, -6.47) | -3.10 (-14.79, 10.18) | -10.88 (-21.35, 0.99) | -10.38 (-20.13, 0.56) |
| West Ujimqin Banner | Inner Mongolia | -4.32 (-6.72, -1.86) | -6.55 (-18.87, 7.63) | -4.71 (-25.94, 22.62) | 0.09 (-21.48, 27.59) |
| Wuchuan | Inner Mongolia | -5.77 (-8.20, -3.26) | -6.29 (-12.61, 0.48) | -1.35 (-16.42, 16.45) | -15.20 (-30.32, 3.21) |
| Wulate Middle Banner | Inner Mongolia | -2.46 (-4.67, -0.19) | 15.25 (6.99, 24.14) | -4.92 (-8.12, -1.62) | 5.42 (-1.09, 12.35) |
| Wuyuan | Inner Mongolia | -6.47 (-8.44, -4.46) | -6.95 (-15.57, 2.54) | -8.33 (-14.59, -1.60) | 1.31 (-18.17, 25.42) |
| Xilinhot | Inner Mongolia | -7.02 (-9.57, -4.41) | -12.02 (-24.18, 2.09) | -10.94 (-36.99, 25.88) | -3.63 (-12.07, 5.62) |
| Xinghe | Inner Mongolia | -2.76 (-5.82, 0.41) | -2.24 (-14.08, 11.24) | -12.67 (-38.67, 24.35) | 1.10 (-16.01, 21.70) |
| Yakeshi | Inner Mongolia | -4.31 (-7.95, -0.53) | -8.00 (-18.80, 4.24) | -4.27 (-38.84, 49.84) | 18.33 (5.37, 32.88) |
| Zarout Banner | Inner Mongolia | -1.13 (-2.89, 0.66) | -7.49 (-19.45, 6.26) | -1.46 (-9.43, 7.22) | 6.37 (-8.87, 24.15) |
| Zhalantun | Inner Mongolia | -2.24 (-7.13, 2.91) | -1.53 (-27.26, 33.29) | -25.41 (-45.97, 2.97) | 21.78 (8.84, 36.26) |
| Zhenglan Banner | Inner Mongolia | -10.15 (-12.20, -8.06) | -7.27 (-21.54, 9.60) | -16.72 (-26.91, -5.11) | -8.69 (-21.88, 6.73) |
| Zhengxiangbai Banner | Inner Mongolia | -8.62 (-10.97, -6.21) | -7.84 (-19.19, 5.10) | -8.80 (-26.80, 13.63) | -8.47 (-28.73, 17.56) |
| Zhuozi | Inner Mongolia | -2.69 (-5.70, 0.41) | -11.53 (-16.37, -6.41) | 4.58 (-28.13, 52.18) | -4.46 (-10.45, 1.94) |
| Baoying | Jiangsu | -4.29 (-7.05, -1.44) | -18.88 (-25.04, -12.21) | 7.15 (-6.59, 22.91) | -12.21 (-21.13, -2.28) |
| Binhai | Jiangsu | -5.90 (-7.66, -4.11) | -10.04 (-19.57, 0.61) | 3.01 (-7.58, 14.80) | -3.67 (-15.40, 9.70) |
| Changshu | Jiangsu | -4.14 (-5.16, -3.10) | 1.30 (-10.30, 14.40) | -5.68 (-8.79, -2.47) | -2.59 (-5.38, 0.28) |
| Dafeng | Jiangsu | -6.13 (-8.40, -3.8) | -1.18 (-8.99, 7.29) | -2.13 (-6.40, 2.33) | -12.56 (-23.87, 0.42) |
| Danyang | Jiangsu | -5.09 (-7.22, -2.91) | -9.10 (-34.46, 26.05) | -5.86 (-10.30, -1.21) | -5.85 (-13.74, 2.75) |
| Donghai | Jiangsu | -2.10 (-5.19, 1.09) | -6.72 (-22.20, 11.84) | 1.57 (-5.61, 9.30) | -10.64 (-14.00, -7.16) |
| Dongtai | Jiangsu | -3.78 (-5.69, -1.82) | -6.60 (-11.01, -1.98) | -7.59 (-30.89, 23.58) | -7.57 (-11.17, -3.83) |
| Feng | Jiangsu | -7.11 (-9.29, -4.88) | -0.57 (-12.61, 13.13) | -8.83 (-14.76, -2.49) | -19.01 (-24.89, -12.67) |
| Funing | Jiangsu | -1.77 (-4.22, 0.75) | 4.48 (-16.39, 30.57) | 7.31 (-9.29, 26.95) | -8.12 (-21.13, 7.05) |
| Ganyu | Jiangsu | -2.37 (-4.09, -0.61) | -2.60 (-12.46, 8.38) | -1.22 (-12.65, 11.72) | -10.85 (-15.15, -6.35) |
| Gaochun | Jiangsu | -2.35 (-5.10, 0.48) | -16.88 (-34.17, 4.96) | 8.90 (-1.14, 19.95) | -8.35 (-15.65, -0.42) |
| Gaoyou | Jiangsu | -5.91 (-7.79, -4.00) | -15.89 (-27.28, -2.72) | -0.05 (-10.11, 11.13) | -7.32 (-15.19, 1.27) |
| Guannan | Jiangsu | -5.05 (-7.88, -2.14) | 0.92 (-6.33, 8.73) | 0.06 (-12.86, 14.90) | -15.32 (-23.88, -5.80) |
| Guanyun | Jiangsu | -2.51 (-6.53, 1.69) | -11.27 (-31.04, 14.15) | 6.42 (-0.68, 14.03) | -14.33 (-18.42, -10.04) |
| Haian | Jiangsu | -7.50 (-9.43, -5.52) | -10.01 (-20.30, 1.61) | 0.52 (-11.35, 13.98) | -18.22 (-24.30, -11.65) |
| Haimen | Jiangsu | -6.92 (-8.27, -5.55) | -8.54 (-18.57, 2.72) | -1.56 (-7.49, 4.75) | -9.57 (-21.23, 3.81) |
| Hongze | Jiangsu | -6.57 (-9.12, -3.94) | -10.52 (-15.95, -4.75) | -11.97 (-36.46, 21.96) | 3.72 (-7.72, 16.57) |
| Jiangdou | Jiangsu | -5.46 (-6.76, -4.15) | -5.79 (-12.70, 1.67) | -3.29 (-10.90, 4.96) | -12.35 (-18.29, -5.99) |
| Jiangyan | Jiangsu | -5.94 (-8.12, -3.72) | 4.09 (-2.87, 11.55) | -14.57 (-31.12, 5.97) | -4.94 (-15.39, 6.80) |
| Jiangyin | Jiangsu | -6.24 (-7.45, -5.01) | -13.31 (-20.25, -5.76) | -6.03 (-9.77, -2.14) | -3.42 (-7.91, 1.30) |
| Jianhu | Jiangsu | -5.28 (-7.40, -3.12) | -2.96 (-8.36, 2.77) | -0.77 (-13.82, 14.25) | -16.03 (-24.54, -6.56) |
| Jingjiang | Jiangsu | -5.24 (-6.20, -4.28) | -6.90 (-9.82, -3.88) | -7.01 (-10.40, -3.50) | 0.25 (-8.94, 10.37) |
| Jinhu | Jiangsu | -5.51 (-7.29, -3.71) | -11.59 (-25.63, 5.09) | 0.72 (-8.34, 10.68) | -4.39 (-12.51, 4.48) |
| Jintan | Jiangsu | -5.86 (-6.95, -4.75) | -10.22 (-17.62, -2.16) | -7.43 (-14.40, 0.10) | -3.78 (-10.92, 3.93) |
| Jurong | Jiangsu | -7.19 (-8.96, -5.38) | -6.38 (-16.22, 4.62) | -11.06 (-24.09, 4.21) | -4.73 (-12.76, 4.04) |
| Kunshan | Jiangsu | -8.98 (-12.37, -5.46) | -1.27 (-10.87, 9.37) | -15.26 (-35.63, 11.57) | 4.97 (-1.23, 11.55) |
| Lianshui | Jiangsu | -5.87 (-7.58, -4.14) | -9.52 (-19.26, 1.38) | -1.39 (-5.68, 3.09) | -9.63 (-23.38, 6.58) |
| Lishui | Jiangsu | -7.73 (-11.17, -4.15) | -18.09 (-35.37, 3.82) | -10.75 (-34.72, 22.02) | -4.40 (-14.22, 6.54) |
| Liyang | Jiangsu | -6.01 (-7.93, -4.06) | -10.35 (-16.73, -3.47) | -6.36 (-18.75, 7.92) | 4.96 (-7.18, 18.68) |
| Pei | Jiangsu | -9.76 (-11.98, -7.49) | -2.66 (-17.40, 14.71) | -22.34 (-33.45, -9.38) | -13.57 (-19.61, -7.07) |
| Pizhou | Jiangsu | -4.35 (-5.91, -2.76) | 5.78 (-1.23, 13.28) | -4.03 (-11.92, 4.57) | -7.02 (-11.07, -2.79) |
| Qidong | Jiangsu | -6.08 (-7.11, -5.04) | -2.96 (-12.95, 8.16) | -6.05 (-9.68, -2.27) | -10.87 (-15.30, -6.22) |
| Rudong | Jiangsu | -7.75 (-9.86, -5.58) | -13.3 (-23.89, -1.25) | 4.63 (-9.84, 21.41) | -16.10 (-19.72, -12.31) |
| Rugao | Jiangsu | -6.54 (-8.18, -4.86) | -4.44 (-16.17, 8.93) | 2.34 (-6.77, 12.34) | -11.45 (-20.70, -1.13) |
| Sheyang | Jiangsu | -8.33 (-9.67, -6.98) | -6.59 (-16.10, 4.00) | -12.82 (-24.58, 0.78) | -7.53 (-16.58, 2.51) |
| Shuyang | Jiangsu | -3.81 (-6.04, -1.53) | -6.69 (-16.95, 4.83) | -0.74 (-12.11, 12.10) | -10.47 (-21.40, 1.97) |
| Sihong | Jiangsu | -6.32 (-8.07, -4.54) | -1.63 (-19.18, 19.74) | -4.92 (-19.86, 12.80) | -2.19 (-10.83, 7.30) |
| Siyang | Jiangsu | -1.29 (-3.90, 1.38) | -8.95 (-25.21, 10.84) | 12.59 (-10.07, 40.95) | -6.47 (-14.74, 2.60) |
| Suining | Jiangsu | -5.71 (-7.49, -3.89) | 0.18 (-6.30, 7.12) | -4.16 (-8.54, 0.43) | -15.83 (-20.26, -11.15) |
| Taicang | Jiangsu | -5.04 (-6.83, -3.21) | -11.32 (-25.42, 5.44) | -8.59 (-16.93, 0.58) | 3.29 (-2.14, 9.02) |
| Taixing | Jiangsu | -9.67 (-11.34, -7.96) | -5.35 (-14.72, 5.05) | -16.54 (-24.98, -7.16) | -1.80 (-13.93, 12.04) |
| Tongshan | Jiangsu | -9.29 (-11.85, -6.66) | 7.11 (1.10, 13.49) | -8.71 (-29.77, 18.66) | -5.49 (-11.97, 1.46) |
| Tongzhou | Jiangsu | -7.88 (-8.87, -6.89) | -10.44 (-19.81, 0.03) | -6.07 (-13.02, 1.44) | -11.33 (-16.05, -6.33) |
| Wujiang | Jiangsu | -6.76 (-8.30, -5.19) | -5.38 (-9.21, -1.38) | -7.80 (-19.42, 5.49) | 1.01 (-1.65, 3.75) |
| Xiangshui | Jiangsu | -4.56 (-6.39, -2.68) | -4.70 (-18.03, 10.81) | -2.25 (-15.89, 13.59) | -6.77 (-18.78, 7.02) |
| Xinghua | Jiangsu | -4.84 (-6.56, -3.10) | 4.12 (-13.49, 25.33) | -6.78 (-13.36, 0.29) | -7.17 (-10.84, -3.34) |
| Xinyi | Jiangsu | -3.91 (-5.66, -2.12) | -1.37 (-12.56, 11.25) | -0.73 (-6.42, 5.29) | -12.99 (-23.77, -0.69) |
| Xuyi | Jiangsu | -6.21 (-8.63, -3.73) | -17.69 (-36.92, 7.41) | -1.34 (-12.15, 10.80) | -5.66 (-17.50, 7.87) |
| Yangzhong | Jiangsu | -4.63 (-7.05, -2.15) | -0.97 (-27.40, 35.09) | 0.28 (-6.19, 7.19) | -14.31 (-23.43, -4.11) |
| Yixing | Jiangsu | -6.66 (-7.87, -5.44) | -11.71 (-20.94, -1.41) | -4.74 (-7.42, -1.97) | -2.28 (-9.23, 5.19) |
| Yizheng | Jiangsu | -6.07 (-8.20, -3.89) | -15.08 (-26.09, -2.42) | 3.49 (-7.27, 15.49) | -12.88 (-24.07, -0.03) |
| Zhangjiagang | Jiangsu | -7.18 (-8.59, -5.74) | -11.58 (-21.54, -0.36) | -6.86 (-13.28, 0.04) | -3.32 (-5.43, -1.16) |
| Anfu | Jiangxi | -4.66 (-5.72, -3.59) | -8.89 (-19.83, 3.54) | -4.88 (-9.67, 0.18) | -3.07 (-9.56, 3.89) |
| Anyi | Jiangxi | 1.44 (-1.94, 4.94) | -13.42 (-34.70, 14.79) | 11.82 (-11.64, 41.52) | -7.00 (-11.35, -2.42) |
| Anyuan | Jiangxi | -7.16 (-9.92, -4.32) | -4.08 (-12.19, 4.78) | -5.72 (-13.35, 2.58) | 0.85 (-10.35, 13.44) |
| Chaisang | Jiangxi | -0.56 (-2.23, 1.14) | -8.94 (-16.01, -1.27) | 6.43 (-3.64, 17.55) | 0.35 (-9.60, 11.40) |
| Chongren | Jiangxi | -4.51 (-6.15, -2.85) | -12.01 (-20.14, -3.06) | -7.83 (-14.74, -0.36) | -0.24 (-4.41, 4.11) |
| Chongyi | Jiangxi | -0.51 (-3.49, 2.56) | -15.79 (-38.87, 15.98) | 8.30 (-0.21, 17.54) | -1.33 (-9.79, 7.92) |
| Dayu | Jiangxi | -5.07 (-7.70, -2.36) | -17.65 (-33.70, 2.28) | -8.83 (-21.05, 5.28) | 4.19 (-4.68, 13.89) |
| Dean | Jiangxi | -2.33 (-4.65, 0.04) | -12.23 (-16.26, -8.00) | 2.82 (-16.02, 25.87) | -9.18 (-15.35, -2.56) |
| Dexing | Jiangxi | -2.85 (-4.39, -1.28) | 2.58 (-11.74, 19.22) | -5.56 (-16.86, 7.26) | -3.59 (-14.46, 8.67) |
| Dingnan | Jiangxi | -4.28 (-6.21, -2.30) | -2.98 (-23.54, 23.10) | -12.29 (-17.76, -6.45) | -3.01 (-12.31, 7.28) |
| Dongxiang | Jiangxi | -5.90 (-9.35, -2.33) | -5.84 (-21.13, 12.40) | -5.61 (-14.58, 4.29) | 17.25 (1.07, 36.02) |
| Douchang | Jiangxi | -1.82 (-3.23, -0.38) | -4.43 (-13.05, 5.06) | 4.32 (-5.49, 15.16) | -1.14 (-9.25, 7.70) |
| Fengcheng | Jiangxi | -1.09 (-3.54, 1.43) | -10.56 (-34.62, 22.34) | 0.06 (-10.32, 11.65) | 6.40 (2.08, 10.92) |
| Fengxin | Jiangxi | -0.76 (-2.97, 1.49) | -7.16 (-31.85, 26.46) | -3.49 (-10.20, 3.73) | 4.70 (-1.27, 11.04) |
| Fenyi | Jiangxi | -7.09 (-9.53, -4.58) | -17.53 (-26.02, -8.06) | -15.08 (-30.07, 3.12) | 0.52 (-9.96, 12.21) |
| Fuliang | Jiangxi | -4.81 (-6.23, -3.36) | -9.81 (-19.52, 1.08) | -6.95 (-14.08, 0.78) | -3.11 (-15.76, 11.45) |
| Gan | Jiangxi | -3.36 (-6.19, -0.44) | -19.06 (-37.55, 4.91) | 2.51 (-7.11, 13.12) | -7.28 (-17.56, 4.28) |
| Gaoan | Jiangxi | -4.63 (-6.04, -3.20) | -5.06 (-16.50, 7.94) | -9.31 (-16.35, -1.68) | 0.66 (-11.34, 14.29) |
| Guangchang | Jiangxi | -5.49 (-7.33, -3.61) | -10.03 (-15.09, -4.67) | -2.13 (-14.35, 11.84) | -10.46 (-20.92, 1.38) |
| Guangfeng | Jiangxi | -2.56 (-4.33, -0.76) | 0.98 (-18.85, 25.67) | -7.81 (-18.61, 4.42) | -4.42 (-9.81, 1.28) |
| Guangxin | Jiangxi | -3.84 (-5.95, -1.68) | -7.86 (-29.32, 20.11) | -13.25 (-20.45, -5.39) | -0.98 (-7.85, 6.41) |
| Guixi | Jiangxi | -3.64 (-4.79, -2.47) | -2.47 (-11.07, 6.96) | -2.44 (-5.03, 0.23) | -1.21 (-4.45, 2.13) |
| Hengfeng | Jiangxi | -1.24 (-3.13, 0.68) | -2.07 (-21.83, 22.69) | -5.37 (-17.35, 8.36) | 3.81 (-7.80, 16.88) |
| Huichang | Jiangxi | -0.97 (-3.75, 1.89) | -14.69 (-29.94, 3.89) | 1.91 (-10.57, 16.12) | 10.72 (1.03, 21.35) |
| Hukou | Jiangxi | -1.21 (-2.88, 0.49) | -6.15 (-24.05, 15.97) | 0.45 (-9.85, 11.93) | -3.29 (-6.05, -0.44) |
| Jian | Jiangxi | -3.66 (-5.84, -1.43) | -11.10 (-16.00, -5.93) | 2.73 (-5.04, 11.14) | 3.01 (-3.58, 10.06) |
| Jingan | Jiangxi | -2.70 (-5.33, 0.00) | -8.61 (-37.35, 33.32) | 1.08 (-6.87, 9.71) | -5.57 (-19.83, 11.23) |
| Jinggangshan | Jiangxi | -8.49 (-10.56, -6.37) | -12.67 (-29.06, 7.51) | -12.89 (-25.3, 1.58) | -1.10 (-16.44, 17.07) |
| Jinxi | Jiangxi | -8.77 (-10.25, -7.26) | -5.13 (-19.18, 11.36) | -10.14 (-20.10, 1.06) | -2.78 (-10.91, 6.10) |
| Jinxian | Jiangxi | -3.86 (-5.77, -1.90) | -12.88 (-23.00, -1.43) | -2.50 (-13.58, 10.00) | 1.60 (-6.52, 10.43) |
| Jishui | Jiangxi | -6.11 (-7.28, -4.92) | -8.39 (-14.47, -1.87) | -0.87 (-8.51, 7.40) | -0.84 (-8.78, 7.80) |
| Le'an | Jiangxi | -3.18 (-4.30, -2.04) | 0.56 (-12.18, 15.15) | -2.50 (-6.31, 1.46) | -5.11 (-10.36, 0.45) |
| Leping | Jiangxi | -1.30 (-3.73, 1.18) | -12.64 (-33.68, 15.07) | 4.13 (-2.73, 11.48) | -6.90 (-10.49, -3.17) |
| Lianhua | Jiangxi | -2.74 (-5.45, 0.05) | -0.23 (-6.53, 6.50) | 4.15 (-19.33, 34.46) | -6.43 (-13.71, 1.45) |
| Lichuan | Jiangxi | -2.04 (-4.16, 0.13) | -1.34 (-10.37, 8.60) | 0.85 (-5.25, 7.35) | -14.22 (-20.95, -6.92) |
| Longnan | Jiangxi | -3.85 (-6.39, -1.25) | -10.31 (-36.62, 26.93) | -2.16 (-5.02, 0.79) | -9.63 (-23.58, 6.86) |
| Lushan | Jiangxi | -2.01 (-3.41, -0.58) | -4.00 (-17.23, 11.33) | -8.04 (-12.34, -3.53) | 0.30 (-8.99, 10.54) |
| Luxi | Jiangxi | -1.65 (-3.05, -0.23) | -1.40 (-16.01, 15.75) | 1.04 (-4.74, 7.17) | 1.72 (-6.82, 11.03) |
| Nanchang | Jiangxi | -3.05 (-5.06, -1.00) | -12.29 (-23.27, 0.25) | -2.19 (-4.92, 0.61) | -12.65 (-24.18, 0.63) |
| Nancheng | Jiangxi | -2.63 (-4.14, -1.10) | -3.14 (-19.38, 16.38) | -1.53 (-4.84, 1.90) | 3.53 (0.55, 6.59) |
| Nanfeng | Jiangxi | -0.36 (-2.12, 1.44) | -9.96 (-20.03, 1.38) | 6.10 (-1.12, 13.84) | -0.68 (-9.02, 8.42) |
| Nankang | Jiangxi | -4.53 (-6.26, -2.76) | -10.31 (-24.78, 6.95) | -7.40 (-18.62, 5.37) | 1.21 (-9.44, 13.12) |
| Ningdou | Jiangxi | -2.91 (-4.43, -1.37) | -4.36 (-20.72, 15.38) | -2.78 (-5.80, 0.34) | -8.11 (-13.51, -2.37) |
| Pengze | Jiangxi | -5.25 (-6.92, -3.56) | 1.85 (-1.44, 5.25) | -9.10 (-18.17, 0.98) | -3.12 (-7.59, 1.56) |
| Poyang | Jiangxi | -3.14 (-5.30, -0.93) | -5.06 (-23.56, 17.91) | -1.67 (-14.43, 13.00) | -4.40 (-13.52, 5.69) |
| Quannan | Jiangxi | -4.69 (-6.15, -3.22) | -2.10 (-19.88, 19.65) | -5.31 (-15.47, 6.08) | -3.25 (-9.81, 3.79) |
| Ruichang | Jiangxi | -3.65 (-4.98, -2.29) | -1.67 (-12.48, 10.49) | -5.80 (-14.38, 3.65) | 3.31 (-0.65, 7.42) |
| Ruijin | Jiangxi | -3.97 (-6.34, -1.53) | -12.64 (-25.87, 2.96) | -9.38 (-19.70, 2.27) | 3.84 (0.06, 7.76) |
| Shanggao | Jiangxi | -0.55 (-3.07, 2.03) | -8.44 (-33.32, 25.74) | 0.19 (-6.63, 7.51) | -6.26 (-18.84, 8.28) |
| Shangli | Jiangxi | -1.67 (-4.05, 0.77) | -7.33 (-29.96, 22.6) | -6.31 (-12.64, 0.48) | 4.75 (-2.65, 12.72) |
| Shangyou | Jiangxi | -1.80 (-3.82, 0.27) | -5.17 (-19.28, 11.40) | -1.26 (-17.87, 18.72) | -0.62 (-8.78, 8.27) |
| Shicheng | Jiangxi | -2.33 (-3.77, -0.87) | -0.92 (-13.93, 14.06) | -2.19 (-11.27, 7.82) | 4.72 (-5.05, 15.48) |
| Suichuan | Jiangxi | -3.84 (-5.67, -1.97) | -9.66 (-30.37, 17.19) | -7.02 (-14.44, 1.05) | -3.01 (-9.33, 3.74) |
| Taihe | Jiangxi | -7.00 (-9.33, -4.62) | -16.95 (-34.30, 4.99) | -9.70 (-19.48, 1.27) | 3.10 (-1.40, 7.81) |
| Tonggu | Jiangxi | -0.56 (-2.70, 1.62) | 5.09 (-13.13, 27.13) | -5.01 (-12.17, 2.73) | -5.94 (-21.62, 12.88) |
| Wanan | Jiangxi | -7.29 (-9.92, -4.59) | -13.01 (-25.06, 0.98) | -14.1 (-25.83, -0.51) | 10.71 (-0.44, 23.11) |
| Wannian | Jiangxi | -1.97 (-4.29, 0.41) | -7.91 (-32.42, 25.51) | -7.15 (-17.59, 4.61) | 4.49 (-1.52, 10.87) |
| Wanzai | Jiangxi | 0.62 (-0.97, 2.25) | -1.29 (-16.78, 17.09) | -6.86 (-12.01, -1.41) | 0.57 (-9.85, 12.19) |
| Wuning | Jiangxi | -1.22 (-4.01, 1.64) | -14.58 (-29.40, 3.36) | -2.58 (-12.43, 8.38) | 4.09 (-18.62, 33.15) |
| Wuyuan | Jiangxi | -5.73 (-8.15, -3.24) | -14.84 (-35.97, 13.26) | -4.35 (-15.52, 8.28) | 1.16 (-14.12, 19.17) |
| Xiajiang | Jiangxi | -7.36 (-9.56, -5.12) | -4.66 (-19.89, 13.46) | -7.41 (-16.48, 2.64) | 4.26 (-17.98, 32.54) |
| Xinfeng | Jiangxi | -1.75 (-3.75, 0.29) | -2.14 (-12.70, 9.70) | 0.97 (-5.93, 8.37) | 2.64 (-3.37, 9.02) |
| Xingan | Jiangxi | -2.71 (-4.89, -0.47) | -16.14 (-30.85, 1.69) | 1.96 (-8.96, 14.20) | -1.37 (-3.75, 1.06) |
| Xingguo | Jiangxi | -3.74 (-6.34, -1.07) | -0.35 (-8.30, 8.29) | -8.65 (-16.22, -0.39) | -13.12 (-33.73, 13.89) |
| Xinjian | Jiangxi | -4.41 (-5.51, -3.31) | -5.38 (-14.41, 4.61) | -4.95 (-8.58, -1.17) | -5.58 (-17.10, 7.53) |
| Xiushui | Jiangxi | -4.17 (-5.33, -3.00) | -7.17 (-13.75, -0.09) | -4.63 (-14.72, 6.67) | -2.97 (-7.76, 2.08) |
| Xunwu | Jiangxi | -1.47 (-3.13, 0.21) | -0.15 (-19.63, 24.04) | -5.85 (-16.23, 5.82) | -2.07 (-8.85, 5.21) |
| Yanshan | Jiangxi | -4.14 (-5.52, -2.73) | -11.96 (-15.96, -7.76) | -5.53 (-10.37, -0.42) | -1.91 (-10.76, 7.82) |
| Yifeng | Jiangxi | -5.58 (-7.98, -3.12) | -10.99 (-22.40, 2.10) | 7.42 (-3.31, 19.34) | -0.70 (-5.69, 4.55) |
| Yihuang | Jiangxi | -4.26 (-6.60, -1.87) | -3.07 (-31.55, 37.26) | -1.86 (-18.59, 18.31) | -5.27 (-9.03, -1.34) |
| Yiyang | Jiangxi | -1.69 (-3.15, -0.21) | 0.72 (-8.98, 11.46) | -5.62 (-19.03, 10.02) | -4.65 (-11.96, 3.27) |
| Yongfeng | Jiangxi | -2.69 (-4.05, -1.30) | -0.81 (-17.63, 19.44) | -4.84 (-7.13, -2.49) | -7.09 (-9.93, -4.16) |
| Yongxin | Jiangxi | -6.16 (-8.44, -3.83) | -8.75 (-27.18, 14.33) | -4.12 (-8.62, 0.61) | 3.10 (-14.27, 23.99) |
| Yongxiu | Jiangxi | -4.67 (-6.28, -3.03) | 1.52 (-15.87, 22.51) | 0.17 (-1.18, 1.55) | -6.80 (-12.65, -0.57) |
| Yudou | Jiangxi | -4.24 (-5.82, -2.63) | -2.48 (-20.96, 20.32) | -7.23 (-15.44, 1.77) | -2.69 (-8.14, 3.09) |
| Yugan | Jiangxi | -4.92 (-6.87, -2.93) | -8.69 (-32.14, 22.87) | -6.79 (-14.13, 1.17) | -3.76 (-13.18, 6.68) |
| Yujiang | Jiangxi | -4.58 (-6.75, -2.37) | -16.99 (-33.48, 3.61) | -4.80 (-12.70, 3.81) | -0.63 (-4.51, 3.40) |
| Yushan | Jiangxi | -3.39 (-6.11, -0.59) | -3.49 (-9.97, 3.47) | -13.12 (-17.97, -7.97) | 14.24 (6.26, 22.82) |
| Zhangshu | Jiangxi | -1.96 (-3.49, -0.41) | -5.49 (-19.10, 10.42) | -2.92 (-10.96, 5.84) | 1.63 (-5.01, 8.74) |
| Zixi | Jiangxi | -4.28 (-7.19, -1.27) | -12.13 (-36.46, 21.52) | 0.15 (-18.07, 22.43) | 2.33 (-13.47, 21.01) |
| Antu | Jilin | -6.95 (-9.32, -4.51) | -1.33 (-22.33, 25.34) | -6.05 (-24.62, 17.10) | -13.55 (-19.20, -7.51) |
| Changbai | Jilin | -5.96 (-7.47, -4.43) | -5.33 (-17.09, 8.10) | -5.28 (-13.82, 4.11) | -10.21 (-22.84, 4.50) |
| Changling | Jilin | -2.98 (-4.31, -1.64) | -4.53 (-19.20, 12.80) | -6.40 (-14.53, 2.50) | -2.88 (-8.47, 3.04) |
| Daan | Jilin | -6.06 (-7.40, -4.69) | -6.67 (-14.40, 1.77) | -9.75 (-21.30, 3.50) | -10.04 (-20.41, 1.69) |
| Dehui | Jilin | -0.37 (-3.53, 2.90) | -1.99 (-16.05, 14.42) | 11.85 (-18.48, 53.46) | -15.59 (-23.68, -6.65) |
| Dongfeng | Jilin | -3.42 (-5.45, -1.35) | -0.86 (-16.83, 18.19) | -6.29 (-13.91, 2.02) | -10.54 (-18.78, -1.47) |
| Dongliao | Jilin | -7.43 (-8.69, -6.14) | -5.83 (-12.31, 1.13) | -9.79 (-23.53, 6.42) | -6.94 (-9.53, -4.28) |
| Dunhua | Jilin | -6.03 (-9.59, -2.33) | 8.11 (-19.61, 45.40) | -9.31 (-16.79, -1.15) | -9.76 (-21.63, 3.89) |
| Fusong | Jilin | -6.56 (-9.98, -3.02) | -8.04 (-34.96, 30.04) | -8.57 (-15.67, -0.88) | -21.12 (-29.15, -12.18) |
| Fuyu | Jilin | -7.36 (-9.30, -5.38) | 6.15 (-7.02, 21.18) | -11.27 (-17.03, -5.11) | -0.17 (-4.95, 4.86) |
| Gongzhuling | Jilin | -1.33 (-3.50, 0.88) | -6.67 (-22.44, 12.29) | -1.06 (-19.20, 21.17) | -7.24 (-17.65, 4.49) |
| Helong | Jilin | -7.49 (-10.74, -4.13) | 5.41 (-28.10, 54.54) | -14.00 (-25.75, -0.39) | -13.04 (-23.88, -0.66) |
| Huadian | Jilin | -9.39 (-11.38, -7.36) | -6.21 (-28.56, 23.12) | -17.3 (-26.09, -7.47) | -4.99 (-8.69, -1.14) |
| Huinan | Jilin | -6.74 (-9.27, -4.13) | -7.53 (-17.07, 3.12) | 0.13 (-26.67, 36.70) | 1.99 (-11.85, 18.00) |
| Hunchun | Jilin | -7.59 (-9.36, -5.78) | -6.25 (-23.03, 14.20) | -13.77 (-18.21, -9.09) | -3.84 (-13.23, 6.57) |
| Jian | Jilin | -6.11 (-7.60, -4.59) | -4.18 (-18.42, 12.54) | -12.49 (-21.00, -3.06) | -5.36 (-12.91, 2.84) |
| Jiangyuan | Jilin | -7.75 (-10.03, -5.41) | -12.14 (-30.15, 10.50) | -12.61 (-19.89, -4.67) | 2.32 (-18.79, 28.91) |
| Jiaohe | Jilin | -2.18 (-3.94, -0.38) | -4.61 (-25.86, 22.73) | -1.66 (-7.74, 4.83) | -2.41 (-14.23, 11.03) |
| Jingyu | Jilin | -8.74 (-11.04, -6.37) | -5.83 (-14.28, 3.46) | -13.53 (-23.80, -1.88) | -2.15 (-27.70, 32.43) |
| Jiutai | Jilin | -5.43 (-9.16, -1.54) | 1.68 (-13.70, 19.81) | -0.46 (-20.70, 24.94) | -16.3 (-20.59, -11.78) |
| Linjiang | Jilin | -5.38 (-7.11, -3.62) | -2.19 (-15.49, 13.19) | -7.87 (-12.07, -3.48) | -11.87 (-21.45, -1.12) |
| Lishu | Jilin | -2.21 (-5.27, 0.95) | -17.18 (-37.08, 9.02) | -3.79 (-22.43, 19.33) | 1.57 (-11.43, 16.47) |
| Liuhe | Jilin | -7.69 (-9.77, -5.57) | -0.09 (-12.11, 13.57) | -17.84 (-23.21, -12.09) | 2.16 (-6.35, 11.44) |
| Longjing | Jilin | -8.24 (-11.32, -5.05) | -1.14 (-24.38, 29.24) | -21.22 (-24.51, -17.80) | -19.46 (-25.06, -13.45) |
| Meihekou | Jilin | -5.95 (-7.54, -4.33) | -3.91 (-19.89, 15.25) | -10.05 (-16.73, -2.84) | -0.09 (-9.05, 9.74) |
| Nongan | Jilin | -3.51 (-6.24, -0.70) | 4.95 (-9.84, 22.18) | -3.59 (-17.66, 12.89) | -14.11 (-24.73, -1.99) |
| Panshi | Jilin | -5.34 (-6.50, -4.16) | -6.71 (-20.88, 10.00) | -6.12 (-13.57, 1.98) | -6.02 (-10.47, -1.34) |
| Qian Gorlos | Jilin | -4.94 (-7.08, -2.75) | -2.17 (-10.99, 7.52) | -7.37 (-16.52, 2.77) | -7.26 (-17.91, 4.76) |
| Qianan | Jilin | -7.74 (-9.90, -5.53) | -10.28 (-33.71, 21.41) | -11.69 (-16.35, -6.76) | -0.42 (-8.20, 8.02) |
| Shuangliao | Jilin | -3.25 (-4.45, -2.03) | -1.13 (-4.59, 2.46) | 0.64 (-6.96, 8.87) | -11.02 (-15.53, -6.28) |
| Shulan | Jilin | -5.28 (-7.26, -3.27) | 4.34 (-10.31, 21.39) | -1.97 (-10.49, 7.36) | -4.03 (-4.83, -3.23) |
| Taonan | Jilin | -5.88 (-7.94, -3.77) | 5.23 (-6.38, 18.27) | -10.41 (-14.35, -6.28) | -13.96 (-25.59, -0.52) |
| Tonghua | Jilin | -2.50 (-4.17, -0.79) | -3.09 (-18.25, 14.90) | -8.75 (-17.56, 1.00) | 0.20 (-4.68, 5.34) |
| Tongyu | Jilin | -4.01 (-6.50, -1.45) | -6.78 (-30.65, 25.30) | -4.16 (-9.89, 1.94) | -2.00 (-9.88, 6.56) |
| Tumen | Jilin | -5.63 (-8.60, -2.56) | 5.24 (-21.69, 41.42) | -6.29 (-22.70, 13.60) | -3.06 (-24.98, 25.27) |
| Wangqing | Jilin | -7.52 (-11.74, -3.09) | -0.91 (-29.26, 38.80) | -10.15 (-25.31, 8.09) | -24.94 (-35.01, -13.31) |
| Yanji | Jilin | -7.28 (-10.37, -4.08) | 5.13 (-29.32, 56.35) | -13.19 (-15.95, -10.34) | -3.77 (-16.50, 10.90) |
| Yitong | Jilin | -1.80 (-4.38, 0.84) | -9.52 (-24.51, 8.43) | -7.82 (-23.92, 11.70) | -4.64 (-6.63, -2.61) |
| Yongji | Jilin | -7.68 (-9.31, -6.03) | -7.02 (-16.60, 3.66) | -8.90 (-18.31, 1.60) | 0.26 (-12.89, 15.39) |
| Yushu | Jilin | -6.86 (-10.07, -3.53) | 8.16 (-6.25, 24.78) | -6.62 (-15.31, 2.97) | -18.3 (-23.43, -12.83) |
| Zhenlai | Jilin | -7.41 (-9.72, -5.04) | -7.71 (-22.54, 9.95) | -12.24 (-20.91, -2.61) | -14.78 (-24.45, -3.86) |
| Beipiao | Liaoning | -0.04 (-2.63, 2.63) | 10.57 (-6.17, 30.29) | -8.13 (-15.25, -0.41) | -1.17 (-17.44, 18.32) |
| Beizhen | Liaoning | -0.77 (-2.53, 1.01) | 2.41 (-6.07, 11.66) | -1.84 (-12.53, 10.17) | 0.79 (-9.85, 12.68) |
| Benxi | Liaoning | 1.28 (-0.58, 3.19) | -4.59 (-17.08, 9.78) | -2.79 (-12.22, 7.65) | 10.35 (7.98, 12.77) |
| Changhai | Liaoning | 0.91 (-1.17, 3.03) | 2.68 (-11.21, 18.74) | 0.10 (-17.35, 21.24) | 0.54 (-5.24, 6.67) |
| Changtu | Liaoning | 3.65 (1.80, 5.54) | 3.08 (-10.15, 18.26) | 16.29 (9.64, 23.34) | -0.55 (-7.17, 6.54) |
| Chaoyang | Liaoning | 4.24 (1.81, 6.74) | -2.83 (-17.94, 15.07) | 1.08 (-14.30, 19.22) | 2.21 (-18.58, 28.32) |
| Dashiqiao | Liaoning | 0.80 (-1.40, 3.05) | 1.53 (-10.39, 15.04) | 5.63 (-11.79, 26.50) | 5.24 (-2.97, 14.15) |
| Dawa | Liaoning | -3.76 (-5.38, -2.11) | -2.99 (-9.01, 3.42) | -3.53 (-10.21, 3.66) | -0.04 (-21.14, 26.72) |
| Dengta | Liaoning | -5.08 (-7.68, -2.41) | 9.95 (-9.66, 33.82) | -5.36 (-18.94, 10.48) | 7.36 (-0.06, 15.33) |
| Diaobingshan | Liaoning | -0.45 (-4.24, 3.49) | 14.10 (-27.5, 79.55) | -4.89 (-14.06, 5.26) | 3.98 (-4.57, 13.29) |
| Donggang | Liaoning | -1.18 (-2.23, -0.13) | -0.16 (-4.90, 4.82) | -0.60 (-11.44, 11.56) | -2.00 (-11.59, 8.63) |
| Faku | Liaoning | -1.40 (-3.01, 0.24) | 3.56 (-9.51, 18.50) | -4.92 (-15.47, 6.94) | -0.84 (-9.96, 9.22) |
| Fengcheng | Liaoning | 2.21 (0.83, 3.61) | 11.64 (-0.04, 24.69) | -1.25 (-7.32, 5.21) | 2.95 (0.54, 5.41) |
| Fushun | Liaoning | -1.15 (-3.83, 1.62) | 14.63 (7.16, 22.62) | -9.17 (-23.9, 8.42) | -0.6 (-11.81, 12.03) |
| Fuxin | Liaoning | 3.24 (1.26, 5.27) | 3.86 (-7.35, 16.42) | 6.88 (-5.32, 20.64) | -2.84 (-10.76, 5.78) |
| Gaizhou | Liaoning | 1.65 (-1.85, 5.28) | -2.86 (-21.71, 20.54) | 3.85 (-31.19, 56.75) | 1.25 (-0.63, 3.17) |
| Haicheng | Liaoning | 3.11 (1.87, 4.37) | -2.55 (-11.38, 7.16) | 2.05 (-4.47, 9.02) | 2.07 (-8.68, 14.09) |
| Heishan | Liaoning | 3.76 (1.42, 6.16) | 14.99 (-9.68, 46.39) | 1.58 (-4.53, 8.07) | 2.08 (-4.06, 8.63) |
| Huanren | Liaoning | -2.46 (-3.73, -1.17) | -6.85 (-12.54, -0.78) | -5.13 (-13.01, 3.47) | 0.68 (-11.08, 13.99) |
| Jianchang | Liaoning | -0.01 (-2.04, 2.06) | -0.38 (-12.76, 13.76) | 3.14 (-4.88, 11.83) | 14.03 (-1.53, 32.05) |
| Jianping | Liaoning | 0.20 (-1.36, 1.78) | -1.75 (-6.98, 3.76) | -5.99 (-14.14, 2.94) | 4.66 (-5.70, 16.15) |
| Kaiyuan | Liaoning | -0.84 (-4.56, 3.03) | -5.39 (-38.9, 46.51) | 22.39 (8.47, 38.11) | 1.07 (-10.48, 14.11) |
| Kangping | Liaoning | -4.39 (-6.88, -1.84) | -0.68 (-12.63, 12.92) | -13.49 (-26.84, 2.31) | 12.51 (3.46, 22.35) |
| Kazuo | Liaoning | 0.06 (-1.90, 2.06) | 0.04 (-6.12, 6.61) | -6.44 (-21.39, 11.34) | -4.14 (-21.63, 17.24) |
| Kuandian | Liaoning | 1.33 (-1.49, 4.24) | 2.60 (-19.60, 30.93) | 6.35 (-11.89, 28.37) | 9.19 (-8.46, 30.24) |
| Liaoyang | Liaoning | -5.01 (-7.94, -2.00) | 8.13 (-8.16, 27.32) | -12.18 (-23.37, 0.65) | 13.65 (1.44, 27.32) |
| Liaozhong | Liaoning | -7.25 (-11.61, -2.68) | 0.07 (-26.56, 36.35) | -15.08 (-38.73, 17.69) | 22.44 (5.26, 42.42) |
| Linghai | Liaoning | -0.14 (-1.77, 1.51) | 1.43 (-3.00, 6.06) | 1.37 (-7.83, 11.49) | 4.51 (-5.64, 15.75) |
| Lingyuan | Liaoning | -2.25 (-4.54, 0.10) | -4.68 (-18.19, 11.05) | -8.27 (-29.53, 19.41) | 4.62 (-10.54, 22.35) |
| Panshan | Liaoning | -3.93 (-5.50, -2.34) | -0.49 (-4.77, 3.98) | -2.26 (-5.31, 0.90) | -2.15 (-23.43, 25.05) |
| Pulandian | Liaoning | -0.34 (-1.97, 1.32) | -0.52 (-4.33, 3.44) | 1.31 (-10.94, 15.23) | -0.80 (-6.00, 4.68) |
| Qingyuan | Liaoning | 0.41 (-2.45, 3.35) | 1.31 (-5.24, 8.31) | -2.57 (-16.24, 13.32) | -5.21 (-24.34, 18.75) |
| Suizhong | Liaoning | 1.49 (-0.76, 3.79) | -8.27 (-28.37, 17.47) | -0.92 (-4.93, 3.25) | 3.85 (-10.36, 20.30) |
| Taian | Liaoning | 2.47 (1.42, 3.53) | 2.20 (-5.80, 10.88) | 3.62 (-4.31, 12.22) | -2.12 (-9.98, 6.43) |
| Tieling | Liaoning | 0.86 (-2.74, 4.58) | 2.58 (-27.29, 44.70) | 19.37 (-5.61, 50.94) | 6.74 (-6.23, 21.51) |
| Wafangdian | Liaoning | -0.54 (-1.42, 0.35) | 1.48 (-5.28, 8.72) | -0.21 (-9.93, 10.55) | -0.90 (-6.22, 4.72) |
| Xifeng | Liaoning | 2.16 (-0.63, 5.03) | 0.66 (-13.19, 16.71) | 23.77 (4.14, 47.10) | 7.96 (-0.22, 16.82) |
| Xinbin | Liaoning | -2.58 (-4.21, -0.91) | -1.01 (-12.60, 12.13) | -8.74 (-21.23, 5.74) | -5.38 (-16.89, 7.73) |
| Xingcheng | Liaoning | -3.12 (-4.77, -1.43) | 2.76 (-3.65, 9.58) | -1.75 (-15.96, 14.86) | 2.40 (-9.37, 15.69) |
| Xinmin | Liaoning | -0.37 (-1.93, 1.22) | 2.52 (-9.24, 15.79) | -4.56 (-12.42, 4.00) | 1.86 (-4.19, 8.29) |
| Xiuyan | Liaoning | -0.63 (-1.88, 0.63) | -0.07 (-7.87, 8.38) | -4.85 (-12.15, 3.06) | 6.24 (2.81, 9.79) |
| Yi | Liaoning | -0.87 (-3.06, 1.37) | 0.36 (-9.48, 11.26) | 3.95 (2.81, 5.11) | -11.63 (-16.8, -6.15) |
| Zhangwu | Liaoning | -3.35 (-5.40, -1.26) | -6.43 (-15.85, 4.05) | 5.52 (-1.13, 12.62) | 0.63 (-22.53, 30.72) |
| Zhuanghe | Liaoning | 0.51 (-0.49, 1.52) | -0.36 (-4.04, 3.45) | 3.16 (-3.85, 10.69) | -2.39 (-6.54, 1.94) |
| Haiyuan | Ningxia | -5.15 (-9.30, -0.81) | -8.88 (-32.41, 22.83) | 23.84 (5.90, 44.82) | -9.50 (-22.51, 5.70) |
| Helan | Ningxia | -3.13 (-5.80, -0.38) | 3.63 (-12.96, 23.38) | -6.08 (-12.01, 0.24) | 7.45 (-22.41, 48.81) |
| Jingyuan | Ningxia | -8.15 (-11.33, -4.85) | -3.90 (-31.28, 34.38) | -19.61 (-31.04, -6.28) | -9.51 (-32.13, 20.64) |
| Lingwu | Ningxia | -4.80 (-5.96, -3.61) | -1.93 (-14.56, 12.57) | -2.34 (-10.19, 6.20) | -2.98 (-9.65, 4.19) |
| Longde | Ningxia | -5.18 (-8.59, -1.64) | 11.89 (-25.52, 68.08) | -6.76 (-24.18, 14.67) | -4.15 (-17.73, 11.67) |
| Pengyang | Ningxia | -2.69 (-5.91, 0.63) | -13.66 (-33.11, 11.45) | -4.20 (-26.06, 24.12) | -6.88 (-23.76, 13.74) |
| Pingluo | Ningxia | -3.78 (-7.57, 0.17) | -3.06 (-11.88, 6.64) | 9.38 (1.38, 18.02) | 2.60 (-25.29, 40.91) |
| Qingtongxia | Ningxia | -0.29 (-2.92, 2.40) | -4.72 (-22.74, 17.49) | -0.04 (-5.31, 5.53) | -15.94 (-20.43, -11.20) |
| Tongxin | Ningxia | -1.53 (-3.77, 0.75) | -8.82 (-21.24, 5.55) | 8.38 (1.16, 16.11) | 1.99 (-5.53, 10.12) |
| Xiji | Ningxia | -1.61 (-4.31, 1.16) | -7.28 (-23.63, 12.57) | -0.97 (-24.65, 30.14) | -8.53 (-16.78, 0.54) |
| Yanchi | Ningxia | -0.08 (-3.30, 3.24) | -13.13 (-36.47, 18.79) | 15.51 (1.60, 31.32) | -5.80 (-16.69, 6.51) |
| Yongning | Ningxia | -5.27 (-7.38, -3.12) | -2.76 (-9.42, 4.39) | -13.27 (-24.70, -0.11) | 2.05 (-20.21, 30.51) |
| Zhongning | Ningxia | -7.42 (-10.89, -3.81) | -26.70 (-45.32, -1.75) | 0.65 (-9.47, 11.90) | -5.99 (-15.22, 4.24) |
| Banma | Qinghai | 6.78 (3.66, 9.98) | 6.55 (-11.03, 27.61) | 14.57 (-16.80, 57.76) | 4.21 (-7.36, 17.22) |
| Chengduo | Qinghai | 20.20 (11.96, 29.06) | 86.02 (24.80, 177.26) | -3.66 (-16.43, 11.06) | 13.23 (8.61, 18.05) |
| Dari | Qinghai | 9.08 (5.70, 12.57) | -1.12 (-10.90, 9.73) | 22.21 (4.13, 43.43) | -2.64 (-12.38, 8.19) |
| Datong | Qinghai | 1.66 (-2.15, 5.61) | -10.65 (-31.76, 16.98) | 16.07 (-7.04, 44.92) | -13.82 (-28.83, 4.36) |
| Delingha | Qinghai | -2.53 (-6.00, 1.07) | 1.16 (-33.22, 53.26) | -6.67 (-30.51, 25.34) | 7.76 (-11.49, 31.21) |
| Doulan | Qinghai | 2.18 (-0.47, 4.90) | -7.31 (-26.64, 17.11) | -2.47 (-18.54, 16.79) | 10.34 (-8.11, 32.50) |
| Gande | Qinghai | 10.25 (7.98, 12.56) | 10.67 (-3.49, 26.90) | 8.68 (-1.97, 20.49) | 8.19 (-0.11, 17.18) |
| Gangcha | Qinghai | 2.64 (-0.22, 5.59) | 0.67 (-12.84, 16.27) | -10.19 (-23.95, 6.07) | 8.82 (-9.41, 30.71) |
| Geermu | Qinghai | -7.83 (-10.37, -5.23) | -0.63 (-20.55, 24.28) | -12.72 (-27.73, 5.41) | -13.28 (-30.90, 8.82) |
| Gonghe | Qinghai | 2.31 (-0.78, 5.50) | -7.95 (-35.64, 31.66) | 7.62 (-12.15, 31.85) | -2.46 (-10.35, 6.12) |
| Guide | Qinghai | 1.22 (-1.27, 3.77) | -1.57 (-20.91, 22.51) | 6.24 (0.32, 12.50) | 3.84 (-22.51, 39.15) |
| Guinan | Qinghai | 6.51 (3.70, 9.39) | -5.60 (-27.54, 22.99) | 17.69 (14.31, 21.18) | 8.51 (-9.45, 30.02) |
| Haiyan | Qinghai | 6.11 (2.67, 9.66) | 12.84 (-10.20, 41.78) | 28.26 (24.97, 31.63) | -0.06 (-20.13, 25.05) |
| Henan | Qinghai | 9.55 (6.06, 13.15) | -4.83 (-14.91, 6.44) | 5.49 (-3.51, 15.32) | 27.34 (1.91, 59.12) |
| Hualong | Qinghai | 7.35 (2.56, 12.37) | 44.53 (-3.05, 115.45) | 7.72 (-6.98, 24.75) | 2.99 (-3.68, 10.13) |
| Huangyuan | Qinghai | 0.17 (-3.28, 3.75) | -6.71 (-24.42, 15.16) | -2.32 (-27.84, 32.22) | -4.19 (-27.97, 27.43) |
| Huangzhong | Qinghai | -3.92 (-5.98, -1.81) | -9.87 (-25.08, 8.43) | -2.47 (-16.39, 13.78) | -0.08 (-16.52, 19.61) |
| Huzhu | Qinghai | 2.71 (0.40, 5.08) | -4.86 (-15.90, 7.62) | -3.31 (-18.19, 14.28) | 2.19 (-2.01, 6.58) |
| Jianzha | Qinghai | 11.78 (6.38, 17.47) | 0.66 (-13.01, 16.48) | 24.15 (6.81, 44.32) | 1.92 (-14.05, 20.85) |
| Jiuzhi | Qinghai | 6.96 (3.84, 10.18) | 8.99 (-13.07, 36.66) | -3.07 (-11.74, 6.45) | -2.87 (-22.71, 22.07) |
| Ledou | Qinghai | -3.54 (-5.70, -1.33) | -6.21 (-25.45, 18.00) | 6.42 (-2.58, 16.26) | -5.34 (-17.90, 9.13) |
| Maduo | Qinghai | 6.65 (4.10, 9.27) | 11.40 (-6.52, 32.75) | 1.72 (-16.52, 23.93) | 2.47 (-13.83, 21.86) |
| Maqin | Qinghai | 6.08 (2.47, 9.82) | 5.48 (-1.38, 12.82) | 2.46 (-17.13, 26.69) | 13.97 (-8.17, 41.46) |
| Menyuan | Qinghai | 2.56 (-0.36, 5.57) | -0.55 (-7.81, 7.30) | 9.74 (-8.56, 31.71) | -5.99 (-26.35, 19.99) |
| Minhe | Qinghai | 3.33 (0.67, 6.06) | -7.02 (-16.64, 3.70) | 1.38 (-11.53, 16.18) | -0.77 (-14.23, 14.80) |
| Nangqian | Qinghai | 21.13 (9.48, 34.02) | 36.99 (-64.03, 421.82) | -0.63 (-22.96, 28.18) | 1.70 (-11.34, 16.66) |
| Pingan | Qinghai | -1.12 (-5.28, 3.22) | -4.34 (-20.43, 15.01) | 13.97 (-16.22, 55.04) | 23.55 (4.75, 45.71) |
| Qilian | Qinghai | 0.65 (-3.07, 4.51) | 11.87 (1.36, 23.47) | -4.53 (-27.08, 25.01) | 27.54 (8.48, 49.95) |
| Qumalai | Qinghai | 24.18 (16.16, 32.75) | 32.41 (-49.31, 245.88) | 29.42 (0.69, 66.36) | 4.45 (-9.73, 20.86) |
| Tianjun | Qinghai | -0.17 (-5.52, 5.48) | -4.68 (-12.38, 3.69) | 1.75 (-48.90, 102.59) | 2.69 (-21.17, 33.77) |
| Tongde | Qinghai | 13.80 (10.33, 17.38) | 12.14 (-25.08, 67.85) | 4.03 (-7.76, 17.34) | 16.99 (-5.25, 44.44) |
| Tongren | Qinghai | 9.64 (6.02, 13.38) | -0.07 (-26.97, 36.73) | 11.75 (-10.65, 39.77) | -1.71 (-23.39, 26.10) |
| Wulan | Qinghai | -1.88 (-3.61, -0.11) | 4.66 (1.06, 8.38) | -6.22 (-22.17, 12.99) | 1.03 (-13.13, 17.50) |
| Xinghai | Qinghai | 5.54 (3.32, 7.81) | 5.70 (-15.66, 32.48) | -1.56 (-18.90, 19.48) | 7.95 (1.14, 15.21) |
| Xunhua | Qinghai | 4.29 (2.22, 6.39) | 7.73 (-17.63, 40.90) | -0.70 (-11.45, 11.36) | 0.32 (-4.65, 5.54) |
| Yushu | Qinghai | 18.45 (9.84, 27.73) | 84.19 (0.56, 237.38) | 6.11 (-6.61, 20.55) | 4.21 (-12.77, 24.50) |
| Zaduo | Qinghai | 21.41 (10.69, 33.18) | 30.42 (-60.52, 330.84) | 9.50 (-12.26, 36.65) | 12.19 (-4.85, 32.29) |
| Zeku | Qinghai | 13.13 (10.65, 15.67) | 4.13 (-9.05, 19.22) | 22.15 (16.04, 28.59) | 9.05 (3.67, 14.70) |
| Zhiduo | Qinghai | 29.97 (18.39, 42.69) | 69.26 (-51.54, 491.23) | 21.02 (3.10, 42.06) | 3.55 (-17.90, 30.62) |
| Ansai | Shaanxi | -1.77 (-4.22, 0.74) | -0.64 (-29.23, 39.50) | -11.55 (-17.14, -5.57) | 2.79 (-2.83, 8.73) |
| Baihe | Shaanxi | -5.16 (-7.27, -3.00) | -8.50 (-25.40, 12.23) | 2.90 (-13.03, 21.76) | -11.91 (-20.91, -1.88) |
| Baishui | Shaanxi | -2.92 (-4.56, -1.26) | -3.96 (-25.24, 23.38) | -3.34 (-4.47, -2.20) | -0.54 (-8.14, 7.70) |
| Binzhou | Shaanxi | -4.71 (-6.70, -2.68) | -13.89 (-22.02, -4.92) | -0.58 (-6.75, 6.00) | 3.45 (-10.83, 20.02) |
| Changwu | Shaanxi | -2.66 (-5.35, 0.11) | -10.43 (-25.68, 7.95) | -4.87 (-11.57, 2.33) | 15.27 (-4.62, 39.32) |
| Chengcheng | Shaanxi | -3.98 (-5.88, -2.05) | -10.05 (-20.11, 1.27) | -4.38 (-17.95, 11.43) | -7.01 (-21.24, 9.79) |
| Chenggu | Shaanxi | -5.74 (-7.28, -4.18) | -11.36 (-13.16, -9.52) | -8.94 (-23.51, 8.40) | -9.94 (-19.94, 1.31) |
| Chunhua | Shaanxi | -4.36 (-6.77, -1.88) | -5.50 (-22.40, 15.09) | -7.13 (-26.18, 16.83) | 6.56 (-8.37, 23.91) |
| Dali | Shaanxi | -4.59 (-6.26, -2.90) | -14.08 (-24.83, -1.78) | -5.86 (-14.52, 3.68) | -1.47 (-7.59, 5.05) |
| Danfeng | Shaanxi | -2.64 (-5.38, 0.18) | -4.34 (-21.00, 15.85) | -5.71 (-19.08, 9.87) | 17.05 (8.81, 25.91) |
| Dingbian | Shaanxi | -5.75 (-8.21, -3.22) | -4.94 (-27.84, 25.21) | 0.64 (-5.56, 7.25) | 0.23 (-7.09, 8.13) |
| Feng | Shaanxi | -5.40 (-8.11, -2.61) | -2.75 (-20.00, 18.20) | -13.58 (-33.49, 12.30) | -3.70 (-16.34, 10.84) |
| Fengxiang | Shaanxi | -4.90 (-6.49, -3.28) | 2.73 (-13.41, 21.87) | -6.49 (-12.99, 0.50) | -8.00 (-16.67, 1.57) |
| Foping | Shaanxi | -0.51 (-4.30, 3.44) | -8.41 (-13.97, -2.50) | 17.23 (-9.70, 52.20) | -14.96 (-40.42, 21.39) |
| Fu | Shaanxi | -3.34 (-6.16, -0.44) | 12.10 (-4.43, 31.50) | -8.93 (-24.07, 9.22) | -11.28 (-18.58, -3.33) |
| Fufeng | Shaanxi | -4.10 (-5.49, -2.68) | -3.60 (-10.37, 3.69) | -6.95 (-14.16, 0.86) | 5.62 (-3.99, 16.19) |
| Fugu | Shaanxi | -0.98 (-5.01, 3.22) | -13.38 (-36.62, 18.38) | 21.04 (-4.13, 52.82) | -16.57 (-21.49, -11.33) |
| Fuping | Shaanxi | -3.59 (-5.30, -1.85) | -4.75 (-20.59, 14.25) | -2.26 (-19.13, 18.12) | -1.02 (-7.64, 6.08) |
| Ganquan | Shaanxi | -4.08 (-6.71, -1.36) | -9.45 (-31.86, 20.33) | -11.29 (-22.28, 1.26) | 0.01 (-21.60, 27.58) |
| Gaoling | Shaanxi | -5.52 (-9.02, -1.90) | -15.24 (-38.22, 16.29) | -12.12 (-17.44, -6.45) | 17.03 (0.40, 36.41) |
| Hancheng | Shaanxi | -3.63 (-4.62, -2.62) | -2.56 (-13.67, 9.97) | -4.82 (-6.90, -2.69) | -4.92 (-7.66, -2.10) |
| Hanyin | Shaanxi | -5.31 (-7.86, -2.69) | -16.91 (-39.66, 14.43) | -2.47 (-15.28, 12.29) | -8.56 (-12.85, -4.07) |
| Hengshan | Shaanxi | 0.67 (-1.60, 3.00) | 0.89 (-12.14, 15.85) | 0.97 (-11.42, 15.08) | -12.36 (-26.45, 4.43) |
| Heyang | Shaanxi | -2.65 (-4.85, -0.39) | -7.44 (-26.80, 17.05) | -0.71 (-17.49, 19.48) | -6.18 (-14.26, 2.66) |
| Huangling | Shaanxi | -0.91 (-3.33, 1.57) | 1.91 (-11.25, 17.02) | -9.37 (-26.13, 11.19) | 1.82 (-14.18, 20.81) |
| Huanglong | Shaanxi | -5.63 (-7.60, -3.61) | -7.69 (-20.55, 7.25) | -10.79 (-20.35, -0.09) | -1.53 (-21.97, 24.27) |
| Huayin | Shaanxi | -4.14 (-5.93, -2.31) | -9.01 (-25.20, 10.70) | 1.24 (-11.14, 15.34) | -4.05 (-16.47, 10.20) |
| Huazhou | Shaanxi | -2.81 (-5.22, -0.35) | -10.90 (-25.39, 6.41) | 1.37 (-8.42, 12.20) | -12.06 (-25.83, 4.28) |
| Huyi | Shaanxi | -1.27 (-2.74, 0.22) | -1.47 (-16.52, 16.29) | 2.03 (-9.07, 14.50) | 1.34 (-6.89, 10.30) |
| Jia | Shaanxi | 1.53 (-0.85, 3.96) | -1.94 (-15.94, 14.39) | 5.70 (0.30, 11.38) | -3.46 (-16.25, 11.28) |
| Jingbian | Shaanxi | 4.15 (1.84, 6.50) | -0.48 (-19.11, 22.44) | -5.03 (-19.62, 12.20) | -1.74 (-10.37, 7.73) |
| Jingyang | Shaanxi | -0.30 (-2.78, 2.25) | -12.41 (-26.64, 4.58) | 0.11 (-19.75, 24.89) | 0.51 (-12.58, 15.56) |
| Langao | Shaanxi | -0.20 (-3.65, 3.37) | 3.66 (-6.46, 14.88) | 11.23 (-8.39, 35.06) | -19.79 (-34.44, -1.86) |
| Lantian | Shaanxi | 0.60 (-0.94, 2.17) | 3.81 (-6.01, 14.65) | 5.72 (-6.98, 20.15) | -0.86 (-4.29, 2.70) |
| Linyou | Shaanxi | -0.67 (-2.99, 1.70) | -2.75 (-28.24, 31.78) | 0.32 (-9.34, 11.01) | -8.85 (-18.98, 2.54) |
| Liquan | Shaanxi | -4.91 (-7.32, -2.43) | -14.88 (-29.52, 2.81) | 2.57 (-13.30, 21.35) | 5.85 (-2.47, 14.87) |
| Liuba | Shaanxi | -8.95 (-13.82, -3.79) | 3.61 (-20.76, 35.47) | 0.56 (-29.14, 42.70) | 4.92 (-39.10, 80.76) |
| Long | Shaanxi | -5.32 (-7.12, -3.48) | -4.48 (-18.70, 12.24) | -3.72 (-18.86, 14.25) | -5.66 (-20.99, 12.65) |
| Lueyang | Shaanxi | -6.25 (-8.35, -4.12) | -8.58 (-20.70, 5.38) | -2.74 (-18.67, 16.31) | -7.17 (-21.26, 9.45) |
| Luochuan | Shaanxi | 0.48 (-2.70, 3.77) | -11.49 (-34.84, 20.23) | -5.73 (-22.34, 14.43) | 4.87 (-10.86, 23.38) |
| Luonan | Shaanxi | -1.69 (-5.32, 2.07) | -19.95 (-42.30, 11.06) | 6.03 (-20.03, 40.58) | 0.72 (-12.44, 15.86) |
| Mei | Shaanxi | -3.88 (-6.68, -1.01) | -12.02 (-39.65, 28.26) | 0.41 (-8.60, 10.31) | -3.79 (-21.31, 17.64) |
| Mian | Shaanxi | -5.89 (-8.23, -3.50) | -12.68 (-23.21, -0.70) | 4.08 (-15.98, 28.92) | -8.99 (-17.39, 0.27) |
| Mizhi | Shaanxi | -4.34 (-6.44, -2.21) | -9.63 (-23.47, 6.70) | 1.43 (-4.08, 7.25) | -7.90 (-24.62, 12.51) |
| Nanzheng | Shaanxi | -7.13 (-8.23, -6.02) | -5.18 (-11.30, 1.36) | -6.97 (-9.01, -4.89) | -11.24 (-20.07, -1.44) |
| Ningqiang | Shaanxi | -6.07 (-8.19, -3.90) | -12.28 (-17.03, -7.27) | 4.29 (-14.92, 27.83) | -11.72 (-24.49, 3.21) |
| Ningshan | Shaanxi | -0.08 (-2.41, 2.31) | 0.83 (-19.95, 26.99) | 9.27 (-0.81, 20.39) | 9.75 (-5.32, 27.22) |
| Pingli | Shaanxi | -0.01 (-2.94, 3.01) | 6.76 (-9.64, 26.14) | 4.10 (-18.74, 33.38) | -5.74 (-21.85, 13.70) |
| Pucheng | Shaanxi | -3.59 (-5.13, -2.02) | 1.10 (-17.55, 23.98) | -2.97 (-11.84, 6.80) | -4.82 (-13.47, 4.69) |
| Qian | Shaanxi | -2.34 (-3.65, -1.01) | -6.56 (-10.10, -2.88) | -4.73 (-17.14, 9.54) | -0.77 (-12.76, 12.87) |
| Qianyang | Shaanxi | -4.85 (-6.78, -2.89) | -12.08 (-21.57, -1.44) | 2.80 (-11.16, 18.97) | -9.55 (-22.69, 5.82) |
| Qingjian | Shaanxi | 1.25 (-1.37, 3.94) | -1.05 (-9.11, 7.72) | -14.47 (-31.39, 6.63) | -2.50 (-13.72, 10.18) |
| Qishan | Shaanxi | -2.58 (-4.36, -0.77) | -2.67 (-9.69, 4.90) | -4.33 (-13.79, 6.17) | -0.57 (-22.19, 27.07) |
| Sanyuan | Shaanxi | -8.29 (-11.15, -5.35) | -2.01 (-26.97, 31.49) | -5.11 (-21.91, 15.32) | 7.67 (-3.34, 19.92) |
| Shangnan | Shaanxi | -5.54 (-9.06, -1.88) | -18.86 (-26.02, -11.00) | -1.39 (-20.70, 22.62) | 4.41 (-10.49, 21.79) |
| Shanyang | Shaanxi | -0.10 (-3.32, 3.22) | -13.56 (-22.06, -4.13) | -3.00 (-10.50, 5.13) | 3.92 (-3.47, 11.87) |
| Shenmu | Shaanxi | -4.63 (-6.33, -2.91) | 5.44 (-2.88, 14.47) | -3.09 (-11.41, 6.02) | -4.79 (-11.53, 2.45) |
| Shiquan | Shaanxi | -1.60 (-5.48, 2.43) | -8.47 (-25.42, 12.34) | 13.18 (4.47, 22.63) | -22.99 (-38.78, -3.13) |
| Suide | Shaanxi | -5.81 (-8.41, -3.14) | -14.08 (-39.51, 22.03) | -10.36 (-17.61, -2.48) | 3.17 (-7.15, 14.62) |
| Taibai | Shaanxi | -4.39 (-6.60, -2.13) | -5.78 (-9.49, -1.93) | -3.18 (-16.49, 12.26) | -3.08 (-22.67, 21.46) |
| Tongguan | Shaanxi | -4.01 (-6.57, -1.38) | -2.38 (-10.72, 6.74) | -6.19 (-32.01, 29.44) | 3.41 (-12.22, 21.81) |
| Wubao | Shaanxi | -2.98 (-5.62, -0.26) | -9.98 (-32.28, 19.64) | -5.27 (-21.26, 13.97) | -3.01 (-18.11, 14.88) |
| Wugong | Shaanxi | -6.25 (-8.46, -3.99) | -14.66 (-30.45, 4.71) | -1.59 (-7.44, 4.62) | 6.50 (-3.93, 18.07) |
| Wuqi | Shaanxi | -7.52 (-10.37, -4.57) | -8.69 (-22.73, 7.89) | -12.84 (-32.92, 13.26) | -12.58 (-24.10, 0.69) |
| Xingping | Shaanxi | -2.81 (-4.29, -1.31) | -5.56 (-15.77, 5.88) | -0.62 (-15.61, 17.04) | -0.96 (-11.21, 10.47) |
| Xixiang | Shaanxi | -1.69 (-3.43, 0.09) | -4.41 (-12.99, 5.02) | -0.60 (-4.68, 3.65) | -7.86 (-21.25, 7.80) |
| Xunyang | Shaanxi | -4.46 (-6.50, -2.37) | -9.50 (-14.42, -4.30) | -1.43 (-12.16, 10.61) | 0.47 (-6.85, 8.36) |
| Xunyi | Shaanxi | -2.78 (-4.87, -0.65) | -8.30 (-20.04, 5.16) | 6.49 (-4.04, 18.18) | -3.48 (-22.43, 20.09) |
| Yanchang | Shaanxi | -0.03 (-1.72, 1.68) | -4.74 (-22.85, 17.63) | 3.79 (-6.41, 15.10) | 2.73 (-5.04, 11.14) |
| Yanchuan | Shaanxi | 1.25 (-1.96, 4.57) | -2.47 (-32.41, 40.75) | -12.5 (-22.25, -1.52) | 7.14 (-11.13, 29.18) |
| Yang | Shaanxi | -5.13 (-7.87, -2.31) | -16.31 (-24.03, -7.82) | -3.61 (-17.28, 12.32) | -1.35 (-9.87, 7.97) |
| Yichuan | Shaanxi | -2.69 (-4.82, -0.51) | 1.17 (-9.35, 12.92) | -3.46 (-14.75, 9.33) | 14.05 (5.74, 23.01) |
| Yijun | Shaanxi | -5.38 (-7.93, -2.76) | -4.46 (-12.03, 3.77) | -8.60 (-17.82, 1.65) | 5.34 (-4.53, 16.23) |
| Yongshou | Shaanxi | -0.81 (-3.32, 1.77) | -9.32 (-31.77, 20.53) | -1.55 (-21.01, 22.71) | 5.43 (-1.03, 12.31) |
| Zhashui | Shaanxi | -1.86 (-4.32, 0.66) | 0.49 (-16.58, 21.05) | 0.11 (-7.86, 8.76) | 14.52 (-2.11, 33.98) |
| Zhenan | Shaanxi | -2.06 (-4.87, 0.82) | -9.76 (-23.08, 5.85) | -1.36 (-15.01, 14.49) | 9.48 (-9.27, 32.10) |
| Zhenping | Shaanxi | 3.03 (1.17, 4.92) | 1.94 (-14.35, 21.32) | 2.54 (-7.94, 14.21) | -1.51 (-13.8, 12.53) |
| Zhenba | Shaanxi | -8.10 (-11.57, -4.49) | -6.98 (-15.00, 1.80) | -8.24 (-18.36, 3.12) | 5.82 (-14.93, 31.63) |
| Zhidan | Shaanxi | -1.09 (-4.23, 2.16) | 20.81 (14.07, 27.95) | -7.79 (-23.10, 10.56) | -3.43 (-22.87, 20.93) |
| Zhouzhi | Shaanxi | -1.40 (-3.60, 0.85) | -12.19 (-25.37, 3.31) | -2.95 (-13.83, 9.31) | 10.08 (7.06, 13.19) |
| Zichang | Shaanxi | -2.10 (-4.53, 0.39) | 7.72 (-0.68, 16.82) | -10.91 (-26.33, 7.73) | 1.95 (-8.22, 13.26) |
| Ziyang | Shaanxi | -3.99 (-6.75, -1.14) | -0.32 (-18.12, 21.36) | -1.11 (-14.08, 13.81) | -11.65 (-19.9, -2.56) |
| Zizhou | Shaanxi | 0.65 (-1.99, 3.35) | -0.40 (-15.31, 17.12) | 5.54 (-4.89, 17.11) | -4.31 (-23.31, 19.39) |
| Anqiu | Shandong | -3.12 (-4.80, -1.42) | -4.26 (-16.56, 9.86) | 1.57 (-2.22, 5.51) | -13.42 (-20.55, -5.64) |
| Boxing | Shandong | -9.97 (-13.27, -6.54) | -1.63 (-9.01, 6.36) | -25.87 (-38.96, -9.97) | 8.60 (-11.20, 32.81) |
| Cao | Shandong | -4.82 (-7.06, -2.52) | 11.60 (-5.80, 32.21) | -5.53 (-19.39, 10.71) | -7.24 (-13.83, -0.14) |
| Changdao | Shandong | -0.96 (-6.32, 4.71) | -21.65 (-53.56, 32.18) | 10.13 (-6.24, 29.35) | -5.65 (-43.84, 58.52) |
| Changle | Shandong | -5.65 (-8.65, -2.56) | 7.21 (0.89, 13.94) | -19.60 (-36.63, 2.00) | 8.74 (4.04, 13.66) |
| Changyi | Shandong | -1.77 (-3.71, 0.21) | -5.79 (-25.00, 18.33) | 1.17 (-13.13, 17.84) | 2.18 (0.17, 4.22) |
| Chengwu | Shandong | -3.41 (-4.73, -2.06) | 1.41 (-8.47, 12.35) | -3.10 (-4.77, -1.40) | -9.55 (-14.71, -4.08) |
| Chiping | Shandong | -2.03 (-3.59, -0.44) | 1.48 (-12.13, 17.20) | 2.02 (-4.72, 9.25) | -9.82 (-18.26, -0.50) |
| Dan | Shandong | -3.31 (-5.03, -1.56) | 6.05 (-3.89, 17.01) | -4.09 (-9.01, 1.09) | -8.53 (-11.55, -5.41) |
| Dingtao | Shandong | -1.82 (-3.67, 0.07) | -2.05 (-16.00, 14.23) | 8.23 (-3.72, 21.66) | -7.47 (-17.78, 4.13) |
| Donga | Shandong | -0.31 (-3.29, 2.77) | 20.85 (5.51, 38.42) | 2.59 (-15.20, 24.12) | -7.44 (-23.57, 12.08) |
| Dongming | Shandong | -1.96 (-3.56, -0.33) | -1.03 (-10.25, 9.12) | 6.86 (-7.03, 22.83) | -1.31 (-11.14, 9.62) |
| Dongping | Shandong | -6.06 (-9.50, -2.49) | 11.7 (5.27, 18.52) | -4.24 (-17.05, 10.55) | -24.73 (-31.97, -16.72) |
| Fei | Shandong | -5.55 (-7.71, -3.33) | 2.95 (-4.20, 10.64) | -6.30 (-29.31, 24.20) | -3.16 (-16.91, 12.86) |
| Feicheng | Shandong | -6.36 (-8.62, -4.04) | -3.92 (-15.79, 9.62) | 2.72 (1.18, 4.30) | -1.11 (-20.84, 23.55) |
| Gaomi | Shandong | -2.77 (-4.56, -0.95) | -6.78 (-14.55, 1.70) | 2.72 (-13.39, 21.84) | -4.68 (-21.27, 15.41) |
| Gaoqing | Shandong | -3.93 (-6.95, -0.80) | 1.95 (-5.49, 9.97) | -0.74 (-8.80, 8.04) | -21.84 (-33.70, -7.86) |
| Gaotang | Shandong | -1.28 (-2.37, -0.18) | 4.86 (3.40, 6.34) | -0.52 (-12.55, 13.17) | -1.37 (-5.43, 2.87) |
| Guan | Shandong | -3.17 (-4.65, -1.66) | -0.30 (-8.38, 8.50) | 0.71 (-16.13, 20.94) | -2.66 (-7.50, 2.43) |
| Guangrao | Shandong | -1.05 (-2.73, 0.65) | 2.23 (-9.96, 16.08) | -4.74 (-21.87, 16.16) | -4.65 (-12.87, 4.35) |
| Haiyang | Shandong | -2.93 (-4.89, -0.93) | 6.52 (-3.86, 18.03) | -1.69 (-12.64, 10.64) | -9.10 (-20.71, 4.21) |
| Huantai | Shandong | -5.68 (-6.46, -4.90) | -8.31 (-15.49, -0.52) | -2.23 (-2.91, -1.56) | -7.41 (-9.11, -5.67) |
| Huimin | Shandong | -5.11 (-7.99, -2.14) | 5.75 (0.42, 11.37) | -19.52 (-30.36, -7.00) | -0.95 (-25.96, 32.51) |
| Jiaozhou | Shandong | -2.01 (-4.11, 0.13) | -7.09 (-15.74, 2.44) | -12.84 (-25.23, 1.60) | 7.54 (4.19, 11.01) |
| Jiaxiang | Shandong | -1.94 (-3.08, -0.78) | 0.68 (-9.39, 11.88) | -1.72 (-13.15, 11.22) | -0.28 (-6.41, 6.25) |
| Jimo | Shandong | -2.32 (-3.79, -0.82) | -2.93 (-14.65, 10.41) | 2.90 (-2.55, 8.66) | -2.04 (-13.64, 11.13) |
| Jinxiang | Shandong | -5.94 (-9.20, -2.56) | -2.96 (-19.65, 17.19) | -18.76 (-30.33, -5.26) | 9.33 (-15.98, 42.25) |
| Jiyang | Shandong | -4.44 (-6.03, -2.83) | 1.55 (-8.81, 13.08) | 1.22 (-3.59, 6.27) | -12.8 (-18.12, -7.13) |
| Ju | Shandong | -4.00 (-5.72, -2.26) | -9.36 (-18.66, 1.02) | 0.77 (-16.87, 22.15) | -1.08 (-11.29, 10.30) |
| Juancheng | Shandong | -3.30 (-4.96, -1.62) | 1.85 (-10.18, 15.49) | 5.48 (-6.47, 18.96) | -5.05 (-10.48, 0.71) |
| Junan | Shandong | -2.15 (-4.70, 0.47) | 7.75 (-6.68, 24.41) | -3.29 (-13.61, 8.26) | -13.93 (-22.20, -4.79) |
| Juye | Shandong | -4.82 (-6.14, -3.49) | -1.13 (-8.35, 6.67) | 1.00 (-7.77, 10.59) | -9.46 (-12.15, -6.70) |
| Kenli | Shandong | -4.47 (-5.61, -3.33) | -2.28 (-9.08, 5.02) | -2.63 (-14.31, 10.66) | -5.76 (-10.35, -0.92) |
| Laixi | Shandong | 0.34 (-1.42, 2.13) | 7.79 (-8.60, 27.12) | -0.54 (-10.67, 10.75) | -0.29 (-11.13, 11.88) |
| Laiyang | Shandong | -6.62 (-8.76, -4.44) | 5.84 (-0.89, 13.04) | -8.61 (-15.63, -1.00) | -9.58 (-24.23, 7.91) |
| Laizhou | Shandong | -4.78 (-6.25, -3.29) | -6.85 (-14.66, 1.66) | -3.98 (-6.81, -1.05) | 4.72 (-1.62, 11.48) |
| Lanling | Shandong | -3.35 (-5.68, -0.97) | 5.23 (1.03, 9.61) | -1.41 (-7.52, 5.10) | -15.64 (-22.31, -8.40) |
| Leling | Shandong | -4.05 (-6.10, -1.95) | 4.54 (-15.64, 29.54) | -8.74 (-14.94, -2.09) | 2.24 (-6.79, 12.14) |
| Liangshan | Shandong | -3.64 (-5.90, -1.33) | -5.47 (-24.39, 18.18) | -7.76 (-12.71, -2.53) | -11.76 (-19.16, -3.67) |
| Lijin | Shandong | -7.39 (-10.13, -4.57) | -13.00 (-29.30, 7.05) | 7.78 (-9.50, 28.35) | -9.81 (-19.30, 0.78) |
| Lingcheng | Shandong | -6.98 (-8.77, -5.16) | -16.28 (-18.75, -13.74) | -4.19 (-12.64, 5.08) | 3.74 (-2.08, 9.91) |
| Linqing | Shandong | -2.21 (-4.82, 0.47) | -0.30 (-7.58, 7.56) | 16.92 (-0.10, 36.85) | -2.88 (-15.77, 11.98) |
| Linqu | Shandong | -4.16 (-6.47, -1.79) | -3.57 (-11.69, 5.29) | -3.07 (-13.88, 9.09) | -6.35 (-15.02, 3.21) |
| Linshu | Shandong | -4.64 (-6.58, -2.65) | -6.89 (-24.68, 15.09) | -3.98 (-11.23, 3.87) | -4.85 (-11.35, 2.13) |
| Linyi | Shandong | -4.86 (-6.35, -3.34) | -6.57 (-20.13, 9.31) | -8.46 (-12.75, -3.96) | -5.67 (-15.87, 5.76) |
| Longkou | Shandong | -1.79 (-3.44, -0.10) | 6.20 (-3.34, 16.69) | -2.30 (-10.21, 6.30) | 3.70 (-11.96, 22.15) |
| Mengyin | Shandong | -1.55 (-3.85, 0.81) | 3.82 (-7.71, 16.79) | 0.90 (-11.41, 14.91) | -9.48 (-19.48, 1.75) |
| Ningjin | Shandong | -4.14 (-6.51, -1.70) | 10.54 (-13.02, 40.47) | -4.27 (-14.12, 6.71) | -1.34 (-11.96, 10.57) |
| Ningyang | Shandong | -7.68 (-10.77, -4.48) | 9.27 (-3.95, 24.31) | -3.77 (-12.72, 6.09) | -23.78 (-32.18, -14.35) |
| Penglai | Shandong | -0.76 (-5.45, 4.16) | 25.21 (-5.95, 66.69) | -16.75 (-37.73, 11.30) | -8.70 (-25.20, 11.43) |
| Pingdu | Shandong | 0.10 (-1.06, 1.28) | 1.99 (-3.44, 7.74) | 0.37 (-4.88, 5.92) | 0.03 (-14.20, 16.63) |
| Pingyi | Shandong | -0.86 (-3.73, 2.09) | 13.11 (-8.07, 39.16) | -2.77 (-12.05, 7.48) | -8.49 (-12.87, -3.89) |
| Pingyin | Shandong | -5.22 (-6.21, -4.22) | -2.98 (-6.63, 0.82) | 0.79 (-3.76, 5.56) | -3.11 (-9.89, 4.18) |
| Pingyuan | Shandong | -4.02 (-5.66, -2.36) | 4.26 (-1.71, 10.60) | -2.94 (-12.13, 7.22) | -12.69 (-20.15, -4.52) |
| Qihe | Shandong | -3.49 (-5.11, -1.85) | -8.74 (-14.90, -2.12) | -4.56 (-13.11, 4.84) | 7.77 (-0.60, 16.85) |
| Qingyun | Shandong | -5.99 (-9.46, -2.38) | -0.64 (-8.64, 8.06) | -22.97 (-31.69, -13.14) | 3.72 (-14.94, 26.48) |
| Qingzhou | Shandong | -6.78 (-8.89, -4.61) | -2.70 (-17.89, 15.30) | -10.74 (-20.23, -0.12) | -3.00 (-9.95, 4.49) |
| Qixia | Shandong | -0.74 (-3.51, 2.11) | 12.29 (-6.01, 34.15) | 0.43 (-4.49, 5.61) | -9.67 (-20.04, 2.05) |
| Qufu | Shandong | -3.37 (-4.35, -2.38) | -2.45 (-11.56, 7.60) | -0.49 (-9.92, 9.92) | -3.40 (-9.19, 2.76) |
| Rongcheng | Shandong | -0.84 (-2.60, 0.96) | 3.62 (-3.80, 11.6) | -1.05 (-17.84, 19.17) | 8.45 (0.16, 17.42) |
| Rushan | Shandong | -5.25 (-7.44, -3.01) | 0.51 (-15.46, 19.51) | -11.10 (-22.35, 1.77) | 8.94 (4.73, 13.31) |
| Shanghe | Shandong | -4.87 (-6.91, -2.80) | 7.40 (-8.07, 25.47) | -6.33 (-22.87, 13.75) | -7.78 (-12.83, -2.44) |
| Shen | Shandong | -2.95 (-4.16, -1.73) | 0.16 (-8.26, 9.36) | -8.67 (-13.50, -3.57) | -3.24 (-6.39, 0.02) |
| Shouguang | Shandong | -2.18 (-4.23, -0.09) | -5.91 (-20.27, 11.03) | 3.31 (-15.83, 26.79) | -2.75 (-12.83, 8.49) |
| Sishui | Shandong | -4.41 (-6.75, -2.02) | 4.30 (-7.80, 17.99) | -12.01 (-19.20, -4.19) | 9.27 (-7.07, 28.47) |
| Tancheng | Shandong | -3.96 (-5.98, -1.89) | -0.19 (-16.13, 18.77) | -2.78 (-8.33, 3.10) | -12.67 (-20.44, -4.15) |
| Tengzhou | Shandong | -2.73 (-4.39, -1.03) | -1.31 (-9.92, 8.11) | 10.01 (2.04, 18.59) | -6.14 (-9.18, -3.00) |
| Weishan | Shandong | -2.87 (-4.49, -1.21) | 2.13 (-9.93, 15.80) | -4.37 (-16.04, 8.93) | -6.73 (-14.25, 1.46) |
| Wendeng | Shandong | -0.07 (-1.42, 1.30) | -4.14 (-14.05, 6.91) | -4.46 (-15.32, 7.80) | -1.18 (-7.38, 5.44) |
| Wenshang | Shandong | -2.08 (-3.07, -1.08) | 0.59 (-5.10, 6.62) | -2.66 (-13.93, 10.09) | -4.36 (-7.40, -1.21) |
| Wucheng | Shandong | -4.05 (-5.55, -2.53) | -9.16 (-25.76, 11.17) | -4.76 (-10.86, 1.75) | -0.43 (-7.03, 6.65) |
| Wudi | Shandong | -5.13 (-6.28, -3.97) | -4.09 (-12.28, 4.87) | -4.74 (-14.92, 6.65) | -3.98 (-15.36, 8.93) |
| Wulian | Shandong | -5.39 (-7.74, -2.97) | 3.26 (-0.32, 6.97) | -2.66 (-14.98, 11.45) | -19.77 (-27.28, -11.49) |
| Xiajin | Shandong | -5.60 (-8.05, -3.08) | -3.38 (-13.43, 7.84) | 3.28 (-5.64, 13.04) | -4.47 (-15.10, 7.50) |
| Xintai | Shandong | -5.59 (-7.32, -3.82) | -0.47 (-5.71, 5.07) | -1.01 (-15.09, 15.40) | -3.75 (-12.73, 6.16) |
| Yanggu | Shandong | -2.63 (-3.46, -1.78) | 0.84 (-8.28, 10.86) | -1.68 (-3.62, 0.30) | -2.80 (-7.74, 2.40) |
| Yangxin | Shandong | -3.35 (-5.19, -1.47) | -1.03 (-13.13, 12.75) | -10.51 (-25.81, 7.96) | -4.49 (-13.44, 5.38) |
| Yanzhou | Shandong | -6.52 (-8.62, -4.38) | -4.00 (-25.93, 24.43) | -12.65 (-21.79, -2.45) | -6.30 (-22.78, 13.72) |
| Yinan | Shandong | -2.88 (-4.88, -0.85) | -1.41 (-22.13, 24.82) | -2.91 (-9.88, 4.60) | -7.92 (-12.45, -3.16) |
| Yishui | Shandong | -2.29 (-4.31, -0.22) | 1.46 (-12.34, 17.44) | 2.73 (-7.17, 13.68) | -10.90 (-17.90, -3.30) |
| Yiyuan | Shandong | -1.58 (-3.01, -0.13) | 2.01 (-3.95, 8.33) | -2.46 (-13.71, 10.25) | 3.70 (-7.11, 15.77) |
| Yucheng | Shandong | -5.41 (-6.84, -3.96) | -4.80 (-15.82, 7.65) | 1.28 (-3.08, 5.84) | -3.51 (-17.14, 12.37) |
| Yuncheng | Shandong | -3.99 (-5.58, -2.38) | 3.08 (-3.58, 10.20) | -2.84 (-14.04, 9.83) | 0.48 (-14.64, 18.27) |
| Yutai | Shandong | -2.78 (-5.33, -0.17) | -9.21 (-21.46, 4.95) | -3.63 (-21.83, 18.80) | 4.94 (-9.42, 21.59) |
| Zhangqiu | Shandong | -5.29 (-6.78, -3.78) | 4.12 (-1.13, 9.66) | -5.14 (-12.28, 2.59) | -5.63 (-15.41, 5.27) |
| Zhanhua | Shandong | -3.29 (-4.69, -1.87) | -0.18 (-6.40, 6.46) | -4.09 (-7.45, -0.59) | -6.76 (-24.04, 14.47) |
| Zhaoyuan | Shandong | 1.28 (0.56, 2.01) | 2.33 (-2.64, 7.54) | 2.35 (-6.73, 12.32) | 1.74 (-1.34, 4.90) |
| Zhucheng | Shandong | -3.03 (-3.92, -2.14) | -4.31 (-9.96, 1.68) | -1.51 (-4.34, 1.40) | -6.91 (-15.07, 2.03) |
| Zoucheng | Shandong | -7.00 (-9.58, -4.35) | 4.58 (-4.71, 14.77) | -7.58 (-15.89, 1.55) | -12.14 (-25.69, 3.88) |
| Zouping | Shandong | -8.21 (-9.76, -6.63) | 1.77 (-7.22, 11.63) | -8.81 (-20.97, 5.21) | -6.24 (-11.73, -0.40) |
| Anze | Shanxi | -11.11 (-13.14, -9.03) | -4.60 (-21.02, 15.22) | -14.19 (-18.73, -9.40) | -8.55 (-27.58, 15.47) |
| Baode | Shanxi | -6.76 (-8.50, -4.99) | -7.38 (-19.35, 6.38) | -5.58 (-19.05, 10.14) | -2.21 (-15.23, 12.81) |
| Dai | Shanxi | -6.80 (-8.17, -5.41) | -4.02 (-13.27, 6.21) | -12.08 (-18.59, -5.05) | -7.20 (-21.20, 9.29) |
| Daning | Shanxi | -4.85 (-7.27, -2.36) | 7.36 (-8.09, 25.40) | -14.56 (-18.98, -9.91) | 1.56 (-16.83, 24.02) |
| Dingxiang | Shanxi | -4.11 (-6.70, -1.46) | -1.18 (-15.35, 15.36) | -16.36 (-35.40, 8.31) | -11.70 (-19.98, -2.56) |
| Fangshan | Shanxi | -6.77 (-10.27, -3.13) | -7.42 (-19.80, 6.88) | -18.38 (-38.92, 9.07) | 17.00 (-5.23, 44.43) |
| Fanzhi | Shanxi | -3.80 (-5.20, -2.38) | -9.23 (-20.99, 4.28) | -4.22 (-9.81, 1.72) | -7.36 (-16.51, 2.80) |
| Fenxi | Shanxi | -2.29 (-5.90, 1.46) | 22.36 (8.33, 38.21) | -13.10 (-20.24, -5.33) | 2.52 (-9.68, 16.38) |
| Fenyang | Shanxi | -8.55 (-11.67, -5.33) | -1.97 (-5.95, 2.18) | -6.56 (-14.87, 2.57) | -12.05 (-28.09, 7.58) |
| Fushan | Shanxi | -9.67 (-13.39, -5.79) | 4.52 (-10.45, 21.99) | -16.32 (-26.80, -4.32) | 2.35 (-13.42, 20.99) |
| Gaoping | Shanxi | -5.95 (-7.96, -3.90) | -7.57 (-19.92, 6.67) | -9.92 (-21.31, 3.11) | -15.92 (-26.15, -4.27) |
| Gu | Shanxi | -11.89 (-15.00, -8.67) | -6.87 (-20.91, 9.65) | -13.58 (-24.10, -1.60) | -6.49 (-35.14, 34.81) |
| Guangling | Shanxi | -3.15 (-5.11, -1.15) | -6.38 (-24.66, 16.34) | -11.41 (-19.03, -3.08) | -0.55 (-10.16, 10.08) |
| Gujiao | Shanxi | -10.16 (-13.9, -6.25) | 4.61 (-19.36, 35.72) | -11.21 (-38.43, 28.03) | -20.9 (-34.71, -4.16) |
| Hejin | Shanxi | -6.19 (-9.34, -2.93) | 3.64 (-26.26, 45.66) | -7.51 (-17.38, 3.55) | -11.16 (-24.64, 4.73) |
| Hequ | Shanxi | -5.47 (-7.60, -3.29) | -13.57 (-32.67, 10.96) | -9.54 (-14.34, -4.47) | 2.57 (-2.28, 7.67) |
| Heshun | Shanxi | -8.92 (-11.54, -6.21) | 1.57 (-11.34, 16.36) | -15.25 (-25.47, -3.63) | -10.23 (-31.30, 17.31) |
| Hongtong | Shanxi | -3.05 (-4.34, -1.74) | -5.41 (-16.21, 6.79) | -3.12 (-14.78, 10.14) | 1.12 (-5.33, 8.00) |
| Houma | Shanxi | -9.76 (-12.67, -6.76) | 3.38 (-12.77, 22.53) | -22.45 (-32.90, -10.36) | -3.18 (-16.99, 12.92) |
| Huairen | Shanxi | -8.77 (-12.35, -5.05) | -8.91 (-24.20, 9.45) | -17.90 (-42.38, 16.98) | -0.91 (-14.14, 14.35) |
| Huguan | Shanxi | -5.96 (-8.62, -3.21) | 2.52 (-18.82, 29.47) | -8.39 (-20.25, 5.24) | 7.16 (-11.02, 29.05) |
| Hunyuan | Shanxi | -4.90 (-7.18, -2.55) | -16.04 (-30.07, 0.80) | -8.65 (-17.26, 0.86) | -0.55 (-11.44, 11.69) |
| Huozhou | Shanxi | -4.23 (-7.53, -0.81) | 18.73 (-4.14, 47.05) | -12.27 (-24.16, 1.49) | -3.33 (-13.39, 7.91) |
| Ji | Shanxi | -6.78 (-8.67, -4.85) | 7.65 (1.47, 14.21) | -9.45 (-12.72, -6.06) | -6.28 (-14.98, 3.32) |
| Jiaocheng | Shanxi | -12.84 (-15.06, -10.56) | -16.47 (-35.65, 8.43) | -11.05 (-26.13, 7.11) | -10.34 (-29.86, 14.61) |
| Jiaokou | Shanxi | -4.36 (-8.21, -0.36) | 19.81 (-22.43, 85.07) | -14.46 (-22.88, -5.11) | -2.44 (-14.73, 11.64) |
| Jiexiu | Shanxi | -12.74 (-15.68, -9.69) | -7.91 (-17.26, 2.50) | -23.47 (-45.08, 6.65) | -6.72 (-17.06, 4.91) |
| Jingle | Shanxi | -8.97 (-10.82, -7.09) | -3.73 (-20.65, 16.79) | -13.81 (-22.08, -4.66) | 0.38 (-8.17, 9.73) |
| Jishan | Shanxi | -7.11 (-10.87, -3.20) | 12.53 (-2.51, 29.89) | -13.86 (-16.99, -10.62) | -10.23 (-28.02, 11.95) |
| Lan | Shanxi | -4.99 (-7.86, -2.03) | -9.14 (-40.82, 39.49) | -0.09 (-14.48, 16.73) | -5.05 (-18.76, 10.98) |
| Licheng | Shanxi | -4.09 (-6.04, -2.11) | -13.14 (-21.66, -3.68) | -5.59 (-15.41, 5.37) | 5.85 (-8.26, 22.12) |
| Lin | Shanxi | -4.01 (-6.99, -0.93) | -8.04 (-25.27, 13.17) | -15.43 (-37.12, 13.75) | 2.22 (-16.07, 24.50) |
| Lingchuan | Shanxi | -5.60 (-7.46, -3.70) | -4.40 (-17.56, 10.87) | -8.19 (-20.65, 6.23) | -14.48 (-18.47, -10.30) |
| Lingqiu | Shanxi | -4.95 (-6.68, -3.18) | -7.75 (-27.66, 17.64) | -7.67 (-13.66, -1.27) | -2.52 (-14.30, 10.89) |
| Lingshi | Shanxi | -11.01 (-13.88, -8.05) | -1.84 (-13.31, 11.16) | -4.74 (-12.17, 3.31) | -18.12 (-36.27, 5.20) |
| Linyi | Shanxi | -5.79 (-8.35, -3.16) | 1.85 (-15.88, 23.33) | -13.13 (-19.26, -6.53) | -9.14 (-18.92, 1.83) |
| Liulin | Shanxi | -4.96 (-7.00, -2.88) | -6.59 (-18.15, 6.61) | -12.29 (-25.09, 2.71) | 3.02 (-13.59, 22.83) |
| Loufan | Shanxi | -6.17 (-8.91, -3.35) | 10.41 (-4.15, 27.20) | -14.93 (-23.34, -5.60) | -14.35 (-20.30, -7.96) |
| Lucheng | Shanxi | -5.87 (-7.58, -4.13) | -2.45 (-8.07, 3.52) | -7.63 (-16.99, 2.80) | 5.86 (-6.57, 19.95) |
| Ningwu | Shanxi | -5.93 (-8.17, -3.63) | -4.41 (-19.51, 13.53) | 3.76 (-20.38, 35.21) | -8.30 (-14.23, -1.96) |
| Pianguan | Shanxi | -11.15 (-13.71, -8.53) | -17.23 (-31.80, 0.45) | -16.09 (-24.79, -6.39) | 1.78 (-23.61, 35.62) |
| Pingding | Shanxi | -6.08 (-8.23, -3.88) | -16.61 (-31.61, 1.68) | -9.30 (-17.76, 0.04) | 3.29 (-5.05, 12.36) |
| Pinglu | Shanxi | -7.69 (-9.71, -5.62) | -1.00 (-13.39, 13.16) | -6.70 (-11.79, -1.32) | -9.37 (-28.29, 14.54) |
| Pingshun | Shanxi | -6.01 (-8.46, -3.49) | -11.44 (-23.55, 2.60) | -12.45 (-24.09, 0.97) | 10.75 (-4.54, 28.48) |
| Pingyao | Shanxi | -7.35 (-9.95, -4.68) | -2.72 (-21.77, 20.97) | -11.26 (-18.19, -3.74) | -20.33 (-31.99, -6.66) |
| Pu | Shanxi | -9.00 (-11.81, -6.09) | 4.94 (-11.00, 23.74) | -15.67 (-21.03, -9.94) | -13.02 (-33.39, 13.59) |
| Qi | Shanxi | -12.60 (-17.77, -7.10) | -6.72 (-22.61, 12.43) | 3.75 (-13.78, 24.84) | 6.10 (-20.56, 41.71) |
| Qin | Shanxi | -8.37 (-10.75, -5.92) | -2.23 (-10.12, 6.35) | -9.79 (-24.61, 7.95) | -0.11 (-23.15, 29.84) |
| Qingxu | Shanxi | -9.54 (-13.20, -5.73) | -5.68 (-15.76, 5.61) | -4.32 (-11.32, 3.23) | -29.92 (-48.80, -4.09) |
| Qinshui | Shanxi | -11.02 (-13.43, -8.53) | -15.35 (-25.51, -3.81) | -1.50 (-18.62, 19.21) | -24.17 (-27.75, -20.42) |
| Qinyuan | Shanxi | -7.10 (-9.33, -4.82) | 0.57 (-8.55, 10.61) | -13.67 (-29.59, 5.85) | -2.95 (-12.17, 7.25) |
| Quwo | Shanxi | -12.56 (-15.41, -9.61) | -8.85 (-17.11, 0.23) | -17.62 (-29.07, -4.33) | -4.38 (-11.77, 3.64) |
| Ruicheng | Shanxi | -2.96 (-7.21, 1.49) | -7.64 (-51.09, 74.40) | -7.57 (-12.96, -1.85) | -8.68 (-12.12, -5.11) |
| Shangdang | Shanxi | -6.75 (-9.98, -3.40) | -1.35 (-31.78, 42.65) | -10.01 (-24.00, 6.54) | 5.98 (-16.80, 34.98) |
| Shanyin | Shanxi | -4.46 (-6.23, -2.65) | -8.84 (-16.11, -0.95) | -5.19 (-22.80, 16.43) | -8.68 (-20.89, 5.42) |
| Shenchi | Shanxi | -7.86 (-9.18, -6.51) | -3.82 (-14.53, 8.23) | -11.54 (-23.67, 2.51) | -6.32 (-13.79, 1.80) |
| Shilou | Shanxi | -3.36 (-5.57, -1.11) | 7.15 (-10.14, 27.76) | -2.58 (-14.61, 11.14) | -6.82 (-15.53, 2.79) |
| Shouyang | Shanxi | -2.69 (-4.00, -1.35) | -3.20 (-14.19, 9.20) | -2.40 (-14.70, 11.67) | -5.78 (-15.61, 5.19) |
| Taigu | Shanxi | -11.15 (-14.79, -7.36) | -6.17 (-20.36, 10.55) | -9.89 (-19.34, 0.66) | -26.12 (-40.37, -8.45) |
| Tianzhen | Shanxi | -4.21 (-5.75, -2.64) | -5.08 (-17.96, 9.82) | -0.32 (-14.52, 16.23) | -5.98 (-15.70, 4.86) |
| Tunliu | Shanxi | -6.64 (-9.22, -3.99) | 2.96 (-5.64, 12.34) | -10.70 (-19.40, -1.06) | 9.36 (-8.25, 30.35) |
| Wanrong | Shanxi | -2.89 (-5.20, -0.53) | 6.33 (-16.92, 36.08) | -5.14 (-8.50, -1.65) | -4.67 (-9.68, 0.62) |
| Wenshui | Shanxi | -13.37 (-15.73, -10.94) | 3.34 (-4.34, 11.63) | -18.09 (-29.71, -4.54) | -11.85 (-21.14, -1.47) |
| Wenxi | Shanxi | -5.41 (-8.29, -2.44) | 0.23 (-24.43, 32.93) | -14.92 (-21.17, -8.17) | -8.72 (-17.49, 0.99) |
| Wutai | Shanxi | -4.69 (-7.05, -2.27) | 1.93 (-19.39, 28.88) | -7.89 (-17.30, 2.61) | 2.85 (-5.05, 11.40) |
| Wuxiang | Shanxi | -10.28 (-13.63, -6.81) | -6.68 (-20.03, 8.91) | -17.88 (-36.69, 6.52) | 5.11 (-16.80, 32.78) |
| Wuzhai | Shanxi | -9.70 (-12.50, -6.81) | -23.55 (-35.88, -8.85) | -6.96 (-18.40, 6.08) | -9.33 (-30.25, 17.88) |
| Xi | Shanxi | -7.24 (-9.76, -4.65) | -5.75 (-17.44, 7.60) | -8.00 (-22.04, 8.57) | -10.73 (-37.39, 27.28) |
| Xia | Shanxi | -2.35 (-4.26, -0.40) | 2.74 (-15.38, 24.74) | -8.23 (-18.06, 2.79) | -4.41 (-13.35, 5.46) |
| Xiangfen | Shanxi | -11.84 (-15.53, -7.99) | -9.54 (-31.29, 19.11) | -21.97 (-41.98, 4.94) | -1.47 (-20.68, 22.40) |
| Xiangning | Shanxi | -3.19 (-5.33, -1.00) | 6.17 (-11.60, 27.51) | -12.31 (-20.40, -3.40) | 0.71 (-5.76, 7.62) |
| Xiangyuan | Shanxi | -9.07 (-10.45, -7.66) | -11.19 (-20.34, -0.98) | -12.36 (-22.93, -0.34) | -7.20 (-20.74, 8.66) |
| Xiaoyi | Shanxi | -7.99 (-10.27, -5.66) | 0.84 (-19.76, 26.73) | -6.18 (-12.95, 1.11) | -10.13 (-18.15, -1.33) |
| Xing | Shanxi | -3.80 (-6.63, -0.89) | 12.02 (2.37, 22.58) | -2.81 (-15.32, 11.53) | 2.23 (-14.28, 21.92) |
| Xinjiang | Shanxi | -5.23 (-8.41, -1.94) | 1.62 (-17.51, 25.18) | -7.91 (-16.82, 1.96) | -17.34 (-28.05, -5.04) |
| Xiyang | Shanxi | -10.70 (-12.77, -8.59) | -9.14 (-14.58, -3.36) | -20.63 (-29.68, -10.41) | -18.44 (-31.97, -2.21) |
| Yangcheng | Shanxi | -5.20 (-7.54, -2.80) | -8.05 (-24.47, 11.94) | -6.06 (-17.09, 6.43) | -18.29 (-30.11, -4.47) |
| Yanggao | Shanxi | -5.71 (-8.64, -2.68) | 11.07 (-8.69, 35.11) | -15.06 (-19.30, -10.60) | -1.20 (-11.21, 9.95) |
| Yangqu | Shanxi | -6.17 (-8.65, -3.62) | 4.76 (-9.18, 20.85) | -14.43 (-24.37, -3.19) | -16.24 (-20.27, -12.00) |
| Yicheng | Shanxi | -11.08 (-13.28, -8.83) | -14.63 (-24.89, -2.98) | -21.70 (-38.18, -0.84) | -2.79 (-9.17, 4.04) |
| Ying | Shanxi | -3.27 (-6.79, 0.37) | 2.33 (-38.91, 71.40) | -4.36 (-16.91, 10.08) | -2.36 (-13.80, 10.60) |
| Yonghe | Shanxi | -5.66 (-7.87, -3.41) | 1.86 (-13.99, 20.64) | -3.71 (-13.42, 7.10) | -2.73 (-25.14, 26.39) |
| Yongji | Shanxi | -3.13 (-7.21, 1.14) | 15.18 (-19.15, 64.09) | -11.45 (-17.03, -5.49) | -12.04 (-24.85, 2.96) |
| Youyu | Shanxi | -6.21 (-8.72, -3.63) | -11.40 (-32.57, 16.42) | -0.98 (-14.88, 15.18) | -16.21 (-28.82, -1.37) |
| Yu | Shanxi | -6.21 (-8.55, -3.81) | -2.45 (-4.75, -0.09) | -16.89 (-22.99, -10.30) | 1.57 (-13.63, 19.44) |
| Yuanping | Shanxi | -7.66 (-9.70, -5.57) | -9.45 (-20.20, 2.74) | 2.18 (-11.13, 17.48) | -9.35 (-11.80, -6.83) |
| Yuanqu | Shanxi | -4.82 (-6.76, -2.84) | -2.92 (-20.81, 19.02) | -9.32 (-23.32, 7.23) | -10.91 (-20.67, 0.05) |
| Yunzhou | Shanxi | -6.47 (-8.71, -4.18) | -18.50 (-26.33, -9.83) | -8.75 (-20.41, 4.61) | -0.84 (-16.75, 18.10) |
| Yushe | Shanxi | -10.46 (-13.06, -7.78) | -5.20 (-11.95, 2.07) | -7.76 (-18.89, 4.90) | -13.47 (-40.82, 26.52) |
| Zezhou | Shanxi | -5.75 (-7.79, -3.66) | -7.56 (-22.47, 10.23) | -2.05 (-7.45, 3.66) | -16.41 (-30.91, 1.14) |
| Zhangzi | Shanxi | -5.68 (-7.98, -3.32) | 4.77 (-17.84, 33.62) | -8.51 (-14.16, -2.49) | 0.70 (-13.14, 16.74) |
| Zhongyang | Shanxi | -3.74 (-6.25, -1.15) | 8.17 (-10.11, 30.17) | -5.25 (-17.40, 8.68) | -14.35 (-19.02, -9.41) |
| Zuoquan | Shanxi | -7.35 (-10.27, -4.33) | -2.79 (-14.21, 10.15) | -5.96 (-12.24, 0.78) | -6.37 (-17.26, 5.95) |
| Zuoyun | Shanxi | -4.81 (-7.46, -2.09) | -13.99 (-40.26, 23.84) | -9.91 (-18.64, -0.24) | -1.74 (-12.88, 10.82) |
| Aba | Sichuan | -5.16 (-8.61, -1.57) | 6.17 (-16.05, 34.27) | -7.54 (-43.12, 50.31) | -7.30 (-15.98, 2.28) |
| Anyue | Sichuan | -2.16 (-5.98, 1.83) | 7.53 (-5.59, 22.48) | 0.45 (-1.06, 1.99) | -19.68 (-29.90, -7.97) |
| Anzhou | Sichuan | -10.91 (-18.02, -3.18) | 5.86 (-9.35, 23.62) | -0.64 (-7.36, 6.57) | -5.98 (-67.48, 171.8) |
| Baiyu | Sichuan | 7.98 (2.24, 14.06) | 0.25 (-45.50, 84.42) | 5.45 (-17.29, 34.43) | -11.45 (-42.60, 36.61) |
| Baoxing | Sichuan | -5.85 (-8.98, -2.61) | 11.37 (0.14, 23.86) | -10.02 (-36.33, 27.17) | -0.45 (-20.56, 24.74) |
| Barkam | Sichuan | -10.22 (-13.77, -6.52) | 7.37 (-8.41, 25.86) | -17.32 (-35.72, 6.36) | -11.05 (-41.23, 34.64) |
| Batang | Sichuan | 4.41 (1.26, 7.66) | -4.04 (-23.89, 20.98) | 13.10 (-2.28, 30.90) | 3.63 (-14.78, 26.03) |
| Beichuan | Sichuan | -4.36 (-7.10, -1.54) | -8.85 (-25.33, 11.26) | -0.35 (-12.7, 13.74) | -20.39 (-29.83, -9.69) |
| Butuo | Sichuan | 4.74 (1.06, 8.55) | 6.96 (-7.76, 24.04) | -2.65 (-22.50, 22.28) | 20.78 (-4.43, 52.64) |
| Cangxi | Sichuan | -6.50 (-8.44, -4.52) | -18.07 (-23.47, -12.28) | 0.15 (-15.43, 18.6) | -4.85 (-10.5, 1.16) |
| Changning | Sichuan | -0.8 (-2.20, 0.61) | -5.26 (-15.44, 6.15) | 3.36 (-6.07, 13.73) | 0.55 (-11.79, 14.62) |
| Chongzhou | Sichuan | -7.72 (-10.27, -5.11) | -13.45 (-20.17, -6.17) | -5.30 (-21.63, 14.44) | 4.19 (-6.78, 16.45) |
| Dachuan | Sichuan | -10.3 (-12.72, -7.82) | -1.58 (-30.19, 38.74) | -9.83 (-19.45, 0.95) | -10.69 (-24.29, 5.34) |
| Danleng | Sichuan | -4.29 (-6.53, -1.99) | -9.79 (-29.21, 14.95) | 2.36 (-6.67, 12.25) | 5.70 (-7.65, 20.99) |
| Danba | Sichuan | -3.30 (-5.32, -1.23) | -10.29 (-18.27, -1.53) | -2.51 (-8.45, 3.81) | -8.33 (-27.46, 15.86) |
| Daocheng | Sichuan | 5.12 (1.69, 8.67) | 1.60 (-19.2, 27.75) | 21.49 (9.49, 34.80) | 4.14 (-25.54, 45.66) |
| Daofu | Sichuan | 6.31 (1.71, 11.13) | 13.29 (-6.46, 37.22) | 29.88 (-2.27, 72.61) | 12.02 (-22.25, 61.39) |
| Dayi | Sichuan | -7.26 (-9.06, -5.43) | -10.88 (-20.81, 0.31) | -4.09 (-14.55, 7.67) | -4.26 (-12.74, 5.04) |
| Daying | Sichuan | -7.26 (-9.25, -5.23) | -15.91 (-28.00, -1.79) | -0.06 (-5.35, 5.52) | -14.78 (-25.41, -2.64) |
| Dazhu | Sichuan | -8.36 (-8.99, -7.73) | -7.90 (-15.07, -0.12) | -9.06 (-15.33, -2.34) | -7.63 (-9.35, -5.89) |
| Dechang | Sichuan | -5.57 (-9.66, -1.28) | -13.56 (-20.69, -5.79) | -18.08 (-33.24, 0.53) | -5.57 (-14.69, 4.53) |
| Dege | Sichuan | 1.05 (-2.85, 5.11) | -2.51 (-11.19, 7.03) | -9.57 (-30.60, 17.84) | -11.22 (-41.26, 34.18) |
| Derong | Sichuan | 2.00 (-1.81, 5.96) | -2.04 (-26.63, 30.78) | 24.58 (-0.01, 55.21) | -10.15 (-24.25, 6.56) |
| Dujiangyan | Sichuan | -7.07 (-9.52, -4.55) | -15.76 (-34.44, 8.25) | -1.89 (-13.80, 11.66) | -2.62 (-4.22, -0.99) |
| Ebian | Sichuan | -4.76 (-8.36, -1.02) | -22.41 (-31.70, -11.86) | -7.44 (-22.78, 10.93) | 9.83 (-0.16, 20.81) |
| Emeishan | Sichuan | -9.79 (-12.16, -7.36) | -12.82 (-30.99, 10.13) | -10.99 (-22.42, 2.11) | -1.07 (-15.99, 16.49) |
| Fushun | Sichuan | -0.33 (-1.94, 1.32) | 3.77 (-8.72, 17.97) | 1.51 (-12.27, 17.45) | -6.79 (-15.90, 3.31) |
| Ganluo | Sichuan | -3.23 (-6.38, 0.02) | 7.31 (-9.52, 27.28) | -22.00 (-38.37, -1.27) | -4.17 (-10.63, 2.76) |
| Ganzi | Sichuan | 6.44 (3.13, 9.85) | 13.57 (2.15, 26.27) | 11.29 (-9.44, 36.75) | -4.14 (-19.97, 14.82) |
| Gao | Sichuan | -1.13 (-2.09, -0.16) | 0.47 (-5.76, 7.11) | -2.00 (-8.42, 4.87) | -3.80 (-10.14, 3.00) |
| Gong | Sichuan | -2.34 (-3.84, -0.81) | -0.30 (-10.82, 11.46) | -4.13 (-14.83, 7.91) | -8.05 (-15.96, 0.60) |
| Guanghan | Sichuan | -5.32 (-8.06, -2.51) | -0.43 (-5.12, 4.50) | 0.39 (-7.94, 9.47) | -19.92 (-39.05, 5.21) |
| Gulin | Sichuan | -5.54 (-6.59, -4.48) | -2.26 (-10.21, 6.38) | -4.81 (-9.16, -0.25) | -4.73 (-9.95, 0.79) |
| Hanyuan | Sichuan | -1.47 (-3.76, 0.87) | -9.09 (-22.30, 6.36) | -0.17 (-9.36, 9.96) | 3.27 (-19.40, 32.31) |
| Heishui | Sichuan | -9.09 (-12.98, -5.02) | 1.08 (-15.21, 20.49) | -7.79 (-22.42, 9.59) | 1.94 (-30.71, 49.99) |
| Hejiang | Sichuan | -5.02 (-6.81, -3.20) | -11.08 (-17.83, -3.77) | -5.07 (-12.13, 2.56) | -4.64 (-9.95, 0.99) |
| Hongya | Sichuan | -6.52 (-8.41, -4.59) | -0.69 (-17.14, 19.02) | -7.16 (-16.68, 3.46) | -10.46 (-27.15, 10.06) |
| Hongyuan | Sichuan | -2.94 (-10.17, 4.87) | 30.91 (-21.70, 118.87) | -16.99 (-36.69, 8.84) | 43.72 (23.48, 67.27) |
| Huaying | Sichuan | -5.02 (-7.58, -2.40) | -7.05 (-22.31, 11.20) | 0.20 (-10.35, 11.99) | -14.31 (-32.05, 8.08) |
| Huidong | Sichuan | -0.31 (-1.75, 1.15) | -5.89 (-13.51, 2.40) | 3.84 (-8.51, 17.86) | -1.39 (-11.89, 10.36) |
| Huili | Sichuan | -6.40 (-11.72, -0.76) | 23.31 (-29.32, 115.13) | 2.73 (-2.03, 7.71) | -3.80 (-19.84, 15.46) |
| Jiajiang | Sichuan | -8.97 (-10.75, -7.15) | -5.64 (-19.05, 9.99) | -7.66 (-21.38, 8.46) | -2.56 (-18.16, 16.00) |
| Jiangan | Sichuan | -1.62 (-3.47, 0.26) | 2.73 (-5.40, 11.56) | -0.04 (-18.39, 22.43) | -9.30 (-17.36, -0.45) |
| Jiange | Sichuan | -4.89 (-7.34, -2.37) | -16.11 (-19.56, -12.52) | 13.09 (1.52, 25.98) | -3.10 (-10.33, 4.71) |
| Jiangyou | Sichuan | -3.48 (-4.55, -2.40) | -1.84 (-11.75, 9.18) | -5.37 (-9.50, -1.05) | -7.26 (-13.87, -0.15) |
| Jianyang | Sichuan | -4.84 (-8.82, -0.68) | -5.76 (-13.38, 2.53) | 5.70 (-14.46, 30.62) | -7.39 (-17.53, 4.01) |
| Jinchuan | Sichuan | -2.20 (-5.39, 1.10) | -5.62 (-20.25, 11.71) | -8.46 (-29.95, 19.61) | -1.37 (-11.55, 9.98) |
| Jingyan | Sichuan | -4.37 (-6.05, -2.65) | -10.47 (-12.76, -8.12) | -3.79 (-23.01, 20.23) | -4.86 (-13.05, 4.10) |
| Jintang | Sichuan | -1.75 (-3.66, 0.21) | -10.50 (-15.00, -5.76) | 2.30 (-12.95, 20.22) | -3.46 (-14.71, 9.27) |
| Jinyang | Sichuan | 2.74 (0.44, 5.09) | 8.85 (-2.37, 21.35) | 0.26 (-17.68, 22.13) | 5.24 (-9.00, 21.71) |
| Jiulong | Sichuan | 1.73 (-1.18, 4.72) | 5.22 (-28.29, 54.37) | 4.83 (-14.03, 27.84) | 4.79 (0.63, 9.12) |
| Jiuzhaigou | Sichuan | -2.60 (-5.69, 0.59) | 16.07 (-1.93, 37.38) | -6.18 (-25.77, 18.58) | -1.34 (-15.89, 15.73) |
| Junlian | Sichuan | -2.01 (-4.27, 0.30) | 0.36 (-24.81, 33.96) | 0.10 (-9.80, 11.08) | -5.85 (-15.75, 5.21) |
| Kaijiang | Sichuan | -8.52 (-11.50, -5.45) | -26.89 (-32.04, -21.34) | 0.30 (-9.74, 11.47) | -6.01 (-17.46, 7.03) |
| Kangding | Sichuan | -6.79 (-11.25, -2.11) | -3.60 (-20.22, 16.47) | 10.20 (-4.01, 26.51) | -4.55 (-9.62, 0.82) |
| Langzhong | Sichuan | -5.82 (-8.02, -3.57) | -14.64 (-25.73, -1.89) | -1.26 (-8.11, 6.10) | 6.51 (2.71, 10.45) |
| Leibo | Sichuan | 7.43 (5.24, 9.66) | 16.55 (12.45, 20.80) | 13.77 (3.87, 24.61) | 2.59 (-17.53, 27.62) |
| Lezhi | Sichuan | -3.78 (-8.38, 1.05) | 2.10 (-17.78, 26.79) | 7.95 (0.73, 15.69) | -19.28 (-28.00, -9.51) |
| Li | Sichuan | -5.39 (-7.92, -2.79) | -1.06 (-22.99, 27.10) | 2.48 (-15.34, 24.04) | 1.65 (-11.02, 16.13) |
| Linshui | Sichuan | -3.06 (-4.93, -1.16) | 4.41 (-7.78, 18.21) | -9.41 (-14.01, -4.56) | 7.07 (1.30, 13.17) |
| Litang | Sichuan | 4.79 (-1.03, 10.95) | 15.5 (-10.34, 48.79) | -11.88 (-36.36, 22.01) | 38.25 (0.61, 89.96) |
| Longchang | Sichuan | -5.86 (-7.32, -4.36) | -3.35 (-10.69, 4.59) | -5.01 (-15.75, 7.09) | -4.91 (-16.52, 8.30) |
| Lu | Sichuan | -8.64 (-10.16, -7.10) | -9.31 (-18.36, 0.75) | -13.09 (-22.96, -1.94) | 0.38 (-7.11, 8.48) |
| Luding | Sichuan | -1.41 (-5.66, 3.02) | 1.08 (-39.11, 67.81) | -2.90 (-16.31, 12.66) | -19.73 (-27.51, -11.11) |
| Luhuo | Sichuan | -0.78 (-6.01, 4.75) | 6.42 (1.23, 11.89) | -18.38 (-40.95, 12.81) | 35.15 (-8.01, 98.56) |
| Luojiang | Sichuan | -5.82 (-9.79, -1.68) | -11.40 (-22.22, 0.92) | 3.46 (-8.26, 16.67) | -25.25 (-48.12, 7.70) |
| Lushan | Sichuan | -6.44 (-9.67, -3.09) | 9.60 (3.42, 16.16) | -14.79 (-30.51, 4.47) | 5.89 (-11.04, 26.03) |
| Mabian | Sichuan | 2.99 (0.22, 5.84) | -8.22 (-16.39, 0.74) | 10.61 (-3.99, 27.44) | -10.68 (-24.20, 5.25) |
| Mao | Sichuan | -6.10 (-10.41, -1.58) | -26.90 (-39.15, -12.18) | 2.39 (-15.84, 24.56) | 15.86 (0.15, 34.04) |
| Meigu | Sichuan | 10.13 (5.45, 15.03) | 8.93 (-44.25, 112.83) | 0.58 (-7.90, 9.84) | 12.13 (1.22, 24.21) |
| Mianning | Sichuan | -0.78 (-3.69, 2.23) | 14.20 (-8.57, 42.64) | -11.97 (-21.96, -0.71) | 12.15 (9.02, 15.37) |
| Mianzhu | Sichuan | -4.84 (-7.06, -2.56) | -2.45 (-19.09, 17.61) | 0.06 (-5.17, 5.58) | -16.87 (-24.65, -8.28) |
| Mingshan | Sichuan | -6.31 (-11.51, -0.81) | 8.92 (-3.09, 22.41) | -25.31 (-33.05, -16.67) | 37.48 (6.57, 77.36) |
| Miyi | Sichuan | -2.93 (-6.40, 0.66) | -5.38 (-21.45, 13.98) | -11.53 (-24.09, 3.11) | 6.77 (-17.76, 38.61) |
| Muchuan | Sichuan | 0.83 (-1.28, 2.98) | -0.45 (-18.16, 21.10) | 8.95 (-0.44, 19.22) | -1.03 (-20.08, 22.56) |
| Muli | Sichuan | 5.94 (3.58, 8.35) | 11.12 (-16.24, 47.41) | -3.03 (-11.39, 6.12) | 2.99 (-5.36, 12.09) |
| Nanbu | Sichuan | -0.76 (-2.73, 1.24) | -4.32 (-9.20, 0.82) | 10.68 (-3.95, 27.55) | -9.03 (-17.72, 0.57) |
| Nanjiang | Sichuan | -2.07 (-3.64, -0.48) | -4.00 (-6.38, -1.57) | 5.84 (1.57, 10.30) | -3.84 (-14.19, 7.75) |
| Nanxi | Sichuan | 0.62 (-1.65, 2.93) | 6.77 (-11.08, 28.19) | -5.23 (-21.08, 13.80) | -6.07 (-10.23, -1.72) |
| Ningnan | Sichuan | -0.10 (-2.65, 2.52) | 9.22 (-5.43, 26.14) | -5.34 (-11.42, 1.15) | 10.48 (2.72, 18.82) |
| Pengan | Sichuan | -5.94 (-7.19, -4.67) | -10.16 (-15.62, -4.34) | 0.84 (-8.30, 10.90) | -7.63 (-16.06, 1.63) |
| Pengxi | Sichuan | -4.67 (-6.07, -3.25) | -13.32 (-22.49, -3.07) | -4.47 (-10.05, 1.45) | -7.80 (-13.31, -1.95) |
| Pengzhou | Sichuan | -5.29 (-7.44, -3.09) | -15.22 (-30.85, 3.94) | -2.58 (-15.53, 12.35) | -6.48 (-10.27, -2.53) |
| Pengshan | Sichuan | -3.39 (-5.25, -1.50) | -7.95 (-20.64, 6.77) | -3.32 (-11.19, 5.25) | -3.40 (-17.93, 13.70) |
| Pidou | Sichuan | -7.31 (-9.31, -5.26) | -7.95 (-24.49, 12.20) | -15.26 (-25.74, -3.30) | 1.52 (-5.48, 9.04) |
| Pingchang | Sichuan | -9.47 (-11.03, -7.89) | -9.00 (-18.54, 1.65) | -6.61 (-12.44, -0.39) | -0.50 (-10.53, 10.65) |
| Pingshan | Sichuan | -2.84 (-4.04, -1.63) | -8.92 (-17.49, 0.53) | -2.71 (-6.38, 1.11) | 2.80 (-3.31, 9.30) |
| Pingwu | Sichuan | -0.21 (-3.60, 3.30) | 0.61 (-16.1, 20.65) | 8.19 (-9.08, 28.74) | -13.10 (-40.98, 27.94) |
| Puge | Sichuan | 3.46 (-1.24, 8.38) | -15.36 (-30.26, 2.72) | -2.50 (-15.82, 12.92) | 13.22 (1.32, 26.51) |
| Pujiang | Sichuan | -5.17 (-7.23, -3.07) | 1.22 (-7.12, 10.30) | -0.53 (-13.21, 13.99) | -0.98 (-10.73, 9.83) |
| Qianwei | Sichuan | -4.77 (-7.54, -1.91) | -10.73 (-15.85, -5.30) | -0.81 (-10.87, 10.38) | 17.14 (7.23, 27.97) |
| Qingchuan | Sichuan | -0.95 (-2.71, 0.85) | -6.26 (-15.10, 3.50) | 0.02 (-8.48, 9.32) | -1.43 (-19.96, 21.40) |
| Qingshen | Sichuan | -6.45 (-8.22, -4.64) | -4.73 (-8.63, -0.67) | -4.86 (-15.35, 6.94) | -7.98 (-13.55, -2.04) |
| Qionglai | Sichuan | -5.35 (-7.59, -3.06) | -10.24 (-19.98, 0.69) | -9.05 (-14.97, -2.72) | 0.76 (-6.93, 9.08) |
| Qu | Sichuan | -3.94 (-5.55, -2.31) | 5.08 (-5.33, 16.62) | -5.27 (-13.07, 3.23) | -7.37 (-20.70, 8.19) |
| Rangtang | Sichuan | -5.65 (-12.58, 1.81) | 31.23 (5.10, 63.86) | -36.85 (-50.12, -20.05) | 34.25 (16.58, 54.60) |
| Renshou | Sichuan | -7.69 (-9.88, -5.45) | 1.70 (-6.14, 10.19) | -19.03 (-32.03, -3.55) | 0 (-5.32, 5.61) |
| Rong | Sichuan | 0.16 (-1.95, 2.31) | -13.65 (-16.55, -10.65) | 7.95 (-5.93, 23.87) | 0.79 (-4.97, 6.89) |
| Ruoergai | Sichuan | -0.40 (-3.31, 2.59) | 2.21 (-14.38, 22.01) | 5.54 (-26.15, 50.82) | 5.65 (-10.37, 24.53) |
| Santai | Sichuan | -1.77 (-4.67, 1.23) | -0.96 (-14.14, 14.24) | 4.35 (-4.29, 13.78) | -18.03 (-28.47, -6.06) |
| Seda | Sichuan | 4.67 (-2.97, 12.90) | 49.19 (30.37, 70.72) | -8.54 (-43.08, 46.94) | 41.80 (-1.75, 104.65) |
| Shehong | Sichuan | -5.11 (-6.11, -4.10) | -6.03 (-10.83, -0.98) | -1.42 (-8.30, 5.97) | -2.31 (-8.82, 4.67) |
| Shenfang | Sichuan | -5.29 (-8.92, -1.50) | -10.27 (-30.34, 15.58) | -0.12 (-4.29, 4.24) | -23.87 (-33.67, -12.62) |
| Shimian | Sichuan | -3.17 (-5.80, -0.47) | -13.18 (-22.29, -3.01) | -6.07 (-15.36, 4.23) | 14.75 (-2.92, 35.63) |
| Shiqu | Sichuan | 13.98 (11.39, 16.63) | 10.33 (-13.66, 40.99) | 19.63 (7.42, 33.23) | 2.68 (-9.30, 16.25) |
| Shuangliu | Sichuan | -4.60 (-5.75, -3.43) | -5.31 (-16.36, 7.20) | -6.01 (-10.05, -1.79) | -0.07 (-6.09, 6.35) |
| Songpan | Sichuan | -0.53 (-4.18, 3.26) | 4.87 (-8.43, 20.10) | -7.26 (-27.43, 18.51) | 4.99 (-1.28, 11.65) |
| Tianquan | Sichuan | -1.81 (-5.14, 1.62) | 9.11 (-2.74, 22.41) | -10.87 (-30.81, 14.82) | 19.02 (14.46, 23.76) |
| Tongjiang | Sichuan | -8.02 (-10.58, -5.38) | -12.73 (-19.41, -5.49) | -11.4 (-27.23, 7.88) | 9.28 (-0.82, 20.41) |
| Wangcang | Sichuan | -3.75 (-5.77, -1.69) | -16.60 (-24.18, -8.27) | -1.15 (-8.24, 6.49) | -3.20 (-12.14, 6.65) |
| Wanyuan | Sichuan | -2.83 (-4.50, -1.12) | 0.65 (-12.71, 16.06) | 1.39 (-9.27, 13.29) | -1.96 (-17.22, 16.10) |
| Weiyuan | Sichuan | -5.19 (-6.32, -4.05) | -3.03 (-11.17, 5.85) | -2.77 (-9.65, 4.63) | -4.52 (-17.08, 9.93) |
| Wenchuan | Sichuan | -9.36 (-12.35, -6.26) | -19.87 (-34.71, -1.66) | -2.84 (-20.57, 18.85) | 7.36 (-12.65, 31.95) |
| Wusheng | Sichuan | -1.03 (-2.32, 0.28) | 0.60 (-1.77, 3.03) | 0.66 (-14.07, 17.92) | -3.17 (-5.43, -0.86) |
| Xiangcheng | Sichuan | 5.83 (3.32, 8.41) | -2.78 (-15.73, 12.17) | 3.00 (-6.89, 13.94) | -3.36 (-24.36, 23.47) |
| Xiaojin | Sichuan | -4.27 (-8.57, 0.24) | -11.86 (-27.21, 6.74) | 3.63 (-32.67, 59.50) | 8.56 (-19.13, 45.72) |
| Xichang | Sichuan | -3.20 (-5.53, -0.81) | 1.64 (-10.36, 15.24) | -10.85 (-22.75, 2.89) | 0.73 (-19.32, 25.77) |
| Xichong | Sichuan | -3.55 (-5.60, -1.45) | -8.35 (-24.19, 10.79) | -4.05 (-14.88, 8.16) | 5.27 (-9.56, 22.54) |
| Xide | Sichuan | 5.22 (0.44, 10.23) | 6.28 (-14.20, 31.65) | -10.60 (-11.94, -9.23) | 33.60 (-8.96, 96.06) |
| Xingjing | Sichuan | -4.81 (-10.44, 1.17) | -10.07 (-45.56, 48.53) | -13.91 (-29.45, 5.05) | 1.10 (-13.94, 18.75) |
| Xingwen | Sichuan | 0.44 (-1.20, 2.10) | 0.25 (-13.26, 15.86) | 4.09 (-5.31, 14.42) | -0.76 (-10.2, 9.67) |
| Xinjin | Sichuan | -4.84 (-6.76, -2.87) | -9.03 (-20.28, 3.81) | -1.25 (-6.45, 4.24) | 0.22 (-5.12, 5.86) |
| Xinlong | Sichuan | -7.60 (-14.06, -0.66) | 4.44 (-11.95, 23.88) | 20.05 (-3.88, 49.93) | -17.65 (-46.04, 25.67) |
| Xuanhan | Sichuan | -8.72 (-10.39, -7.01) | -16.23 (-29.72, -0.15) | -1.80 (-10.87, 8.20) | -7.16 (-13.91, 0.13) |
| Xuyong | Sichuan | -2.57 (-4.24, -0.86) | -3.70 (-19.70, 15.47) | 2.80 (-7.52, 14.27) | -5.80 (-15.14, 4.58) |
| Xuzhou | Sichuan | -0.59 (-3.02, 1.89) | -0.93 (-22.81, 27.16) | 1.61 (-7.12, 11.16) | -9.67 (-15.39, -3.56) |
| Yajiang | Sichuan | 4.29 (-0.03, 8.79) | -0.96 (-8.44, 7.13) | 28.29 (1.94, 61.45) | -6.78 (-41.35, 48.16) |
| Yanbian | Sichuan | -3.26 (-4.91, -1.58) | -3.75 (-17.86, 12.79) | -1.31 (-3.64, 1.08) | 3.59 (-8.22, 16.92) |
| Yanting | Sichuan | -10.26 (-12.74, -7.70) | -11.44 (-23.40, 2.39) | 3.90 (-7.52, 16.74) | -22.14 (-34.32, -7.71) |
| Yanyuan | Sichuan | -0.46 (-5.39, 4.73) | 0.17 (-20.36, 25.99) | -19.94 (-37.44, 2.44) | 13.87 (-12.67, 48.48) |
| Yilong | Sichuan | -2.64 (-4.06, -1.20) | -1.96 (-9.31, 5.99) | 1.95 (-12.87, 19.30) | -2.11 (-9.17, 5.50) |
| Yingshan | Sichuan | -1.82 (-4.20, 0.62) | 6.83 (-4.77, 19.85) | 0.40 (-4.67, 5.73) | -15.48 (-27.42, -1.58) |
| Yuechi | Sichuan | -0.52 (-1.70, 0.67) | -6.30 (-12.84, 0.73) | 0.83 (-2.20, 3.95) | 4.79 (-1.02, 10.95) |
| Yuexi | Sichuan | 3.59 (-0.48, 7.83) | 7.87 (-19.76, 45.03) | -4.23 (-18.35, 12.34) | 3.17 (-14.06, 23.87) |
| Zhaojiao | Sichuan | 2.11 (-1.46, 5.8) | -0.02 (-25.21, 33.64) | 5.46 (-0.46, 11.73) | 26.96 (7.87, 49.42) |
| Zhongjiang | Sichuan | -1.95 (-4.11, 0.26) | -6.93 (-28.88, 21.81) | -0.43 (-4.61, 3.95) | -6.01 (-15.30, 4.29) |
| Zitong | Sichuan | -2.81 (-5.58, 0.05) | -2.21 (-13.89, 11.06) | 6.12 (-15.23, 32.84) | -17.50 (-26.34, -7.60) |
| Zizhong | Sichuan | -4.32 (-6.37, -2.22) | -1.54 (-13.22, 11.72) | -0.92 (-7.47, 6.08) | -11.57 (-20.5, -1.64) |
| Jinghai | Tianjin | -1.64 (-5.95, 2.86) | -18.47 (-34.75, 1.87) | 7.29 (-13.56, 33.18) | 12.69 (-1.23, 28.57) |
| Jizhou | Tianjin | -4.71 (-6.46, -2.92) | -8.32 (-19.91, 4.96) | -7.11 (-20.71, 8.81) | 4.36 (-2.84, 12.10) |
| Ninghe | Tianjin | -1.09 (-2.93, 0.79) | -4.41 (-16.89, 9.95) | 4.07 (-6.98, 16.43) | 9.70 (-0.60, 21.06) |
| Aheqi | Xinjiang | -3.50 (-6.13, -0.79) | 3.21 (-25.33, 42.67) | -10.37 (-26.36, 9.10) | 4.30 (-1.15, 10.06) |
| Akesu | Xinjiang | 2.09 (-5.77, 10.60) | -25.07 (-51.08, 14.76) | 30.94 (16.38, 47.31) | 24.09 (-2.00, 57.14) |
| Aketao | Xinjiang | -1.87 (-5.43, 1.83) | 5.18 (-7.79, 19.96) | -10.09 (-20.52, 1.70) | -1.02 (-40.42, 64.45) |
| Alear | Xinjiang | -5.17 (-10.85, 0.86) | 0 (0, 0) | #NUM! | -6.52 (-30.61, 25.92) |
| Aletai | Xinjiang | -0.43 (-2.02, 1.17) | -2.48 (-11.93, 7.98) | 5.76 (0.50, 11.30) | 2.22 (-12.36, 19.24) |
| Atushi | Xinjiang | -5.12 (-7.39, -2.79) | 1.50 (-14.98, 21.17) | -15.52 (-27.66, -1.34) | -8.87 (-20.40, 4.33) |
| Avati | Xinjiang | 3.73 (-4.06, 12.16) | -29.29 (-54.02, 8.76) | 35.93 (2.45, 80.35) | 4.17 (-39.20, 78.48) |
| Bachu | Xinjiang | 5.25 (1.24, 9.43) | -6.85 (-29.20, 22.56) | 11.45 (-15.02, 46.17) | -2.68 (-33.80, 43.06) |
| Baicheng | Xinjiang | 3.48 (-3.22, 10.65) | -22.99 (-31.18, -13.82) | 33.66 (-0.36, 79.30) | 23.12 (-17.89, 84.62) |
| Barkol | Xinjiang | -4.61 (-8.44, -0.61) | -12.36 (-26.96, 5.16) | 6.88 (-7.47, 23.45) | -26.25 (-41.08, -7.67) |
| Bohu | Xinjiang | -3.50 (-5.43, -1.52) | -7.45 (-20.78, 8.13) | 5.08 (-11.11, 24.22) | -5.03 (-14.41, 5.38) |
| Bole | Xinjiang | -2.46 (-4.62, -0.25) | -0.95 (-8.52, 7.25) | -1.56 (-8.93, 6.41) | -7.03 (-29.73, 23.00) |
| Buerjin | Xinjiang | -4.69 (-6.01, -3.35) | -3.17 (-16.33, 12.06) | -3.54 (-16.48, 11.41) | -5.30 (-12.84, 2.89) |
| Cele | Xinjiang | -4.56 (-9.51, 0.66) | 0.98 (-6.03, 8.51) | -8.06 (-29.04, 19.13) | 14.23 (-45.35, 138.79) |
| Changji | Xinjiang | -7.50 (-10.08, -4.84) | -7.31 (-19.10, 6.20) | -7.52 (-23.78, 12.21) | -5.94 (-21.23, 12.32) |
| Emin | Xinjiang | 3.62 (1.14, 6.17) | -5.35 (-17.47, 8.56) | 15.08 (0.06, 32.35) | 2.91 (-17.53, 28.42) |
| Fuhai | Xinjiang | -1.60 (-4.19, 1.07) | 3.67 (-8.73, 17.76) | 6.18 (-8.11, 22.70) | -12.44 (-28.77, 7.64) |
| Fukang | Xinjiang | -5.91 (-9.35, -2.34) | -16.24 (-33.22, 5.05) | 6.52 (-0.51, 14.04) | 1.41 (-15.81, 22.15) |
| Fuyun | Xinjiang | -0.53 (-3.62, 2.66) | -10.09 (-24.89, 7.63) | 14.45 (-8.40, 43.01) | -10.27 (-20.91, 1.82) |
| Gaochang | Xinjiang | -7.70 (-9.78, -5.57) | -7.89 (-23.97, 11.61) | 0.58 (-7.94, 9.90) | -8.11 (-22.20, 8.54) |
| Gashi | Xinjiang | 0.86 (-1.20, 2.97) | 13.47 (4.24, 23.53) | -9.39 (-15.24, -3.13) | 0.11 (-11.27, 12.95) |
| Gongliu | Xinjiang | -1.63 (-4.00, 0.79) | -11.33 (-22.18, 1.03) | 1.13 (-15.15, 20.54) | -0.51 (-23.29, 29.02) |
| Habahe | Xinjiang | -3.00 (-5.14, -0.80) | 6.93 (-3.53, 18.52) | 0.38 (-13.03, 15.85) | -5.88 (-18.97, 9.33) |
| Hejing | Xinjiang | -1.11 (-5.32, 3.28) | -7.72 (-44.76, 54.13) | 10.25 (-8.65, 33.06) | -8.51 (-35.53, 29.83) |
| Heshuo | Xinjiang | 0.62 (-2.38, 3.70) | -11.05 (-14.82, -7.12) | 19.38 (8.62, 31.21) | -5.26 (-25.95, 21.22) |
| Hetian | Xinjiang | -5.38 (-9.87, -0.67) | 3.60 (-25.70, 44.46) | -9.08 (-23.64, 8.25) | 23.76 (1.74, 50.55) |
| Hetian | Xinjiang | 7.54 (2.77, 12.54) | -0.68 (-41.76, 69.37) | 12.95 (-7.17, 37.45) | 4.38 (-32.14, 60.54) |
| Hoboksar | Xinjiang | -4.09 (-5.99, -2.15) | 9.04 (3.13, 15.29) | -5.60 (-14.36, 4.05) | 3.52 (-5.59, 13.52) |
| Huocheng | Xinjiang | -1.20 (-3.24, 0.88) | -7.75 (-29.56, 20.81) | 0.21 (-5.98, 6.81) | -3.12 (-13.87, 8.98) |
| Hutubi | Xinjiang | -6.01 (-10.32, -1.50) | -30.47 (-44.75, -12.49) | 14.12 (5.58, 23.35) | -13.77 (-17.06, -10.34) |
| Jimsar | Xinjiang | 0.30 (-3.01, 3.73) | -10.45 (-35.55, 24.44) | 19.65 (10.46, 29.60) | -6.08 (-14.94, 3.71) |
| Jimunai | Xinjiang | -4.22 (-7.32, -1.01) | 7.81 (-9.80, 28.86) | -5.47 (-21.97, 14.52) | -9.09 (-23.21, 7.62) |
| Jinghe | Xinjiang | -3.36 (-6.26, -0.36) | -2.64 (-27.29, 30.38) | 12.07 (-7.75, 36.15) | -10.64 (-16.39, -4.50) |
| Kashi | Xinjiang | -2.13 (-5.82, 1.70) | 8.09 (-17.80, 42.15) | -11.28 (-23.59, 3.03) | 6.75 (-30.87, 64.82) |
| Keping | Xinjiang | 0.44 (-5.28, 6.50) | -29.16 (-43.70, -10.87) | 27.71 (5.25, 54.96) | -4.74 (-20.96, 14.80) |
| Kuche | Xinjiang | 3.53 (-1.29, 8.60) | -14.97 (-34.39, 10.19) | 21.42 (-12.18, 67.88) | 8.23 (-9.17, 28.98) |
| Kuerle | Xinjiang | -6.30 (-10.3, -2.13) | -7.38 (-31.19, 24.66) | 5.53 (-10.68, 24.69) | -0.48 (-21.28, 25.80) |
| Kuitun | Xinjiang | 6.15 (3.59, 8.77) | 4.41 (-17.67, 32.43) | 19.76 (1.89, 40.75) | 1.56 (-8.45, 12.66) |
| Luntai | Xinjiang | -2.52 (-8.62, 3.99) | -15.15 (-35.76, 12.08) | -12.31 (-26.82, 5.08) | 13.06 (-51.64, 164.3) |
| Luopu | Xinjiang | 4.35 (0, 8.89) | -11.55 (-21.11, -0.83) | 30.50 (6.71, 59.6) | 2.59 (-24.20, 38.84) |
| Maigaiti | Xinjiang | 5.91 (3.06, 8.84) | 8.52 (-10.91, 32.19) | -3.14 (-17.20, 13.32) | 5.66 (-19.08, 37.96) |
| Manas | Xinjiang | -4.66 (-6.59, -2.69) | -0.34 (-14.83, 16.61) | 4.65 (-11.70, 24.02) | -5.52 (-15.05, 5.09) |
| Minfeng | Xinjiang | -1.06 (-6.69, 4.91) | -5.53 (-29.82, 27.16) | 22.93 (-5.73, 60.31) | -1.21 (-32.49, 44.55) |
| Mori | Xinjiang | -3.06 (-7.82, 1.94) | -21.17 (-39.96, 3.51) | 31.07 (14.61, 49.90) | -9.30 (-21.40, 4.66) |
| Moyu | Xinjiang | 1.94 (-0.88, 4.84) | 0.18 (-15.57, 18.88) | 5.44 (-7.56, 20.27) | 4.23 (-29.48, 54.06) |
| Nileke | Xinjiang | -3.16 (-6.49, 0.30) | -13.48 (-31.34, 9.04) | 3.83 (-6.01, 14.69) | -7.74 (-40.49, 43.05) |
| Pishan | Xinjiang | 1.07 (-1.93, 4.16) | -6.11 (-20.97, 11.53) | 11.01 (-20.93, 55.84) | 6.85 (1.11, 12.92) |
| Qapqal Xibe | Xinjiang | -4.94 (-7.22, -2.60) | -12.49 (-16.22, -8.60) | 6.60 (-4.34, 18.80) | -5.79 (-27.55, 22.52) |
| Qiemo | Xinjiang | -5.95 (-11.57, 0.03) | -0.79 (-36.41, 54.77) | -3.90 (-26.43, 25.53) | 3.93 (-43.21, 90.20) |
| Qinghe | Xinjiang | -4.45 (-6.72, -2.14) | -8.69 (-19.37, 3.40) | -2.47 (-26.81, 29.98) | -1.11 (-14.80, 14.79) |
| Qitai | Xinjiang | 0.72 (-2.67, 4.22) | -15.08 (-31.77, 5.69) | 16.88 (5.04, 30.05) | -9.97 (-24.50, 7.36) |
| Ruoqiang | Xinjiang | -8.96 (-14.53, -3.03) | -12.45 (-35.00, 17.92) | 17.98 (-25.37, 86.51) | -23.29 (-57.26, 37.65) |
| Shache | Xinjiang | 4.95 (1.30, 8.72) | 15.36 (9.10, 21.97) | -0.15 (-20.10, 24.77) | 15.63 (-24.66, 77.48) |
| Shanshan | Xinjiang | -7.81 (-12.03, -3.39) | -9.35 (-40.66, 38.49) | 5.65 (-8.35, 21.79) | -9.97 (-46.51, 51.51) |
| Shawan | Xinjiang | -6.44 (-10.04, -2.69) | 19.83 (-11.18, 61.66) | -16.97 (-25.36, -7.64) | 2.32 (-4.23, 9.32) |
| Shaya | Xinjiang | -0.80 (-4.21, 2.73) | -7.28 (-21.75, 9.86) | 15.09 (-6.27, 41.31) | 9.36 (-8.93, 31.31) |
| Shihezi | Xinjiang | -1.42 (-4.33, 1.58) | -7.10 (-28.6, 20.88) | 13.07 (-12.25, 45.70) | 4.28 (-1.06, 9.90) |
| Shufu | Xinjiang | 3.00 (-0.11, 6.21) | 10.90 (-1.97, 25.46) | -1.65 (-13.45, 11.75) | 0.65 (-34.57, 54.83) |
| Shule | Xinjiang | 9.06 (5.27, 12.99) | 29.52 (1.01, 66.08) | 12.13 (-1.76, 27.98) | 2.13 (-28.68, 46.26) |
| Tacheng | Xinjiang | -1.66 (-3.24, -0.05) | 0.48 (-6.19, 7.61) | 6.19 (-3.04, 16.30) | 3.04 (-10.84, 19.07) |
| Tashkurgan Tajik | Xinjiang | 0.21 (-2.59, 3.09) | 4.27 (-16.57, 30.32) | -4.79 (-16.78, 8.92) | -4.10 (-32.36, 35.95) |
| Tekes | Xinjiang | -1.93 (-4.47, 0.68) | -0.59 (-12.01, 12.32) | 8.85 (-13.94, 37.67) | -4.50 (-21.94, 16.83) |
| Toksun | Xinjiang | -9.49 (-12.99, -5.84) | -7.03 (-35.92, 34.88) | -4.79 (-23.81, 18.97) | -3.61 (-29.67, 32.12) |
| Tumxuk | Xinjiang | -0.09 (-6.07, 6.28) | 0 (0, 0) | #NUM! | -4.06 (-21.42, 17.15) |
| Tuoli | Xinjiang | -0.05 (-1.85, 1.78) | 3.02 (-6.52, 13.54) | -5.95 (-16.56, 6.02) | 0.58 (-10.96, 13.62) |
| Urumchi | Xinjiang | 1.93 (-2.48, 6.53) | -5.01 (-14.11, 5.06) | 20.35 (-19.37, 79.64) | -14.88 (-38.54, 17.89) |
| Weili | Xinjiang | -4.00 (-8.77, 1.01) | -26.45 (-42.85, -5.34) | 5.49 (-28.83, 56.37) | -11.91 (-39.02, 27.26) |
| Wenquan | Xinjiang | -1.17 (-3.51, 1.22) | -7.14 (-22.61, 11.41) | -0.34 (-12.10, 12.99) | -1.67 (-26.86, 32.19) |
| Wenxiu | Xinjiang | -0.93 (-5.01, 3.33) | -16.23 (-34.17, 6.59) | 6.43 (-16.86, 36.25) | 18.12 (-9.55, 54.26) |
| Wujiaqu | Xinjiang | -0.48 (-3.64, 2.79) | 0 (0, 0) | #NUM! | -1.49 (-17.07, 17.02) |
| Wuqia | Xinjiang | -1.67 (-6.00, 2.85) | 18.55 (6.87, 31.51) | -9.88 (-26.35, 10.26) | 8.48 (-37.20, 87.39) |
| Wushi | Xinjiang | 2.19 (-5.43, 10.42) | -21.45 (-48.22, 19.17) | 23.19 (-8.63, 66.08) | 24.86 (-1.63, 58.48) |
| Wusu | Xinjiang | -8.25 (-9.82, -6.65) | -4.88 (-10.14, 0.68) | -9.01 (-23.34, 7.99) | -4.29 (-7.92, -0.51) |
| Xinhe | Xinjiang | -1.65 (-7.39, 4.45) | -9.94 (-26.98, 11.08) | -19.88 (-47.22, 21.63) | 16.56 (-33.00, 102.79) |
| Xinyuan | Xinjiang | -1.61 (-3.58, 0.39) | -6.62 (-13.50, 0.80) | 2.18 (-5.20, 10.13) | 1.50 (-22.96, 33.72) |
| Yanqihuizu | Xinjiang | 3.08 (-2.05, 8.47) | -13.23 (-25.42, 0.95) | 19.25 (-2.25, 45.48) | -3.60 (-48.86, 81.70) |
| Yecheng | Xinjiang | 9.14 (4.97, 13.47) | 25.34 (20.55, 30.32) | 11.16 (-2.09, 26.19) | -5.09 (-42.42, 56.44) |
| Yingjisha | Xinjiang | 7.87 (4.39, 11.46) | 23.88 (8.51, 41.43) | 1.03 (-14.25, 19.05) | 2.01 (-29.29, 47.18) |
| Yining | Xinjiang | -2.87 (-4.87, -0.84) | -3.74 (-5.82, -1.60) | -5.64 (-16.63, 6.79) | -7.04 (-30.25, 23.90) |
| Yining | Xinjiang | -1.01 (-3.96, 2.03) | -3.44 (-9.92, 3.51) | 11.74 (0.52, 24.21) | -0.04 (-31.79, 46.48) |
| Yiwu | Xinjiang | -3.15 (-6.71, 0.54) | -8.69 (-22.81, 8.01) | -8.83 (-31.46, 21.29) | -12.67 (-43.49, 34.96) |
| Yizhou | Xinjiang | -2.43 (-4.37, -0.45) | -10.25 (-29.69, 14.56) | -3.13 (-12.94, 7.78) | -4.15 (-12.42, 4.89) |
| Yuepuhu | Xinjiang | 5.48 (-1.10, 12.50) | -13.98 (-35.96, 15.54) | 20.39 (-10.03, 61.10) | -20.03 (-54.52, 40.60) |
| Yumin | Xinjiang | -1.12 (-2.99, 0.79) | 6.42 (1.72, 11.33) | -3.10 (-15.82, 11.54) | -4.19 (-21.97, 17.64) |
| Yutian | Xinjiang | 1.60 (-1.90, 5.23) | -0.46 (-32.60, 47.01) | 1.05 (-17.99, 24.51) | 16.20 (-9.65, 49.44) |
| Zepu | Xinjiang | 7.33 (4.23, 10.52) | 8.81 (-5.65, 25.49) | 3.97 (-11.36, 21.95) | -1.87 (-32.87, 43.45) |
| Zhaosu | Xinjiang | 1.74 (-4.48, 8.37) | -26.72 (-44.54, -3.18) | 37.77 (-8.54, 107.52) | -10.70 (-29.99, 13.91) |
| Anduo | Xizang | 11.10 (2.21, 20.76) | -30.64 (-70.95, 65.61) | 24.99 (-19.99, 95.26) | 20.94 (5.32, 38.87) |
| Angren | Xizang | 18.12 (9.88, 26.98) | 81.80 (-5.59, 250.07) | 37.88 (11.79, 70.06) | -6.12 (-32.72, 30.99) |
| Bange | Xizang | 13.88 (3.96, 24.74) | 24.25 (-15.37, 82.42) | 30.27 (-11.09, 90.88) | 20.95 (-22.18, 87.97) |
| Baqing | Xizang | 28.64 (16.58, 41.95) | 98.45 (-71.87, 1300.01) | 15.24 (-9.46, 46.68) | 25.78 (-16.05, 88.45) |
| Baxiu | Xizang | 6.01 (1.22, 11.04) | -3.27 (-47.56, 78.42) | 3.20 (-13.55, 23.19) | 14.10 (-11.35, 46.85) |
| Bayi | Xizang | -0.25 (-2.17, 1.72) | -6.41 (-22.53, 13.08) | -1.06 (-15.08, 15.28) | -2.28 (-15.47, 12.97) |
| Bianba | Xizang | 6.10 (2.12, 10.23) | -2.68 (-14.05, 10.20) | 3.10 (-35.11, 63.81) | 3.20 (-22.51, 37.45) |
| Biru | Xizang | 27.08 (17.30, 37.69) | -1.34 (-99.81, 51407.37) | 36.95 (-7.86, 103.55) | 19.04 (8.67, 30.41) |
| Bomi | Xizang | -1.13 (-5.79, 3.76) | -19.56 (-41.48, 10.56) | -1.63 (-30.86, 39.95) | 17.39 (-10.69, 54.31) |
| Caiya | Xizang | 6.43 (2.08, 10.96) | 3.45 (-25.17, 43.02) | 1.49 (-13.84, 19.54) | 10.96 (-17.48, 49.19) |
| Chayu | Xizang | 7.45 (3.07, 12.01) | 7.97 (-22.52, 50.47) | 42.52 (31.10, 54.95) | 0.24 (-5.23, 6.02) |
| Cuomei | Xizang | 0.15 (-4.35, 4.86) | -14.59 (-44.24, 30.82) | 1.65 (-28.35, 44.22) | 1.30 (-32.24, 51.46) |
| Cuona | Xizang | 5.19 (-0.27, 10.94) | 7.21 (-25.44, 54.17) | -17.35 (-44.19, 22.40) | 1.64 (-30.39, 48.40) |
| Cuoqin | Xizang | 0.40 (-8.77, 10.48) | -28.84 (-65.90, 48.47) | 7.10 (-36.05, 79.38) | 30.16 (-35.2, 161.44) |
| Dangxiong | Xizang | 2.50 (-1.26, 6.41) | 25.23 (-16.59, 88.01) | 0.94 (-16.61, 22.19) | -5.88 (-11.69, 0.32) |
| Dazi | Xizang | 6.04 (-0.98, 13.56) | 43.72 (-33.69, 211.50) | 7.91 (-18.00, 42.01) | 7.25 (-25.33, 54.04) |
| Dingjie | Xizang | 5.54 (1.80, 9.43) | 13.11 (-15.93, 52.18) | 5.66 (-22.71, 44.43) | 3.34 (-24.42, 41.29) |
| Dingqing | Xizang | 3.29 (-0.07, 6.75) | -9.78 (-35.97, 27.11) | -4.33 (-15.74, 8.62) | 4.90 (0.20, 9.83) |
| Dingri | Xizang | 4.30 (-0.34, 9.15) | -2.63 (-31.95, 39.32) | -1.18 (-38.31, 58.29) | 9.54 (-25.20, 60.42) |
| Duilongdeqing | Xizang | 5.43 (1.47, 9.54) | 5.04 (0.42, 9.88) | 33.54 (-6.87, 91.50) | 1.74 (-17.63, 25.67) |
| Gaer | Xizang | 16.85 (5.99, 28.82) | 73.18 (-69.40, 880.17) | -7.85 (-18.32, 3.98) | 21.47 (-1.94, 50.47) |
| Gaize | Xizang | 17.87 (8.91, 27.56) | 25.51 (-57.36, 269.39) | 7.38 (-47.16, 118.23) | 40.56 (-15.43, 133.64) |
| Gangba | Xizang | 5.30 (-5.38, 17.19) | 25.34 (-99.86, 109563.130) | 5.94 (-26.42, 52.54) | -7.33 (-57.50, 102.05) |
| Geji | Xizang | 16.41 (9.18, 24.13) | 28.27 (-80.38, 738.43) | 5.34 (-29.85, 58.21) | 28.44 (0.67, 63.89) |
| Gongbujiangda | Xizang | 2.02 (-0.30, 4.40) | 6.25 (-13.82, 31.00) | 10.17 (-5.75, 28.78) | -0.26 (-15.04, 17.10) |
| Gongga | Xizang | 2.11 (-0.09, 4.37) | -1.47 (-17.74, 18.03) | 6.99 (-11.07, 28.72) | 8.12 (-10.38, 30.45) |
| Gongjiao | Xizang | 9.25 (4.80, 13.88) | -12.25 (-39.46, 27.21) | 7.50 (-6.93, 24.17) | 12.48 (-7.46, 36.70) |
| Gyaca | Xizang | -0.20 (-5.40, 5.29) | 11.26 (-14.85, 45.38) | -25.57 (-45.68, 2.00) | 5.89 (-30.02, 60.23) |
| Jiali | Xizang | 33.81 (20.39, 48.72) | 66.42 (-96.35, 7478.11) | 136.79 (75.15, 220.12) | 4.61 (-15.64, 29.73) |
| Jiangda | Xizang | 9.97 (6.27, 13.79) | 6.99 (-10.95, 28.53) | -3.61 (-17.59, 12.75) | 8.19 (-18.15, 43.02) |
| Jiangzi | Xizang | 0.99 (-4.01, 6.26) | -15.58 (-49.64, 41.51) | 9.53 (-16.44, 43.58) | -3.29 (-37.99, 50.83) |
| Jilong | Xizang | 7.97 (-0.93, 17.68) | 79.73 (-35.76, 402.85) | 37.60 (1.11, 87.26) | -4.30 (-29.64, 30.18) |
| Kangma | Xizang | 3.99 (-1.11, 9.36) | 33.20 (-33.26, 165.83) | -2.80 (-26.35, 28.30) | -6.28 (-26.46, 19.43) |
| Karuo | Xizang | -0.48 (-3.85, 3.01) | 5.97 (-14.50, 31.34) | -11.49 (-23.91, 2.97) | 15.22 (-17.29, 60.49) |
| Lang | Xizang | 7.81 (0.62, 15.52) | -14.88 (-56.39, 66.12) | 48.52 (32.14, 66.94) | 6.41 (-24.20, 49.39) |
| Langqiazi | Xizang | 3.25 (0.31, 6.29) | -5.27 (-21.38, 14.14) | 10.35 (-0.93, 22.93) | -6.67 (-19.77, 8.57) |
| Lazi | Xizang | 5.16 (-5.78, 17.36) | -34.27 (-84.03, 170.44) | 8.42 (-1.39, 19.20) | -10.06 (-17.99, -1.36) |
| Leiwuqi | Xizang | 2.24 (-1.37, 5.98) | -11.77 (-38.72, 27.04) | -5.02 (-26.94, 23.47) | 7.14 (3.00, 11.45) |
| Lhozhag | Xizang | -4.34 (-10.34, 2.05) | 24.65 (-7.62, 68.20) | -1.02 (-22.88, 27.04) | -20.62 (-52.59, 32.89) |
| Linzhou | Xizang | 6.98 (1.41, 12.86) | 25.13 (-33.28, 134.69) | 24.10 (1.70, 51.43) | -9.05 (-16.40, -1.05) |
| Longzi | Xizang | 3.88 (1.38, 6.45) | -5.41 (-26.66, 22.00) | 7.32 (-0.43, 15.68) | 2.22 (-14.96, 22.86) |
| Luolong | Xizang | 8.63 (5.90, 11.44) | -5.65 (-17.06, 7.32) | 15.39 (-2.84, 37.03) | 11.30 (-8.84, 35.89) |
| Mangkang | Xizang | 3.88 (-0.67, 8.64) | -22.67 (-43.88, 6.56) | 20.26 (6.78, 35.43) | 11.84 (-4.59, 31.12) |
| Milin | Xizang | 0.61 (-2.76, 4.09) | -10.76 (-32.79, 18.49) | -4.38 (-29.78, 30.20) | 13.21 (1.90, 25.76) |
| Motuo | Xizang | 1.67 (-1.49, 4.92) | 3.83 (-29.60, 53.12) | 0.63 (-13.39, 16.92) | 0.03 (-22.08, 28.41) |
| Mozhugongka | Xizang | 2.61 (-2.24, 7.69) | 0.70 (-29.69, 44.21) | 19.93 (-23.15, 87.17) | 2.50 (-6.76, 12.67) |
| Naidong | Xizang | 4.26 (0.27, 8.42) | -2.12 (-45.71, 76.49) | 2.99 (-3.67, 10.12) | 8.66 (-5.95, 25.53) |
| Nanmulin | Xizang | 8.66 (3.17, 14.44) | 6.15 (-48.50, 118.79) | 4.60 (-26.70, 49.26) | -2.17 (-11.86, 8.59) |
| Nielamu | Xizang | 15.56 (5.71, 26.33) | 21.42 (-99.83, 87253.53) | 11.04 (-17.27, 49.03) | 13.63 (2.37, 26.14) |
| Nierong | Xizang | 24.68 (10.54, 40.64) | 28.79 (-100.00, 4247844.35) | 40.82 (-8.87, 117.61) | 17.22 (-11.19, 54.72) |
| Nima | Xizang | 26.06 (17.37, 35.39) | 0 (0, 0) | -4.50 (-57.33, 113.71) | 16.73 (2.86, 32.47) |
| Nimu | Xizang | -0.95 (-4.82, 3.07) | -17.05 (-24.85, -8.43) | 6.31 (-33.15, 69.05) | -11.19 (-15.56, -6.58) |
| Pulan | Xizang | 18.40 (11.44, 25.80) | 59.51 (-8.46, 177.96) | 6.72 (-24.81, 51.46) | 18.39 (-7.31, 51.22) |
| Qiongjie | Xizang | 0.03 (-3.85, 4.06) | 1.46 (-26.54, 40.14) | 17.69 (10.68, 25.13) | 19.26 (1.69, 39.86) |
| Qushui | Xizang | -1.06 (-6.82, 5.05) | -12.65 (-33.93, 15.47) | 43.58 (4.97, 96.40) | -9.15 (-42.62, 43.85) |
| Qusong | Xizang | 4.43 (-0.16, 9.22) | 11.43 (-4.25, 29.67) | 17.94 (-25.69, 87.19) | 15.43 (0.81, 32.17) |
| Renbu | Xizang | 5.43 (-6.40, 18.75) | 135.63 (-17.01, 569.03) | -5.84 (-21.96, 13.62) | -4.87 (-27.41, 24.66) |
| Ritu | Xizang | 12.69 (4.23, 21.85) | 2.37 (-44.54, 88.96) | -1.94 (-55.89, 117.95) | 11.74 (-44.73, 125.89) |
| Saga | Xizang | 7.48 (-1.93, 17.78) | 9.05 (-78.65, 456.97) | 2.82 (-55.13, 135.63) | 11.20 (-22.82, 60.21) |
| Sajia | Xizang | 13.17 (5.87, 20.98) | 36.23 (-15.41, 119.4) | -9.99 (-21.60, 3.35) | 2.55 (-24.53, 39.34) |
| Sanee | Xizang | 32.16 (12.34, 55.47) | 12.72 (-89.06, 1061.00) | 8.12 (-31.83, 71.47) | 9.87 (-11.19, 35.93) |
| Sangri | Xizang | -0.07 (-5.92, 6.15) | -4.29 (-32.84, 36.38) | -5.84 (-42.53, 54.25) | 0.72 (-17.55, 23.04) |
| Sangzhuzi | Xizang | 7.03 (-0.03, 14.59) | -3.32 (-63.27, 154.46) | 7.44 (-11.9, 31.03) | -7.49 (-19.68, 6.56) |
| Shenza | Xizang | 25.24 (15.34, 35.99) | 0 (0, 0) | 49.09 (14.53, 94.09) | 9.17 (-22.51, 53.81) |
| Suo | Xizang | 42.03 (29.55, 55.71) | 92.38 (-71.14, 1182.28) | 29.66 (2.44, 64.12) | 31.99 (3.69, 68.00) |
| Xietongmen | Xizang | 8.87 (-1.41, 20.23) | 97.63 (-49.01, 666.04) | -10.05 (-23.37, 5.58) | 2.97 (-10.90, 19.01) |
| Yadong | Xizang | -1.35 (-6.78, 4.39) | -1.34 (-48.71, 89.80) | -3.98 (-27.57, 27.29) | 9.85 (-35.60, 87.38) |
| Zhada | Xizang | 16.14 (7.70, 25.25) | 12.29 (-98.97, 12139.15) | 13.01 (-25.52, 71.47) | 54.57 (43.33, 66.70) |
| Zhanang | Xizang | 2.93 (0.24, 5.70) | 1.58 (-16.23, 23.18) | 7.07 (-15.09, 35.02) | -4.02 (-23.46, 20.34) |
| Zhongba | Xizang | 14.77 (5.29, 25.11) | 10.95 (-63.92, 241.24) | 8.92 (-46.46, 121.59) | 1.60 (-11.29, 16.37) |
| Zuogong | Xizang | 11.39 (5.85, 17.22) | 9.24 (-45.52, 119.06) | 9.52 (-22.51, 54.80) | 10.84 (-15.8, 45.91) |
| Anning | Yunnan | -3.80 (-6.10, -1.45) | -7.95 (-20.25, 6.24) | -9.07 (-11.20, -6.89) | 12.05 (-4.34, 31.23) |
| Binchuan | Yunnan | -4.90 (-7.52, -2.21) | -18.55 (-21.09, -15.92) | -3.05 (-20.55, 18.31) | 3.32 (-7.39, 15.27) |
| Cangyuan | Yunnan | -1.04 (-3.76, 1.74) | -1.57 (-19.74, 20.70) | -3.66 (-13.85, 7.74) | -4.76 (-29.20, 28.11) |
| Changning | Yunnan | -8.05 (-11.15, -4.85) | -17.49 (-35.14, 4.95) | -0.04 (-9.61, 10.55) | 0.76 (-6.64, 8.75) |
| Chenggong | Yunnan | -2.91 (-6.45, 0.76) | -11.17 (-26.06, 6.73) | -6.87 (-26.20, 17.53) | 14.28 (7.70, 21.27) |
| Chengjiang | Yunnan | -4.91 (-7.59, -2.14) | -16.58 (-35.93, 8.61) | -6.46 (-20.81, 10.5) | -4.94 (-20.58, 13.78) |
| Chuxiong | Yunnan | -6.91 (-8.53, -5.26) | -0.87 (-15.28, 15.99) | -7.10 (-8.97, -5.20) | -11.95 (-25.17, 3.61) |
| Daguan | Yunnan | -3.57 (-6.71, -0.33) | 20.24 (5.04, 37.63) | -9.53 (-16.82, -1.59) | 0.90 (-21.97, 30.47) |
| Dali | Yunnan | -7.09 (-10.42, -3.64) | -18.98 (-33.16, -1.80) | -3.04 (-13.72, 8.96) | 6.78 (-11.84, 29.32) |
| Dayao | Yunnan | -3.15 (-4.65, -1.62) | -9.16 (-20.54, 3.85) | 2.07 (-2.99, 7.39) | -7.89 (-13.31, -2.14) |
| Deqin | Yunnan | 0.86 (-1.67, 3.45) | -11.85 (-29.04, 9.48) | 3.36 (-10.27, 19.07) | 7.58 (-7.63, 25.31) |
| Eryuan | Yunnan | -1.64 (-3.30, 0.06) | -10.99 (-16.80, -4.77) | 3.17 (-6.55, 13.9) | 0.97 (-10.77, 14.26) |
| Eshan | Yunnan | -3.84 (-7.05, -0.51) | -19.45 (-43.96, 15.80) | -6.69 (-21.73, 11.24) | -7.61 (-19.25, 5.71) |
| Fengqing | Yunnan | -1.23 (-4.16, 1.79) | -4.50 (-19.89, 13.85) | -1.00 (-11.71, 11.00) | -14.20 (-35.35, 13.88) |
| Fugong | Yunnan | 1.36 (-0.67, 3.43) | 13.66 (1.26, 27.57) | 0.88 (-5.49, 7.69) | 5.16 (-9.39, 22.05) |
| Fumin | Yunnan | 1.28 (-1.45, 4.09) | 7.93 (-7.67, 26.17) | -5.90 (-19.55, 10.05) | 5.16 (-4.64, 15.96) |
| Funing | Yunnan | -5.41 (-9.20, -1.47) | -7.23 (-20.39, 8.10) | 4.77 (-33.46, 64.97) | -1.82 (-24.78, 28.15) |
| Fuyuan | Yunnan | 0.28 (-1.87, 2.49) | -6.12 (-11.72, -0.17) | 4.36 (-19.01, 34.47) | -1.55 (-15.90, 15.25) |
| Gejiu | Yunnan | -8.75 (-11.98, -5.41) | -22.33 (-34.87, -7.38) | -8.59 (-15.08, -1.60) | 8.40 (-3.40, 21.64) |
| Gengma | Yunnan | 0.88 (-2.19, 4.04) | -1.42 (-12.33, 10.86) | 6.93 (-8.70, 25.24) | -10.09 (-34.38, 23.17) |
| Gongshan | Yunnan | 4.99 (2.80, 7.23) | 2.31 (-20.16, 31.10) | 6.72 (-7.20, 22.73) | 14.86 (7.16, 23.12) |
| Guangnan | Yunnan | 1.07 (-0.65, 2.82) | 8.69 (-6.48, 26.33) | -1.42 (-10.95, 9.13) | 6.43 (-4.21, 18.24) |
| Hekou | Yunnan | -4.54 (-8.12, -0.83) | -10.90 (-20.10, -0.64) | 2.97 (-9.63, 17.32) | -9.04 (-36.36, 30.01) |
| Heqing | Yunnan | -1.66 (-4.07, 0.80) | 0.47 (-17.25, 21.99) | 2.01 (-6.29, 11.05) | -12.90 (-20.42, -4.68) |
| Honghe | Yunnan | 3.60 (0.23, 7.10) | -1.34 (-26.02, 31.57) | -0.91 (-17.16, 18.54) | 23.22 (-1.45, 54.07) |
| Huaning | Yunnan | -7.31 (-9.99, -4.56) | 3.27 (-18.22, 30.40) | -5.25 (-9.48, -0.82) | -18.52 (-31.08, -3.67) |
| Huaping | Yunnan | -3.70 (-6.62, -0.68) | 6.41 (-18.28, 38.56) | -5.06 (-22.37, 16.11) | -4.08 (-10.37, 2.64) |
| Huize | Yunnan | 0.41 (-1.51, 2.36) | -5.16 (-15.03, 5.85) | 2.69 (-15.28, 24.47) | -2.64 (-15.99, 12.85) |
| Jianchuan | Yunnan | 2.02 (0.37, 3.70) | 5.77 (-9.74, 23.93) | -1.37 (-5.69, 3.16) | -0.71 (-16.68, 18.33) |
| Jiangcheng | Yunnan | 2.69 (0.91, 4.50) | -2.82 (-14.4, 10.32) | 4.91 (-5.42, 16.37) | 0.88 (-8.83, 11.62) |
| Jiangchuan | Yunnan | -2.03 (-4.01, -0.02) | -12.75 (-22.48, -1.78) | -0.73 (-15.87, 17.12) | 0.88 (-9.58, 12.55) |
| Jianshui | Yunnan | -4.45 (-7.82, -0.95) | -22.83 (-44.35, 7.01) | -1.44 (-3.50, 0.67) | -2.11 (-17.70, 16.44) |
| Jingdong | Yunnan | 5.41 (2.80, 8.09) | 7.59 (-5.36, 22.32) | 10.9 (-3.99, 28.09) | 25.55 (16.86, 34.89) |
| Jinggu | Yunnan | 4.68 (2.00, 7.44) | -8.07 (-24.43, 11.83) | 5.02 (-6.60, 18.08) | 20.10 (12.02, 28.77) |
| Jinghong | Yunnan | 0.29 (-1.94, 2.57) | -5.72 (-11.31, 0.22) | 0.15 (-2.31, 2.67) | 5.80 (-2.27, 14.54) |
| Jinning | Yunnan | -3.13 (-5.21, -0.99) | -10.69 (-22.19, 2.49) | 4.07 (-7.18, 16.70) | -6.78 (-25.05, 15.94) |
| Jinping | Yunnan | -0.11 (-2.50, 2.34) | -0.98 (-15.06, 15.43) | 2.65 (-9.71, 16.70) | 10.10 (-15.23, 43.01) |
| Kaiyuan | Yunnan | -1.85 (-4.98, 1.38) | -8.27 (-20.85, 6.31) | -6.72 (-25.06, 16.11) | 15.35 (-12.98, 52.89) |
| Lancang | Yunnan | 4.68 (2.94, 6.45) | 4.59 (-1.37, 10.90) | 5.34 (-10.55, 24.06) | 15.86 (8.71, 23.49) |
| Lanping | Yunnan | 6.38 (3.75, 9.08) | -1.23 (-20.59, 22.86) | 10.5 (-2.57, 25.33) | 14.39 (-10.42, 46.06) |
| Lianghe | Yunnan | 1.84 (-1.20, 4.98) | 3.96 (-21.08, 36.94) | -7.49 (-10.86, -4.00) | 21.53 (3.43, 42.80) |
| Longchuan | Yunnan | -1.06 (-3.85, 1.81) | -9.63 (-16.96, -1.66) | 0.13 (-9.31, 10.56) | 12.14 (-1.68, 27.91) |
| Longling | Yunnan | -3.49 (-5.40, -1.55) | -3.02 (-25.65, 26.48) | -4.74 (-13.03, 4.34) | 2.67 (-7.19, 13.57) |
| Ludian | Yunnan | 3.38 (0.71, 6.13) | 1.92 (-13.65, 20.30) | 6.61 (0.89, 12.66) | -8.87 (-31.77, 21.71) |
| Lufeng | Yunnan | -1.15 (-2.62, 0.34) | -2.26 (-13.98, 11.06) | -0.89 (-11.24, 10.67) | -4.79 (-18.37, 11.06) |
| Luliang | Yunnan | -1.08 (-4.24, 2.18) | 1.61 (-31.63, 51.02) | 14.07 (5.11, 23.81) | -4.57 (-12.68, 4.28) |
| Luoping | Yunnan | -3.36 (-5.21, -1.47) | -5.90 (-15.04, 4.22) | 0.61 (-15.22, 19.40) | -0.98 (-10.68, 9.78) |
| Luquan | Yunnan | 2.59 (-1.22, 6.55) | 0.27 (-14.67, 17.83) | -4.41 (-31.46, 33.32) | 3.05 (-9.63, 17.51) |
| Lushui | Yunnan | 2.12 (-2.02, 6.43) | 12.24 (-14.37, 47.12) | 12.65 (-8.03, 37.99) | 21.38 (6.07, 38.89) |
| Luxi | Yunnan | -0.98 (-3.09, 1.18) | -6.50 (-25.3, 17.04) | -7.36 (-19.59, 6.74) | -5.28 (-12.91, 3.01) |
| Lvchun | Yunnan | 1.51 (-0.96, 4.04) | 14.47 (9.26, 19.93) | 0.91 (-15.81, 20.95) | 9.49 (0.03, 19.83) |
| Maguan | Yunnan | -2.38 (-5.10, 0.42) | -3.60 (-27.85, 28.80) | 6.49 (-2.22, 15.98) | 2.56 (-15.20, 24.05) |
| Malipo | Yunnan | 0.77 (-1.76, 3.37) | 10.73 (-16.62, 47.05) | -2.79 (-11.43, 6.69) | 9.36 (-4.69, 25.49) |
| Malong | Yunnan | -2.86 (-6.09, 0.48) | 11.94 (-15.19, 47.76) | -7.08 (-26.81, 17.97) | 6.22 (-15.93, 34.21) |
| Mang | Yunnan | 0.13 (-2.60, 2.93) | -9.33 (-21.72, 5.03) | 2.77 (-6.25, 12.66) | 10.65 (6.40, 15.08) |
| Menghai | Yunnan | 7.41 (2.93, 12.08) | -3.78 (-14.16, 7.86) | -2.45 (-9.41, 5.06) | 9.49 (-26.28, 62.62) |
| Mengla | Yunnan | 1.37 (-2.61, 5.52) | -12.42 (-30.25, 9.96) | -7.21 (-19.47, 6.91) | 13.10 (-5.59, 35.49) |
| Menglian | Yunnan | 8.60 (6.00, 11.26) | 0.85 (-13.94, 18.18) | 12.02 (-15.90, 49.23) | 3.88 (-1.25, 9.27) |
| Mengzi | Yunnan | -1.96 (-5.02, 1.19) | -14.09 (-37.63, 18.34) | 2.20 (-19.21, 29.29) | 3.05 (-9.84, 17.77) |
| Midu | Yunnan | -4.06 (-7.13, -0.88) | -18.76 (-24.97, -12.04) | 1.78 (-6.89, 11.26) | 6.35 (-2.17, 15.60) |
| Mile | Yunnan | 0.07 (-3.19, 3.44) | -17.36 (-29.11, -3.65) | 2.47 (-0.13, 5.13) | 11.77 (5.86, 18.01) |
| Mojiang | Yunnan | 4.48 (1.20, 7.86) | -3.05 (-20.01, 17.51) | 6.55 (2.11, 11.18) | 28.81 (1.54, 63.39) |
| Mouding | Yunnan | -3.14 (-5.03, -1.21) | 2.38 (-3.42, 8.53) | -0.80 (-5.10, 3.69) | -12.37 (-24.62, 1.87) |
| Nanhua | Yunnan | 0.37 (-1.10, 1.86) | 5.98 (-6.16, 19.69) | -4.28 (-8.36, -0.03) | -3.80 (-13.76, 7.31) |
| Nanjian | Yunnan | -0.23 (-2.36, 1.95) | -2.16 (-11.94, 8.71) | 2.83 (-16.61, 26.79) | 11.45 (1.31, 22.60) |
| Ninger | Yunnan | 1.07 (-2.23, 4.47) | -12.71 (-25.89, 2.81) | -0.21 (-13.01, 14.47) | 16.80 (3.68, 31.59) |
| Ninglang | Yunnan | 1.78 (-2.23, 5.96) | 22.33 (-6.82, 60.59) | -9.08 (-23.89, 8.62) | 4.83 (-14.45, 28.46) |
| Pingbian | Yunnan | 0.36 (-2.46, 3.27) | -6.83 (-21.18, 10.14) | 7.11 (-13.24, 32.23) | 15.40 (-5.75, 41.29) |
| Qiaojia | Yunnan | 1.56 (-1.53, 4.76) | 8.92 (-27.59, 63.86) | 2.44 (-3.70, 8.99) | -0.96 (-14.45, 14.67) |
| Qiubei | Yunnan | 1.17 (-0.45, 2.82) | -0.81 (-19.65, 22.45) | -0.08 (-6.28, 6.53) | -0.01 (-12.53, 14.29) |
| Ruili | Yunnan | 0.97 (-1.40, 3.40) | -8.58 (-20.16, 4.69) | 8.52 (1.67, 15.84) | -4.41 (-23.86, 20.00) |
| Shidian | Yunnan | 1.99 (-0.23, 4.25) | -11.69 (-23.23, 1.59) | 2.64 (-9.02, 15.79) | 9.18 (2.26, 16.57) |
| Shilin | Yunnan | -4.91 (-8.86, -0.78) | -19.37 (-28.29, -9.33) | -10.56 (-19.74, -0.33) | 20.17 (6.32, 35.82) |
| Shiping | Yunnan | -2.37 (-4.14, -0.57) | -11.96 (-15.54, -8.23) | -0.50 (-8.94, 8.74) | 6.33 (1.06, 11.88) |
| Shizong | Yunnan | 4.66 (2.43, 6.94) | 9.03 (-17.65, 44.37) | 9.99 (-3.91, 25.90) | 0.88 (-6.82, 9.22) |
| Shuangbai | Yunnan | -8.27 (-11.85, -4.55) | -25.69 (-45.82, 1.94) | -0.05 (-7.46, 7.95) | -3.84 (-9.28, 1.93) |
| Shuangjiang | Yunnan | -1.23 (-3.18, 0.77) | 3.60 (-10.51, 19.93) | -0.97 (-20.45, 23.27) | -7.23 (-14.65, 0.84) |
| Shuifu | Yunnan | 0.47 (-3.67, 4.80) | 18.39 (-22.12, 79.99) | -1.87 (-16.17, 14.86) | 7.71 (-25.79, 56.33) |
| Simao | Yunnan | 6.05 (3.76, 8.40) | -3.59 (-18.37, 13.87) | 6.20 (-9.55, 24.68) | 4.79 (-12.50, 25.50) |
| Songming | Yunnan | 2.52 (-0.23, 5.36) | -5.69 (-8.97, -2.29) | 10.58 (-16.49, 46.42) | -2.31 (-9.60, 5.57) |
| Suijiang | Yunnan | -1.57 (-3.93, 0.83) | -4.30 (-18.16, 11.89) | 6.84 (-14.37, 33.29) | -0.97 (-7.86, 6.44) |
| Tengchong | Yunnan | 1.89 (-0.13, 3.95) | -2.87 (-17.85, 14.85) | -0.31 (-8.13, 8.18) | 3.33 (-15.51, 26.37) |
| Tonghai | Yunnan | -5.56 (-8.31, -2.73) | -16.38 (-34.12, 6.15) | 2.58 (-3.65, 9.21) | -16.14 (-31.43, 2.56) |
| Weishan | Yunnan | -2.14 (-7.58, 3.61) | -21.21 (-40.55, 4.43) | 1.22 (-11.69, 16.01) | 20.10 (1.94, 41.49) |
| Weixi | Yunnan | 1.74 (-2.62, 6.29) | 11.59 (3.72, 20.05) | -4.97 (-13.53, 4.44) | 39.92 (17.03, 67.29) |
| Weixin | Yunnan | 4.62 (1.77, 7.55) | 17.79 (-9.67, 53.61) | 2.01 (-8.46, 13.68) | 5.92 (-8.29, 22.32) |
| Wenshan | Yunnan | -3.00 (-5.09, -0.87) | 8.60 (-9.74, 30.67) | -6.47 (-12.69, 0.20) | -5.26 (-17.55, 8.86) |
| Wuding | Yunnan | -2.37 (-6.84, 2.31) | -22.86 (-46.63, 11.50) | 6.68 (-11.02, 27.90) | -10.58 (-36.59, 26.09) |
| Xianggelila | Yunnan | -0.70 (-3.08, 1.73) | -10.38 (-18.67, -1.24) | 2.75 (-8.26, 15.10) | 3.05 (-8.85, 16.50) |
| Xiangyun | Yunnan | -1.67 (-4.13, 0.86) | -3.53 (-20.21, 16.63) | -7.85 (-24.77, 12.89) | -5.14 (-14.28, 4.99) |
| Xichou | Yunnan | -0.36 (-3.65, 3.04) | 8.10 (-10.21, 30.13) | -7.80 (-15.91, 1.09) | -6.05 (-15.07, 3.93) |
| Ximeng | Yunnan | 6.85 (4.77, 8.97) | 10.77 (-1.59, 24.68) | -2.78 (-8.16, 2.92) | 13.30 (-6.80, 37.75) |
| Xinping | Yunnan | -1.42 (-3.58, 0.78) | -13.22 (-26.72, 2.76) | -0.23 (-11.27, 12.19) | 6.95 (-1.63, 16.27) |
| Xuanwei | Yunnan | -3.17 (-5.94, -0.32) | 1.43 (-25.93, 38.90) | -1.45 (-10.00, 7.92) | -5.29 (-20.35, 12.63) |
| Xundian | Yunnan | 2.36 (-0.25, 5.04) | -5.25 (-20.30, 12.65) | -0.81 (-15.30, 16.17) | 17.57 (-1.18, 39.88) |
| Yangbi | Yunnan | -0.24 (-3.91, 3.58) | -6.79 (-19.08, 7.36) | 12.73 (-12.91, 45.91) | -17.05 (-39.07, 12.93) |
| Yanjin | Yunnan | -0.89 (-2.73, 0.99) | -1.63 (-15.77, 14.87) | -3.97 (-8.18, 0.44) | 10.50 (-4.73, 28.16) |
| Yanshan | Yunnan | -0.21 (-2.35, 1.98) | -3.73 (-21.71, 18.37) | -7.24 (-14.12, 0.20) | -2.69 (-21.60, 20.78) |
| Yaoan | Yunnan | -2.45 (-4.16, -0.72) | -12.36 (-20.44, -3.46) | 3.46 (-0.02, 7.06) | -1.91 (-12.45, 9.89) |
| Yiliang | Yunnan | -2.77 (-5.21, -0.28) | -6.98 (-23.01, 12.38) | -5.59 (-11.99, 1.27) | 4.50 (-12.09, 24.22) |
| Yiliang | Yunnan | 2.79 (-0.77, 6.48) | -9.80 (-30.72, 17.43) | 14.56 (8.19, 21.29) | 23.48 (2.40, 48.91) |
| Yimen | Yunnan | -2.29 (-5.44, 0.96) | -11.42 (-39.86, 30.46) | -6.66 (-15.85, 3.55) | -8.78 (-28.40, 16.22) |
| Yingjiang | Yunnan | -0.96 (-3.75, 1.92) | -13.76 (-33.21, 11.34) | 6.06 (-6.58, 20.42) | 10.33 (-1.17, 23.17) |
| Yongde | Yunnan | -5.20 (-8.92, -1.33) | 13.92 (-13.27, 49.64) | -13.23 (-23.73, -1.28) | 17.34 (8.17, 27.27) |
| Yongping | Yunnan | -0.10 (-2.93, 2.81) | -4.65 (-17.43, 10.11) | -0.66 (-5.70, 4.66) | -3.73 (-32.06, 36.42) |
| Yongren | Yunnan | -2.32 (-3.92, -0.69) | 2.01 (-7.41, 12.38) | 2.62 (-9.30, 16.11) | -1.77 (-17.35, 16.75) |
| Yongshan | Yunnan | -3.48 (-6.11, -0.78) | 8.43 (0.57, 16.91) | -3.29 (-7.12, 0.70) | 15.22 (0.53, 32.05) |
| Yongsheng | Yunnan | 0.14 (-1.94, 2.27) | -1.38 (-23.06, 26.42) | 0.76 (-4.64, 6.46) | -0.84 (-13.94, 14.26) |
| Yuanjiang | Yunnan | -0.90 (-4.13, 2.43) | -17.13 (-39.55, 13.61) | 5.40 (-5.01, 16.94) | 7.63 (-3.62, 20.20) |
| Yuanmou | Yunnan | -1.27 (-4.53, 2.10) | -18.99 (-22.94, -14.84) | 4.06 (0.67, 7.57) | 5.25 (-4.70, 16.24) |
| Yuanyang | Yunnan | 0.65 (-1.54, 2.89) | -4.90 (-16.25, 8.00) | 3.29 (-6.59, 14.23) | 4.81 (-15.03, 29.27) |
| Yulong | Yunnan | -0.99 (-4.34, 2.49) | -9.23 (-39.4, 35.97) | 2.80 (-12.55, 20.84) | 6.27 (-16.78, 35.70) |
| Yun | Yunnan | -1.07 (-3.24, 1.15) | -9.41 (-20.00, 2.58) | 5.94 (3.03, 8.94) | -12.07 (-24.00, 1.75) |
| Yunlong | Yunnan | -3.66 (-6.76, -0.45) | -1.39 (-10.69, 8.88) | -12.63 (-26.88, 4.39) | -0.88 (-13.86, 14.06) |
| Zhanyi | Yunnan | -1.36 (-4.02, 1.38) | -17.17 (-34.10, 4.12) | 3.88 (-11.08, 21.36) | -0.65 (-7.18, 6.33) |
| Zhenkang | Yunnan | 0 (-3.91, 4.07) | 16.32 (5.00, 28.87) | -11.07 (-39.51, 30.74) | -1.08 (-22.15, 25.68) |
| Zhenxiong | Yunnan | 0.03 (-2.69, 2.83) | -3.22 (-18.85, 15.42) | 1.72 (-3.89, 7.65) | -2.97 (-20.74, 18.78) |
| Zhenyuan | Yunnan | 4.59 (1.67, 7.60) | 12.96 (-16.47, 52.76) | 6.93 (-2.99, 17.88) | 4.89 (-12.69, 26.01) |
| Anji | Zhejiang | -4.18 (-5.34, -3.00) | -4.84 (-12.09, 3.01) | -7.74 (-14.85, -0.04) | -9.24 (-17.93, 0.37) |
| Cangnan | Zhejiang | -7.80 (-9.73, -5.83) | -14.77 (-24.11, -4.27) | -3.17 (-15.53, 10.99) | -3.44 (-10.75, 4.46) |
| Changshan | Zhejiang | -1.95 (-4.20, 0.34) | 1.49 (-7.65, 11.53) | -0.75 (-12.31, 12.35) | 4.27 (-1.11, 9.94) |
| Changxing | Zhejiang | -6.55 (-8.07, -5.01) | -9.36 (-16.69, -1.39) | -6.76 (-18.30, 6.41) | -0.42 (-7.73, 7.47) |
| Chunan | Zhejiang | 1.19 (-0.78, 3.20) | -2.80 (-9.37, 4.25) | 12.24 (-1.67, 28.12) | -3.42 (-18.58, 14.56) |
| Cixi | Zhejiang | -4.79 (-6.58, -2.97) | 4.49 (-10.60, 22.14) | -8.33 (-13.25, -3.14) | 2.43 (-6.70, 12.44) |
| Daishan | Zhejiang | -4.08 (-6.03, -2.09) | -3.69 (-7.66, 0.46) | -0.32 (-11.84, 12.72) | -13.25 (-31.14, 9.30) |
| Deqing | Zhejiang | -5.56 (-7.71, -3.36) | -17.97 (-24.27, -11.15) | -2.89 (-14.15, 9.85) | -0.07 (-9.45, 10.27) |
| Dongtou | Zhejiang | -4.45 (-7.35, -1.45) | -4.37 (-15.66, 8.44) | 11.92 (-11.42, 41.42) | 1.88 (-11.72, 17.59) |
| Dongyang | Zhejiang | -1.22 (-2.61, 0.19) | 0.21 (-11.89, 13.97) | 3.91 (-8.56, 18.09) | -5.09 (-11.66, 1.95) |
| Fenghua | Zhejiang | -2.46 (-3.34, -1.56) | -1.00 (-7.19, 5.61) | -0.90 (-11.80, 11.34) | -1.19 (-4.66, 2.41) |
| Fuyang | Zhejiang | 1.88 (-0.73, 4.56) | 2.08 (-9.80, 15.52) | 8.74 (-14.49, 38.28) | -12.39 (-18.80, -5.47) |
| Haining | Zhejiang | -5.69 (-7.49, -3.84) | -8.54 (-20.50, 5.22) | -9.77 (-15.61, -3.53) | 3.06 (-3.02, 9.52) |
| Haiyan | Zhejiang | -3.83 (-5.90, -1.72) | -13.28 (-17.08, -9.31) | -3.05 (-14.89, 10.44) | -6.64 (-11.10, -1.96) |
| Jiande | Zhejiang | -1.36 (-3.16, 0.48) | -0.38 (-10.03, 10.30) | 7.18 (-2.03, 17.25) | -3.67 (-21.87, 18.75) |
| Jiangshan | Zhejiang | -5.16 (-7.01, -3.26) | -7.35 (-15.00, 0.98) | 8.63 (-0.87, 19.03) | 1.20 (-5.18, 8.02) |
| Jiashan | Zhejiang | -5.43 (-7.50, -3.31) | -2.31 (-10.21, 6.27) | -9.34 (-25.12, 9.76) | -1.25 (-5.23, 2.90) |
| Jingning | Zhejiang | -2.08 (-4.37, 0.27) | -9.10 (-15.02, -2.76) | -7.80 (-28.26, 18.50) | 1.25 (-16.35, 22.56) |
| Jinyun | Zhejiang | -2.77 (-4.82, -0.67) | -12.49 (-32.78, 13.92) | -0.58 (-7.40, 6.74) | 0.82 (-1.89, 3.61) |
| Kaihua | Zhejiang | -0.93 (-2.22, 0.38) | -5.87 (-10.38, -1.14) | -5.21 (-14.5, 5.08) | 1.82 (-6.57, 10.96) |
| Keqiao | Zhejiang | -6.42 (-7.91, -4.91) | -3.52 (-14.66, 9.08) | -3.15 (-14.04, 9.11) | -8.38 (-22.28, 8.00) |
| Lanxi | Zhejiang | 0.56 (-1.24, 2.39) | -9.62 (-21.88, 4.56) | 1.92 (-4.05, 8.26) | -1.69 (-7.30, 4.26) |
| Leqing | Zhejiang | -5.66 (-6.92, -4.39) | -5.66 (-9.56, -1.58) | 3.36 (-2.76, 9.88) | -9.58 (-14.81, -4.03) |
| Linan | Zhejiang | -1.43 (-2.51, -0.35) | -3.86 (-10.90, 3.75) | 1.15 (-9.34, 12.85) | 0.27 (-7.00, 8.10) |
| Linhai | Zhejiang | -5.14 (-6.36, -3.91) | -5.38 (-14.88, 5.18) | -2.98 (-10.6, 5.29) | 2.09 (-3.50, 8.01) |
| Longquan | Zhejiang | 0.71 (-1.61, 3.08) | -7.35 (-23.52, 12.25) | 11.38 (0.48, 23.47) | -8.66 (-12.87, -4.24) |
| Longyou | Zhejiang | -5.66 (-6.74, -4.56) | -7.86 (-14.65, -0.53) | 0.72 (-2.77, 4.34) | -1.49 (-7.39, 4.79) |
| Ninghai | Zhejiang | -5.85 (-7.88, -3.79) | 2.25 (-14.77, 22.66) | -2.38 (-17.19, 15.09) | -5.29 (-13.54, 3.74) |
| Panan | Zhejiang | -7.28 (-10.65, -3.79) | -19.78 (-35.74, 0.16) | -2.90 (-15.55, 11.65) | 2.01 (-14.0, 21.01) |
| Pinghu | Zhejiang | -5.80 (-8.54, -2.97) | -12.07 (-21.44, -1.59) | -0.15 (-19.42, 23.74) | -2.67 (-6.16, 0.96) |
| Pingyang | Zhejiang | -7.71 (-9.86, -5.50) | -6.79 (-18.47, 6.57) | -7.23 (-14.91, 1.14) | 6.98 (2.67, 11.47) |
| Pujiang | Zhejiang | -7.43 (-8.87, -5.96) | -2.08 (-14.54, 12.2) | -4.93 (-11.42, 2.05) | -10.41 (-23.30, 4.65) |
| Qingtian | Zhejiang | -3.98 (-5.46, -2.48) | -8.10 (-16.31, 0.92) | 3.08 (-6.17, 13.25) | -8.42 (-17.50, 1.65) |
| Qingyuan | Zhejiang | -2.68 (-5.05, -0.25) | -3.96 (-16.66, 10.68) | -4.45 (-21.51, 16.32) | -3.94 (-20.21, 15.66) |
| Ruian | Zhejiang | -4.61 (-5.65, -3.56) | -7.02 (-19.14, 6.92) | -6.35 (-11.92, -0.43) | -4.85 (-9.78, 0.35) |
| Sanmen | Zhejiang | -4.85 (-6.55, -3.12) | -8.70 (-15.97, -0.80) | -2.34 (-12.22, 8.66) | 1.05 (-9.66, 13.03) |
| Shangyu | Zhejiang | -5.33 (-6.59, -4.06) | -0.96 (-9.62, 8.53) | -1.84 (-7.83, 4.54) | -0.39 (-10.08, 10.36) |
| Shengsi | Zhejiang | -6.76 (-10.19, -3.20) | -10.41 (-30.77, 15.94) | -16.85 (-42.34, 19.92) | 7.61 (-14.02, 34.67) |
| Shengzhou | Zhejiang | -2.28 (-3.69, -0.86) | 2.42 (-3.86, 9.10) | -10.08 (-18.69, -0.55) | -1.73 (-6.10, 2.86) |
| Songyang | Zhejiang | -1.79 (-3.72, 0.18) | -5.29 (-21.32, 14.01) | 6.04 (-1.66, 14.34) | -3.91 (-20.60, 16.28) |
| Suichang | Zhejiang | -1.03 (-2.93, 0.92) | -6.52 (-19.77, 8.92) | 4.22 (-9.51, 20.03) | 1.19 (-13.93, 18.96) |
| Taishun | Zhejiang | -2.62 (-4.84, -0.35) | -4.16 (-22.61, 18.68) | 8.38 (-5.50, 24.29) | -7.50 (-14.78, 0.39) |
| Tiantai | Zhejiang | -4.26 (-6.15, -2.34) | -4.48 (-25.45, 22.40) | -3.93 (-12.16, 5.07) | -10.82 (-16.37, -4.90) |
| Tonglu | Zhejiang | -1.97 (-3.06, -0.87) | -3.94 (-12.64, 5.64) | 1.91 (-5.12, 9.47) | -0.06 (-9.54, 10.42) |
| Tongxiang | Zhejiang | -3.89 (-5.00, -2.77) | -0.69 (-12.23, 12.36) | -7.16 (-14.54, 0.86) | -4.31 (-10.96, 2.83) |
| Wencheng | Zhejiang | -8.78 (-10.61, -6.91) | -12.43 (-27.69, 6.04) | -3.83 (-18.36, 13.28) | -12.66 (-19.87, -4.80) |
| Wenling | Zhejiang | -6.58 (-9.37, -3.69) | -14.75 (-34.17, 10.40) | -1.88 (-15.33, 13.7) | 6.69 (5.95, 7.42) |
| Wuyi | Zhejiang | -5.05 (-6.51, -3.58) | -7.29 (-16.93, 3.48) | -4.75 (-13.76, 5.19) | -7.55 (-20.00, 6.85) |
| Xiangshan | Zhejiang | -0.48 (-1.93, 0.99) | -7.35 (-14.96, 0.94) | -2.85 (-5.63, 0.02) | -6.62 (-16.34, 4.23) |
| Xianju | Zhejiang | -0.65 (-2.05, 0.78) | -7.41 (-15.96, 2.00) | 0.67 (-6.83, 8.76) | 6.61 (-0.05, 13.71) |
| Xinchang | Zhejiang | -3.20 (-4.57, -1.82) | -5.85 (-17.30, 7.19) | -5.45 (-14.55, 4.61) | 2.15 (-6.82, 11.98) |
| Yiwu | Zhejiang | -4.35 (-7.90, -0.66) | -3.30 (-27.18, 28.41) | -6.74 (-29.23, 22.9) | 0.62 (-2.84, 4.20) |
| Yongjia | Zhejiang | -7.22 (-8.92, -5.49) | -15.36 (-22.75, -7.27) | -1.04 (-4.37, 2.42) | -4.92 (-12.25, 3.02) |
| Yongkang | Zhejiang | -2.78 (-4.76, -0.75) | -9.15 (-17.55, 0.11) | 1.19 (-7.81, 11.07) | -4.54 (-14.72, 6.85) |
| Yuhuan | Zhejiang | -10.88 (-12.64, -9.08) | -3.77 (-10.96, 4.00) | -14.55 (-22.83, -5.39) | -7.46 (-14.57, 0.23) |
| Yunhe | Zhejiang | -4.30 (-6.24, -2.33) | -2.30 (-22.25, 22.77) | -9.33 (-16.93, -1.03) | 3.26 (-5.88, 13.29) |
| Yuyao | Zhejiang | -5.79 (-6.89, -4.68) | -4.49 (-12.39, 4.12) | -6.41 (-8.37, -4.40) | 1.10 (-8.21, 11.35) |
| Zhuji | Zhejiang | -5.05 (-6.18, -3.92) | -2.90 (-12.52, 7.78) | -2.44 (-14.09, 10.79) | -5.17 (-10.04, -0.03) |

# Table S5. The estimated annual percentage change (EAPC) of urbanization score from 2005 to 2019

| **Name** | **Province** | **EAPC (%, 95% CI)** | | | |
| --- | --- | --- | --- | --- | --- |
|  |  | **2005-2019** | **2005-2009** | **2010-2014** | **2005-2019** |
| Changfeng | Anhui | 6.83 (6.09, 7.57) | 6.80 (2.29, 11.50) | 6.76 (-0.97, 15.09) | 4.21 (-0.48, 9.13) |
| Chaohu | Anhui | 4.31 (3.24, 5.39) | -1.30 (-6.48, 4.17) | 7.33 (2.04, 12.89) | 4.23 (-0.36, 9.03) |
| Dangshan | Anhui | 3.16 (2.50, 3.84) | 1.32 (-3.26, 6.12) | 3.12 (-0.30, 6.67) | 3.46 (-0.06, 7.10) |
| Dangtu | Anhui | 6.87 (5.56, 8.20) | 2.55 (0.12, 5.04) | 10.45 (0.05, 21.94) | 0.91 (-5.16, 7.36) |
| Dingyuan | Anhui | 3.17 (2.74, 3.59) | 2.13 (-2.00, 6.44) | 3.30 (-0.29, 7.02) | 2.88 (-0.50, 6.38) |
| Dongzhi | Anhui | 2.98 (2.10, 3.87) | 1.32 (-3.84, 6.76) | 2.95 (-2.83, 9.07) | 2.19 (-2.45, 7.06) |
| Fanchang | Anhui | 6.37 (4.93, 7.84) | 5.63 (0.70, 10.80) | 7.75 (-0.05, 16.15) | -0.30 (-6.68, 6.52) |
| Feidong | Anhui | 6.42 (5.34, 7.52) | 4.99 (0.30, 9.90) | 9.11 (-3.36, 23.20) | 5.32 (-0.71, 11.72) |
| Feixi | Anhui | 7.72 (6.80, 8.64) | 6.35 (0.95, 12.03) | 7.57 (-1.99, 18.06) | 5.05 (-2.10, 12.73) |
| Fengtai | Anhui | 2.53 (1.97, 3.09) | 0.61 (-1.85, 3.13) | 3.85 (-1.01, 8.95) | 2.56 (-2.95, 8.38) |
| Fengyang | Anhui | 3.98 (3.14, 4.83) | 2.04 (-3.63, 8.04) | 2.30 (-3.08, 7.97) | 7.05 (0.88, 13.60) |
| Funan | Anhui | 3.11 (2.24, 3.98) | 0.43 (-6.05, 7.36) | 2.66 (-0.49, 5.92) | 6.33 (3.12, 9.63) |
| Guangde | Anhui | 3.94 (2.90, 5.00) | 3.45 (-2.09, 9.30) | 6.01 (-1.44, 14.02) | 3.69 (-2.35, 10.10) |
| Guzhen | Anhui | 3.36 (2.51, 4.22) | 0.29 (-3.90, 4.67) | 5.29 (-0.37, 11.27) | 4.52 (-1.49, 10.89) |
| Hanshan | Anhui | 3.99 (3.29, 4.70) | 3.00 (0.89, 5.16) | 4.59 (-1.79, 11.39) | 3.37 (-0.32, 7.20) |
| He | Anhui | 4.90 (4.04, 5.76) | 2.97 (-1.74, 7.90) | 5.09 (-1.81, 12.47) | 3.74 (-1.08, 8.80) |
| Huaining | Anhui | 2.98 (2.29, 3.67) | 0.06 (-4.72, 5.08) | 3.58 (-0.87, 8.23) | 3.29 (-1.62, 8.44) |
| Huaiyuan | Anhui | 3.40 (2.53, 4.29) | 0.77 (-3.37, 5.09) | 3.16 (-1.11, 7.62) | 2.43 (-3.20, 8.39) |
| Huoqiu | Anhui | 2.83 (2.22, 3.44) | 2.95 (-4.39, 10.85) | 2.24 (-1.90, 6.55) | 2.91 (-0.93, 6.90) |
| Huoshan | Anhui | 2.22 (1.39, 3.06) | 2.31 (-2.29, 7.13) | 4.84 (0.62, 9.24) | -1.38 (-8.33, 6.10) |
| Jieshou | Anhui | 5.28 (4.25, 6.32) | -0.7 (-4.81, 3.59) | 7.46 (4.58, 10.42) | 5.22 (1.82, 8.72) |
| Jing | Anhui | 2.84 (2.03, 3.65) | 0.69 (-2.36, 3.83) | 4.92 (0.33, 9.72) | 1.19 (-4.71, 7.45) |
| Jingde | Anhui | 2.44 (1.36, 3.52) | -0.87 (-3.96, 2.33) | 2.79 (-2.80, 8.70) | 1.54 (-6.08, 9.78) |
| Jinzhai | Anhui | 3.09 (2.07, 4.13) | 1.07 (-4.00, 6.41) | 3.15 (-0.82, 7.28) | 5.30 (-0.58, 11.53) |
| Jixi | Anhui | 2.32 (1.55, 3.11) | 1.16 (-5.53, 8.32) | 3.66 (-1.25, 8.82) | 0.64 (-4.77, 6.35) |
| Laian | Anhui | 5.68 (4.57, 6.79) | 1.73 (-3.49, 7.23) | 7.20 (3.92, 10.58) | 9.20 (2.61, 16.21) |
| Langxi | Anhui | 3.88 (3.05, 4.71) | -0.18 (-1.45, 1.11) | 6.77 (2.62, 11.08) | 2.36 (-5.49, 10.87) |
| Lingbi | Anhui | 2.39 (1.79, 2.99) | 0.65 (-4.95, 6.59) | 3.51 (0.55, 6.55) | 2.24 (-2.33, 7.01) |
| Linquan | Anhui | 2.97 (2.02, 3.92) | 0.15 (-3.09, 3.50) | 1.33 (-0.59, 3.29) | 7.21 (2.21, 12.47) |
| Lixin | Anhui | 2.46 (1.84, 3.09) | 0.70 (-5.54, 7.36) | 1.23 (-1.17, 3.70) | 3.22 (-1.48, 8.14) |
| Lujiang | Anhui | 4.43 (3.23, 5.64) | 0.50 (-3.37, 4.53) | 5.72 (-0.95, 12.84) | 6.48 (1.27, 11.95) |
| Mengcheng | Anhui | 3.63 (2.77, 4.51) | -0.26 (-5.13, 4.86) | 5.43 (0.93, 10.13) | 1.12 (-3.19, 5.63) |
| Mingguang | Anhui | 4.99 (3.61, 6.40) | 1.34 (-5.14, 8.26) | 4.59 (-2.62, 12.32) | 6.93 (1.45, 12.70) |
| Nanling | Anhui | 5.20 (4.21, 6.20) | 2.82 (-0.18, 5.92) | 6.98 (1.49, 12.77) | 3.30 (-2.92, 9.91) |
| Ningguo | Anhui | 2.75 (1.80, 3.71) | 0.85 (-3.30, 5.18) | 5.07 (-0.98, 11.49) | 1.01 (-6.11, 8.68) |
| Qianshan | Anhui | 2.92 (2.26, 3.58) | 1.03 (-4.13, 6.48) | 3.51 (-0.56, 7.76) | 1.80 (-2.82, 6.65) |
| Qimen | Anhui | 1.57 (0.58, 2.56) | -0.64 (-3.51, 2.31) | 3.08 (-2.86, 9.39) | -0.81 (-6.80, 5.57) |
| Qingyang | Anhui | 3.26 (2.45, 4.09) | 1.09 (-1.20, 3.42) | 3.65 (-2.70, 10.42) | 2.73 (-3.23, 9.07) |
| Quanjiao | Anhui | 5.14 (4.46, 5.81) | 2.66 (-3.21, 8.90) | 4.49 (2.55, 6.46) | 6.80 (1.62, 12.24) |
| She | Anhui | 1.89 (1.04, 2.75) | 0.22 (-2.79, 3.33) | 2.97 (-2.68, 8.94) | 0.99 (-4.78, 7.10) |
| Shitai | Anhui | 2.69 (1.87, 3.53) | 1.76 (-3.32, 7.11) | 3.13 (-2.40, 8.98) | 0.62 (-4.85, 6.40) |
| Shou | Anhui | 2.44 (1.80, 3.10) | 1.72 (-4.32, 8.14) | 2.21 (-2.34, 6.97) | 3.41 (2.33, 4.50) |
| Shucheng | Anhui | 3.24 (2.39, 4.09) | 1.52 (-2.29, 5.48) | 3.34 (-2.13, 9.12) | 3.30 (-5.16, 12.52) |
| Si | Anhui | 3.36 (2.26, 4.47) | 1.06 (-4.18, 6.59) | 2.34 (-2.08, 6.96) | 8.32 (1.90, 15.15) |
| Suixi | Anhui | 3.60 (2.86, 4.35) | 3.30 (-1.54, 8.37) | 6.45 (-1.40, 14.92) | 2.28 (-2.37, 7.15) |
| Taihe | Anhui | 3.21 (2.42, 4.00) | 0.43 (-3.67, 4.70) | 1.06 (-3.10, 5.39) | 5.61 (2.84, 8.46) |
| Taihu | Anhui | 2.66 (1.98, 3.34) | 1.34 (-3.10, 6.00) | 2.85 (-1.15, 7.02) | 3.30 (-0.90, 7.67) |
| Tianchang | Anhui | 4.73 (3.86, 5.60) | 1.71 (-2.46, 6.06) | 8.31 (1.58, 15.48) | 2.07 (-2.51, 6.88) |
| Tongcheng | Anhui | 3.34 (2.73, 3.95) | 1.83 (-0.29, 3.99) | 5.34 (0.93, 9.93) | 3.96 (-0.30, 8.41) |
| Wangjiang | Anhui | 2.30 (1.67, 2.92) | 1.89 (-3.57, 7.66) | 0.62 (-1.08, 2.35) | 5.23 (-0.02, 10.76) |
| Woyang | Anhui | 2.82 (2.08, 3.56) | 1.02 (-4.09, 6.40) | 1.10 (-0.84, 3.08) | 6.36 (0.81, 12.21) |
| Wuhe | Anhui | 3.64 (2.94, 4.35) | 1.80 (-3.04, 6.87) | 4.22 (-0.34, 8.99) | 3.10 (-1.69, 8.11) |
| Wuhu | Anhui | 5.78 (4.70, 6.88) | 5.62 (-6.80, 19.70) | 8.03 (2.27, 14.12) | 2.19 (-3.14, 7.80) |
| Wuwei | Anhui | 3.56 (2.86, 4.26) | 2.22 (-0.29, 4.80) | 4.38 (-2.12, 11.31) | 2.95 (-2.74, 8.96) |
| Xiao | Anhui | 2.72 (2.06, 3.39) | 1.77 (-3.67, 7.51) | 3.49 (-0.33, 7.44) | 5.32 (0.71, 10.13) |
| Xiuning | Anhui | 2.10 (1.28, 2.92) | 0.76 (-3.73, 5.46) | 2.12 (-3.21, 7.74) | 2.13 (-3.15, 7.70) |
| Xiusong | Anhui | 2.33 (1.57, 3.09) | 1.14 (-4.46, 7.08) | 2.50 (-2.08, 7.29) | 3.67 (-1.98, 9.63) |
| Yi | Anhui | 1.20 (0.27, 2.14) | 0.74 (-4.29, 6.03) | 1.54 (-4.85, 8.36) | 0.84 (-5.05, 7.09) |
| Yian | Anhui | 5.76 (4.61, 6.92) | 0.72 (-3.35, 4.98) | 8.57 (0.60, 17.16) | 1.86 (-6.63, 11.14) |
| Yingshang | Anhui | 3.49 (2.75, 4.22) | 1.97 (-1.96, 6.07) | 2.91 (-2.74, 8.9) | 6.12 (3.13, 9.19) |
| Yuexi | Anhui | 2.76 (1.93, 3.60) | 1.26 (-2.37, 5.01) | 3.31 (-1.03, 7.84) | 2.52 (-4.34, 9.86) |
| Zongyang | Anhui | 1.82 (1.22, 2.42) | 0.99 (-3.26, 5.43) | 2.04 (-0.94, 5.10) | 1.59 (-4.62, 8.20) |
| Miyun | Beijing | 1.12 (-0.19, 2.45) | -4.52 (-13.36, 5.23) | 0.36 (-6.35, 7.54) | 2.29 (-3.58, 8.51) |
| Yanqing | Beijing | 1.05 (-0.62, 2.74) | -5.47 (-12.27, 1.86) | 0.00 (-7.76, 8.42) | 6.28 (-2.45, 15.79) |
| Bishan | Chongqing | 5.99 (4.49, 7.52) | -0.03 (-5.90, 6.20) | 13.46 (3.35, 24.56) | 1.77 (-4.90, 8.91) |
| Chengkou | Chongqing | 3.08 (2.55, 3.61) | 2.01 (-2.66, 6.90) | 4.38 (-0.57, 9.57) | 3.12 (0.36, 5.96) |
| Dazu | Chongqing | 5.54 (4.47, 6.62) | 0.49 (-6.66, 8.20) | 10.70 (6.74, 14.80) | 4.36 (2.10, 6.67) |
| Dianjiang | Chongqing | 3.51 (2.48, 4.56) | 0.88 (-7.92, 10.53) | 6.23 (-0.30, 13.19) | 4.10 (0.98, 7.31) |
| Fengdou | Chongqing | 4.03 (2.92, 5.16) | 1.15 (-5.39, 8.15) | 5.41 (1.38, 9.60) | 6.01 (2.91, 9.20) |
| Fengjie | Chongqing | 3.97 (3.11, 4.83) | 1.59 (-4.29, 7.84) | 5.22 (0.50, 10.15) | 4.59 (2.58, 6.63) |
| Hechuan | Chongqing | 3.77 (2.78, 4.76) | -0.10 (-5.00, 5.06) | 6.48 (-1.28, 14.86) | 2.18 (-0.61, 5.05) |
| Jiangjin | Chongqing | 4.90 (3.76, 6.05) | 1.42 (-4.38, 7.58) | 9.05 (0.29, 18.57) | 4.10 (0.92, 7.39) |
| Kaizhou | Chongqing | 3.73 (2.99, 4.47) | 2.04 (-2.80, 7.12) | 6.45 (2.13, 10.96) | 2.16 (0.58, 3.76) |
| Liangping | Chongqing | 4.24 (3.11, 5.37) | -0.04 (-7.18, 7.66) | 6.75 (1.21, 12.60) | 4.28 (-0.38, 9.16) |
| Nanchuan | Chongqing | 2.61 (1.87, 3.37) | 1.01 (-3.77, 6.03) | 2.89 (-1.64, 7.63) | 3.08 (-1.07, 7.40) |
| Pengshui | Chongqing | 3.54 (2.66, 4.43) | 1.73 (-4.25, 8.07) | 5.30 (0.64, 10.18) | 3.98 (0.53, 7.54) |
| Qijiang | Chongqing | 3.67 (2.68, 4.66) | 0.23 (-5.11, 5.86) | 7.33 (1.21, 13.82) | 3.36 (1.68, 5.06) |
| Rongchang | Chongqing | 4.75 (3.67, 5.83) | 2.55 (-4.67, 10.32) | 8.73 (0.52, 17.60) | 4.62 (1.26, 8.09) |
| Shizhu | Chongqing | 4.73 (3.79, 5.68) | 1.72 (-3.70, 7.44) | 6.59 (1.02, 12.46) | 4.21 (3.11, 5.32) |
| Tongliang | Chongqing | 5.47 (4.14, 6.81) | 0.45 (-6.39, 7.79) | 11.41 (4.04, 19.31) | 4.06 (1.63, 6.55) |
| Tongnan | Chongqing | 4.49 (3.54, 5.44) | 0.15 (-7.94, 8.94) | 7.17 (5.05, 9.32) | 4.10 (2.37, 5.87) |
| Wulong | Chongqing | 3.95 (3.11, 4.79) | 1.49 (-3.49, 6.72) | 5.16 (0.55, 9.99) | 3.69 (0.58, 6.89) |
| Wushan | Chongqing | 3.40 (2.53, 4.28) | 1.38 (-4.86, 8.03) | 2.72 (-0.38, 5.92) | 6.48 (3.40, 9.66) |
| Wuxi | Chongqing | 3.36 (2.61, 4.11) | 2.10 (-4.73, 9.41) | 3.37 (-1.32, 8.29) | 3.00 (0.70, 5.35) |
| Xiushan | Chongqing | 5.15 (4.21, 6.09) | 3.39 (-1.87, 8.93) | 7.65 (1.56, 14.11) | 6.14 (0.21, 12.42) |
| Yongchuan | Chongqing | 3.94 (2.88, 5.02) | -0.40 (-8.71, 8.66) | 7.53 (0.84, 14.68) | 2.87 (-1.90, 7.88) |
| Youyang | Chongqing | 3.99 (3.22, 4.75) | 3.28 (-4.43, 11.6) | 4.73 (0.49, 9.14) | 3.31 (-0.23, 6.97) |
| Yunyang | Chongqing | 3.58 (2.77, 4.40) | 0.53 (-5.74, 7.21) | 4.88 (0.41, 9.56) | 4.20 (-0.13, 8.72) |
| Zhong | Chongqing | 3.37 (2.51, 4.24) | 2.55 (-4.97, 10.66) | 5.00 (-0.27, 10.54) | 4.01 (0.40, 7.75) |
| Anxi | Fujian | 4.16 (3.16, 5.18) | -0.56 (-3.94, 2.93) | 6.33 (-1.02, 14.23) | 4.69 (-0.01, 9.61) |
| Changle | Fujian | 7.53 (5.60, 9.49) | -1.65 (-11.09, 8.79) | 17.01 (5.26, 30.08) | 6.53 (1.06, 12.30) |
| Changtai | Fujian | 7.82 (6.12, 9.54) | 0.91 (-3.79, 5.84) | 17.13 (6.41, 28.92) | 2.59 (-3.63, 9.22) |
| Changting | Fujian | 4.13 (3.34, 4.93) | 3.75 (-2.16, 10.03) | 4.60 (-0.64, 10.12) | 3.98 (-3.05, 11.52) |
| Datian | Fujian | 4.42 (3.58, 5.27) | 2.21 (-3.29, 8.03) | 6.51 (1.37, 11.91) | 1.09 (-5.84, 8.52) |
| Dehua | Fujian | 3.41 (2.36, 4.46) | -1.13 (-3.92, 1.74) | 7.46 (2.67, 12.47) | 1.38 (-5.88, 9.20) |
| Dongshan | Fujian | 6.63 (5.10, 8.17) | 1.01 (-10.95, 14.58) | 13.94 (3.25, 25.73) | 5.40 (-0.54, 11.71) |
| Fuan | Fujian | 4.36 (3.48, 5.26) | 2.35 (0.54, 4.18) | 8.69 (2.28, 15.5) | 1.86 (-4.50, 8.65) |
| Fuding | Fujian | 4.83 (3.70, 5.97) | 0.11 (-3.44, 3.80) | 10.60 (5.75, 15.67) | 0.72 (-5.86, 7.76) |
| Fuqing | Fujian | 4.80 (3.87, 5.73) | 0.29 (-5.89, 6.89) | 6.37 (-2.10, 15.57) | 6.44 (2.50, 10.52) |
| Guangze | Fujian | 3.79 (2.68, 4.92) | 0.51 (-2.41, 3.52) | 7.48 (0.58, 14.85) | 0.62 (-7.28, 9.20) |
| Gutian | Fujian | 3.45 (2.39, 4.52) | -0.39 (-4.85, 4.28) | 6.29 (-0.73, 13.80) | 0.25 (-6.88, 7.92) |
| Huaan | Fujian | 5.17 (4.08, 6.26) | 1.23 (-2.03, 4.60) | 8.89 (2.89, 15.24) | 3.36 (-4.70, 12.11) |
| Huian | Fujian | 4.36 (3.39, 5.33) | 0.11 (-5.37, 5.92) | 5.19 (0.19, 10.45) | 6.63 (1.87, 11.62) |
| Jiangle | Fujian | 3.54 (2.55, 4.54) | 0.51 (-3.81, 5.02) | 6.11 (-1.43, 14.22) | 1.15 (-7.41, 10.5) |
| Jianning | Fujian | 3.83 (2.92, 4.76) | 2.85 (-2.08, 8.04) | 4.83 (-2.43, 12.64) | 1.39 (-7.61, 11.27) |
| Jianou | Fujian | 3.95 (2.97, 4.94) | 1.95 (-2.02, 6.08) | 6.05 (-1.29, 13.94) | 0.96 (-6.14, 8.59) |
| Jianyang | Fujian | 4.08 (3.23, 4.94) | 1.57 (-2.47, 5.79) | 5.06 (-0.59, 11.04) | 1.47 (-5.65, 9.12) |
| Jinjiang | Fujian | -1.13 (-2.40, 0.16) | -2.30 (-10.93, 7.15) | -2.79 (-9.40, 4.30) | 3.19 (1.93, 4.47) |
| Liancheng | Fujian | 5.03 (4.11, 5.95) | 1.65 (-2.36, 5.81) | 6.30 (0.07, 12.92) | 3.77 (-5.48, 13.94) |
| Lianjiang | Fujian | 6.05 (4.95, 7.17) | 3.06 (-0.49, 6.73) | 11.82 (1.30, 23.43) | 3.96 (-0.87, 9.03) |
| Luoyuan | Fujian | 5.74 (4.61, 6.87) | 3.20 (-2.97, 9.76) | 10.48 (3.74, 17.66) | 1.16 (-6.60, 9.55) |
| Mingxi | Fujian | 3.51 (2.60, 4.42) | 1.34 (-1.73, 4.52) | 6.01 (-1.18, 13.72) | 2.93 (-4.36, 10.77) |
| Minhou | Fujian | 5.79 (4.48, 7.11) | 1.22 (-3.78, 6.49) | 11.63 (-0.71, 25.52) | 1.58 (-3.11, 6.50) |
| Minqing | Fujian | 4.17 (2.96, 5.39) | 0.73 (-1.21, 2.72) | 6.66 (-0.10, 13.88) | 6.44 (-1.47, 14.99) |
| Nanan | Fujian | 3.66 (2.99, 4.34) | 1.60 (-2.71, 6.11) | 6.56 (-0.19, 13.78) | 3.95 (0.99, 6.99) |
| Nanjing | Fujian | 4.73 (3.56, 5.92) | -0.02 (-5.31, 5.57) | 7.26 (-1.53, 16.83) | 4.77 (-2.61, 12.71) |
| Ninghua | Fujian | 3.62 (2.77, 4.47) | 3.36 (-1.94, 8.94) | 3.34 (-4.31, 11.61) | 3.60 (-4.45, 12.33) |
| Pinghe | Fujian | 3.90 (3.12, 4.69) | 1.12 (-3.00, 5.41) | 6.42 (2.08, 10.95) | 3.38 (-2.89, 10.05) |
| Pingnan | Fujian | 3.40 (2.53, 4.28) | 1.29 (0.00, 2.59) | 4.58 (-1.95, 11.55) | 1.37 (-7.71, 11.36) |
| Pingtan | Fujian | 6.92 (5.58, 8.28) | 3.75 (1.86, 5.68) | 14.97 (6.38, 24.25) | 6.28 (-5.72, 19.81) |
| Pucheng | Fujian | 3.91 (3.00, 4.84) | 1.95 (-2.02, 6.08) | 4.52 (-1.82, 11.27) | 1.54 (-6.21, 9.92) |
| Qingliu | Fujian | 4.32 (3.48, 5.17) | 3.04 (-2.71, 9.14) | 5.88 (0.88, 11.14) | 1.63 (-6.77, 10.78) |
| Sha | Fujian | 4.01 (2.99, 5.05) | 0.43 (-1.62, 2.51) | 7.76 (0.74, 15.28) | 0.49 (-7.42, 9.08) |
| Shanghang | Fujian | 5.14 (4.14, 6.15) | 1.26 (-3.84, 6.62) | 7.31 (1.21, 13.78) | 2.39 (-5.70, 11.18) |
| Shaowu | Fujian | 3.19 (1.81, 4.60) | -2.62 (-6.14, 1.03) | 6.36 (-1.58, 14.93) | -1.56 (-9.34, 6.89) |
| Shishi | Fujian | 1.01 (0.54, 1.49) | 2.02 (-4.64, 9.15) | 0.57 (-1.26, 2.45) | 0.33 (-1.43, 2.12) |
| Shouning | Fujian | 3.76 (2.97, 4.55) | 1.01 (-3.01, 5.20) | 3.79 (-2.03, 9.96) | 3.06 (-4.23, 10.90) |
| Shunchang | Fujian | 3.14 (2.14, 4.14) | -0.19 (-2.90, 2.59) | 3.87 (-2.52, 10.67) | 0.48 (-6.86, 8.39) |
| Songxi | Fujian | 3.50 (2.59, 4.41) | 1.35 (-2.05, 4.88) | 4.61 (-1.55, 11.15) | 2.96 (-3.45, 9.80) |
| Taining | Fujian | 2.89 (1.84, 3.95) | 1.46 (-1.03, 4.01) | 5.72 (-0.36, 12.18) | -1.61 (-10.7, 8.41) |
| Wuping | Fujian | 4.76 (3.82, 5.70) | 0.72 (-5.62, 7.48) | 5.81 (-0.48, 12.50) | 3.76 (-4.03, 12.20) |
| Wuyishan | Fujian | 3.82 (2.65, 5.00) | 0.62 (-2.32, 3.65) | 8.60 (1.06, 16.69) | 0.41 (-5.37, 6.54) |
| Xianyou | Fujian | 6.24 (4.97, 7.53) | 1.36 (-1.48, 4.29) | 11.76 (5.70, 18.15) | 1.40 (-3.11, 6.12) |
| Xiapu | Fujian | 3.48 (2.53, 4.43) | 0.26 (-2.17, 2.76) | 6.57 (2.06, 11.28) | 1.62 (-4.19, 7.79) |
| Yongan | Fujian | 2.84 (1.69, 4.01) | 0.29 (-3.18, 3.88) | 6.85 (-1.27, 15.64) | -1.85 (-9.55, 6.50) |
| Yongchun | Fujian | 4.43 (3.34, 5.53) | -0.85 (-3.79, 2.18) | 9.28 (1.39, 17.79) | 2.90 (-3.50, 9.74) |
| Yongding | Fujian | 3.14 (2.06, 4.22) | -0.59 (-3.12, 2.00) | 6.75 (-1.34, 15.50) | 2.21 (-4.79, 9.73) |
| Yongtai | Fujian | 4.77 (3.81, 5.73) | 3.34 (-1.82, 8.77) | 6.49 (-1.67, 15.34) | 5.48 (-3.21, 14.95) |
| Youxi | Fujian | 3.33 (2.32, 4.34) | 0.9 (-4.05, 6.11) | 5.41 (-1.90, 13.26) | -0.67 (-8.35, 7.65) |
| Yunxiao | Fujian | 5.26 (4.13, 6.40) | 2.34 (-3.12, 8.11) | 6.48 (0.05, 13.31) | 5.75 (0.61, 11.17) |
| Zhangping | Fujian | 4.13 (3.20, 5.06) | 1.86 (-2.81, 6.76) | 5.36 (-2.06, 13.35) | 1.61 (-7.02, 11.05) |
| Zhangpu | Fujian | 6.13 (4.88, 7.39) | 0.46 (-4.14, 5.27) | 11.74 (2.99, 21.24) | 6.35 (0.80, 12.20) |
| Zhaoan | Fujian | 4.71 (3.62, 5.81) | 0.16 (-6.08, 6.81) | 6.75 (1.22, 12.57) | 5.05 (0.84, 9.43) |
| Zhenghe | Fujian | 3.49 (2.54, 4.44) | 1.00 (-3.33, 5.53) | 4.66 (-0.96, 10.59) | 4.08 (-3.46, 12.20) |
| Zherong | Fujian | 2.93 (2.01, 3.85) | 2.91 (0.69, 5.19) | 4.24 (-2.56, 11.52) | 2.32 (-7.67, 13.39) |
| Zhouning | Fujian | 3.82 (3.06, 4.58) | 0.39 (-3.00, 3.91) | 4.31 (0.52, 8.23) | 3.52 (-4.63, 12.37) |
| Aksay | Gansu | 0.15 (-2.98, 3.37) | 4.05 (-4.11, 12.90) | 13.81 (2.93, 25.83) | -16.20 (-28.97, -1.14) |
| Cheng | Gansu | 1.52 (0.46, 2.59) | -1.40 (-10.92, 9.14) | 3.14 (-1.76, 8.29) | 1.22 (-3.08, 5.72) |
| Chongxin | Gansu | 3.50 (2.86, 4.15) | 2.32 (-3.38, 8.36) | 3.01 (-1.27, 7.47) | 4.52 (-0.95, 10.30) |
| Dangchang | Gansu | 4.41 (3.44, 5.38) | 1.33 (-5.40, 8.55) | 8.18 (5.76, 10.66) | 0.14 (-4.13, 4.60) |
| Diebu | Gansu | 3.04 (1.26, 4.85) | -4.31 (-14.83, 7.52) | 7.74 (3.54, 12.10) | -1.18 (-9.93, 8.42) |
| Dongxiangzu | Gansu | 3.53 (2.49, 4.58) | 5.19 (-1.38, 12.21) | 2.96 (-3.60, 9.96) | 8.91 (0.15, 18.44) |
| Dunhuang | Gansu | 1.55 (-0.51, 3.65) | 1.39 (-1.32, 4.18) | 9.19 (-0.93, 20.34) | -11.68 (-19.98, -2.51) |
| Gangu | Gansu | 2.87 (2.24, 3.50) | 3.15 (-2.93, 9.62) | 3.91 (-1.12, 9.19) | 3.67 (-0.90, 8.45) |
| Gaolan | Gansu | 9.34 (7.16, 11.57) | -0.58 (-4.89, 3.93) | 16.68 (4.83, 29.87) | 13.88 (6.44, 21.84) |
| Gaotai | Gansu | 5.08 (3.3, 6.88) | 0.48 (-3.46, 4.59) | 12.15 (3.35, 21.70) | -5.27 (-13.83, 4.13) |
| Guanghe | Gansu | 4.10 (3.13, 5.08) | 3.86 (-2.21, 10.29) | 3.47 (-4.18, 11.72) | 7.21 (2.26, 12.40) |
| Guazhou | Gansu | 2.49 (1.20, 3.79) | 1.40 (-3.58, 6.64) | 6.81 (-3.39, 18.09) | -0.92 (-13.80, 13.88) |
| Gulang | Gansu | 4.97 (3.53, 6.43) | 0.54 (-5.15, 6.57) | 5.64 (-1.21, 12.96) | 11.86 (0.48, 24.53) |
| Heshui | Gansu | 8.17 (6.85, 9.52) | 2.22 (-5.12, 10.12) | 8.48 (0.21, 17.43) | 1.50 (-2.52, 5.69) |
| Hezheng | Gansu | 2.76 (2.00, 3.52) | 2.70 (-3.60, 9.40) | 2.93 (-2.17, 8.29) | -0.33 (-6.75, 6.52) |
| Hezuo | Gansu | 2.64 (1.57, 3.73) | 4.47 (-2.75, 12.22) | 4.03 (-3.09, 11.68) | 1.04 (-5.66, 8.21) |
| Huachi | Gansu | 3.79 (2.31, 5.29) | 6.26 (1.52, 11.21) | 8.91 (-1.86, 20.86) | -2.69 (-11.32, 6.77) |
| Huan | Gansu | 9.04 (6.87, 11.25) | 6.23 (-4.75, 18.48) | 17.93 (2.89, 35.17) | 2.34 (-0.36, 5.11) |
| Huating | Gansu | 1.15 (0.54, 1.76) | 2.40 (-1.78, 6.75) | 1.41 (-4.85, 8.07) | 1.85 (-3.54, 7.54) |
| Hui | Gansu | 2.92 (2.03, 3.82) | 1.22 (-3.51, 6.19) | 3.40 (-1.54, 8.60) | 0.97 (-5.22, 7.55) |
| Huining | Gansu | 5.41 (4.53, 6.30) | 4.90 (1.18, 8.76) | 7.96 (-2.09, 19.04) | 4.48 (-2.98, 12.51) |
| Jingchuan | Gansu | 2.40 (1.54, 3.27) | -1.74 (-8.56, 5.60) | 4.79 (0.23, 9.56) | -0.41 (-3.66, 2.94) |
| Jingning | Gansu | 4.11 (3.19, 5.03) | 0.37 (-6.43, 7.66) | 3.95 (-3.34, 11.79) | 3.15 (-1.29, 7.79) |
| Jingtai | Gansu | 2.40 (1.29, 3.53) | 2.11 (-8.37, 13.78) | 8.74 (4.57, 13.08) | 0.96 (-5.93, 8.34) |
| Jingyuan | Gansu | 3.17 (2.31, 4.03) | 4.20 (-4.53, 13.73) | 0.83 (-6.30, 8.51) | 5.69 (2.24, 9.25) |
| Jinta | Gansu | 3.58 (1.82, 5.36) | 1.55 (-2.59, 5.86) | 11.89 (1.72, 23.08) | -6.50 (-13.86, 1.49) |
| Jishishan | Gansu | 2.81 (1.88, 3.75) | 3.12 (-3.20, 9.86) | 2.50 (-5.28, 10.91) | 3.14 (-2.52, 9.13) |
| Kang | Gansu | 3.13 (2.42, 3.84) | 1.89 (-4.02, 8.16) | 4.52 (1.22, 7.92) | -0.42 (-3.85, 3.14) |
| Kangle | Gansu | 3.08 (2.30, 3.86) | 2.96 (-2.89, 9.16) | 2.95 (-1.48, 7.59) | 4.18 (-0.75, 9.36) |
| Li | Gansu | 2.58 (1.82, 3.34) | 3.91 (-4.85, 13.47) | -0.61 (-4.31, 3.24) | 3.23 (0.38, 6.17) |
| Liangdang | Gansu | 1.62 (0.98, 2.26) | -1.18 (-4.45, 2.20) | 3.14 (1.60, 4.70) | -0.75 (-4.55, 3.21) |
| Lingtai | Gansu | 3.36 (2.57, 4.17) | 0.35 (-5.06, 6.07) | 3.77 (0.56, 7.10) | -0.91 (-3.71, 1.97) |
| Lintan | Gansu | 2.26 (1.75, 2.76) | 2.11 (-2.79, 7.25) | 1.31 (-2.66, 5.44) | 1.78 (-1.90, 5.59) |
| Lintao | Gansu | 3.28 (2.51, 4.05) | 4.73 (-0.04, 9.73) | 4.05 (-2.05, 10.53) | 5.95 (-0.45, 12.75) |
| Linxia | Gansu | 1.86 (-0.10, 3.86) | -6.94 (-18.07, 5.71) | 0.79 (-19.15, 25.65) | 1.78 (-3.31, 7.14) |
| Linxia | Gansu | 3.25 (2.61, 3.89) | 2.05 (-4.74, 9.31) | 3.75 (-0.41, 8.09) | 2.07 (-2.69, 7.06) |
| Linze | Gansu | 4.02 (2.76, 5.29) | 1.37 (-1.08, 3.88) | 8.09 (3.06, 13.37) | -3.09 (-12.88, 7.81) |
| Longxi | Gansu | 4.92 (3.69, 6.17) | 2.30 (-5.03, 10.19) | 4.75 (-9.19, 20.83) | 4.70 (-2.24, 12.12) |
| Luqu | Gansu | 1.51 (0.32, 2.72) | 1.88 (-6.31, 10.80) | 4.37 (-3.80, 13.22) | -1.50 (-11.00, 9.01) |
| Maqu | Gansu | 0.31 (-0.61, 1.25) | 0.08 (-4.95, 5.37) | 0.55 (-4.24, 5.58) | 0.76 (-6.20, 8.24) |
| Min | Gansu | 3.16 (2.40, 3.93) | 0.72 (-6.03, 7.94) | 2.63 (-2.81, 8.38) | 2.14 (-2.44, 6.94) |
| Minqin | Gansu | 5.16 (3.14, 7.23) | -1.05 (-5.49, 3.60) | 13.19 (3.98, 23.22) | -5.00 (-13.06, 3.82) |
| Minyue | Gansu | 4.38 (3.31, 5.45) | 4.24 (-3.04, 12.06) | 4.11 (-1.40, 9.93) | 0.55 (-7.85, 9.73) |
| Qinan | Gansu | 2.43 (1.82, 3.04) | 0.99 (-5.21, 7.60) | 2.01 (-1.41, 5.55) | 3.42 (-1.39, 8.46) |
| Qingcheng | Gansu | 0.40 (-0.73, 1.53) | -2.78 (-8.48, 3.27) | 2.78 (-11.66, 19.57) | 0.03 (-3.35, 3.53) |
| Qingshui | Gansu | 4.03 (3.40, 4.66) | 4.04 (-1.49, 9.88) | 4.23 (0.55, 8.05) | 1.40 (-4.05, 7.17) |
| Shandan | Gansu | 4.01 (2.31, 5.74) | -4.18 (-9.62, 1.59) | 8.29 (1.18, 15.90) | -3.20 (-12.06, 6.56) |
| Subei | Gansu | -0.24 (-4.90, 4.65) | 20.37 (13.75, 27.37) | 10.55 (-10.36, 36.33) | -11.37 (-24.92, 4.63) |
| Sunan | Gansu | 3.46 (1.39, 5.58) | 4.17 (-0.69, 9.28) | 10.40 (4.08, 17.12) | -9.34 (-15.38, -2.87) |
| Tianzhu | Gansu | 4.40 (3.05, 5.77) | -3.04 (-5.86, -0.14) | 6.99 (4.13, 9.92) | -1.59 (-10.58, 8.31) |
| Tongwei | Gansu | 4.60 (3.71, 5.51) | 2.25 (-4.39, 9.35) | 6.13 (1.06, 11.45) | 2.21 (-6.57, 11.82) |
| Weiyuan | Gansu | 3.19 (2.54, 3.83) | 2.75 (-1.17, 6.82) | 4.50 (-2.20, 11.67) | 2.73 (-2.13, 7.84) |
| Wen | Gansu | 1.79 (1.00, 2.58) | 1.21 (-4.87, 7.69) | -0.84 (-3.38, 1.77) | 3.53 (-3.20, 10.72) |
| Wushan | Gansu | 2.86 (2.17, 3.56) | 0.86 (-5.46, 7.60) | 4.96 (2.27, 7.73) | 1.29 (-2.86, 5.62) |
| Xiahe | Gansu | 3.21 (2.47, 3.96) | 2.13 (-4.19, 8.86) | 5.29 (1.02, 9.74) | 1.28 (-3.75, 6.56) |
| Xihe | Gansu | 2.35 (1.64, 3.06) | 1.73 (-6.68, 10.90) | 2.76 (-1.23, 6.91) | 3.24 (0.76, 5.77) |
| Yongchang | Gansu | 0.90 (-0.12, 1.93) | -4.21 (-7.22, -1.10) | 3.45 (-3.26, 10.63) | -0.29 (-9.58, 9.94) |
| Yongdeng | Gansu | 5.15 (3.71, 6.61) | -1.93 (-7.30, 3.76) | 7.43 (-6.12, 22.94) | 5.25 (-0.28, 11.09) |
| Yongjing | Gansu | 2.78 (1.57, 4.01) | 6.82 (-1.03, 15.30) | 2.59 (-10.92, 18.15) | 3.57 (-3.35, 10.99) |
| Yumen | Gansu | 0.34 (-0.85, 1.54) | -1.00 (-7.64, 6.13) | 0.87 (-7.48, 9.98) | 1.49 (-11.10, 15.86) |
| Yuzhong | Gansu | 4.70 (3.05, 6.37) | -1.22 (-6.88, 4.77) | 0.47 (-15.22, 19.06) | 11.38 (5.46, 17.64) |
| Zhang | Gansu | 2.24 (1.57, 2.92) | 1.68 (-3.20, 6.81) | 1.75 (-1.44, 5.04) | 3.84 (-1.57, 9.55) |
| Zhangjiachuan | Gansu | 3.04 (2.33, 3.75) | 2.11 (-5.84, 10.72) | 1.91 (-0.48, 4.36) | 0.78 (-3.10, 4.82) |
| Zhengning | Gansu | 2.33 (1.70, 2.96) | 0.43 (-3.94, 5.00) | 2.56 (-0.82, 6.05) | 0.56 (-5.10, 6.57) |
| Zhenyuan | Gansu | 5.68 (4.57, 6.80) | 0.67 (-9.22, 11.64) | 7.11 (0.52, 14.12) | 3.48 (-2.08, 9.35) |
| Zhouqu | Gansu | 2.56 (1.84, 3.28) | 2.21 (-2.18, 6.80) | 2.23 (-1.09, 5.67) | 4.35 (0.04, 8.83) |
| Zhuanglang | Gansu | 3.42 (2.69, 4.16) | -0.38 (-4.94, 4.40) | 5.17 (2.37, 8.05) | 3.07 (-1.46, 7.80) |
| Zhuoni | Gansu | 2.79 (1.85, 3.73) | 2.60 (-2.26, 7.70) | 1.94 (-2.95, 7.09) | 3.48 (-2.44, 9.77) |
| Boluo | Guangdong | 2.49 (1.11, 3.90) | -2.47 (-8.09, 3.48) | 7.60 (3.82, 11.51) | 1.99 (-2.44, 6.62) |
| Chaoan | Guangdong | 0.14 (-0.94, 1.24) | -2.70 (-10.85, 6.20) | -1.49 (-5.76, 2.98) | 3.42 (3.05, 3.79) |
| Conghua | Guangdong | 2.91 (1.54, 4.29) | -1.11 (-4.91, 2.84) | 8.79 (-0.95, 19.47) | -0.65 (-5.13, 4.05) |
| Dapu | Guangdong | 1.94 (0.85, 3.05) | -1.66 (-8.23, 5.38) | 2.76 (-4.43, 10.49) | 2.71 (-2.19, 7.86) |
| Deqing | Guangdong | 3.58 (2.59, 4.58) | 0.92 (-6.74, 9.22) | 5.93 (-1.88, 14.37) | 0.66 (-3.50, 5.01) |
| Dongyuan | Guangdong | 2.56 (1.79, 3.33) | 2.07 (-0.31, 4.52) | 3.90 (-2.53, 10.76) | 2.69 (-1.94, 7.53) |
| Enping | Guangdong | 2.21 (0.99, 3.44) | -2.55 (-8.37, 3.64) | 4.04 (-2.14, 10.61) | 3.54 (-1.77, 9.13) |
| Fengkai | Guangdong | 2.54 (1.70, 3.39) | 1.00 (-5.54, 7.99) | 3.88 (-2.60, 10.78) | -0.58 (-6.66, 5.90) |
| Fengshun | Guangdong | 2.20 (1.12, 3.30) | -1.66 (-7.86, 4.94) | 3.26 (-1.84, 8.62) | 2.86 (-1.43, 7.34) |
| Fogang | Guangdong | 1.51 (0.30, 2.74) | 6.08 (1.74, 10.61) | 0.66 (-10.81, 13.59) | 1.86 (-4.08, 8.17) |
| Gaoyao | Guangdong | 3.02 (1.99, 4.06) | 0.96 (-4.61, 6.85) | 8.92 (0.00, 18.63) | 0.70 (-3.06, 4.61) |
| Gaozhou | Guangdong | 2.72 (1.87, 3.59) | -0.33 (-4.74, 4.28) | 4.87 (0.04, 9.94) | 2.46 (-2.05, 7.17) |
| Guangning | Guangdong | 2.73 (1.95, 3.53) | 1.75 (-4.92, 8.88) | 3.98 (-3.28, 11.78) | 1.04 (-3.19, 5.45) |
| Haifeng | Guangdong | 4.39 (2.94, 5.87) | -1.94 (-8.50, 5.09) | 6.15 (0.57, 12.04) | 8.59 (5.29, 12.00) |
| Heping | Guangdong | 3.06 (2.16, 3.98) | 0.30 (-4.73, 5.59) | 4.90 (-0.54, 10.65) | 1.39 (-2.79, 5.74) |
| Heshan | Guangdong | 1.27 (-0.08, 2.64) | 2.59 (-6.72, 12.82) | 2.60 (-3.01, 8.54) | 2.63 (-0.53, 5.89) |
| Huaiji | Guangdong | 3.27 (0.53, 6.08) | 0.99 (-5.33, 7.73) | 3.22 (-2.69, 9.48) | -12.81 (-32.61, 12.82) |
| Huazhou | Guangdong | 2.67 (1.79, 3.55) | -0.62 (-6.38, 5.49) | 4.61 (-0.31, 9.77) | 3.46 (-1.13, 8.27) |
| Huidong | Guangdong | 3.14 (1.84, 4.46) | -0.93 (-7.60, 6.22) | 6.34 (-0.09, 13.18) | 1.52 (-4.15, 7.52) |
| Huilai | Guangdong | 3.98 (3.17, 4.80) | 1.39 (-4.20, 7.31) | 4.70 (0.51, 9.07) | 6.83 (3.37, 10.40) |
| Jiaoling | Guangdong | 1.98 (0.93, 3.05) | -0.62 (-4.83, 3.77) | 2.35 (-4.33, 9.49) | 3.29 (-1.79, 8.62) |
| Jiedong | Guangdong | 4.37 (3.56, 5.20) | 2.20 (-3.94, 8.72) | 7.54 (2.54, 12.79) | 2.06 (-1.07, 5.29) |
| Jiexi | Guangdong | 3.61 (2.70, 4.53) | 0.71 (-4.71, 6.44) | 5.55 (-0.49, 11.95) | 5.05 (1.64, 8.58) |
| Kaiping | Guangdong | 1.62 (0.02, 3.25) | -5.81 (-12.50, 1.40) | 6.39 (-0.63, 13.89) | 2.08 (-2.05, 6.40) |
| Lechang | Guangdong | 2.27 (1.46, 3.08) | -0.56 (-4.05, 3.07) | 2.19 (-3.33, 8.03) | 1.01 (-3.37, 5.59) |
| Leizhou | Guangdong | 2.70 (2.01, 3.39) | 1.80 (-3.55, 7.45) | 1.77 (-0.29, 3.87) | 4.32 (-1.52, 10.51) |
| Lianjiang | Guangdong | 4.81 (4.00, 5.63) | 2.52 (-3.56, 8.97) | 7.18 (0.63, 14.17) | 2.36 (-2.06, 6.98) |
| Liannan | Guangdong | 2.72 (1.81, 3.64) | 0.00 (-5.81, 6.17) | 4.24 (-3.67, 12.80) | 0.97 (-3.50, 5.65) |
| Lianping | Guangdong | 1.64 (1.05, 2.24) | 1.63 (-1.48, 4.85) | 2.15 (-3.13, 7.72) | 0.92 (-2.65, 4.62) |
| Lianshan | Guangdong | 2.04 (1.29, 2.80) | -0.86 (-5.58, 4.11) | 3.43 (-2.85, 10.12) | -0.24 (-5.11, 4.88) |
| Lianzhou | Guangdong | 2.15 (1.17, 3.13) | 0.32 (-2.85, 3.59) | 3.00 (-6.52, 13.49) | -2.08 (-6.74, 2.81) |
| Longchuan | Guangdong | 2.74 (1.88, 3.62) | 1.34 (-2.65, 5.49) | 2.99 (-3.63, 10.05) | 2.73 (-1.22, 6.84) |
| Longmen | Guangdong | 3.97 (2.92, 5.03) | 0.47 (-5.52, 6.85) | 6.43 (-0.30, 13.60) | -0.03 (-5.82, 6.12) |
| Lufeng | Guangdong | 5.20 (3.96, 6.45) | 0.92 (-6.51, 8.93) | 6.05 (0.50, 11.90) | 9.73 (7.68, 11.82) |
| Luhe | Guangdong | 3.64 (2.20, 5.11) | -1.01 (-5.52, 3.73) | 3.36 (-1.11, 8.04) | 9.60 (4.16, 15.33) |
| Luoding | Guangdong | 2.85 (2.07, 3.64) | 0.75 (-5.22, 7.10) | 4.83 (-0.11, 10.02) | 3.07 (0.11, 6.12) |
| Mei | Guangdong | 1.74 (0.53, 2.97) | -2.60 (-8.80, 4.03) | 4.03 (-1.20, 9.53) | 1.86 (-3.92, 7.98) |
| Nanao | Guangdong | 6.20 (4.83, 7.59) | -0.01 (-5.55, 5.86) | 7.75 (4.53, 11.06) | 7.08 (3.75, 10.52) |
| Nanxiong | Guangdong | 3.02 (2.11, 3.94) | 0.80 (-4.65, 6.56) | 4.94 (-1.33, 11.61) | -1.62 (-7.23, 4.33) |
| Pingyuan | Guangdong | 2.22 (1.53, 2.91) | 1.33 (-3.38, 6.26) | 4.19 (-0.56, 9.16) | 0.47 (-5.62, 6.96) |
| Puning | Guangdong | 5.04 (4.14, 5.95) | 0.09 (-8.71, 9.73) | 4.18 (0.85, 7.63) | 5.43 (2.38, 8.58) |
| Qingxin | Guangdong | 3.36 (2.45, 4.28) | 3.98 (-0.44, 8.60) | 4.20 (-1.58, 10.32) | 1.19 (-8.83, 12.31) |
| Raoping | Guangdong | 3.12 (2.24, 4.00) | -0.04 (-5.55, 5.79) | 5.00 (0.06, 10.17) | 3.37 (0.00, 6.86) |
| Renhua | Guangdong | 1.63 (0.74, 2.52) | 1.44 (-1.93, 4.94) | 2.19 (-5.64, 10.68) | -2.03 (-9.12, 5.61) |
| Ruyuan | Guangdong | 2.16 (1.17, 3.16) | -0.48 (-6.32, 5.72) | 3.85 (-4.22, 12.60) | 0.75 (-5.70, 7.63) |
| Shixing | Guangdong | 2.41 (1.41, 3.42) | -0.19 (-3.53, 3.26) | 4.84 (-2.83, 13.13) | -1.82 (-8.10, 4.88) |
| Sihui | Guangdong | 2.73 (1.63, 3.85) | 6.91 (3.68, 10.25) | 8.64 (1.57, 16.19) | 0.05 (-2.35, 2.51) |
| Suixi | Guangdong | 4.05 (3.32, 4.79) | 1.94 (-4.42, 8.71) | 6.03 (-0.48, 12.96) | 3.25 (-0.92, 7.59) |
| Taishan | Guangdong | 2.44 (1.67, 3.21) | 0.14 (-5.90, 6.58) | 3.36 (-0.66, 7.55) | 3.24 (-1.00, 7.65) |
| Wengyuan | Guangdong | 2.62 (1.80, 3.45) | 0.36 (-4.71, 5.71) | 4.44 (-1.28, 10.50) | 1.47 (-4.10, 7.38) |
| Wuchuan | Guangdong | 5.41 (4.44, 6.40) | 1.08 (-5.53, 8.16) | 8.05 (4.59, 11.62) | 6.77 (2.99, 10.70) |
| Wuhua | Guangdong | 2.92 (2.10, 3.74) | 1.10 (-4.77, 7.34) | 3.02 (-0.99, 7.18) | 4.40 (1.82, 7.04) |
| Xinfeng | Guangdong | 3.12 (2.11, 4.13) | 1.31 (-4.16, 7.11) | 6.55 (-1.39, 15.14) | -0.68 (-7.23, 6.33) |
| Xingning | Guangdong | 1.99 (1.26, 2.73) | 0.36 (-4.93, 5.94) | 2.15 (-1.75, 6.21) | 3.29 (1.01, 5.63) |
| Xinxing | Guangdong | 3.36 (2.63, 4.10) | 1.01 (-3.32, 5.55) | 5.11 (-0.05, 10.54) | 2.31 (-3.61, 8.60) |
| Xinyi | Guangdong | 2.84 (1.89, 3.79) | -1.08 (-6.23, 4.36) | 4.68 (-0.81, 10.48) | 0.88 (-5.76, 7.99) |
| Xuwen | Guangdong | 2.49 (1.90, 3.07) | 1.33 (-3.75, 6.68) | 2.58 (-0.57, 5.83) | 3.83 (0.06, 7.74) |
| Yangchun | Guangdong | 2.45 (1.66, 3.24) | 2.67 (-3.00, 8.68) | 6.03 (2.95, 9.21) | -1.24 (-5.75, 3.49) |
| Yangdong | Guangdong | 3.68 (2.68, 4.69) | 1.74 (-2.95, 6.65) | 8.70 (3.73, 13.90) | -0.99 (-6.47, 4.80) |
| Yangshan | Guangdong | 1.78 (1.23, 2.35) | 1.18 (-2.70, 5.21) | 0.81 (-4.50, 6.42) | 0.46 (-3.57, 4.66) |
| Yangxi | Guangdong | 3.46 (2.26, 4.67) | 0.48 (-6.38, 7.83) | 8.65 (1.02, 16.84) | -0.66 (-6.58, 5.63) |
| Yingde | Guangdong | 2.84 (2.14, 3.54) | 2.29 (-1.29, 6.00) | 3.60 (-3.22, 10.90) | 1.15 (-2.38, 4.82) |
| Yunan | Guangdong | 3.58 (2.57, 4.59) | 1.14 (-5.80, 8.59) | 6.43 (-3.33, 17.17) | 1.03 (-2.62, 4.82) |
| Yunan | Guangdong | 2.09 (1.20, 2.99) | -0.23 (-4.97, 4.75) | 3.90 (-2.94, 11.22) | 1.64 (-2.94, 6.43) |
| Zengcheng | Guangdong | -0.19 (-1.55, 1.18) | -6.71 (-16.97, 4.81) | 4.77 (0.47, 9.25) | -0.11 (-2.85, 2.71) |
| Zijin | Guangdong | 3.14 (2.32, 3.97) | 1.43 (-5.60, 8.99) | 3.85 (-2.93, 11.11) | 2.26 (-1.25, 5.90) |
| Bama | Guangxi | 2.27 (1.42, 3.13) | 2.48 (-2.37, 7.57) | 0.43 (-5.23, 6.42) | 3.35 (-1.43, 8.36) |
| Beiliu | Guangxi | 3.06 (2.44, 3.69) | 2.72 (-2.68, 8.43) | 2.74 (-2.34, 8.08) | 3.68 (0.32, 7.15) |
| Binyang | Guangxi | 4.42 (3.46, 5.38) | 2.46 (-2.45, 7.63) | 5.73 (-1.72, 13.74) | 3.26 (-1.25, 7.99) |
| Bobai | Guangxi | 2.56 (2.01, 3.10) | 2.85 (-2.04, 7.99) | 2.12 (-1.81, 6.21) | 2.01 (-1.98, 6.15) |
| Cangwu | Guangxi | 0.73 (-0.19, 1.66) | 3.02 (-2.74, 9.12) | -1.52 (-9.66, 7.35) | 2.66 (-1.23, 6.71) |
| Cenxi | Guangxi | 3.11 (2.35, 3.88) | 3.13 (-1.14, 7.59) | 3.79 (-0.95, 8.75) | 0.38 (-7.61, 9.05) |
| Dahua | Guangxi | 2.81 (1.83, 3.79) | 2.69 (-1.43, 6.98) | 3.04 (-4.07, 10.67) | 1.67 (-3.36, 6.95) |
| Daxin | Guangxi | 2.82 (1.97, 3.69) | 2.66 (-1.43, 6.92) | 3.47 (-3.18, 10.57) | -0.31 (-8.18, 8.22) |
| Debao | Guangxi | 3.19 (2.37, 4.01) | 4.85 (0.80, 9.07) | 2.53 (-3.45, 8.88) | 0.79 (-6.71, 8.90) |
| Donglan | Guangxi | 2.66 (1.79, 3.54) | 2.32 (-2.86, 7.78) | 1.20 (-4.20, 6.89) | 2.52 (-2.84, 8.17) |
| Dongxing | Guangxi | 3.07 (1.89, 4.26) | 1.62 (-0.72, 4.02) | 9.71 (0.53, 19.72) | -1.92 (-7.62, 4.14) |
| Douan | Guangxi | 2.49 (1.65, 3.34) | 1.96 (-2.96, 7.13) | 2.32 (-3.84, 8.88) | 4.01 (0.25, 7.91) |
| Fengshan | Guangxi | 2.12 (1.26, 2.99) | 1.72 (-3.19, 6.88) | 0.72 (-5.95, 7.87) | 1.91 (-2.50, 6.53) |
| Fuchuan | Guangxi | 2.97 (2.15, 3.79) | 0.11 (-2.36, 2.64) | 4.67 (-0.97, 10.63) | 2.85 (-3.04, 9.08) |
| Fusui | Guangxi | 2.99 (2.07, 3.91) | 1.12 (-2.49, 4.86) | 3.46 (-3.38, 10.79) | 1.05 (-6.92, 9.71) |
| Gongcheng | Guangxi | 2.37 (1.59, 3.17) | -0.25 (-3.62, 3.23) | 4.03 (-2.30, 10.77) | -0.10 (-6.28, 6.48) |
| Guanyang | Guangxi | 2.55 (1.71, 3.41) | 1.77 (-0.37, 3.96) | 3.42 (-2.42, 9.61) | -2.01 (-9.05, 5.58) |
| Guiping | Guangxi | 3.30 (2.63, 3.98) | 2.49 (-2.72, 7.97) | 3.60 (-1.92, 9.43) | 2.33 (-2.34, 7.22) |
| Heng | Guangxi | 4.52 (3.74, 5.29) | 4.02 (-1.75, 10.13) | 6.56 (0.57, 12.91) | 1.73 (-4.08, 7.88) |
| Hepu | Guangxi | 3.19 (2.52, 3.86) | 1.53 (-2.64, 5.88) | 3.79 (0.69, 6.99) | 2.61 (-2.51, 7.99) |
| Heshan | Guangxi | 1.13 (-0.23, 2.52) | -4.16 (-9.55, 1.55) | 9.02 (2.97, 15.43) | -3.50 (-7.40, 0.56) |
| Huanjiang | Guangxi | 2.22 (1.36, 3.08) | 0.94 (-4.17, 6.34) | 1.54 (-4.82, 8.31) | 2.09 (-3.43, 7.93) |
| Jingxi | Guangxi | 3.70 (2.61, 4.79) | 4.74 (0.03, 9.67) | 4.60 (-1.12, 10.66) | -0.89 (-12.58, 12.37) |
| Jinxiu | Guangxi | 2.57 (1.73, 3.43) | 0.48 (-4.46, 5.67) | 1.59 (-3.57, 7.03) | 2.85 (-4.23, 10.45) |
| Leye | Guangxi | 2.35 (1.43, 3.28) | 1.77 (-4.91, 8.92) | 1.75 (-5.14, 9.14) | 2.40 (-1.69, 6.67) |
| Lingchuan | Guangxi | 2.25 (1.42, 3.08) | 1.05 (-1.41, 3.58) | 4.93 (-1.11, 11.34) | -1.08 (-4.88, 2.87) |
| Lingshan | Guangxi | 2.68 (1.76, 3.62) | 1.30 (-4.84, 7.84) | 2.45 (-1.18, 6.21) | 3.29 (-1.90, 8.76) |
| Lingui | Guangxi | 3.86 (2.88, 4.85) | 2.62 (0.75, 4.52) | 8.15 (0.76, 16.08) | -1.28 (-5.78, 3.44) |
| Lingyun | Guangxi | 2.61 (1.82, 3.41) | 1.58 (-4.01, 7.48) | 1.52 (-3.90, 7.23) | 2.09 (-3.40, 7.91) |
| Lipu | Guangxi | 3.06 (2.07, 4.06) | 1.94 (-1.59, 5.60) | 4.77 (-3.01, 13.17) | -1.57 (-7.82, 5.11) |
| Liucheng | Guangxi | 3.47 (2.57, 4.37) | 2.20 (-1.98, 6.56) | 4.85 (-2.12, 12.31) | 1.56 (-4.40, 7.90) |
| Liujiang | Guangxi | 3.76 (2.85, 4.68) | 3.53 (-1.75, 9.09) | 5.34 (-3.40, 14.88) | 1.58 (-3.29, 6.70) |
| Longan | Guangxi | 3.26 (2.52, 4.01) | 3.47 (1.84, 5.14) | 3.41 (-3.21, 10.48) | 3.06 (-3.73, 10.33) |
| Longlin | Guangxi | 2.39 (1.57, 3.22) | 4.20 (-0.22, 8.82) | 2.39 (-3.12, 8.21) | 1.63 (-2.75, 6.21) |
| Longsheng | Guangxi | 2.44 (1.34, 3.55) | 2.25 (-1.61, 6.27) | 4.47 (-3.67, 13.30) | -3.01 (-10.49, 5.10) |
| Long州 | Guangxi | 3.53 (2.46, 4.61) | 1.44 (-2.00, 4.99) | 4.02 (-1.18, 9.50) | -1.29 (-11.18, 9.70) |
| Luchuan | Guangxi | 3.33 (2.61, 4.07) | 3.45 (-2.43, 9.70) | 4.98 (-1.06, 11.39) | 0.74 (-4.00, 5.71) |
| Luocheng | Guangxi | 1.94 (1.10, 2.79) | 0.59 (-3.86, 5.25) | 1.53 (-4.90, 8.40) | 2.50 (-2.64, 7.90) |
| Luzhai | Guangxi | 2.93 (2.09, 3.79) | 4.46 (1.50, 7.52) | 3.59 (-3.29, 10.97) | 1.44 (-5.17, 8.52) |
| Mashan | Guangxi | 3.25 (2.48, 4.01) | 2.80 (-2.15, 7.99) | 2.08 (-3.99, 8.53) | 4.58 (-0.92, 10.37) |
| Mengshan | Guangxi | 2.32 (1.53, 3.12) | 0.81 (-2.08, 3.79) | 2.67 (-2.52, 8.14) | -1.17 (-9.02, 7.36) |
| Nandan | Guangxi | 1.69 (0.68, 2.72) | -0.30 (-6.51, 6.34) | 1.05 (-7.29, 10.14) | 0.60 (-6.53, 8.29) |
| Napo | Guangxi | 3.22 (2.27, 4.19) | 1.14 (-4.33, 6.93) | 3.07 (-0.96, 7.27) | 2.93 (-1.60, 7.66) |
| Ningming | Guangxi | 3.40 (2.48, 4.33) | 2.35 (-3.46, 8.50) | 3.60 (-2.29, 9.85) | -0.45 (-8.83, 8.70) |
| Pingle | Guangxi | 2.46 (1.71, 3.22) | 0.53 (-2.88, 4.06) | 3.85 (-1.83, 9.86) | 0.36 (-5.19, 6.24) |
| Pingnan | Guangxi | 3.55 (2.75, 4.35) | 2.14 (-3.63, 8.26) | 3.82 (-1.08, 8.95) | 2.87 (-3.12, 9.22) |
| Pingxiang | Guangxi | 2.96 (1.83, 4.10) | -0.80 (-7.40, 6.28) | 3.36 (-2.16, 9.18) | 1.55 (-6.66, 10.48) |
| Pingguo | Guangxi | 2.27 (1.16, 3.39) | 0.56 (-2.76, 4.00) | 4.73 (-3.10, 13.19) | 0.55 (-5.99, 7.54) |
| Pubei | Guangxi | 2.99 (2.23, 3.76) | 2.45 (-1.50, 6.56) | 3.24 (-1.79, 8.53) | 1.98 (-5.65, 10.23) |
| Quanzhou | Guangxi | 2.27 (1.57, 2.98) | 1.09 (-2.32, 4.63) | 2.99 (-2.60, 8.91) | 0.15 (-5.17, 5.77) |
| Rong | Guangxi | 3.26 (2.40, 4.12) | 2.90 (-1.40, 7.39) | 3.58 (-3.18, 10.81) | 0.81 (-5.42, 7.46) |
| Rongan | Guangxi | 2.59 (1.75, 3.44) | 2.51 (-1.50, 6.69) | 2.44 (-2.38, 7.50) | 1.59 (-7.22, 11.23) |
| Rongshui | Guangxi | 3.33 (2.44, 4.22) | 2.65 (-1.49, 6.96) | 3.33 (-2.91, 9.98) | 2.30 (-3.44, 8.38) |
| Sanjiang | Guangxi | 3.27 (2.48, 4.08) | 3.66 (-2.34, 10.03) | 1.86 (-4.16, 8.26) | 3.30 (-1.43, 8.25) |
| Shanglin | Guangxi | 3.72 (2.95, 4.49) | 2.44 (-1.34, 6.35) | 3.77 (-2.59, 10.54) | 3.06 (-2.38, 8.81) |
| Shangsi | Guangxi | 2.86 (1.96, 3.77) | 2.36 (-3.39, 8.45) | 3.56 (-2.74, 10.26) | -0.97 (-8.76, 7.49) |
| Teng | Guangxi | 3.20 (2.39, 4.02) | 2.67 (-2.03, 7.59) | 4.06 (-3.15, 11.81) | 1.09 (-5.99, 8.70) |
| Tiandeng | Guangxi | 2.63 (1.89, 3.37) | 4.26 (0.10, 8.60) | 2.15 (-4.64, 9.42) | 1.72 (-3.71, 7.45) |
| Tiandong | Guangxi | 4.08 (3.22, 4.94) | 1.81 (-3.01, 6.88) | 4.48 (-1.42, 10.73) | 1.37 (-6.93, 10.40) |
| Tiane | Guangxi | 1.41 (0.01, 2.82) | 5.45 (2.35, 8.65) | -0.79 (-10.24, 9.64) | -0.51 (-7.65, 7.19) |
| Tianlin | Guangxi | 2.90 (1.84, 3.98) | 3.25 (-1.10, 7.79) | 0.75 (-4.58, 6.38) | 3.40 (-5.67, 13.35) |
| Tianyang | Guangxi | 3.85 (2.48, 5.24) | 0.95 (-4.00, 6.15) | 6.61 (-1.90, 15.87) | -0.22 (-9.01, 9.42) |
| Wuming | Guangxi | 5.31 (4.15, 6.48) | 3.28 (-0.23, 6.91) | 8.41 (-0.14, 17.69) | -0.63 (-8.62, 8.06) |
| Wuxuan | Guangxi | 3.66 (2.91, 4.42) | 3.14 (-1.27, 7.74) | 6.12 (1.09, 11.40) | 0.80 (-5.31, 7.30) |
| Xiangzhou | Guangxi | 3.90 (3.06, 4.75) | 4.09 (-0.14, 8.51) | 4.64 (-1.06, 10.68) | 0.25 (-7.37, 8.50) |
| Xilin | Guangxi | 2.82 (2.05, 3.60) | 2.97 (-0.93, 7.02) | 2.83 (-2.26, 8.19) | 1.47 (-2.58, 5.69) |
| Xincheng | Guangxi | 3.01 (2.06, 3.97) | 1.99 (-2.10, 6.25) | 3.99 (-3.45, 12.00) | -0.21 (-7.08, 7.17) |
| Xingan | Guangxi | 1.65 (0.74, 2.57) | 0.27 (-1.59, 2.16) | 4.46 (-2.81, 12.27) | -2.13 (-8.24, 4.38) |
| Xingye | Guangxi | 3.32 (2.57, 4.07) | 3.45 (-1.64, 8.81) | 3.25 (-2.31, 9.12) | 2.09 (-3.02, 7.46) |
| Yangshuo | Guangxi | 3.33 (2.62, 4.06) | 2.67 (-0.27, 5.70) | 3.52 (-3.13, 10.64) | 0.13 (-5.49, 6.08) |
| Yizhou | Guangxi | 2.60 (1.53, 3.69) | 0.26 (-3.65, 4.33) | 3.33 (-3.96, 11.16) | 1.38 (-4.55, 7.69) |
| Yongfu | Guangxi | 2.43 (1.40, 3.47) | 0.93 (-0.93, 2.82) | 4.53 (-3.71, 13.47) | -2.92 (-10.72, 5.55) |
| Zhaoping | Guangxi | 2.01 (1.22, 2.80) | -0.17 (-5.41, 5.37) | 2.44 (-3.10, 8.29) | 2.04 (-2.66, 6.96) |
| Zhongshan | Guangxi | 2.43 (1.37, 3.49) | -0.73 (-4.70, 3.40) | 2.42 (-4.80, 10.18) | 0.77 (-4.31, 6.12) |
| Ziyuan | Guangxi | 2.82 (1.93, 3.72) | 1.55 (-2.78, 6.07) | 4.31 (-2.11, 11.15) | -0.75 (-7.99, 7.05) |
| Anlong | Guizhou | 5.06 (3.88, 6.26) | 1.94 (-3.98, 8.23) | 5.88 (-0.50, 12.67) | 2.59 (-5.51, 11.38) |
| Bozhou | Guizhou | 6.52 (4.89, 8.17) | 0.74 (-4.40, 6.15) | 10.29 (-0.38, 22.09) | 2.11 (-6.73, 11.79) |
| Ceheng | Guizhou | 4.84 (3.62, 6.08) | 2.29 (-5.47, 10.68) | 4.04 (-2.40, 10.91) | 3.71 (-2.24, 10.02) |
| Cen巩 | Guizhou | 5.36 (3.90, 6.84) | 1.45 (-3.35, 6.47) | 6.53 (1.48, 11.83) | 2.45 (-9.70, 16.24) |
| Changshun | Guizhou | 5.77 (4.44, 7.11) | 1.27 (-4.58, 7.47) | 8.08 (2.91, 13.51) | 7.71 (1.51, 14.29) |
| Chishui | Guizhou | 5.60 (3.97, 7.25) | 0.82 (-4.98, 6.97) | 8.49 (-0.27, 18.02) | 1.61 (-8.31, 12.60) |
| Congjiang | Guizhou | 3.87 (3.04, 4.71) | 1.91 (-5.74, 10.19) | 4.01 (2.14, 5.92) | 3.53 (-1.49, 8.79) |
| Dafang | Guizhou | 6.27 (5.00, 7.56) | 3.06 (-3.75, 10.35) | 9.98 (1.95, 18.64) | 2.13 (-5.18, 9.99) |
| Danzhai | Guizhou | 5.32 (4.06, 6.59) | 2.32 (-0.71, 5.45) | 7.86 (1.35, 14.79) | 3.77 (-5.20, 13.59) |
| Daozhen | Guizhou | 4.54 (3.15, 5.95) | 1.41 (-7.55, 11.24) | 2.93 (-1.39, 7.44) | 4.19 (-5.41, 14.77) |
| Dejiang | Guizhou | 6.42 (5.12, 7.73) | 1.63 (-3.40, 6.91) | 9.36 (4.34, 14.62) | 5.21 (-2.38, 13.40) |
| Douyun | Guizhou | 4.15 (2.61, 5.73) | 1.26 (-2.03, 4.67) | 3.03 (-4.13, 10.73) | -0.22 (-4.12, 3.84) |
| Dushan | Guizhou | 5.26 (4.02, 6.52) | 1.15 (-4.44, 7.07) | 6.19 (-1.23, 14.16) | 4.73 (-1.21, 11.02) |
| Fenggang | Guizhou | 4.73 (3.59, 5.89) | 1.57 (-2.84, 6.18) | 6.78 (1.62, 12.19) | 4.16 (-1.59, 10.24) |
| Fuquan | Guizhou | 4.72 (3.28, 6.19) | -1.25 (-7.02, 4.87) | 9.43 (2.36, 16.99) | 2.35 (-4.15, 9.30) |
| Guanling | Guizhou | 4.85 (3.76, 5.96) | 1.39 (-4.20, 7.31) | 7.15 (2.81, 11.68) | 0.82 (-4.22, 6.13) |
| Guiding | Guizhou | 4.41 (2.93, 5.92) | -0.36 (-5.70, 5.29) | 8.04 (0.00, 16.72) | 0.56 (-5.64, 7.17) |
| Hezhang | Guizhou | 4.84 (3.90, 5.78) | 1.75 (-4.05, 7.90) | 5.86 (2.22, 9.62) | 2.78 (-3.56, 9.54) |
| Huangping | Guizhou | 5.04 (3.64, 6.45) | 2.47 (-0.77, 5.81) | 6.94 (-1.68, 16.31) | 2.78 (-5.88, 12.23) |
| Huishui | Guizhou | 4.77 (3.61, 5.94) | 1.18 (-4.43, 7.12) | 7.15 (-0.07, 14.90) | 4.09 (-3.11, 11.83) |
| Jiangkou | Guizhou | 4.98 (3.62, 6.36) | 1.01 (-4.33, 6.65) | 7.03 (-0.94, 15.64) | 2.64 (-5.13, 11.05) |
| Jianhe | Guizhou | 4.69 (3.59, 5.80) | 1.94 (-5.85, 10.38) | 5.69 (0.24, 11.43) | 3.08 (-3.70, 10.34) |
| Jinping | Guizhou | 4.59 (3.47, 5.72) | 2.21 (-3.07, 7.78) | 4.95 (0.97, 9.08) | 2.99 (-4.28, 10.81) |
| Jinsha | Guizhou | 5.16 (4.02, 6.32) | 2.61 (-5.14, 10.99) | 9.15 (2.22, 16.55) | 0.34 (-6.25, 7.40) |
| Kaili | Guizhou | 5.38 (3.47, 7.33) | -2.03 (-4.94, 0.97) | 12.67 (0.36, 26.50) | 1.60 (-5.15, 8.83) |
| Kaiyang | Guizhou | 5.01 (3.66, 6.37) | 3.13 (0.12, 6.23) | 9.21 (0.53, 18.64) | -1.09 (-9.16, 7.68) |
| Leishan | Guizhou | 4.93 (3.68, 6.19) | 2.16 (-5.07, 9.94) | 4.75 (-0.30, 10.05) | 4.95 (-3.77, 14.46) |
| Libo | Guizhou | 5.21 (3.78, 6.67) | 5.01 (1.49, 8.65) | 7.06 (-5.22, 20.95) | 1.12 (-7.12, 10.10) |
| Liping | Guizhou | 4.21 (3.05, 5.39) | 2.19 (-3.77, 8.51) | 2.04 (-0.72, 4.87) | 5.11 (-4.37, 15.54) |
| Liuzhite | Guizhou | 5.66 (4.25, 7.09) | 2.72 (-3.51, 9.35) | 11.03 (1.77, 21.13) | -1.20 (-5.27, 3.04) |
| Longli | Guizhou | 7.48 (5.56, 9.43) | 0.86 (-4.56, 6.58) | 12.42 (-1.09, 27.78) | 9.25 (-1.28, 20.90) |
| Luodian | Guizhou | 4.99 (3.72, 6.27) | 1.79 (-3.01, 6.82) | 6.71 (-1.21, 15.27) | 1.65 (-5.40, 9.22) |
| Majiang | Guizhou | 5.31 (4.15, 6.49) | 2.76 (-3.66, 9.62) | 6.01 (-0.62, 13.07) | 3.84 (-3.06, 11.24) |
| Meitan | Guizhou | 4.71 (3.18, 6.26) | -0.24 (-8.90, 9.24) | 6.03 (-0.50, 12.99) | 4.00 (-5.82, 14.83) |
| Nayong | Guizhou | 4.12 (2.99, 5.25) | 0.86 (-5.46, 7.59) | 7.28 (2.76, 12.00) | 1.60 (-5.16, 8.85) |
| Panzhou | Guizhou | 5.09 (3.79, 6.41) | 1.19 (-6.01, 8.95) | 9.49 (1.99, 17.55) | 0.12 (-7.56, 8.43) |
| Pingba | Guizhou | 7.69 (5.59, 9.83) | 1.88 (-2.89, 6.89) | 10.95 (-0.72, 23.98) | 1.22 (-8.90, 12.47) |
| Pingtang | Guizhou | 4.99 (3.62, 6.38) | 2.21 (-2.45, 7.09) | 5.24 (-1.52, 12.48) | 4.95 (-3.83, 14.52) |
| Puan | Guizhou | 4.65 (3.58, 5.74) | 2.21 (-4.37, 9.25) | 4.82 (0.91, 8.88) | 2.98 (-3.04, 9.37) |
| Puding | Guizhou | 4.89 (3.59, 6.22) | 0.35 (-6.33, 7.51) | 6.90 (-0.50, 14.85) | 3.74 (-2.56, 10.44) |
| Qianxi | Guizhou | 4.97 (4.07, 5.87) | 3.05 (-3.88, 10.47) | 6.49 (1.31, 11.93) | 3.18 (-2.38, 9.05) |
| Qinglong | Guizhou | 4.88 (3.74, 6.03) | 2.08 (-4.66, 9.29) | 5.00 (0.74, 9.45) | 3.57 (-3.32, 10.94) |
| Qingzhen | Guizhou | 6.19 (4.58, 7.82) | 1.03 (-3.63, 5.93) | 12.08 (1.06, 24.31) | 0.35 (-4.74, 5.72) |
| Qixingguan | Guizhou | 5.98 (4.17, 7.81) | 0.73 (-4.26, 5.98) | 8.04 (-1.21, 18.15) | 0.83 (-8.54, 11.16) |
| Renhuai | Guizhou | 7.81 (6.40, 9.24) | 4.58 (-2.86, 12.59) | 12.55 (0.99, 25.43) | 7.72 (-3.62, 20.39) |
| Rongjiang | Guizhou | 3.94 (2.77, 5.13) | 2.04 (-4.85, 9.42) | 4.26 (-4.17, 13.43) | 2.41 (-2.63, 7.70) |
| Sandou | Guizhou | 4.32 (3.16, 5.49) | 1.39 (-4.65, 7.81) | 5.19 (-1.20, 11.99) | 4.22 (-1.44, 10.21) |
| Sansui | Guizhou | 5.34 (3.99, 6.70) | 0.70 (-4.41, 6.08) | 7.18 (3.07, 11.44) | 3.87 (-4.80, 13.35) |
| Shibing | Guizhou | 3.67 (2.78, 4.57) | 3.12 (-3.27, 9.93) | 5.05 (-3.09, 13.87) | 1.72 (-1.27, 4.80) |
| Shiqian | Guizhou | 5.16 (3.80, 6.54) | 0.70 (-5.67, 7.51) | 7.08 (0.60, 13.98) | 3.44 (-2.32, 9.54) |
| Shuicheng | Guizhou | 4.94 (3.77, 6.12) | 2.27 (-5.36, 10.52) | 8.11 (-0.04, 16.92) | 2.73 (-4.83, 10.89) |
| Sinan | Guizhou | 4.82 (3.65, 5.99) | 1.23 (-3.58, 6.27) | 4.97 (-0.30, 10.52) | 4.08 (-1.38, 9.84) |
| Songtao | Guizhou | 5.20 (4.18, 6.23) | 2.20 (-2.12, 6.71) | 6.59 (0.84, 12.67) | 3.32 (-4.41, 11.68) |
| Suiyang | Guizhou | 4.83 (3.66, 6.02) | 0.91 (-6.14, 8.49) | 5.92 (-0.69, 12.98) | 2.63 (-2.64, 8.18) |
| Taijiang | Guizhou | 4.14 (3.04, 5.25) | 2.03 (-4.29, 8.78) | 6.21 (-1.16, 14.14) | 2.41 (-4.18, 9.46) |
| Tianzhu | Guizhou | 4.82 (3.51, 6.14) | 1.78 (-2.78, 6.55) | 5.98 (-1.74, 14.31) | 0.30 (-7.89, 9.22) |
| Tongzi | Guizhou | 4.08 (2.79, 5.39) | -0.05 (-9.09, 9.88) | 7.02 (0.60, 13.85) | 1.05 (-4.83, 7.30) |
| Wangmo | Guizhou | 5.18 (3.69, 6.69) | 2.62 (-4.40, 10.16) | 4.70 (-2.51, 12.45) | 3.79 (-4.15, 12.39) |
| Weining | Guizhou | 4.74 (3.69, 5.79) | 2.82 (-2.42, 8.35) | 7.65 (1.65, 13.99) | 1.51 (-3.58, 6.86) |
| Wengan | Guizhou | 5.28 (3.93, 6.65) | 2.06 (-3.07, 7.46) | 6.91 (-0.50, 14.88) | 1.83 (-5.47, 9.69) |
| Wuchuan | Guizhou | 5.53 (3.94, 7.14) | 2.89 (-2.17, 8.21) | 4.25 (-2.06, 10.96) | 4.44 (-9.41, 20.41) |
| Xifeng | Guizhou | 5.31 (3.61, 7.04) | 2.76 (-0.81, 6.46) | 12.19 (2.53, 22.76) | -4.05 (-11.71, 4.28) |
| Xingren | Guizhou | 5.10 (4.37, 5.84) | 3.21 (-3.33, 10.2) | 6.38 (0.48, 12.63) | 3.40 (-1.80, 8.87) |
| Xingyi | Guizhou | 4.95 (3.29, 6.63) | -0.09 (-6.09, 6.30) | 10.87 (-5.87, 30.59) | -1.68 (-6.63, 3.52) |
| Xishui | Guizhou | 5.15 (4.06, 6.24) | 2.72 (-4.69, 10.70) | 6.08 (0.14, 12.37) | 2.84 (-4.02, 10.20) |
| Xiuwen | Guizhou | 6.21 (4.83, 7.60) | 2.38 (-2.36, 7.36) | 10.20 (0.19, 21.21) | 2.14 (-4.41, 9.13) |
| Yanhe | Guizhou | 5.13 (3.96, 6.32) | 2.25 (-3.98, 8.87) | 4.42 (-1.37, 10.55) | 4.31 (-1.70, 10.68) |
| Yinjiang | Guizhou | 5.62 (4.24, 7.02) | 1.75 (-3.68, 7.48) | 4.82 (1.75, 7.97) | 3.37 (-4.05, 11.37) |
| Yuping | Guizhou | 6.80 (5.03, 8.61) | 0.12 (-4.28, 4.72) | 13.84 (1.09, 28.18) | 1.86 (-8.15, 12.96) |
| Yuqing | Guizhou | 4.86 (3.37, 6.38) | 0.79 (-4.46, 6.32) | 8.03 (-1.05, 17.94) | 0.75 (-7.50, 9.73) |
| Zhenfeng | Guizhou | 5.83 (4.87, 6.81) | 3.19 (-2.54, 9.25) | 6.75 (4.45, 9.10) | 3.44 (-2.18, 9.38) |
| Zhengan | Guizhou | 5.81 (4.42, 7.22) | 2.34 (-4.78, 9.99) | 5.91 (-1.42, 13.77) | 6.23 (-2.51, 15.75) |
| Zhenning | Guizhou | 5.41 (4.28, 6.56) | 2.68 (-4.32, 10.18) | 9.23 (3.01, 15.83) | 2.99 (-1.67, 7.87) |
| Zhenyuan | Guizhou | 4.36 (3.25, 5.47) | 1.60 (-4.25, 7.81) | 7.98 (0.02, 16.57) | 0.01 (-6.61, 7.10) |
| Zhijin | Guizhou | 5.18 (3.97, 6.41) | 2.96 (-5.55, 12.23) | 8.68 (0.41, 17.64) | 1.97 (-3.51, 7.75) |
| Ziyun | Guizhou | 5.53 (4.14, 6.93) | 1.66 (-4.62, 8.36) | 7.10 (5.31, 8.91) | 2.62 (-6.38, 12.48) |
| Baisha | Hainan | 2.89 (2.12, 3.66) | 2.42 (-2.16, 7.21) | 4.94 (0.94, 9.10) | -0.18 (-4.60, 4.44) |
| Baoting | Hainan | 3.20 (2.50, 3.90) | 2.04 (-3.79, 8.22) | 4.20 (0.57, 7.95) | 0.04 (-5.14, 5.51) |
| Changjiang | Hainan | 3.14 (2.33, 3.96) | 0.57 (-3.22, 4.50) | 5.91 (1.09, 10.97) | -0.24 (-5.99, 5.86) |
| Chengmai | Hainan | 5.17 (3.97, 6.39) | 0.13 (-7.18, 8.03) | 9.71 (2.39, 17.55) | 1.12 (-5.56, 8.28) |
| Dingan | Hainan | 3.75 (3.03, 4.47) | 3.00 (-3.01, 9.40) | 4.75 (0.36, 9.32) | -0.06 (-3.72, 3.75) |
| Dongfang | Hainan | 3.23 (2.39, 4.09) | -0.34 (-4.47, 3.97) | 6.20 (1.51, 11.10) | 4.79 (0.74, 9.00) |
| Ledong | Hainan | 4.03 (3.28, 4.78) | 3.31 (-3.47, 10.56) | 4.21 (0.44, 8.13) | 2.87 (-2.96, 9.06) |
| Lingao | Hainan | 3.97 (3.00, 4.94) | 0.14 (-5.40, 6.00) | 8.17 (3.85, 12.68) | 1.33 (-3.90, 6.84) |
| Lingshui | Hainan | 5.83 (4.79, 6.88) | 7.31 (-5.13, 21.37) | 5.19 (-1.48, 12.31) | 4.10 (-0.63, 9.05) |
| Qionghai | Hainan | 2.61 (1.57, 3.67) | -2.67 (-10.03, 5.30) | 3.95 (-1.24, 9.41) | 2.42 (-3.46, 8.65) |
| Qiongzhong | Hainan | 2.82 (1.99, 3.66) | 1.35 (-3.98, 6.98) | 4.20 (0.47, 8.07) | -0.75 (-6.37, 5.21) |
| Tunchang | Hainan | 2.17 (1.62, 2.72) | -0.34 (-3.64, 3.07) | 3.06 (0.65, 5.53) | 0.42 (-3.69, 4.70) |
| Wanning | Hainan | 3.03 (2.20, 3.85) | -1.07 (-7.95, 6.32) | 5.36 (0.91, 10.00) | 3.26 (-0.09, 6.72) |
| Wenchang | Hainan | 2.46 (1.74, 3.19) | -0.36 (-6.50, 6.20) | 3.02 (-2.65, 9.02) | 2.79 (-2.45, 8.31) |
| Wuzhishan | Hainan | 2.90 (1.88, 3.93) | -0.04 (-3.53, 3.58) | 5.61 (2.33, 9.00) | 0.46 (-6.97, 8.48) |
| Anguo | Hebei | 2.19 (0.73, 3.67) | 0.68 (-8.62, 10.92) | -0.25 (-4.84, 4.56) | 9.12 (6.82, 11.46) |
| Anping | Hebei | 0.70 (0.05, 1.35) | 1.90 (-0.55, 4.41) | 1.36 (-6.74, 10.16) | 1.16 (-3.95, 6.54) |
| Anxin | Hebei | 2.16 (1.07, 3.26) | -1.09 (-10.27, 9.04) | -2.34 (-6.48, 1.97) | 6.65 (2.50, 10.97) |
| Baixiang | Hebei | 2.20 (1.47, 2.92) | -1.13 (-5.93, 3.92) | 2.59 (-0.23, 5.49) | 2.02 (-0.90, 5.03) |
| Bazhou | Hebei | -0.05 (-1.41, 1.32) | -3.92 (-8.35, 0.72) | -3.31 (-15.5, 10.65) | 1.68 (-3.82, 7.48) |
| Botou | Hebei | 1.52 (0.52, 2.53) | -1.26 (-5.30, 2.95) | 2.49 (-0.14, 5.19) | 3.13 (0.13, 6.21) |
| Boye | Hebei | 2.55 (1.91, 3.20) | 2.70 (-2.21, 7.87) | 0.67 (-2.26, 3.69) | 4.60 (0.55, 8.80) |
| Cang | Hebei | 3.16 (1.92, 4.41) | -0.57 (-7.02, 6.32) | 4.71 (-2.78, 12.78) | 3.26 (-2.95, 9.87) |
| Caofeidian | Hebei | 7.26 (5.03, 9.55) | 6.86 (-3.02, 17.75) | 19.67 (-3.90, 49.01) | 5.72 (-5.47, 18.23) |
| Changli | Hebei | 2.81 (1.93, 3.71) | 2.78 (-4.59, 10.73) | 4.58 (-1.57, 11.13) | 2.11 (-3.96, 8.55) |
| Chengan | Hebei | 2.85 (2.04, 3.67) | -0.19 (-6.27, 6.28) | -0.50 (-4.42, 3.59) | 4.43 (-0.86, 10.01) |
| Chengde | Hebei | 3.04 (2.27, 3.81) | 4.01 (0.68, 7.45) | 3.28 (-1.60, 8.40) | -0.09 (-8.47, 9.07) |
| Chicheng | Hebei | 1.73 (0.75, 2.72) | 5.04 (-0.80, 11.24) | 1.04 (-5.66, 8.23) | -1.86 (-10.46, 7.56) |
| Chongli | Hebei | 1.57 (0.72, 2.43) | 2.86 (-0.50, 6.32) | 0.88 (-5.45, 7.63) | -0.97 (-10.43, 9.47) |
| Ci | Hebei | -0.70 (-1.61, 0.22) | -4.72 (-14.48, 6.16) | 0.00 (-4.40, 4.60) | -0.24 (-4.26, 3.93) |
| Dachang | Hebei | 4.93 (3.28, 6.61) | 1.50 (-5.01, 8.45) | -2.18 (-18.18, 16.93) | 3.59 (-2.61, 10.17) |
| Dacheng | Hebei | 1.46 (0.40, 2.53) | -2.41 (-10.26, 6.12) | 0.91 (-4.82, 6.99) | 3.23 (-2.97, 9.83) |
| Daming | Hebei | 2.13 (1.52, 2.74) | 0.66 (-5.34, 7.05) | 1.41 (-0.17, 3.01) | 2.14 (-3.40, 8.00) |
| Dingxing | Hebei | 1.66 (0.84, 2.48) | 1.07 (-3.74, 6.12) | -0.77 (-6.99, 5.87) | 3.46 (-1.06, 8.18) |
| Dingzhou | Hebei | 3.02 (1.98, 4.08) | -0.98 (-7.23, 5.70) | 2.81 (-2.27, 8.14) | 2.63 (-0.67, 6.04) |
| Dongguang | Hebei | 1.38 (0.49, 2.28) | -3.20 (-9.78, 3.85) | 0.64 (-1.85, 3.19) | 2.27 (-3.48, 8.36) |
| Feixiang | Hebei | 3.71 (2.73, 4.70) | 0.47 (-8.00, 9.71) | 2.21 (-5.95, 11.07) | 3.54 (-0.19, 7.42) |
| Fengning | Hebei | 2.45 (1.31, 3.60) | -0.14 (-2.84, 2.63) | 3.12 (-2.05, 8.57) | 1.27 (-8.00, 11.47) |
| Fucheng | Hebei | 1.82 (0.76, 2.89) | -2.06 (-9.01, 5.41) | 0.08 (-2.53, 2.76) | 2.66 (-4.47, 10.31) |
| Funing | Hebei | 3.31 (2.58, 4.04) | 3.50 (-0.36, 7.51) | 1.79 (-5.04, 9.10) | 3.93 (-2.13, 10.37) |
| Fuping | Hebei | 2.47 (1.44, 3.51) | 3.05 (-1.46, 7.76) | -0.99 (-3.80, 1.90) | 3.79 (-2.25, 10.20) |
| Gaobeidian | Hebei | 0.72 (-0.68, 2.14) | -5.45 (-12.78, 2.50) | -5.03 (-10.14, 0.37) | 5.70 (0.49, 11.18) |
| Gaocheng | Hebei | 3.58 (2.77, 4.39) | 0.87 (-5.95, 8.18) | 5.91 (1.40, 10.62) | 3.05 (-1.98, 8.34) |
| Gaoyang | Hebei | 2.30 (1.35, 3.25) | 0.07 (-6.44, 7.03) | 3.63 (0.51, 6.85) | 6.76 (3.43, 10.19) |
| Gaoyi | Hebei | 2.44 (1.79, 3.10) | 1.79 (-6.21, 10.48) | 3.59 (0.25, 7.05) | 1.92 (-2.36, 6.38) |
| Guan | Hebei | 3.24 (2.26, 4.22) | 5.18 (1.84, 8.64) | 2.31 (-5.08, 10.28) | 5.19 (1.19, 9.35) |
| Guangping | Hebei | 2.24 (1.46, 3.02) | -1.94 (-8.51, 5.09) | 0.59 (-1.57, 2.80) | 3.36 (-1.62, 8.60) |
| Guangzong | Hebei | 2.37 (1.59, 3.15) | 1.33 (-3.72, 6.64) | 0.97 (-3.21, 5.34) | 4.28 (-1.53, 10.44) |
| Guantao | Hebei | 1.36 (0.55, 2.17) | 2.45 (-8.08, 14.18) | -0.75 (-4.21, 2.84) | 0.84 (-3.43, 5.30) |
| Gucheng | Hebei | 1.25 (0.51, 2.00) | 0.23 (-5.26, 6.04) | -0.90 (-2.83, 1.08) | 1.82 (-4.07, 8.06) |
| Guyuan | Hebei | 3.66 (2.40, 4.93) | 2.45 (-2.81, 7.99) | 1.64 (-6.30, 10.25) | 1.23 (-12.29, 16.84) |
| Haixing | Hebei | 3.80 (2.41, 5.21) | 2.21 (-4.79, 9.73) | 3.35 (-4.38, 11.70) | 1.14 (-9.99, 13.65) |
| Hejian | Hebei | 1.89 (1.03, 2.75) | -1.77 (-8.62, 5.60) | 2.11 (-3.12, 7.62) | 3.21 (-2.34, 9.07) |
| Huaian | Hebei | 3.42 (2.19, 4.68) | 8.69 (-1.90, 20.42) | -1.06 (-7.90, 6.29) | 3.09 (-4.39, 11.16) |
| Huailai | Hebei | 1.46 (0.41, 2.52) | -2.55 (-11.47, 7.27) | -1.04 (-9.22, 7.88) | 1.97 (-1.86, 5.95) |
| Huanghua | Hebei | 3.61 (2.59, 4.64) | 0.98 (-2.51, 4.59) | 5.32 (-0.17, 11.13) | 2.58 (-5.58, 11.43) |
| Jing | Hebei | 1.24 (0.12, 2.36) | -3.95 (-13.12, 6.19) | 1.12 (-1.30, 3.61) | 2.01 (-3.87, 8.25) |
| Jingxing | Hebei | 1.46 (0.54, 2.39) | 2.64 (-1.61, 7.07) | 3.13 (-2.52, 9.11) | -2.74 (-11.60, 7.02) |
| Jinzhou | Hebei | 1.63 (0.72, 2.55) | -0.12 (-7.98, 8.41) | 4.40 (-0.79, 9.86) | -1.11 (-6.06, 4.09) |
| Jize | Hebei | 2.46 (1.82, 3.10) | 0.23 (-4.82, 5.55) | 1.86 (-3.47, 7.49) | 2.79 (-2.04, 7.87) |
| Jizhou | Hebei | 0.46 (-0.41, 1.34) | -0.98 (-8.41, 7.05) | 0.17 (-4.39, 4.94) | 0.99 (-3.64, 5.83) |
| Julu | Hebei | 2.53 (1.67, 3.40) | 0.45 (-4.82, 6.01) | 0.77 (-2.51, 4.16) | 5.06 (0.41, 9.91) |
| Kangbao | Hebei | 3.13 (1.58, 4.70) | -1.19 (-8.97, 7.26) | 0.29 (-7.43, 8.65) | 3.66 (-11.46, 21.38) |
| Kuanchen | Hebei | 1.87 (0.30, 3.46) | 8.42 (3.42, 13.66) | 2.53 (-2.76, 8.10) | -4.89 (-17.63, 9.83) |
| Laishui | Hebei | 2.60 (1.82, 3.40) | 0.26 (-4.63, 5.40) | 1.83 (-3.35, 7.29) | 3.14 (-2.77, 9.41) |
| Laiyuan | Hebei | 1.78 (1.28, 2.29) | -0.26 (-4.07, 3.69) | 1.33 (-2.99, 5.85) | 2.19 (-0.99, 5.48) |
| Leting | Hebei | 1.14 (0.06, 2.22) | -1.70 (-10.64, 8.14) | 2.49 (-4.76, 10.29) | -2.73 (-7.17, 1.93) |
| Li | Hebei | 2.59 (1.30, 3.90) | -3.64 (-12.57, 6.20) | 2.82 (0.20, 5.50) | 5.24 (0.94, 9.74) |
| Lincheng | Hebei | 2.44 (1.45, 3.44) | -0.71 (-4.05, 2.76) | 4.77 (-4.34, 14.75) | 0.71 (-8.23, 10.52) |
| Lingshou | Hebei | 1.63 (0.79, 2.47) | 2.95 (-0.87, 6.91) | -0.89 (-5.46, 3.91) | 5.00 (1.05, 9.11) |
| Linxi | Hebei | 1.35 (0.48, 2.22) | 0.34 (-7.21, 8.50) | 0.02 (-2.54, 2.65) | 2.58 (-3.66, 9.22) |
| Linzhang | Hebei | 2.10 (1.44, 2.75) | 1.47 (-4.56, 7.87) | -0.35 (-3.25, 2.62) | 4.01 (-0.83, 9.09) |
| Longhua | Hebei | 2.73 (1.86, 3.61) | 3.47 (0.54, 6.50) | 2.14 (-2.88, 7.42) | 1.69 (-7.90, 12.27) |
| Longyao | Hebei | 0.65 (-0.16, 1.47) | -0.07 (-6.88, 7.24) | -1.00 (-4.98, 3.15) | 2.55 (-1.82, 7.11) |
| Luancheng | Hebei | 2.78 (1.60, 3.98) | -0.47 (-10.09, 10.18) | 0.33 (-11.59, 13.85) | 4.92 (1.77, 8.17) |
| Luannan | Hebei | 1.36 (0.45, 2.28) | 2.03 (-2.88, 7.19) | 4.26 (-3.89, 13.09) | -0.32 (-5.60, 5.27) |
| Luanping | Hebei | 2.76 (1.56, 3.96) | 8.98 (5.42, 12.65) | 3.53 (-4.19, 11.87) | -1.95 (-11.06, 8.08) |
| Luanzhou | Hebei | 2.21 (0.92, 3.52) | -0.10 (-12.47, 14.02) | 3.55 (-7.81, 16.31) | -1.28 (-8.05, 5.98) |
| Lulong | Hebei | 2.10 (1.48, 2.72) | 2.93 (-0.96, 6.97) | 0.99 (-3.44, 5.62) | 3.47 (-3.50, 10.94) |
| Luquan | Hebei | 0.25 (-1.09, 1.60) | -1.30 (-11.66, 10.27) | -5.67 (-16.72, 6.85) | 1.20 (-2.18, 4.70) |
| Mancheng | Hebei | 2.08 (1.17, 2.98) | 1.53 (-3.59, 6.92) | 0.42 (-8.42, 10.11) | 4.95 (1.48, 8.55) |
| Mengcun | Hebei | 0.87 (0.03, 1.71) | -1.91 (-9.98, 6.88) | 0.9 (-4.60, 6.71) | -0.33 (-6.42, 6.15) |
| Nangong | Hebei | 1.96 (1.17, 2.76) | 4.31 (-1.63, 10.6) | -1.45 (-3.60, 0.74) | 4.79 (0.40, 9.38) |
| Nanhe | Hebei | 4.24 (3.27, 5.23) | 1.36 (-4.27, 7.32) | 1.72 (-2.84, 6.50) | 9.20 (4.01, 14.65) |
| Nanpi | Hebei | 2.70 (1.69, 3.71) | 4.69 (-6.63, 17.39) | 2.47 (0.16, 4.84) | 2.59 (-2.63, 8.08) |
| Neiqiu | Hebei | -0.22 (-0.98, 0.56) | -2.30 (-8.39, 4.19) | -0.93 (-8.37, 7.11) | 0.58 (-5.08, 6.57) |
| Ningjin | Hebei | 2.31 (1.45, 3.18) | -0.02 (-4.89, 5.10) | 0.31 (-4.07, 4.89) | 3.54 (-2.02, 9.42) |
| Pingquan | Hebei | 3.27 (2.42, 4.14) | 2.80 (0.31, 5.36) | 4.51 (1.28, 7.84) | -1.41 (-9.67, 7.60) |
| Pingshan | Hebei | 0.42 (-0.26, 1.10) | 0.68 (-2.40, 3.87) | -0.15 (-3.07, 2.87) | 0.01 (-9.51, 10.52) |
| Pingxiang | Hebei | 3.14 (1.78, 4.52) | -0.94 (-9.03, 7.87) | 1.86 (-1.64, 5.49) | 8.1 (3.38, 13.04) |
| Qianan | Hebei | -2.4 (-3.68, -1.12) | -1.74 (-11.03, 8.51) | 0.72 (-13.65, 17.49) | -3.15 (-8.38, 2.37) |
| Qianxi | Hebei | -0.61 (-1.68, 0.48) | 2.66 (-3.42, 9.12) | 0.85 (-8.59, 11.27) | -4.67 (-11.4, 2.57) |
| Qing | Hebei | 0.46 (-0.57, 1.51) | -1.55 (-10.02, 7.71) | -0.27 (-5.44, 5.17) | 0.59 (-6.85, 8.62) |
| Qinghe | Hebei | -0.01 (-1.44, 1.44) | -1.88 (-9.19, 6.03) | -4.57 (-8.28, -0.71) | 3.93 (-4.81, 13.47) |
| Qinglong | Hebei | 2.00 (1.43, 2.58) | 3.37 (-0.93, 7.86) | 1.14 (-0.55, 2.86) | 0.72 (-4.75, 6.50) |
| Qingyuan | Hebei | 2.45 (1.07, 3.85) | -1.93 (-14.07, 11.93) | -2.02 (-8.78, 5.25) | 5.63 (0.86, 10.63) |
| Qiu | Hebei | 1.71 (0.92, 2.51) | 0.61 (-4.78, 6.30) | 2.39 (-3.09, 8.19) | 1.79 (-4.62, 8.63) |
| Quyang | Hebei | 2.35 (1.42, 3.29) | 0.56 (-3.58, 4.89) | 0.73 (-2.25, 3.79) | 5.16 (-0.30, 10.93) |
| Quzhou | Hebei | 2.12 (1.45, 2.79) | -0.29 (-5.79, 5.53) | 1.36 (-1.87, 4.69) | 1.51 (-4.95, 8.42) |
| Raoyang | Hebei | 2.19 (1.01, 3.39) | -2.95 (-9.39, 3.95) | 2.54 (-0.73, 5.93) | 4.83 (-1.17, 11.19) |
| Ren | Hebei | 2.84 (1.99, 3.70) | 0.97 (-4.82, 7.10) | 0.24 (-2.56, 3.12) | 7.62 (3.01, 12.44) |
| Renqiu | Hebei | -0.91 (-2.81, 1.03) | -8.03 (-12.38, -3.45) | 0.42 (-6.76, 8.15) | 4.80 (0.93, 8.83) |
| Rongcheng | Hebei | 2.65 (1.59, 3.73) | -2.78 (-10.49, 5.60) | 2.23 (-0.99, 5.56) | 6.43 (3.94, 8.99) |
| Sanhe | Hebei | -2.42 (-3.82, -1.00) | -0.44 (-4.15, 3.42) | -5.11 (-21.39, 14.54) | 0.38 (-3.57, 4.48) |
| Shahe | Hebei | 0.87 (-0.25, 2.00) | -3.92 (-9.28, 1.76) | 0.31 (-9.33, 10.98) | -0.68 (-6.86, 5.92) |
| Shangyi | Hebei | 5.11 (3.84, 6.39) | 1.66 (-2.73, 6.23) | 4.05 (-2.95, 11.56) | 3.27 (-11.95, 21.12) |
| She | Hebei | -1.62 (-2.54, -0.70) | 3.37 (-1.46, 8.44) | -2.93 (-9.38, 3.98) | -3.81 (-10.19, 3.01) |
| Shenze | Hebei | 1.71 (1.04, 2.39) | 0.55 (-5.31, 6.79) | 1.36 (-2.68, 5.56) | -1.18 (-6.24, 4.16) |
| Shenzhou | Hebei | 2.06 (1.14, 2.99) | -1.69 (-10.43, 7.89) | 3.20 (0.33, 6.16) | 1.59 (-3.95, 7.45) |
| Shunping | Hebei | 1.75 (0.81, 2.69) | 1.14 (-3.48, 5.97) | -2.52 (-11.05, 6.84) | 2.53 (-2.07, 7.35) |
| Suning | Hebei | 1.94 (0.92, 2.97) | 1.12 (-5.12, 7.77) | 4.23 (-2.91, 11.90) | 2.62 (-2.14, 7.61) |
| Tang | Hebei | 2.99 (2.21, 3.78) | 2.73 (-3.98, 9.91) | -0.65 (-4.29, 3.13) | 3.52 (-1.40, 8.69) |
| Tangxian | Hebei | 2.94 (2.15, 3.75) | 2.71 (-3.93, 9.80) | -0.86 (-4.52, 2.94) | 3.42 (-1.66, 8.78) |
| Wangdou | Hebei | 2.46 (1.62, 3.31) | 2.23 (-6.37, 11.63) | 2.88 (1.01, 4.79) | 2.70 (-1.88, 7.51) |
| Wanquan | Hebei | 3.64 (1.89, 5.41) | 9.92 (0.51, 20.22) | -1.16 (-18.40, 19.73) | 2.05 (-3.42, 7.82) |
| Wei | Hebei | 1.95 (1.03, 2.87) | 0.72 (-7.29, 9.41) | -1.82 (-4.71, 1.16) | 5.01 (-1.30, 11.73) |
| Wei | Hebei | 2.55 (1.82, 3.28) | 1.07 (-6.26, 8.96) | 2.00 (-0.30, 4.37) | 3.33 (-1.93, 8.88) |
| Wei | Hebei | 0.18 (-0.73, 1.10) | -2.38 (-7.04, 2.51) | -0.15 (-6.45, 6.59) | 2.25 (-2.89, 7.66) |
| Weichang | Hebei | 3.82 (2.63, 5.03) | 0.96 (-1.21, 3.17) | 3.71 (-0.28, 7.86) | 3.26 (-8.80, 16.91) |
| Wenan | Hebei | 0.77 (-0.47, 2.03) | -2.74 (-11.06, 6.35) | 1.01 (-6.52, 9.15) | 4.27 (-1.95, 10.87) |
| Wuan | Hebei | -2.15 (-3.14, -1.15) | -5.45 (-10.29, -0.36) | 2.60 (-4.87, 10.66) | -2.73 (-7.39, 2.17) |
| Wuji | Hebei | 1.37 (0.61, 2.13) | -0.86 (-7.21, 5.93) | 2.39 (-0.82, 5.72) | 1.36 (-3.59, 6.57) |
| Wuqiang | Hebei | 1.72 (0.88, 2.57) | -0.63 (-4.00, 2.85) | 2.09 (-1.35, 5.64) | 1.99 (-5.34, 9.89) |
| Wuqiao | Hebei | 1.99 (1.15, 2.83) | -2.54 (-6.91, 2.04) | 2.36 (-1.06, 5.90) | 2.04 (-1.88, 6.11) |
| Wuyi | Hebei | 2.53 (1.79, 3.27) | 2.11 (-6.05, 10.97) | 2.49 (-1.02, 6.12) | 2.77 (-1.78, 7.53) |
| Xian | Hebei | 1.84 (1.07, 2.62) | 1.67 (-2.98, 6.53) | 0.87 (-3.87, 5.85) | -0.5 (-8.10, 7.72) |
| Xianghe | Hebei | 2.87 (1.51, 4.24) | 3.58 (-1.76, 9.21) | -5.17 (-10.75, 0.74) | 2.43 (-4.30, 9.64) |
| Xinglong | Hebei | 1.01 (0.22, 1.80) | 1.77 (-3.24, 7.05) | 2.33 (-1.69, 6.52) | -0.22 (-5.00, 4.79) |
| Xingtai | Hebei | 1.37 (0.45, 2.30) | -1.88 (-7.75, 4.37) | -0.24 (-8.01, 8.18) | 3.59 (-3.42, 11.10) |
| Xinhe | Hebei | 1.80 (1.08, 2.52) | -0.49 (-6.76, 6.19) | -0.56 (-3.94, 2.93) | 2.75 (-1.40, 7.07) |
| Xinji | Hebei | 1.18 (0.10, 2.28) | -4.45 (-9.31, 0.68) | 3.08 (-1.18, 7.53) | 2.73 (-1.82, 7.49) |
| Xinle | Hebei | 1.53 (0.72, 2.35) | 0.31 (-5.75, 6.75) | 0.86 (-4.26, 6.26) | 2.96 (-0.92, 7.00) |
| Xiong | Hebei | 2.09 (1.04, 3.15) | 1.39 (-6.70, 10.18) | 0.30 (-3.58, 4.34) | 2.99 (-1.74, 7.95) |
| Xushui | Hebei | 4.72 (3.22, 6.25) | 2.94 (-5.53, 12.16) | 1.08 (-4.70, 7.20) | 11.37 (1.60, 22.08) |
| Yangyuan | Hebei | 1.35 (0.66, 2.04) | 0.21 (-0.92, 1.35) | -0.15 (-7.22, 7.47) | 1.12 (-5.29, 7.97) |
| Yanshan | Hebei | 0.79 (-0.03, 1.62) | 2.43 (-4.57, 9.95) | -2.55 (-8.07, 3.31) | 0.69 (-5.38, 7.15) |
| Yi | Hebei | 2.74 (1.88, 3.60) | 2.05 (-2.82, 7.17) | 1.74 (-1.06, 4.62) | 2.63 (-6.18, 12.27) |
| Yongnian | Hebei | 0.99 (0.18, 1.81) | 0.69 (-4.79, 6.50) | -2.20 (-9.10, 5.23) | 2.96 (-2.17, 8.36) |
| Yongqing | Hebei | 3.73 (2.14, 5.33) | -1.16 (-7.69, 5.83) | 5.16 (1.57, 8.87) | 9.55 (0.67, 19.21) |
| Yuanshi | Hebei | 2.54 (1.98, 3.11) | 2.06 (-1.66, 5.91) | 3.94 (0.19, 7.83) | 1.39 (-3.17, 6.17) |
| Yutian | Hebei | 2.58 (1.73, 3.44) | 1.55 (-6.10, 9.83) | 0.76 (-6.05, 8.07) | 0.31 (-4.53, 5.4) |
| Zanhuang | Hebei | 1.64 (0.82, 2.47) | 0.33 (-4.63, 5.54) | 4.03 (0.65, 7.52) | -2.38 (-9.37, 5.16) |
| Zaoqiang | Hebei | 1.50 (0.52, 2.48) | -1.15 (-9.75, 8.27) | 1.81 (0.16, 3.49) | 2.68 (-2.67, 8.32) |
| Zhangbei | Hebei | 3.63 (2.50, 4.76) | 4.82 (-0.78, 10.74) | -2.20 (-6.10, 1.86) | 2.39 (-8.23, 14.23) |
| Zhao | Hebei | 1.92 (1.31, 2.54) | 0.56 (-5.00, 6.45) | 1.60 (-2.45, 5.82) | 0.75 (-4.76, 6.57) |
| Zhengding | Hebei | 3.81 (1.57, 6.10) | -3.26 (-15.38, 10.59) | -0.14 (-12.64, 14.15) | 14.89 (10.29, 19.68) |
| Zhuolu | Hebei | 2.89 (2.10, 3.69) | 1.63 (-4.09, 7.68) | 3.89 (-2.99, 11.25) | -0.84 (-4.85, 3.34) |
| Zhuozhou | Hebei | -0.21 (-1.82, 1.41) | -6.17 (-16.76, 5.78) | -4.60 (-10.69, 1.91) | 4.53 (-2.67, 12.27) |
| Zunhua | Hebei | -1.68 (-2.62, -0.72) | -0.46 (-5.58, 4.94) | -3.64 (-15.00, 9.23) | -0.23 (-4.63, 4.38) |
| Acheng | Heilongjiang | 1.25 (0.20, 2.31) | 0.80 (-8.92, 11.57) | -0.74 (-7.90, 6.97) | 0.14 (-7.70, 8.64) |
| Anda | Heilongjiang | 2.36 (0.36, 4.39) | 2.66 (-9.30, 16.19) | 8.28 (-2.87, 20.71) | -7.44 (-15.03, 0.84) |
| Baiquan | Heilongjiang | 1.29 (0.51, 2.09) | -0.47 (-7.64, 7.25) | 2.35 (-1.58, 6.44) | -0.19 (-6.00, 5.97) |
| Baoqing | Heilongjiang | 1.55 (0.08, 3.05) | 3.45 (-1.12, 8.24) | 6.57 (-6.10, 20.95) | -5.57 (-12.39, 1.78) |
| Bayan | Heilongjiang | 2.28 (1.31, 3.26) | 1.27 (-5.43, 8.44) | 3.48 (-2.59, 9.92) | -1.79 (-7.56, 4.35) |
| Beian | Heilongjiang | 1.72 (0.49, 2.98) | -2.03 (-5.77, 1.86) | 4.55 (-1.97, 11.50) | -3.39 (-9.92, 3.61) |
| Bin | Heilongjiang | 2.53 (1.24, 3.83) | 1.22 (-5.62, 8.57) | 4.87 (-1.37, 11.52) | -4.70 (-13.57, 5.09) |
| Boli | Heilongjiang | -0.45 (-1.38, 0.48) | -0.74 (-8.30, 7.44) | -2.58 (-7.87, 3.01) | 3.16 (0.35, 6.05) |
| Dongning | Heilongjiang | 2.27 (0.48, 4.09) | 3.79 (-0.20, 7.93) | 5.21 (-1.47, 12.35) | -9.85 (-19.14, 0.51) |
| Dorbod | Heilongjiang | 4.20 (2.27, 6.17) | 1.40 (-7.34, 10.97) | 13.98 (3.90, 25.04) | -3.58 (-13.17, 7.08) |
| Fangzheng | Heilongjiang | 1.64 (0.73, 2.56) | 0.62 (-4.80, 6.36) | 3.48 (-3.09, 10.48) | -1.95 (-9.00, 5.64) |
| Fujin | Heilongjiang | 2.35 (0.83, 3.90) | 2.35 (-5.75, 11.14) | 8.18 (-0.43, 17.53) | -6.45 (-12.32, -0.18) |
| Fuyu | Heilongjiang | 1.42 (0.45, 2.39) | -1.74 (-10.46, 7.83) | 1.35 (-3.79, 6.78) | -2.54 (-7.08, 2.22) |
| Fuyuan | Heilongjiang | 3.84 (2.05, 5.67) | 0.42 (-6.32, 7.64) | -1.59 (-3.41, 0.26) | 2.36 (-10.12, 16.57) |
| Gannan | Heilongjiang | 2.11 (1.17, 3.06) | 0.47 (-8.40, 10.19) | 3.34 (-2.22, 9.23) | 0.81 (-4.67, 6.60) |
| Hailin | Heilongjiang | 2.83 (1.46, 4.21) | 3.56 (-3.53, 11.17) | 5.62 (-0.22, 11.82) | -6.29 (-12.69, 0.58) |
| Hailun | Heilongjiang | 1.04 (0.01, 2.08) | -1.36 (-12.12, 10.72) | 0.01 (-3.63, 3.79) | 0.26 (-6.15, 7.12) |
| Huachuan | Heilongjiang | 4.62 (2.25, 7.04) | 5.85 (-5.99, 19.18) | 13.6 (-8.17, 40.53) | -4.42 (-17.21, 10.34) |
| Huanan | Heilongjiang | 1.85 (0.74, 2.96) | -0.55 (-6.34, 5.59) | 4.14 (-1.25, 9.83) | -2.32 (-10.66, 6.80) |
| Hulin | Heilongjiang | 1.59 (-0.66, 3.88) | 0.40 (-4.78, 5.86) | 14.73 (1.60, 29.55) | -8.00 (-20.57, 6.56) |
| Huma | Heilongjiang | -2.39 (-4.74, 0.02) | 0.24 (-3.06, 3.65) | 1.63 (-5.45, 9.24) | -16.63 (-31.00, 0.72) |
| Jiayin | Heilongjiang | 1.04 (-0.01, 2.11) | -0.85 (-4.38, 2.81) | 2.88 (-1.88, 7.88) | -5.41 (-12.48, 2.24) |
| Jidong | Heilongjiang | 0.77 (0.08, 1.46) | -0.07 (-4.16, 4.21) | 0.99 (-3.68, 5.89) | -1.13 (-7.36, 5.53) |
| Jixian | Heilongjiang | -0.54 (-1.67, 0.60) | 1.25 (-8.39, 11.91) | -1.52 (-10.14, 7.93) | -2.47 (-7.96, 3.35) |
| Kedong | Heilongjiang | 0.78 (-0.29, 1.86) | 2.64 (-4.70, 10.53) | -2.67 (-11.02, 6.47) | -1.33 (-6.45, 4.08) |
| Keshan | Heilongjiang | 0.93 (-0.05, 1.91) | -0.55 (-9.86, 9.73) | -1.27 (-7.63, 5.52) | -1.61 (-6.28, 3.30) |
| Lanxi | Heilongjiang | 2.85 (1.96, 3.75) | 2.83 (-5.81, 12.27) | 2.58 (-2.24, 7.63) | 1.92 (-3.01, 7.11) |
| Lindian | Heilongjiang | 2.41 (1.50, 3.33) | 0.08 (-9.19, 10.30) | 4.67 (-1.67, 11.42) | 1.01 (-3.80, 6.06) |
| Linkou | Heilongjiang | 2.55 (1.71, 3.39) | 1.56 (-2.64, 5.93) | 3.64 (-0.79, 8.27) | -2.63 (-7.58, 2.58) |
| Longjiang | Heilongjiang | 3.15 (2.42, 3.88) | 0.67 (-8.14, 10.32) | 4.20 (0.96, 7.54) | 2.50 (-0.88, 5.99) |
| Luobei | Heilongjiang | 3.28 (-0.29, 6.98) | 1.82 (-2.62, 6.47) | 18.95 (-1.46, 43.59) | -10.40 (-38.34, 30.2) |
| Mingshui | Heilongjiang | 3.27 (2.55, 4.00) | 2.89 (-5.10, 11.55) | 2.52 (-3.10, 8.47) | 2.85 (-0.52, 6.34) |
| Mishan | Heilongjiang | 2.51 (1.40, 3.63) | 0.90 (-4.05, 6.11) | 2.42 (-5.89, 11.47) | -1.87 (-9.16, 6.01) |
| Mohe | Heilongjiang | 0.91 (-0.07, 1.90) | -0.84 (-3.29, 1.68) | 5.61 (-2.03, 13.84) | -1.76 (-8.86, 5.90) |
| Mulan | Heilongjiang | 1.99 (1.13, 2.86) | 0.95 (-4.09, 6.26) | 3.70 (-1.11, 8.74) | -1.18 (-7.19, 5.22) |
| Muleng | Heilongjiang | 2.61 (1.14, 4.10) | 4.16 (-4.30, 13.37) | 4.91 (-3.30, 13.82) | -6.07 (-14.52, 3.22) |
| Nehe | Heilongjiang | 1.08 (-0.17, 2.35) | -4.74 (-14.28, 5.87) | 1.54 (-4.03, 7.43) | 1.06 (-4.72, 7.20) |
| Nenjiang | Heilongjiang | 2.34 (0.97, 3.74) | 2.13 (-3.73, 8.35) | 5.42 (-8.60, 21.60) | -2.32 (-12.92, 9.57) |
| Ningan | Heilongjiang | 2.48 (1.16, 3.82) | 0.71 (-3.84, 5.47) | 5.33 (-2.30, 13.57) | -5.74 (-13.97, 3.27) |
| Qingan | Heilongjiang | 2.26 (1.37, 3.15) | -0.12 (-6.40, 6.59) | 3.68 (-1.74, 9.39) | 0.60 (-2.93, 4.25) |
| Qinggang | Heilongjiang | 2.13 (1.30, 2.96) | -0.49 (-6.86, 6.32) | 3.03 (0.38, 5.74) | 3.09 (-0.73, 7.06) |
| Raohe | Heilongjiang | 0.87 (-1.76, 3.58) | -6.62 (-15.09, 2.70) | 15.01 (-0.58, 33.03) | -9.61 (-24.67, 8.47) |
| Shangzhi | Heilongjiang | 0.81 (-0.04, 1.67) | -0.97 (-5.26, 3.51) | 3.78 (-2.46, 10.42) | -3.07 (-8.29, 2.44) |
| Shuangcheng | Heilongjiang | 1.97 (0.55, 3.41) | 2.03 (-5.99, 10.73) | 5.11 (-2.29, 13.07) | -5.51 (-16.40, 6.80) |
| Suibin | Heilongjiang | 1.77 (0.33, 3.23) | -0.69 (-5.87, 4.76) | 5.46 (-2.53, 14.11) | -4.52 (-17.70, 10.78) |
| Suifenhe | Heilongjiang | 0.54 (-1.87, 3.02) | -1.20 (-12.01, 10.92) | 9.42 (-9.80, 32.75) | -11.33 (-23.17, 2.35) |
| Suileng | Heilongjiang | 2.08 (1.08, 3.08) | 0.74 (-6.28, 8.27) | 2.32 (-2.94, 7.87) | -3.26 (-6.83, 0.45) |
| Sunwu | Heilongjiang | 2.79 (1.80, 3.79) | 0.54 (-6.30, 7.88) | 1.97 (-3.52, 7.77) | 4.68 (-2.77, 12.70) |
| Tahe | Heilongjiang | 1.87 (-0.46, 4.27) | -0.54 (-4.36, 3.43) | 2.07 (-14.82, 22.31) | -5.57 (-26.61, 21.51) |
| Tailai | Heilongjiang | 2.74 (1.77, 3.73) | -0.28 (-9.37, 9.72) | 1.92 (-3.08, 7.17) | 4.14 (-0.66, 9.16) |
| Tangyuan | Heilongjiang | 1.86 (0.90, 2.82) | 5.29 (-3.57, 14.96) | 1.65 (-0.89, 4.26) | -1.95 (-9.01, 5.66) |
| Tieli | Heilongjiang | 0.46 (-0.28, 1.21) | 0.34 (-6.20, 7.33) | 0.35 (-2.52, 3.31) | -0.48 (-3.76, 2.90) |
| Tonghe | Heilongjiang | 1.57 (0.52, 2.64) | -0.34 (-5.09, 4.64) | 1.97 (-3.90, 8.20) | -0.10 (-9.70, 10.52) |
| Tongjiang | Heilongjiang | 3.25 (1.33, 5.22) | 3.27 (-3.43, 10.43) | 9.27 (-10.74, 33.76) | -1.45 (-11.06, 9.21) |
| Wangkui | Heilongjiang | 2.33 (1.56, 3.10) | 2.20 (-7.50, 12.92) | 2.10 (-0.09, 4.33) | 1.27 (-3.90, 6.71) |
| Wuchang | Heilongjiang | 2.52 (1.35, 3.70) | 1.00 (-5.75, 8.24) | 3.74 (-3.14, 11.10) | -2.90 (-10.69, 5.57) |
| Wudalianchi | Heilongjiang | 3.66 (2.42, 4.91) | 0.74 (-7.52, 9.74) | 4.37 (-1.08, 10.12) | 3.06 (-10.74, 19.00) |
| Xunke | Heilongjiang | 2.20 (1.16, 3.24) | -1.13 (-6.43, 4.47) | 5.46 (-4.09, 15.95) | 0.42 (-6.03, 7.31) |
| Yanshou | Heilongjiang | 2.36 (1.20, 3.53) | 0.21 (-7.44, 8.49) | 3.82 (-2.13, 10.13) | -1.70 (-6.22, 3.02) |
| Yian | Heilongjiang | 1.19 (0.49, 1.89) | 1.73 (-4.98, 8.92) | 0.68 (-4.69, 6.36) | -0.46 (-4.85, 4.14) |
| Yilan | Heilongjiang | 1.78 (0.56, 3.02) | 2.00 (-5.62, 10.24) | 3.78 (-1.43, 9.27) | -6.32 (-12.31, 0.09) |
| Youyi | Heilongjiang | -5.12 (-8.54, -1.57) | -3.98 (-8.19, 0.43) | 15.09 (2.02, 29.83) | -0.85 (-9.94, 9.15) |
| Zhaodong | Heilongjiang | 0.17 (-0.93, 1.29) | 3.03 (-2.29, 8.65) | 0.93 (-7.66, 10.32) | -4.92 (-13.12, 4.04) |
| Zhaoyuan | Heilongjiang | 2.54 (1.13, 3.96) | 4.75 (-4.01, 14.31) | 6.48 (0.64, 12.67) | -4.61 (-8.12, -0.96) |
| Zhaozhou | Heilongjiang | 2.98 (1.46, 4.51) | 2.75 (-8.42, 15.29) | 6.36 (-0.08, 13.23) | -5.64 (-9.36, -1.77) |
| Anyang | Henan | 0.75 (-0.15, 1.66) | -2.69 (-12.34, 8.01) | 0.09 (-5.53, 6.04) | 0.35 (-2.28, 3.06) |
| Baofeng | Henan | 2.95 (2.08, 3.83) | -1.27 (-7.10, 4.92) | 5.62 (-0.07, 11.64) | 2.54 (-3.81, 9.30) |
| Biyang | Henan | 4.01 (3.29, 4.74) | 2.70 (-3.33, 9.11) | 4.00 (-0.22, 8.39) | 4.06 (-2.34, 10.88) |
| Boai | Henan | 1.59 (0.23, 2.96) | 0.39 (-13.82, 16.96) | 2.12 (-8.13, 13.51) | 1.60 (-2.51, 5.89) |
| Changge | Henan | 1.95 (1.19, 2.71) | -2.47 (-6.32, 1.55) | 2.46 (-2.31, 7.46) | 3.74 (-1.07, 8.79) |
| Changyuan | Henan | 1.00 (-0.35, 2.36) | -6.14 (-11.65, -0.28) | 1.79 (-3.04, 6.86) | 2.69 (-1.52, 7.08) |
| Dancheng | Henan | 2.48 (1.76, 3.21) | 0.72 (-3.01, 4.60) | 1.59 (-2.65, 6.01) | 4.60 (0.91, 8.44) |
| Dengfeng | Henan | 2.54 (1.69, 3.39) | -1.07 (-4.05, 2.00) | 1.75 (-2.70, 6.41) | 0.94 (-7.73, 10.43) |
| Dengzhou | Henan | 2.97 (2.02, 3.93) | 0.99 (-1.51, 3.57) | 2.67 (-0.96, 6.44) | 1.02 (-1.90, 4.01) |
| Fan | Henan | 3.72 (2.85, 4.61) | 1.06 (-5.50, 8.08) | 5.55 (0.50, 10.84) | 4.30 (-0.06, 8.86) |
| Fangcheng | Henan | 2.99 (2.15, 3.84) | 0.57 (-5.37, 6.89) | 2.95 (-2.14, 8.29) | 3.06 (-2.62, 9.08) |
| Fengqiu | Henan | 2.93 (2.20, 3.67) | 1.46 (-4.03, 7.26) | 2.14 (-0.15, 4.49) | 5.07 (1.25, 9.03) |
| Fugou | Henan | 1.99 (1.11, 2.87) | -0.33 (-4.95, 4.52) | 2.28 (-1.55, 6.26) | 2.23 (-5.33, 10.39) |
| Gongyi | Henan | 0.54 (-0.73, 1.83) | -3.98 (-9.76, 2.18) | 2.07 (-5.54, 10.29) | 2.13 (-3.68, 8.28) |
| Guangshan | Henan | 2.52 (1.91, 3.13) | 2.14 (-2.07, 6.53) | 2.17 (-1.59, 6.07) | 2.48 (-2.59, 7.82) |
| Gushi | Henan | 2.22 (1.67, 2.78) | 1.51 (-2.62, 5.81) | 1.10 (-4.11, 6.60) | 2.87 (-1.19, 7.09) |
| Hua | Henan | 2.55 (1.70, 3.40) | 3.09 (1.85, 4.34) | 1.83 (-1.65, 5.44) | 6.40 (0.72, 12.41) |
| Huaibin | Henan | 1.99 (1.43, 2.55) | 2.90 (-1.23, 7.21) | 1.87 (-2.04, 5.94) | 2.37 (-2.39, 7.36) |
| Huaiyang | Henan | 1.86 (1.20, 2.52) | 0.08 (-4.90, 5.32) | 0.20 (-3.30, 3.83) | 4.41 (0.19, 8.82) |
| Huangchuan | Henan | 2.18 (1.50, 2.87) | 1.35 (-3.90, 6.89) | 2.55 (-2.66, 8.04) | 2.44 (-2.87, 8.03) |
| Hui | Henan | 0.97 (0.26, 1.68) | -2.24 (-7.23, 3.01) | -0.45 (-2.88, 2.04) | -0.37 (-5.55, 5.09) |
| Huojia | Henan | 2.73 (1.97, 3.49) | -0.68 (-6.58, 5.60) | 3.16 (1.08, 5.28) | 3.24 (-1.53, 8.23) |
| Jia | Henan | 1.73 (1.17, 2.29) | 0.00 (-4.31, 4.50) | 2.44 (-1.65, 6.70) | 1.53 (-2.18, 5.39) |
| Jiyuan | Henan | 0.02 (-1.18, 1.23) | -2.29 (-14.62, 11.82) | 2.36 (-6.25, 11.75) | -0.20 (-5.83, 5.77) |
| Jun | Henan | 2.97 (2.47, 3.46) | 2.76 (-2.40, 8.20) | 2.90 (-0.09, 5.98) | 3.51 (-0.51, 7.69) |
| Lankao | Henan | 4.43 (3.16, 5.72) | 1.49 (-5.45, 8.94) | 4.36 (0.41, 8.46) | 6.15 (-0.96, 13.77) |
| Lingbao | Henan | 1.72 (0.69, 2.76) | 1.50 (-1.72, 4.81) | 3.10 (-4.94, 11.81) | -3.52 (-10.13, 3.58) |
| Linying | Henan | 3.08 (2.15, 4.01) | 1.02 (-5.45, 7.94) | 2.30 (-2.99, 7.89) | 6.94 (2.59, 11.47) |
| Linzhou | Henan | 2.22 (1.56, 2.89) | 4.73 (-0.98, 10.78) | 2.07 (-0.97, 5.20) | 0.73 (-5.37, 7.21) |
| Luanchuan | Henan | 1.01 (0.07, 1.96) | 3.55 (-0.39, 7.64) | -0.38 (-5.44, 4.95) | 2.05 (-5.01, 9.63) |
| Luoning | Henan | 3.36 (2.57, 4.16) | 7.04 (3.44, 10.77) | 3.36 (-0.81, 7.70) | 0.79 (-3.25, 5.01) |
| Luoshan | Henan | 2.64 (1.95, 3.34) | 3.04 (-0.01, 6.18) | 4.23 (-1.08, 9.82) | 0.83 (-4.37, 6.32) |
| Lushan | Henan | 2.15 (1.49, 2.83) | 0.27 (-3.37, 4.05) | 3.17 (-0.01, 6.44) | 2.49 (-1.21, 6.34) |
| Lushi | Henan | 3.09 (2.26, 3.92) | 2.33 (-2.73, 7.66) | 2.46 (-1.03, 6.08) | 1.91 (-2.84, 6.88) |
| Luyi | Henan | 3.10 (2.22, 3.98) | 1.99 (-0.83, 4.89) | 4.43 (-1.63, 10.86) | 0.02 (-6.73, 7.26) |
| Mengjin | Henan | 4.01 (3.20, 4.83) | 1.09 (-4.93, 7.49) | 5.68 (-2.29, 14.3) | 3.28 (-1.69, 8.49) |
| Mengzhou | Henan | 1.96 (0.33, 3.63) | -6.57 (-17.28, 5.53) | 4.07 (-3.69, 12.45) | 1.90 (-3.38, 7.48) |
| Mianchi | Henan | 1.88 (0.92, 2.84) | 1.24 (-3.72, 6.45) | 5.27 (-2.99, 14.25) | -1.71 (-8.19, 5.22) |
| Minquan | Henan | 3.57 (3.06, 4.07) | 2.36 (-3.96, 9.10) | 3.63 (2.16, 5.12) | 2.97 (-0.08, 6.11) |
| Nanle | Henan | 4.10 (3.12, 5.09) | -0.15 (-5.52, 5.53) | 4.48 (1.16, 7.91) | 3.53 (0.03, 7.15) |
| Nanzhao | Henan | 1.72 (0.80, 2.65) | -1.02 (-6.24, 4.49) | 0.60 (-2.55, 3.86) | 2.34 (-2.86, 7.82) |
| Neihuang | Henan | 4.31 (2.67, 5.98) | 0.31 (-3.21, 3.96) | 1.46 (-2.28, 5.34) | 6.39 (-4.03, 17.95) |
| Neixiang | Henan | 2.25 (1.27, 3.25) | 0.58 (-3.17, 4.47) | 1.01 (-4.85, 7.23) | 4.08 (-0.48, 8.84) |
| Ningling | Henan | 2.59 (2.01, 3.16) | 1.10 (-3.77, 6.22) | 2.20 (-0.37, 4.83) | 3.54 (-1.43, 8.77) |
| Pingyu | Henan | 3.85 (3.06, 4.63) | 3.48 (-2.89, 10.28) | 2.85 (-2.10, 8.04) | 6.90 (0.53, 13.67) |
| Puyang | Henan | 3.28 (2.30, 4.28) | 1.52 (-7.25, 11.13) | 3.42 (-5.28, 12.92) | 4.86 (2.10, 7.69) |
| Qi | Henan | 2.19 (1.58, 2.80) | 0.61 (-4.70, 6.21) | 1.94 (-1.93, 5.95) | 2.42 (-1.99, 7.02) |
| Qi | Henan | 3.04 (2.02, 4.08) | 1.33 (-1.51, 4.25) | 6.35 (-0.69, 13.9) | 0.14 (-8.64, 9.76) |
| Qingfeng | Henan | 2.68 (1.61, 3.77) | 4.10 (-7.82, 17.55) | 3.29 (-0.80, 7.55) | 3.09 (-1.33, 7.71) |
| Qinyang | Henan | 2.14 (1.47, 2.82) | 1.28 (-4.11, 6.98) | 3.04 (-1.93, 8.25) | 1.98 (-2.94, 7.15) |
| Queshan | Henan | 3.38 (2.48, 4.30) | 1.75 (-2.46, 6.14) | 4.35 (-3.59, 12.94) | 1.52 (-4.41, 7.82) |
| Runan | Henan | 2.65 (2.04, 3.27) | 1.57 (-3.62, 7.03) | 4.55 (1.66, 7.53) | 0.71 (-4.64, 6.37) |
| Ruyang | Henan | 2.89 (2.48, 3.31) | 1.98 (-0.35, 4.37) | 2.88 (1.36, 4.42) | 2.30 (-1.88, 6.65) |
| Ruzhou | Henan | 1.83 (0.92, 2.74) | -0.61 (-4.25, 3.17) | 2.23 (-1.77, 6.40) | 2.82 (-3.05, 9.04) |
| Shangcai | Henan | 2.86 (2.19, 3.54) | 1.12 (-3.22, 5.67) | 2.56 (-2.20, 7.54) | 4.04 (-0.13, 8.39) |
| Shangcheng | Henan | 2.78 (2.18, 3.39) | 2.42 (-1.67, 6.67) | 3.50 (-1.60, 8.86) | 1.97 (-2.61, 6.76) |
| Shangshui | Henan | 2.26 (1.49, 3.04) | -1.24 (-5.47, 3.17) | 3.07 (-0.54, 6.81) | 3.95 (0.57, 7.45) |
| Shenqiu | Henan | 2.40 (1.77, 3.03) | 0.71 (-4.30, 5.98) | 1.93 (-1.01, 4.96) | 4.32 (-0.57, 9.45) |
| Sheqi | Henan | 1.93 (1.39, 2.48) | 2.42 (-3.10, 8.25) | 0.91 (-1.95, 3.86) | 2.38 (-2.38, 7.37) |
| Song | Henan | 2.39 (1.69, 3.10) | 4.01 (-1.79, 10.15) | 0.37 (-3.52, 4.42) | 3.14 (-1.62, 8.14) |
| Sui | Henan | 1.76 (1.11, 2.42) | 0.15 (-5.11, 5.71) | 0.91 (-2.54, 4.47) | 2.88 (-1.02, 6.93) |
| Suiping | Henan | 3.97 (3.33, 4.61) | 2.22 (0.06, 4.43) | 5.21 (-0.21, 10.91) | 2.60 (-2.96, 8.48) |
| Taikang | Henan | 2.91 (2.00, 3.83) | 0.17 (-4.38, 4.94) | 1.23 (-2.71, 5.34) | 5.76 (0.88, 10.86) |
| Taiqian | Henan | 3.36 (2.74, 3.99) | 3.65 (-3.27, 11.06) | 2.57 (-2.03, 7.39) | 4.94 (1.98, 7.99) |
| Tanghe | Henan | 2.00 (1.35, 2.65) | 1.70 (-1.94, 5.47) | 2.09 (-2.78, 7.21) | 1.76 (-3.03, 6.78) |
| Tangyin | Henan | 2.51 (1.80, 3.23) | 0.91 (-1.01, 2.87) | 1.09 (-6.57, 9.37) | 5.30 (0.70, 10.10) |
| Tongbai | Henan | 2.12 (1.38, 2.87) | 2.34 (-0.58, 5.35) | 3.46 (-3.14, 10.51) | 1.53 (-4.47, 7.90) |
| Tongxu | Henan | 2.34 (1.61, 3.09) | -0.39 (-3.93, 3.28) | 4.06 (1.32, 6.88) | 1.53 (-3.96, 7.34) |
| Weihui | Henan | 1.40 (0.45, 2.36) | 0.10 (-5.96, 6.55) | 1.70 (-3.66, 7.36) | 3.26 (-3.99, 11.06) |
| Weishi | Henan | 2.79 (1.96, 3.62) | 0.92 (-3.54, 5.58) | 3.35 (-1.68, 8.64) | 3.76 (-0.73, 8.45) |
| Wen | Henan | 2.20 (1.66, 2.75) | 2.41 (-0.11, 5.00) | 2.21 (-1.76, 6.33) | 1.56 (-3.41, 6.78) |
| Wugang | Henan | -0.24 (-1.70, 1.25) | 1.35 (-15.36, 21.35) | 0.56 (-6.78, 8.48) | 3.32 (-1.49, 8.36) |
| Wuyang | Henan | 2.72 (2.00, 3.44) | 1.80 (-1.20, 4.89) | 2.77 (-1.98, 7.76) | 3.38 (-0.15, 7.03) |
| Wuzhi | Henan | 2.35 (1.40, 3.31) | 0.27 (-3.60, 4.30) | 0.77 (-5.77, 7.76) | 5.18 (0.62, 9.95) |
| Xi | Henan | 2.33 (1.56, 3.10) | 2.53 (-3.66, 9.11) | 1.53 (-3.78, 7.12) | 5.42 (0.83, 10.21) |
| Xiangcheng | Henan | 3.13 (2.26, 4.00) | 0.07 (-2.22, 2.42) | 3.15 (-2.65, 9.29) | 5.33 (-1.23, 12.33) |
| Xiangcheng | Henan | 1.79 (1.12, 2.45) | -1.42 (-4.39, 1.64) | 2.95 (-1.42, 7.52) | 2.27 (-2.02, 6.75) |
| Xiayi | Henan | 1.83 (1.22, 2.44) | -0.57 (-4.16, 3.16) | 2.47 (-0.11, 5.12) | 3.89 (0.69, 7.18) |
| Xichuan | Henan | 2.11 (1.33, 2.89) | 0.15 (-5.40, 6.02) | 3.57 (-1.12, 8.49) | 1.46 (-4.47, 7.77) |
| Xihua | Henan | 1.94 (1.26, 2.62) | 1.20 (-0.49, 2.92) | 1.62 (-1.84, 5.21) | 4.05 (-1.58, 10.00) |
| Xin | Henan | 2.21 (1.29, 3.14) | 0.63 (-3.35, 4.77) | 3.55 (-2.53, 10.00) | 2.31 (-3.53, 8.50) |
| Xinan | Henan | 1.78 (0.80, 2.77) | -2.17 (-7.58, 3.56) | 3.18 (-4.76, 11.79) | 0.38 (-6.06, 7.26) |
| Xincai | Henan | 3.98 (2.97, 4.99) | 1.29 (-4.18, 7.08) | 3.46 (-1.75, 8.95) | 7.47 (1.96, 13.28) |
| Xingyang | Henan | 2.27 (0.97, 3.58) | -2.30 (-13.74, 10.66) | 4.83 (-2.92, 13.2) | 0.27 (-5.50, 6.40) |
| Xinmi | Henan | 3.89 (2.98, 4.81) | -0.43 (-4.56, 3.89) | 4.25 (-0.77, 9.52) | 5.18 (0.99, 9.55) |
| Xinxiang | Henan | -1.73 (-3.19, -0.25) | 2.15 (-14.36, 21.85) | -5.30 (-13.58, 3.77) | 0.96 (-2.28, 4.31) |
| Xinye | Henan | 1.18 (0.42, 1.95) | -0.34 (-3.51, 2.94) | 2.20 (-3.36, 8.08) | 0.28 (-5.78, 6.73) |
| Xinzheng | Henan | 6.27 (4.93, 7.62) | -0.26 (-7.14, 7.14) | 7.49 (1.55, 13.79) | 5.59 (-1.21, 12.86) |
| Xiping | Henan | 3.39 (2.43, 4.36) | -0.22 (-3.67, 3.35) | 3.53 (-2.59, 10.04) | 4.91 (-0.17, 10.26) |
| Xiuwu | Henan | 0.62 (-0.40, 1.65) | -1.38 (-7.02, 4.60) | 3.57 (-4.39, 12.20) | 1.89 (-2.56, 6.55) |
| Xixia | Henan | 1.96 (1.06, 2.87) | 3.09 (-0.77, 7.10) | 2.45 (-4.49, 9.89) | -1.74 (-8.34, 5.34) |
| Yanjin | Henan | 2.10 (1.40, 2.80) | 0.70 (-4.24, 5.89) | 2.77 (0.74, 4.84) | 3.38 (-0.36, 7.26) |
| Yanling | Henan | 2.06 (1.30, 2.82) | -0.32 (-2.34, 1.74) | 2.59 (-1.19, 6.52) | 2.79 (-4.59, 10.74) |
| Yanshi | Henan | 2.10 (0.60, 3.61) | -6.81 (-12.46, -0.79) | 1.91 (-2.93, 6.98) | 5.37 (0.18, 10.83) |
| Ye | Henan | 2.80 (2.02, 3.60) | 0.89 (-5.10, 7.25) | 2.95 (-5.00, 11.56) | 2.18 (-1.87, 6.40) |
| Yichuan | Henan | 1.37 (0.27, 2.48) | -5.28 (-10.34, 0.07) | 3.35 (-2.30, 9.32) | 2.21 (-1.32, 5.87) |
| Yima | Henan | 1.57 (0.03, 3.13) | 2.06 (-6.68, 11.62) | 7.09 (-9.31, 26.45) | -4.48 (-5.48, -3.48) |
| Yiyang | Henan | 4.43 (3.89, 4.97) | 3.05 (-0.80, 7.05) | 4.63 (0.70, 8.72) | 2.92 (-2.14, 8.24) |
| Yongcheng | Henan | 2.86 (1.99, 3.74) | 0.96 (-1.84, 3.84) | 4.41 (-1.36, 10.51) | 1.75 (-7.44, 11.85) |
| Yuanyang | Henan | 3.51 (2.28, 4.75) | -0.21 (-6.69, 6.73) | 2.96 (-0.38, 6.41) | 6.54 (1.87, 11.43) |
| Yucheng | Henan | 2.71 (1.75, 3.67) | -0.45 (-6.37, 5.84) | 3.90 (-2.16, 10.33) | 3.99 (-1.76, 10.07) |
| Yuzhou | Henan | 2.45 (1.90, 3.01) | 0.82 (-2.70, 4.48) | 1.90 (-3.22, 7.28) | 3.05 (-1.41, 7.70) |
| Zhecheng | Henan | 2.71 (2.24, 3.19) | 2.41 (-2.02, 7.03) | 2.75 (0.19, 5.38) | 2.22 (-2.49, 7.15) |
| Zhengyang | Henan | 2.83 (2.08, 3.59) | 1.83 (-4.07, 8.09) | 2.59 (-2.26, 7.68) | 2.69 (-4.47, 10.38) |
| Zhenping | Henan | 1.13 (0.14, 2.13) | -3.03 (-4.28, -1.77) | 2.04 (-3.48, 7.88) | 2.23 (-3.40, 8.19) |
| Zhongmou | Henan | 8.39 (7.48, 9.31) | 3.47 (1.20, 5.78) | 12.19 (11.61, 12.77) | 6.10 (-1.07, 13.78) |
| Anlu | Hubei | 3.17 (2.27, 4.08) | 0.88 (-3.77, 5.76) | 4.29 (-3.73, 12.98) | 2.01 (-3.18, 7.47) |
| Badong | Hubei | 2.87 (2.03, 3.71) | 2.30 (-4.15, 9.18) | 3.29 (-1.80, 8.64) | 1.09 (-3.50, 5.91) |
| Baokang | Hubei | 4.98 (3.72, 6.26) | 1.96 (-2.86, 7.02) | 9.08 (0.58, 18.30) | -1.85 (-8.11, 4.84) |
| Changyang | Hubei | 3.00 (2.12, 3.89) | 1.32 (-2.29, 5.07) | 5.36 (0.15, 10.85) | -0.08 (-5.50, 5.65) |
| Chibi | Hubei | 3.78 (2.57, 5.00) | -0.04 (-1.19, 1.12) | 10.34 (2.65, 18.60) | 0.56 (-6.30, 7.92) |
| Chongyang | Hubei | 2.97 (2.10, 3.85) | 0.80 (-4.38, 6.27) | 3.79 (-1.36, 9.20) | 1.23 (-4.84, 7.69) |
| Dangyang | Hubei | 5.04 (3.66, 6.45) | 1.14 (-1.88, 4.26) | 10.51 (1.66, 20.13) | -2.25 (-10.21, 6.41) |
| Danjiangkou | Hubei | 3.94 (2.76, 5.14) | 0.07 (-3.92, 4.22) | 6.84 (-2.20, 16.72) | -0.61 (-7.39, 6.66) |
| Dawu | Hubei | 3.29 (2.32, 4.27) | 0.84 (-4.62, 6.62) | 3.29 (-1.24, 8.03) | 4.88 (-1.48, 11.65) |
| Daye | Hubei | 4.09 (2.96, 5.24) | -0.26 (-3.48, 3.06) | 9.18 (-1.31, 20.79) | 2.38 (-3.28, 8.37) |
| Enshi | Hubei | 4.12 (3.03, 5.22) | 0.12 (-7.44, 8.29) | 4.64 (-2.75, 12.61) | 3.46 (-2.38, 9.65) |
| Fang | Hubei | 4.16 (3.18, 5.14) | 1.69 (-3.85, 7.55) | 4.38 (-0.60, 9.61) | 2.15 (-2.38, 6.90) |
| Gongan | Hubei | 2.64 (1.64, 3.65) | -0.89 (-4.33, 2.67) | 3.52 (-3.54, 11.11) | 2.83 (-2.85, 8.83) |
| Guangshui | Hubei | 2.73 (1.80, 3.67) | 1.23 (-2.73, 5.35) | 5.07 (-3.13, 13.97) | 1.01 (-5.31, 7.76) |
| Gucheng | Hubei | 4.44 (3.20, 5.69) | 0.60 (-6.24, 7.94) | 9.06 (1.70, 16.96) | 0.19 (-7.20, 8.17) |
| Hanchuan | Hubei | 4.20 (3.28, 5.13) | 0.50 (-2.82, 3.93) | 7.90 (0.37, 16.00) | 1.32 (-3.22, 6.07) |
| Hefeng | Hubei | 2.53 (1.65, 3.42) | 0.61 (-5.06, 6.61) | 3.91 (-1.04, 9.11) | 0.14 (-5.03, 5.59) |
| Hongan | Hubei | 2.72 (1.78, 3.67) | 1.86 (-1.85, 5.72) | 5.10 (0.99, 9.37) | 0.14 (-6.85, 7.65) |
| Honghu | Hubei | 2.44 (1.54, 3.35) | -0.14 (-3.27, 3.09) | 4.12 (-0.25, 8.68) | 4.85 (-0.07, 10.01) |
| Huangmei | Hubei | 2.34 (1.64, 3.06) | 0.17 (-4.95, 5.56) | 3.68 (-0.76, 8.32) | 1.44 (-4.19, 7.40) |
| Jiangling | Hubei | 3.14 (2.30, 3.98) | 0.87 (-2.83, 4.71) | 1.56 (-4.01, 7.44) | 2.91 (-3.76, 10.05) |
| Jianli | Hubei | 2.60 (1.65, 3.55) | -0.36 (-3.93, 3.34) | 3.27 (-2.62, 9.51) | 2.47 (-3.95, 9.31) |
| Jianshi | Hubei | 3.61 (2.69, 4.54) | 1.45 (-3.08, 6.20) | 4.80 (0.83, 8.93) | 0.59 (-5.57, 7.15) |
| Jiayu | Hubei | 5.31 (4.21, 6.43) | 1.62 (-1.34, 4.67) | 9.07 (1.56, 17.15) | 3.18 (-5.69, 12.88) |
| Jingshan | Hubei | 3.97 (2.91, 5.04) | 3.28 (-0.63, 7.35) | 8.9 (2.54, 15.67) | -1.25 (-8.37, 6.41) |
| Laifeng | Hubei | 4.07 (2.99, 5.16) | 1.15 (-3.91, 6.48) | 5.09 (-1.68, 12.32) | 1.36 (-4.38, 7.45) |
| Laohekou | Hubei | 4.68 (3.62, 5.74) | 2.59 (-3.78, 9.37) | 7.81 (0.43, 15.74) | 0.53 (-7.72, 9.51) |
| Lichuan | Hubei | 4.13 (2.98, 5.28) | 0.56 (-3.78, 5.09) | 5.13 (0.82, 9.62) | 3.18 (-2.78, 9.49) |
| Luotian | Hubei | 3.56 (2.73, 4.39) | 1.89 (-3.93, 8.06) | 4.10 (-0.83, 9.27) | 1.95 (-3.07, 7.22) |
| Macheng | Hubei | 4.32 (3.38, 5.26) | 1.71 (-3.94, 7.69) | 5.52 (-1.92, 13.53) | 1.44 (-4.95, 8.26) |
| Nanzhang | Hubei | 4.31 (3.48, 5.15) | 2.12 (-3.00, 7.51) | 6.11 (1.90, 10.50) | 0.85 (-5.96, 8.16) |
| Qianjiang | Hubei | 3.15 (2.25, 4.06) | -1.06 (-3.56, 1.50) | 5.89 (-1.23, 13.53) | 1.92 (-4.57, 8.85) |
| Qichun | Hubei | 2.50 (1.65, 3.36) | 0.50 (-5.20, 6.54) | 3.12 (-1.96, 8.48) | 1.25 (-5.22, 8.15) |
| Shayang | Hubei | 3.59 (2.68, 4.51) | 2.15 (-1.33, 5.76) | 6.28 (-1.93, 15.18) | -0.02 (-6.44, 6.83) |
| Shennongjia | Hubei | 3.45 (2.40, 4.50) | 2.68 (-3.80, 9.60) | 3.77 (-4.06, 12.24) | 1.71 (-4.59, 8.44) |
| Shishou | Hubei | 2.05 (0.99, 3.12) | -2.19 (-5.47, 1.20) | 3.59 (-3.06, 10.70) | 2.06 (-2.85, 7.23) |
| Songzi | Hubei | 2.98 (1.99, 3.99) | -0.78 (-5.78, 4.49) | 4.14 (-2.04, 10.71) | 2.74 (-3.36, 9.22) |
| Tianmen | Hubei | 2.75 (1.88, 3.63) | -1.20 (-4.07, 1.74) | 5.61 (0.48, 11.00) | 2.45 (-3.27, 8.50) |
| Tongcheng | Hubei | 2.86 (2.09, 3.63) | 1.57 (-2.46, 5.76) | 3.58 (-2.01, 9.49) | 2.25 (-3.85, 8.73) |
| Tongshan | Hubei | 3.58 (2.80, 4.37) | 4.17 (-0.06, 8.57) | 5.46 (-1.26, 12.64) | 0.33 (-5.65, 6.68) |
| Tuanfeng | Hubei | 3.17 (2.28, 4.06) | 0.66 (-3.45, 4.95) | 7.07 (0.67, 13.88) | 1.97 (-3.51, 7.77) |
| Wufeng | Hubei | 3.11 (2.17, 4.07) | 0.08 (-5.16, 5.61) | 5.65 (-0.53, 12.22) | 0.59 (-4.23, 5.65) |
| Wuxue | Hubei | 3.60 (2.52, 4.69) | 0.92 (-0.96, 2.83) | 8.31 (-0.35, 17.73) | 1.08 (-3.72, 6.12) |
| Xianfeng | Hubei | 3.66 (2.75, 4.57) | 1.23 (-2.17, 4.74) | 4.07 (-0.97, 9.37) | 1.45 (-5.28, 8.66) |
| Xiantao | Hubei | 3.08 (1.97, 4.19) | -2.53 (-4.76, -0.23) | 6.30 (1.29, 11.56) | 3.91 (-3.42, 11.80) |
| Xiaochang | Hubei | 3.23 (2.45, 4.02) | 0.67 (-3.99, 5.56) | 3.76 (-0.90, 8.65) | 2.79 (-0.77, 6.49) |
| Xingshan | Hubei | 3.87 (2.88, 4.87) | 1.41 (-3.24, 6.28) | 6.61 (0.28, 13.34) | -0.41 (-7.64, 7.38) |
| Xishui | Hubei | 2.96 (2.24, 3.69) | 1.38 (-3.69, 6.72) | 4.77 (-0.21, 10.00) | 1.76 (-3.12, 6.89) |
| Xuanen | Hubei | 2.62 (1.81, 3.43) | 1.93 (-3.06, 7.17) | 2.71 (-1.59, 7.19) | 0.71 (-3.72, 5.34) |
| Yangxin | Hubei | 3.26 (2.37, 4.17) | 2.13 (-1.91, 6.32) | 4.29 (-1.78, 10.74) | 3.51 (-2.24, 9.60) |
| Yicheng | Hubei | 4.75 (3.61, 5.91) | 2.98 (-2.34, 8.59) | 8.65 (1.08, 16.78) | -0.79 (-9.09, 8.27) |
| Yidu | Hubei | 6.23 (4.81, 7.66) | 2.58 (-1.31, 6.63) | 12.01 (2.14, 22.84) | -0.86 (-8.94, 7.93) |
| Yingcheng | Hubei | 3.27 (2.16, 4.38) | -1.01 (-6.11, 4.36) | 6.00 (-0.30, 12.7) | 1.83 (-4.53, 8.62) |
| Yingshan | Hubei | 2.45 (1.43, 3.48) | 1.35 (-2.70, 5.57) | 4.60 (-1.96, 11.59) | 0.51 (-5.46, 6.87) |
| Yuanan | Hubei | 5.55 (3.9, 7.22) | 3.86 (-0.85, 8.78) | 11.67 (3.22, 20.82) | -4.79 (-12.74, 3.89) |
| Yunmeng | Hubei | 1.56 (0.85, 2.27) | -2.14 (-6.11, 2.01) | 3.86 (0.69, 7.14) | 0.53 (-4.53, 5.86) |
| Yunxi | Hubei | 3.76 (2.97, 4.56) | 1.28 (-3.25, 6.03) | 5.46 (4.64, 6.29) | 2.34 (-4.45, 9.62) |
| Zaoyang | Hubei | 4.65 (3.46, 5.85) | 1.33 (-4.01, 6.97) | 8.43 (2.50, 14.70) | -1.45 (-9.68, 7.54) |
| Zhijiang | Hubei | 5.59 (4.14, 7.05) | 0.17 (-5.20, 5.84) | 12.48 (3.05, 22.77) | 0.02 (-6.94, 7.50) |
| Zhongxiang | Hubei | 3.49 (2.56, 4.44) | 2.09 (-1.32, 5.62) | 7.16 (-0.85, 15.81) | -0.51 (-6.66, 6.05) |
| Zhushan | Hubei | 3.76 (2.97, 4.56) | 2.32 (-2.68, 7.59) | 4.59 (0.51, 8.84) | 1.06 (-4.05, 6.44) |
| Zhuxi | Hubei | 4.32 (3.13, 5.52) | 1.77 (-2.47, 6.19) | 4.16 (-1.77, 10.46) | -0.19 (-6.72, 6.81) |
| Zigui | Hubei | 3.77 (2.44, 5.11) | 0.12 (-5.83, 6.45) | 5.36 (-0.96, 12.07) | -1.68 (-9.33, 6.60) |
| Anhua | Hunan | 3.43 (2.58, 4.28) | 1.57 (-3.68, 7.11) | 3.14 (-1.15, 7.61) | 2.38 (-3.72, 8.86) |
| Anren | Hunan | 2.25 (1.19, 3.32) | 0.97 (-2.91, 5.01) | 2.59 (-5.84, 11.78) | 1.10 (-3.48, 5.90) |
| Anxiang | Hunan | 2.68 (1.73, 3.64) | -0.81 (-3.57, 2.04) | 2.70 (-2.07, 7.71) | 1.43 (-3.62, 6.76) |
| Baojing | Hunan | 2.45 (1.53, 3.38) | 1.96 (-3.13, 7.32) | 1.23 (-2.91, 5.55) | 2.92 (-2.30, 8.41) |
| Chaling | Hunan | 3.22 (2.03, 4.42) | 0.48 (-3.81, 4.96) | 6.48 (-2.27, 16.01) | -1.35 (-6.91, 4.54) |
| Changning | Hunan | 3.46 (2.65, 4.28) | 0.43 (-3.71, 4.74) | 6.12 (1.58, 10.86) | 1.81 (-3.08, 6.94) |
| Changsha | Hunan | 7.00 (5.44, 8.59) | 0.71 (-4.83, 6.57) | 12.95 (0.30, 27.19) | 3.09 (-2.54, 9.04) |
| Chengbu | Hunan | 2.24 (1.02, 3.46) | -0.76 (-4.62, 3.27) | 3.11 (-3.67, 10.37) | 0.68 (-3.69, 5.25) |
| Chenxi | Hunan | 2.67 (2.04, 3.31) | 1.02 (-3.51, 5.76) | 3.01 (-1.58, 7.83) | 0.50 (-3.28, 4.43) |
| Cili | Hunan | 3.21 (2.34, 4.08) | 1.63 (-2.12, 5.51) | 2.57 (-1.69, 7.01) | 2.46 (-3.94, 9.28) |
| Dao | Hunan | 3.44 (2.49, 4.39) | 0.76 (-3.74, 5.47) | 4.30 (-1.30, 10.21) | 1.64 (-3.73, 7.31) |
| Dongan | Hunan | 2.79 (1.98, 3.60) | 0.94 (-3.09, 5.14) | 3.39 (-2.27, 9.38) | 2.59 (-1.48, 6.82) |
| Dongkou | Hunan | 2.49 (1.79, 3.19) | 1.49 (-2.33, 5.46) | 3.06 (-1.43, 7.76) | 1.71 (-1.62, 5.16) |
| Fenghuang | Hunan | 3.61 (2.80, 4.42) | 3.36 (0.44, 6.37) | 3.45 (-1.30, 8.43) | 1.32 (-4.90, 7.95) |
| Guidong | Hunan | 2.12 (1.14, 3.10) | -0.36 (-6.61, 6.31) | 2.04 (-3.83, 8.27) | 1.43 (-3.26, 6.35) |
| Guiyang | Hunan | 3.81 (2.74, 4.90) | 0.60 (-3.41, 4.78) | 7.12 (0.38, 14.32) | -0.51 (-7.07, 6.53) |
| Guzhang | Hunan | 2.75 (1.68, 3.83) | 1.11 (-3.21, 5.63) | 3.91 (-3.20, 11.54) | 0.31 (-4.73, 5.62) |
| Hanshou | Hunan | 3.28 (2.50, 4.06) | 1.22 (-1.16, 3.66) | 4.73 (0.35, 9.30) | 2.68 (-3.12, 8.83) |
| Hengdong | Hunan | 3.47 (2.60, 4.35) | 1.61 (-3.96, 7.51) | 4.38 (-0.32, 9.29) | 1.16 (-4.90, 7.61) |
| Hengshan | Hunan | 3.25 (2.38, 4.13) | 0.40 (-4.44, 5.48) | 4.22 (-1.82, 10.63) | 2.54 (-2.58, 7.94) |
| Hengyang | Hunan | 3.19 (2.39, 4.00) | 0.88 (-3.67, 5.64) | 3.61 (-0.44, 7.83) | 1.70 (-3.62, 7.31) |
| Hongjiang | Hunan | 4.06 (2.57, 5.56) | -0.16 (-6.30, 6.39) | 4.74 (-1.95, 11.89) | 4.94 (-4.70, 15.55) |
| Huarong | Hunan | 3.07 (2.19, 3.97) | 1.59 (-2.10, 5.42) | 2.90 (-3.04, 9.21) | -0.25 (-6.19, 6.06) |
| Huayuan | Hunan | 1.83 (0.92, 2.75) | 3.80 (1.64, 6.00) | -0.12 (-3.64, 3.54) | 5.00 (-2.14, 12.65) |
| Huitong | Hunan | 3.12 (2.19, 4.06) | 1.65 (-3.09, 6.64) | 4.71 (-0.70, 10.42) | 1.50 (-3.00, 6.22) |
| Jiahe | Hunan | 4.06 (3.05, 5.09) | 0.40 (-3.28, 4.23) | 6.95 (-0.84, 15.35) | 1.96 (-3.15, 7.34) |
| Jianghua | Hunan | 3.70 (2.56, 4.85) | 0.00 (-5.10, 5.38) | 5.34 (-0.42, 11.43) | 0.61 (-5.21, 6.80) |
| Jiangyong | Hunan | 3.58 (2.62, 4.56) | 0.67 (-3.14, 4.62) | 3.48 (-2.29, 9.59) | 2.53 (-4.11, 9.62) |
| Jin | Hunan | 4.65 (3.56, 5.76) | -0.35 (-2.76, 2.12) | 7.71 (-0.97, 17.16) | 2.43 (-1.77, 6.81) |
| Jingzhou | Hunan | 2.92 (2.13, 3.72) | 2.87 (-0.35, 6.19) | 4.35 (-1.14, 10.14) | 0.06 (-4.61, 4.96) |
| Jishou | Hunan | 3.61 (2.17, 5.06) | -1.63 (-7.48, 4.59) | 7.65 (3.60, 11.85) | 3.75 (-4.03, 12.16) |
| Lanshan | Hunan | 3.36 (2.48, 4.25) | 1.02 (-3.32, 5.55) | 4.91 (0.09, 9.98) | 1.41 (-3.39, 6.45) |
| Leiyang | Hunan | 2.62 (1.74, 3.51) | 0.03 (-4.62, 4.92) | 3.45 (-2.48, 9.74) | 0.50 (-4.41, 5.66) |
| Lengshuijiang | Hunan | 3.01 (1.70, 4.34) | -2.52 (-5.20, 0.23) | 9.09 (-1.28, 20.55) | -0.91 (-5.35, 3.74) |
| Li | Hunan | 3.52 (2.28, 4.77) | -1.57 (-5.04, 2.02) | 6.58 (-0.84, 14.56) | 2.64 (-2.51, 8.06) |
| Lianyuan | Hunan | 2.83 (1.99, 3.67) | -0.02 (-3.59, 3.69) | 4.74 (-1.18, 11.03) | 1.77 (-2.22, 5.92) |
| Liling | Hunan | 3.94 (2.95, 4.94) | -0.12 (-4.11, 4.03) | 5.76 (-0.57, 12.50) | 2.81 (-3.53, 9.56) |
| Linli | Hunan | 3.23 (2.38, 4.09) | -0.07 (-1.68, 1.57) | 4.14 (-1.79, 10.42) | 3.28 (-1.09, 7.84) |
| Linwu | Hunan | 2.75 (1.58, 3.93) | -1.26 (-5.83, 3.54) | 3.44 (-1.79, 8.94) | 2.52 (-4.41, 9.95) |
| Linxiang | Hunan | 3.99 (3.09, 4.89) | 3.71 (-0.62, 8.24) | 5.38 (-2.13, 13.46) | 0.82 (-4.47, 6.41) |
| Liuyang | Hunan | 4.51 (3.53, 5.50) | 1.82 (-1.76, 5.53) | 6.71 (-0.23, 14.12) | -0.12 (-7.58, 7.95) |
| Longhui | Hunan | 2.97 (2.30, 3.65) | 1.31 (-3.39, 6.25) | 3.32 (-0.93, 7.76) | 2.71 (-0.79, 6.33) |
| Longshan | Hunan | 3.65 (2.54, 4.76) | 1.28 (-3.64, 6.46) | 3.43 (-1.90, 9.05) | 4.44 (-1.31, 10.52) |
| Lukou | Hunan | 3.42 (2.51, 4.34) | 0.49 (-3.92, 5.09) | 4.94 (-0.58, 10.78) | 0.88 (-6.17, 8.46) |
| Luxi | Hunan | 2.82 (2.11, 3.54) | 3.84 (-2.04, 10.08) | 1.39 (-4.34, 7.46) | 0.98 (-3.41, 5.57) |
| Mayang | Hunan | 3.57 (2.50, 4.65) | 2.15 (-1.96, 6.42) | 2.81 (-2.42, 8.33) | 1.91 (-3.44, 7.55) |
| Miluo | Hunan | 4.58 (3.56, 5.61) | 2.64 (-1.88, 7.38) | 7.56 (-0.15, 15.87) | -1.00 (-5.46, 3.68) |
| Nan | Hunan | 3.26 (2.47, 4.06) | 1.36 (-3.85, 6.86) | 4.96 (0.87, 9.21) | 1.57 (-3.56, 6.99) |
| Ningxiang | Hunan | 5.25 (4.11, 6.41) | 1.89 (-3.27, 7.31) | 8.10 (0.60, 16.17) | -0.93 (-7.54, 6.16) |
| Ningyuan | Hunan | 3.81 (2.63, 5.00) | -0.03 (-4.94, 5.14) | 3.60 (-0.78, 8.18) | 7.54 (0.54, 15.03) |
| Pingjiang | Hunan | 3.30 (2.42, 4.19) | 1.59 (-2.84, 6.22) | 4.52 (-1.37, 10.76) | 2.14 (-3.47, 8.09) |
| Qidong | Hunan | 2.66 (1.78, 3.54) | 0.47 (-3.05, 4.11) | 3.23 (-0.94, 7.58) | 1.14 (-3.33, 5.83) |
| Qiyang | Hunan | 3.84 (2.77, 4.92) | 0.30 (-3.28, 4.01) | 3.90 (-0.77, 8.78) | 3.07 (-1.21, 7.55) |
| Rucheng | Hunan | 2.20 (0.87, 3.54) | -4.31 (-10.85, 2.71) | 3.98 (0.79, 7.28) | 2.76 (-2.02, 7.78) |
| Sangzhi | Hunan | 2.72 (1.92, 3.54) | 4.27 (0.82, 7.83) | 1.11 (-2.44, 4.79) | 2.23 (-5.52, 10.61) |
| Shaodong | Hunan | 3.98 (3.09, 4.87) | 1.13 (-3.46, 5.94) | 4.86 (-0.45, 10.45) | 4.48 (-0.33, 9.53) |
| Shaoshan | Hunan | 3.43 (2.30, 4.57) | 0.77 (-1.20, 2.78) | 7.85 (-1.98, 18.67) | -1.56 (-8.41, 5.82) |
| Shaoyang | Hunan | 2.82 (2.14, 3.50) | 1.30 (-3.28, 6.09) | 3.53 (-0.09, 7.27) | 2.36 (-1.53, 6.40) |
| Shimen | Hunan | 3.45 (2.49, 4.41) | -0.20 (-4.38, 4.16) | 4.13 (-1.97, 10.61) | 1.55 (-4.12, 7.57) |
| Shuangfeng | Hunan | 3.22 (2.36, 4.10) | 0.54 (-3.11, 4.34) | 5.38 (-1.21, 12.42) | 1.06 (-3.68, 6.04) |
| Shuangpai | Hunan | 2.24 (1.06, 3.43) | -0.18 (-5.35, 5.28) | 2.75 (-3.51, 9.42) | 2.66 (-4.28, 10.12) |
| Suining | Hunan | 2.51 (1.45, 3.58) | 1.34 (-2.91, 5.76) | 1.94 (-3.81, 8.04) | 2.64 (-3.44, 9.10) |
| Taojiang | Hunan | 3.04 (2.21, 3.87) | 0.93 (-3.96, 6.06) | 3.44 (-1.80, 8.96) | 1.31 (-4.55, 7.53) |
| Taoyuan | Hunan | 3.51 (2.30, 4.73) | 0.14 (-2.44, 2.79) | 4.14 (-3.67, 12.59) | -0.35 (-5.98, 5.61) |
| Tongdao | Hunan | 3.16 (2.34, 3.98) | 1.70 (-3.22, 6.86) | 3.21 (-0.52, 7.09) | 3.83 (-0.22, 8.05) |
| Wangcheng | Hunan | 8.82 (7.11, 10.57) | 2.31 (-3.88, 8.90) | 15.71 (2.55, 30.56) | 6.69 (2.23, 11.34) |
| Wugang | Hunan | 2.98 (2.19, 3.77) | 0.52 (-3.80, 5.04) | 4.66 (0.82, 8.64) | 2.42 (-1.02, 5.98) |
| Xiangtan | Hunan | 4.09 (3.13, 5.05) | 1.25 (-2.79, 5.45) | 6.40 (-0.87, 14.20) | 1.83 (-3.21, 7.12) |
| Xiangxiang | Hunan | 3.89 (2.90, 4.89) | 0.54 (-3.09, 4.32) | 7.25 (-0.85, 16.02) | 1.28 (-4.29, 7.18) |
| Xiangyin | Hunan | 3.72 (2.72, 4.74) | 0.42 (-5.18, 6.35) | 6.68 (-0.05, 13.87) | -0.46 (-4.93, 4.22) |
| Xinhua | Hunan | 3.99 (3.03, 4.97) | 1.51 (-3.97, 7.30) | 6.04 (-0.94, 13.52) | 3.31 (-1.19, 8.02) |
| Xinhuang | Hunan | 3.92 (2.95, 4.91) | 1.47 (-3.19, 6.35) | 6.54 (1.36, 11.98) | 1.32 (-3.81, 6.72) |
| Xinning | Hunan | 2.90 (2.00, 3.80) | 0.36 (-4.39, 5.34) | 3.72 (-0.90, 8.54) | 1.23 (-3.45, 6.14) |
| Xinshao | Hunan | 2.94 (2.22, 3.67) | 1.69 (-1.35, 4.82) | 3.32 (-0.09, 6.84) | 3.98 (1.33, 6.71) |
| Xintian | Hunan | 3.49 (2.36, 4.62) | -0.10 (-4.32, 4.30) | 4.77 (0.32, 9.42) | 1.28 (-4.74, 7.69) |
| Xupu | Hunan | 3.52 (2.45, 4.60) | 0.99 (-4.23, 6.48) | 4.62 (-0.86, 10.41) | 3.47 (-3.03, 10.41) |
| Yanling | Hunan | 2.68 (1.63, 3.73) | -0.37 (-5.57, 5.11) | 3.82 (-3.00, 11.13) | 2.00 (-3.84, 8.19) |
| Yizhang | Hunan | 3.27 (2.04, 4.52) | -0.78 (-2.85, 1.32) | 8.20 (0.64, 16.33) | -0.57 (-6.65, 5.89) |
| Yongshun | Hunan | 3.57 (2.54, 4.61) | 0.94 (-2.92, 4.96) | 2.80 (-2.13, 7.99) | 3.10 (-0.86, 7.21) |
| Yongxing | Hunan | 3.91 (2.91, 4.92) | 1.56 (-2.23, 5.49) | 5.94 (-0.64, 12.96) | -1.26 (-7.54, 5.45) |
| You | Hunan | 3.40 (2.41, 4.39) | 1.45 (-1.86, 4.86) | 5.87 (-0.91, 13.12) | -0.29 (-7.92, 7.97) |
| Yuanjiang | Hunan | 2.68 (1.68, 3.69) | -0.29 (-3.15, 2.66) | 3.74 (-3.26, 11.24) | 0.39 (-7.16, 8.56) |
| Yuanling | Hunan | 2.52 (1.69, 3.37) | 1.33 (-2.54, 5.36) | 2.98 (-3.89, 10.34) | -2.35 (-6.76, 2.27) |
| Yueyang | Hunan | 3.45 (2.66, 4.25) | 2.35 (-1.36, 6.21) | 4.21 (-1.77, 10.56) | 3.22 (-3.16, 10.03) |
| Zhijiang | Hunan | 3.14 (2.29, 3.99) | 2.18 (-2.51, 7.11) | 4.20 (-2.76, 11.66) | 0.33 (-4.45, 5.35) |
| Zhongfang | Hunan | 3.99 (3.11, 4.87) | 5.14 (2.80, 7.52) | 5.40 (-2.05, 13.41) | -0.66 (-7.20, 6.34) |
| Zixing | Hunan | 3.88 (2.61, 5.17) | 1.21 (-3.54, 6.19) | 6.73 (-2.40, 16.71) | -3.22 (-10.98, 5.21) |
| Abaga Banner | Inner Mongolia | 2.77 (-1.53, 7.26) | 8.54 (5.03, 12.18) | 12.01 (0.83, 24.42) | -22.57 (-35.32, -7.31) |
| Alxa Left Banner | Inner Mongolia | 0.59 (-2.94, 4.26) | 20.44 (11.44, 30.17) | 1.40 (-9.36, 13.44) | -1.77 (-8.99, 6.02) |
| Alxa Right Banner | Inner Mongolia | -2.28 (-5.24, 0.77) | 7.53 (-3.21, 19.46) | 3.96 (-4.23, 12.85) | -11.16 (-22.98, 2.48) |
| Aohan Banner | Inner Mongolia | 3.55 (2.55, 4.57) | 3.59 (-0.97, 8.36) | 5.45 (1.39, 9.67) | -2.54 (-10.74, 6.41) |
| Ar Horqin Banner | Inner Mongolia | 4.37 (2.73, 6.03) | 0.91 (-4.63, 6.78) | 11.04 (4.57, 17.91) | -5.05 (-12.34, 2.85) |
| Arun Banner | Inner Mongolia | 2.38 (0.89, 3.88) | 5.23 (-0.98, 11.83) | 5.88 (-2.26, 14.70) | -7.04 (-13.31, -0.31) |
| Arxan | Inner Mongolia | -3.18 (-5.43, -0.88) | -9.49 (-16.6, -1.78) | -2.73 (-5.47, 0.08) | 6.76 (0.07, 13.90) |
| Bairin Left Banner | Inner Mongolia | 3.45 (2.23, 4.69) | 0.16 (-2.32, 2.70) | 10.01 (6.43, 13.70) | -2.63 (-10.25, 5.63) |
| Bairin Right Banner | Inner Mongolia | 2.61 (0.89, 4.36) | 2.02 (-0.91, 5.03) | 8.84 (4.10, 13.80) | -9.34 (-16.86, -1.14) |
| Bordered Yellow Banner | Inner Mongolia | 2.31 (-2.74, 7.62) | 20.06 (16.44, 23.80) | 9.21 (-2.59, 22.43) | -26.08 (-39.73, -9.34) |
| Dalad Banner | Inner Mongolia | 1.64 (-1.69, 5.07) | 13.34 (5.78, 21.44) | 9.01 (-0.80, 19.78) | -16.24 (-23.52, -8.27) |
| Darhan Muminggan United Banner | Inner Mongolia | 0.08 (-4.22, 4.58) | 5.24 (0.38, 10.34) | 12.18 (0.16, 25.64) | -25.63 (-36.90, -12.34) |
| Dengkou | Inner Mongolia | 3.55 (1.85, 5.27) | 2.56 (-0.48, 5.70) | 12.71 (-0.17, 27.25) | -3.33 (-11.61, 5.72) |
| Duolun | Inner Mongolia | 2.95 (-0.12, 6.11) | 13.40 (7.46, 19.67) | 7.48 (-0.88, 16.55) | -14.12 (-23.43, -3.68) |
| East Ujimqin Banner | Inner Mongolia | 2.38 (-0.92, 5.79) | 9.64 (-3.96, 25.18) | 9.45 (3.89, 15.31) | -13.64 (-29.35, 5.57) |
| Ejin Banner | Inner Mongolia | -0.60 (-4.52, 3.48) | 19.68 (11.33, 28.66) | 5.75 (-4.85, 17.52) | -4.18 (-23.06, 19.34) |
| Ejin Horo Banner | Inner Mongolia | 2.10 (-0.64, 4.90) | 21.02 (16.13, 26.11) | -6.80 (-23.99, 14.29) | -3.13 (-14.3, 9.49) |
| Erenhot | Inner Mongolia | -0.68 (-3.40, 2.12) | 5.97 (-18.62, 37.99) | -9.05 (-22.39, 6.58) | -4.95 (-16.10, 7.68) |
| Ergun | Inner Mongolia | 1.04 (-0.14, 2.23) | 0.56 (-2.15, 3.35) | 4.96 (-2.06, 12.49) | -6.37 (-13.06, 0.83) |
| Etuoke Banner | Inner Mongolia | 3.27 (1.64, 4.93) | 8.85 (-3.92, 23.31) | 7.75 (-4.62, 21.73) | -0.55 (-6.25, 5.50) |
| Etuokeqian Banner | Inner Mongolia | 7.90 (4.58, 11.31) | 7.18 (1.05, 13.68) | 22.73 (7.44, 40.19) | -9.85 (-22.59, 4.99) |
| Evenk Autonomous Banner | Inner Mongolia | 2.11 (0.58, 3.67) | 3.49 (-2.07, 9.36) | 6.64 (0.16, 13.55) | -7.63 (-14.55, -0.15) |
| Fengzhen | Inner Mongolia | -0.42 (-1.71, 0.90) | -3.78 (-8.09, 0.74) | 3.25 (-6.89, 14.51) | -7.04 (-14.80, 1.43) |
| Genhe | Inner Mongolia | -0.48 (-1.71, 0.76) | -3.72 (-9.43, 2.35) | 1.73 (-6.17, 10.29) | -5.37 (-11.36, 1.02) |
| Guyang | Inner Mongolia | -0.29 (-3.08, 2.59) | 7.97 (2.42, 13.81) | 7.26 (1.20, 13.68) | -16.01 (-25.20, -5.70) |
| Hangjin Banner | Inner Mongolia | 3.54 (1.18, 5.96) | 3.96 (-4.99, 13.76) | 9.55 (-6.00, 27.68) | -9.09 (-25.45, 10.87) |
| Hangjin Rear Banner | Inner Mongolia | 1.05 (-0.33, 2.45) | 6.38 (2.33, 10.59) | 3.69 (1.57, 5.86) | -8.06 (-12.25, -3.67) |
| Harqin Banner | Inner Mongolia | 1.69 (0.72, 2.66) | 7.42 (2.81, 12.23) | -0.20 (-3.45, 3.17) | -2.11 (-8.60, 4.84) |
| Hexigten Banner | Inner Mongolia | 3.33 (0.60, 6.14) | 8.99 (6.17, 11.89) | 10.52 (5.94, 15.30) | -13.41 (-24.80, -0.30) |
| Horinger | Inner Mongolia | -1.25 (-2.49, 0.01) | -1.51 (-3.64, 0.66) | 3.42 (-5.75, 13.49) | -4.90 (-17.51, 9.64) |
| Horqin Left Rear Banner | Inner Mongolia | 4.20 (2.74, 5.67) | 2.06 (-5.68, 10.44) | 7.82 (0.27, 15.94) | 0.12 (-13.59, 16.01) |
| Horqin Left Wing Middle Banner | Inner Mongolia | 2.73 (1.61, 3.86) | 5.05 (-2.34, 13.00) | 5.32 (-1.82, 12.97) | -3.20 (-10.15, 4.28) |
| Horqin Right Front Banner | Inner Mongolia | 2.15 (0.13, 4.22) | -8.09 (-21.05, 6.99) | 10.61 (6.21, 15.21) | -3.91 (-9.15, 1.63) |
| Horqin Right Middle Banner | Inner Mongolia | 4.25 (2.91, 5.62) | 0.50 (-6.13, 7.61) | 9.41 (6.05, 12.87) | -3.10 (-9.55, 3.81) |
| Huade | Inner Mongolia | 4.20 (2.59, 5.84) | -0.76 (-9.52, 8.86) | 6.36 (-2.13, 15.57) | -2.98 (-15.94, 11.97) |
| Huolinguole | Inner Mongolia | -0.34 (-3.62, 3.06) | 17.43 (9.23, 26.26) | -4.85 (-14.18, 5.50) | -12.89 (-21.64, -3.16) |
| Hure Banner | Inner Mongolia | 3.14 (1.48, 4.82) | 7.43 (-0.53, 16.02) | 4.92 (-2.68, 13.11) | -5.70 (-15.06, 4.69) |
| Jalaid Banner | Inner Mongolia | 3.40 (2.14, 4.68) | 1.12 (-4.46, 7.03) | 9.30 (2.16, 16.93) | -3.19 (-7.52, 1.36) |
| Jungar Banner | Inner Mongolia | 2.75 (-0.16, 5.74) | 16.02 (11.89, 20.30) | 6.73 (-3.52, 18.07) | -11.47 (-17.28, -5.25) |
| Kailu | Inner Mongolia | 3.46 (1.24, 5.72) | 4.13 (-8.99, 19.13) | 6.21 (1.06, 11.63) | -9.40 (-16.35, -1.87) |
| Liangcheng | Inner Mongolia | 0.69 (-0.85, 2.25) | 2.47 (0.47, 4.50) | 2.46 (-5.86, 11.51) | -5.99 (-18.67, 8.66) |
| Linxi | Inner Mongolia | 3.51 (2.16, 4.88) | 2.92 (0.61, 5.27) | 10.97 (6.59, 15.54) | -4.04 (-11.70, 4.29) |
| Manzhouli | Inner Mongolia | -3.20 (-5.42, -0.93) | 8.29 (-7.42, 26.67) | -8.88 (-22.38, 6.97) | -0.63 (-16.75, 18.63) |
| Molidavar Daur autonomous banner | Inner Mongolia | 1.80 (0.65, 2.96) | 1.14 (-9.16, 12.61) | 3.15 (-3.24, 9.96) | -3.80 (-10.25, 3.12) |
| Naiman Banner | Inner Mongolia | 2.31 (0.95, 3.69) | 6.27 (3.16, 9.48) | 4.63 (-2.20, 11.93) | -4.64 (-14.80, 6.74) |
| New Barag Left Banner | Inner Mongolia | -0.43 (-2.50, 1.68) | 0.32 (-7.06, 8.29) | 5.94 (-1.92, 14.43) | -11.59 (-19.91, -2.40) |
| New Barag Righr Banner | Inner Mongolia | 0.56 (-2.46, 3.68) | 10.30 (-0.25, 21.97) | 9.06 (-2.48, 21.96) | -16.13 (-26.02, -4.91) |
| Ningcheng | Inner Mongolia | 2.90 (1.98, 3.83) | 2.93 (-0.27, 6.23) | 4.96 (2.49, 7.49) | -3.48 (-9.02, 2.40) |
| Old Barag Banner | Inner Mongolia | 4.20 (2.21, 6.24) | 4.85 (-5.98, 16.92) | 9.20 (-1.00, 20.45) | -6.58 (-13.67, 1.09) |
| Ongniud Banner | Inner Mongolia | 3.18 (1.47, 4.92) | 1.07 (-3.28, 5.62) | 9.33 (2.76, 16.32) | -7.09 (-16.08, 2.87) |
| Oroqen Autonomous Banner | Inner Mongolia | 1.35 (0.24, 2.47) | -1.10 (-6.09, 4.15) | 2.72 (-5.22, 11.32) | -1.91 (-11.49, 8.71) |
| Qahar Right Front Banner | Inner Mongolia | 4.28 (2.64, 5.94) | 2.97 (-1.90, 8.08) | 10.04 (-3.79, 25.85) | 2.50 (-14.33, 22.64) |
| Qahar Right Middle Banner | Inner Mongolia | 3.76 (2.14, 5.40) | -0.23 (-5.71, 5.57) | 3.50 (-4.55, 12.22) | -4.85 (-17.87, 10.24) |
| Qahar Right Rear Banner | Inner Mongolia | 2.39 (0.83, 3.97) | -0.78 (-6.86, 5.69) | 6.30 (-3.10, 16.62) | -3.27 (-15.17, 10.30) |
| Qingshuihe | Inner Mongolia | 4.02 (1.57, 6.54) | 5.11 (4.00, 6.24) | 8.11 (1.69, 14.93) | -13.95 (-24.20, -2.32) |
| Shangdou | Inner Mongolia | 2.93 (1.07, 4.84) | -3.68 (-6.62, -0.64) | 0.49 (-8.76, 10.69) | 5.38 (-17.24, 34.17) |
| Siziwang Banner | Inner Mongolia | 3.22 (1.19, 5.30) | -2.18 (-6.61, 2.47) | 4.21 (-9.30, 19.73) | 1.26 (-22.24, 31.85) |
| Sonid Left Banner | Inner Mongolia | 1.60 (-2.20, 5.56) | 11.17 (8.12, 14.30) | 16.22 (7.98, 25.09) | -22.53 (-32.05, -11.68) |
| Sonid Right Banner | Inner Mongolia | 3.68 (1.44, 5.97) | 5.28 (2.39, 8.26) | 6.96 (-3.41, 18.43) | -11.32 (-21.58, 0.29) |
| Taipusi Banner | Inner Mongolia | 3.53 (0.92, 6.21) | 7.05 (1.13, 13.31) | 9.39 (2.87, 16.31) | -12.32 (-28.75, 7.89) |
| Tumed Left Banner | Inner Mongolia | 2.08 (0.72, 3.46) | 4.11 (-3.28, 12.07) | 5.58 (-4.08, 16.23) | -5.21 (-8.00, -2.33) |
| Tumed Right Banner | Inner Mongolia | 2.56 (-1.43, 6.71) | 11.29 (0.18, 23.64) | 8.95 (1.35, 17.13) | -18.77 (-27.65, -8.81) |
| Tuoketuo | Inner Mongolia | -0.59 (-2.59, 1.44) | 4.98 (0.30, 9.87) | 3.94 (-4.70, 13.36) | -11.98 (-21.14, -1.75) |
| Tuquan | Inner Mongolia | 3.12 (1.99, 4.25) | 0.06 (-7.18, 7.85) | 6.91 (0.63, 13.58) | -2.46 (-7.54, 2.89) |
| Ulanhot | Inner Mongolia | 2.71 (1.54, 3.88) | 3.95 (-8.72, 18.39) | 1.63 (-9.39, 14.00) | 2.46 (-2.59, 7.78) |
| Urad Front Banner | Inner Mongolia | 2.01 (0.49, 3.56) | 4.23 (-2.52, 11.45) | 6.58 (3.07, 10.22) | -6.65 (-15.54, 3.18) |
| Urad Rear Banner | Inner Mongolia | -1.41 (-4.33, 1.59) | 22.31 (5.78, 41.41) | -3.54 (-20.27, 16.71) | -4.14 (-14.85, 7.92) |
| Uxin Banner | Inner Mongolia | 6.58 (3.13, 10.16) | 25.70 (21.20, 30.37) | 15.98 (0.25, 34.18) | -9.77 (-16.16, -2.89) |
| West Ujimqin Banner | Inner Mongolia | 4.34 (2.26, 6.46) | 13.32 (6.96, 20.06) | 1.49 (-14.11, 19.93) | -2.25 (-6.35, 2.03) |
| Wuchuan | Inner Mongolia | 2.35 (-1.49, 6.35) | 0.66 (-3.65, 5.16) | 22.58 (1.28, 48.36) | -19.65 (-31.57, -5.66) |
| Wulate Middle Banner | Inner Mongolia | 3.62 (0.81, 6.50) | 12.64 (9.66, 15.70) | 6.06 (1.66, 10.65) | -8.98 (-20.79, 4.59) |
| Wuyuan | Inner Mongolia | 1.10 (0.01, 2.20) | 1.51 (-5.15, 8.63) | 3.39 (-1.14, 8.13) | -5.58 (-12.76, 2.19) |
| Xilinhot | Inner Mongolia | 0.10 (-1.09, 1.31) | 0.72 (-4.60, 6.33) | 1.92 (-9.63, 14.93) | 2.32 (-6.48, 11.95) |
| Xinghe | Inner Mongolia | 2.71 (1.20, 4.25) | -2.20 (-9.65, 5.87) | -0.14 (-10.91, 11.94) | -1.84 (-14.23, 12.32) |
| Yakeshi | Inner Mongolia | 2.40 (0.41, 4.44) | 2.68 (1.08, 4.31) | 9.18 (3.09, 15.63) | -11.20 (-17.73, -4.15) |
| Zarout Banner | Inner Mongolia | 4.47 (1.89, 7.12) | 7.38 (-0.59, 15.99) | 10.84 (2.45, 19.90) | -12.25 (-18.97, -4.98) |
| Zhalantun | Inner Mongolia | 2.44 (1.27, 3.63) | 0.81 (-5.00, 6.98) | 5.71 (-0.09, 11.85) | -3.82 (-12.92, 6.24) |
| Zhenglan Banner | Inner Mongolia | 1.93 (-0.81, 4.76) | 15.26 (4.84, 26.71) | 16.17 (6.85, 26.30) | -10.61 (-18.06, -2.49) |
| Zhengxiangbai Banner | Inner Mongolia | -0.78 (-5.66, 4.35) | -22.43 (-54.51, 32.30) | 12.79 (4.77, 21.42) | -13.91 (-28.55, 3.72) |
| Zhuozi | Inner Mongolia | 2.20 (0.57, 3.85) | -1.66 (-12.05, 9.97) | 4.00 (-6.52, 15.71) | -2.72 (-17.94, 15.32) |
| Baoying | Jiangsu | 4.11 (3.57, 4.65) | 2.97 (-1.13, 7.25) | 3.53 (0.70, 6.45) | 2.57 (-2.87, 8.31) |
| Binhai | Jiangsu | 2.62 (1.80, 3.45) | -0.26 (-6.55, 6.45) | 1.26 (-3.78, 6.57) | 4.09 (-1.07, 9.51) |
| Changshu | Jiangsu | 1.55 (0.32, 2.79) | -6.05 (-10.91, -0.92) | -0.43 (-8.27, 8.08) | 0.68 (-4.01, 5.60) |
| Dafeng | Jiangsu | 3.95 (3.05, 4.87) | 0.62 (-3.57, 4.98) | 4.85 (-2.15, 12.35) | 2.54 (-5.57, 11.35) |
| Danyang | Jiangsu | 3.17 (2.09, 4.27) | -0.49 (-9.37, 9.26) | 1.50 (-7.77, 11.7) | 0.78 (-3.78, 5.57) |
| Donghai | Jiangsu | 3.63 (2.79, 4.47) | 4.03 (-3.65, 12.32) | 5.96 (1.91, 10.16) | 2.99 (-2.61, 8.91) |
| Dongtai | Jiangsu | 3.00 (2.25, 3.77) | 0.41 (-4.01, 5.03) | 4.17 (-2.97, 11.83) | 1.55 (-4.53, 8.01) |
| Feng | Jiangsu | 3.59 (3.02, 4.16) | 3.74 (1.23, 6.31) | 5.33 (1.87, 8.90) | 0.99 (-4.32, 6.59) |
| Funing | Jiangsu | 3.12 (2.19, 4.07) | 1.64 (-5.42, 9.24) | 0.63 (-5.23, 6.86) | 3.23 (-1.22, 7.89) |
| Ganyu | Jiangsu | 3.42 (2.81, 4.03) | 3.91 (-1.71, 9.86) | 3.04 (-2.35, 8.73) | 4.07 (-0.87, 9.26) |
| Gaochun | Jiangsu | 4.58 (3.54, 5.64) | 2.62 (-6.47, 12.59) | 8.47 (2.97, 14.27) | 1.49 (-4.96, 8.37) |
| Gaoyou | Jiangsu | 4.02 (3.05, 5.01) | 2.00 (-2.60, 6.82) | 4.68 (-2.59, 12.49) | 6.06 (-0.46, 13.00) |
| Guannan | Jiangsu | 4.00 (3.29, 4.72) | 3.22 (-0.68, 7.27) | 3.29 (-4.73, 11.99) | 2.19 (-3.10, 7.77) |
| Guanyun | Jiangsu | 2.88 (2.08, 3.69) | 4.81 (-3.83, 14.23) | 3.27 (-0.57, 7.26) | 2.70 (-2.29, 7.93) |
| Haian | Jiangsu | 4.13 (3.11, 5.16) | 3.66 (-7.06, 15.63) | 5.21 (-0.09, 10.79) | 3.48 (-1.18, 8.37) |
| Haimen | Jiangsu | 4.02 (3.29, 4.75) | 6.77 (2.30, 11.43) | 1.78 (-3.01, 6.82) | 3.34 (-2.57, 9.62) |
| Hongze | Jiangsu | 4.37 (3.49, 5.25) | 3.3 (-1.93, 8.82) | 6.79 (-1.21, 15.44) | 3.09 (-4.71, 11.52) |
| Jiangdou | Jiangsu | 2.58 (1.85, 3.33) | 2.63 (-1.01, 6.41) | 1.49 (-6.90, 10.64) | 1.75 (-4.51, 8.42) |
| Jiangyan | Jiangsu | 2.42 (1.33, 3.53) | -2.46 (-8.78, 4.29) | 1.02 (-5.18, 7.62) | 6.75 (0.45, 13.45) |
| Jiangyin | Jiangsu | -1.21 (-2.95, 0.57) | -2.73 (-19.31, 17.25) | -10.34 (-18.66, -1.17) | 0.63 (-2.28, 3.63) |
| Jianhu | Jiangsu | 4.06 (3.04, 5.09) | 0.32 (-7.29, 8.55) | 3.10 (-2.69, 9.23) | -0.30 (-6.07, 5.84) |
| Jingjiang | Jiangsu | 2.48 (1.20, 3.79) | -0.42 (-9.06, 9.06) | -4.63 (-13.49, 5.13) | 3.76 (-0.34, 8.03) |
| Jinhu | Jiangsu | 4.54 (3.66, 5.43) | 3.10 (-3.00, 9.59) | 6.51 (-0.46, 13.97) | 2.02 (-5.44, 10.06) |
| Jintan | Jiangsu | 5.72 (3.89, 7.59) | -2.72 (-9.84, 4.97) | 8.14 (4.92, 11.46) | 7.89 (0.69, 15.61) |
| Jurong | Jiangsu | 5.64 (4.73, 6.56) | 1.18 (-1.52, 3.96) | 9.53 (3.69, 15.71) | 5.87 (1.21, 10.73) |
| Kunshan | Jiangsu | -1.34 (-3.34, 0.69) | -11.23 (-25.96, 6.43) | -4.23 (-16.61, 9.99) | -0.05 (-3.20, 3.21) |
| Lianshui | Jiangsu | 4.47 (3.72, 5.24) | 0.96 (-4.61, 6.86) | 5.29 (-0.43, 11.34) | 3.46 (-1.48, 8.66) |
| Lishui | Jiangsu | 7.18 (6.05, 8.31) | 2.79 (-1.74, 7.54) | 10.79 (0.87, 21.70) | 6.38 (-1.89, 15.34) |
| Liyang | Jiangsu | 3.27 (2.14, 4.41) | 3.08 (-7.54, 14.91) | 5.60 (-3.92, 16.07) | 4.28 (-0.34, 9.11) |
| Pei | Jiangsu | 2.30 (1.62, 2.98) | 0.69 (-4.27, 5.91) | 0.65 (-5.89, 7.65) | 2.04 (-2.55, 6.85) |
| Pizhou | Jiangsu | 2.90 (1.72, 4.09) | 0.48 (-3.86, 5.01) | 5.03 (-3.81, 14.67) | 3.62 (-0.96, 8.42) |
| Qidong | Jiangsu | 3.70 (2.77, 4.65) | 0.06 (-3.40, 3.65) | 5.88 (-0.89, 13.12) | 4.03 (-3.74, 12.42) |
| Rudong | Jiangsu | 3.53 (2.68, 4.38) | 0.91 (-2.38, 4.31) | 2.19 (-5.10, 10.04) | 4.43 (-2.05, 11.34) |
| Rugao | Jiangsu | 4.39 (3.80, 4.99) | 2.72 (-1.36, 6.97) | 5.19 (-0.91, 11.68) | 3.57 (-0.77, 8.11) |
| Sheyang | Jiangsu | 2.64 (1.69, 3.60) | -1.21 (-6.43, 4.30) | 2.95 (-3.07, 9.35) | 2.33 (-4.42, 9.55) |
| Shuyang | Jiangsu | 2.59 (1.99, 3.19) | 1.52 (-3.45, 6.75) | 1.58 (-2.37, 5.68) | 3.49 (-0.81, 7.98) |
| Sihong | Jiangsu | 2.79 (2.07, 3.52) | 2.85 (-2.90, 8.94) | 2.89 (-1.23, 7.18) | 1.50 (-6.97, 10.75) |
| Siyang | Jiangsu | 3.47 (2.72, 4.22) | 2.59 (-5.26, 11.09) | 3.80 (-2.49, 10.50) | 4.56 (0.24, 9.08) |
| Suining | Jiangsu | 4.14 (3.27, 5.03) | 1.16 (-6.48, 9.42) | 5.46 (-1.51, 12.93) | 2.44 (-2.96, 8.15) |
| Taicang | Jiangsu | 1.39 (-0.06, 2.86) | -2.88 (-13.94, 9.60) | -4.62 (-14.16, 5.97) | 2.75 (-5.05, 11.19) |
| Taixing | Jiangsu | 5.31 (4.62, 6.01) | 3.80 (0.35, 7.37) | 4.40 (-4.15, 13.71) | 4.45 (0.29, 8.78) |
| Tongshan | Jiangsu | 4.20 (3.42, 4.99) | 2.86 (-3.93, 10.12) | 4.67 (0.95, 8.54) | 1.61 (-3.47, 6.96) |
| Tongzhou | Jiangsu | 6.21 (5.36, 7.07) | 3.78 (-3.63, 11.77) | 6.34 (-0.87, 14.08) | 3.41 (-0.88, 7.88) |
| Wujiang | Jiangsu | 4.59 (2.61, 6.60) | -5.06 (-18.96, 11.24) | 10.14 (-5.61, 28.52) | 1.22 (-3.04, 5.68) |
| Xiangshui | Jiangsu | 4.97 (3.73, 6.22) | 2.61 (-5.34, 11.22) | 5.01 (-5.92, 17.21) | 0.38 (-4.27, 5.25) |
| Xinghua | Jiangsu | 2.67 (1.94, 3.41) | 0.98 (-2.75, 4.86) | 2.65 (-3.68, 9.40) | 2.96 (-4.29, 10.76) |
| Xinyi | Jiangsu | 4.07 (3.37, 4.78) | 2.79 (-0.66, 6.37) | 6.25 (-0.42, 13.37) | 3.33 (-1.38, 8.28) |
| Xuyi | Jiangsu | 3.89 (3.05, 4.74) | 3.16 (-3.90, 10.74) | 3.95 (-0.87, 9.00) | -0.02 (-6.56, 6.98) |
| Yangzhong | Jiangsu | 3.88 (2.69, 5.08) | -0.21 (-4.86, 4.66) | 0.69 (-9.76, 12.34) | 5.82 (-2.53, 14.87) |
| Yixing | Jiangsu | 3.41 (2.21, 4.63) | -2.49 (-13.45, 9.85) | 3.61 (-4.77, 12.71) | 4.09 (-0.79, 9.22) |
| Yizheng | Jiangsu | 4.80 (3.70, 5.91) | 2.03 (-8.73, 14.05) | 6.60 (-1.80, 15.72) | 2.20 (-4.13, 8.96) |
| Zhangjiagang | Jiangsu | -0.60 (-2.21, 1.04) | -4.28 (-22.88, 18.80) | -3.27 (-11.46, 5.69) | 1.85 (-4.30, 8.39) |
| Anfu | Jiangxi | 2.81 (2.00, 3.63) | 0.78 (-4.33, 6.16) | 3.65 (-2.05, 9.68) | 0.93 (-4.22, 6.36) |
| Anyi | Jiangxi | 2.61 (1.64, 3.59) | 2.03 (-3.18, 7.53) | 1.40 (-6.02, 9.41) | 0.22 (-7.19, 8.23) |
| Anyuan | Jiangxi | 3.08 (2.12, 4.06) | 1.33 (-5.02, 8.10) | 2.90 (-2.28, 8.35) | 4.03 (-1.62, 10.00) |
| Chaisang | Jiangxi | 5.94 (5.01, 6.88) | 2.57 (-1.56, 6.88) | 9.54 (0.22, 19.72) | 5.38 (-0.12, 11.17) |
| Chongren | Jiangxi | 2.61 (1.72, 3.51) | 0.83 (-3.58, 5.44) | 2.51 (-3.51, 8.9) | 3.31 (-2.41, 9.36) |
| Chongyi | Jiangxi | 2.69 (1.84, 3.55) | 1.80 (-2.28, 6.05) | 2.88 (-3.16, 9.30) | 0.59 (-4.99, 6.50) |
| Dayu | Jiangxi | 2.06 (1.15, 2.99) | -0.08 (-5.19, 5.31) | 2.83 (-2.24, 8.16) | 1.25 (-5.16, 8.1) |
| Dean | Jiangxi | 3.50 (2.50, 4.51) | -1.97 (-4.96, 1.11) | 6.46 (1.87, 11.25) | 1.97 (-5.16, 9.64) |
| Dexing | Jiangxi | 1.34 (0.33, 2.36) | -1.35 (-8.14, 5.95) | 1.99 (-4.43, 8.83) | -0.01 (-5.99, 6.36) |
| Dingnan | Jiangxi | 3.24 (2.39, 4.09) | 1.68 (-3.11, 6.71) | 3.88 (-1.38, 9.42) | 3.16 (-3.93, 10.77) |
| Dongxiang | Jiangxi | 4.32 (3.24, 5.41) | 0.14 (-4.74, 5.27) | 6.54 (-1.36, 15.07) | 1.23 (-2.66, 5.27) |
| Douchang | Jiangxi | 2.78 (2.09, 3.48) | 0.65 (-4.09, 5.63) | 2.73 (0.53, 4.97) | 3.78 (-0.05, 7.76) |
| Fengcheng | Jiangxi | 2.55 (1.96, 3.14) | 1.28 (-1.82, 4.47) | 3.33 (-2.06, 9.01) | 1.77 (-3.61, 7.46) |
| Fengxin | Jiangxi | 2.46 (1.21, 3.73) | -1.65 (-5.99, 2.90) | 3.34 (-3.60, 10.77) | 4.10 (-3.97, 12.85) |
| Fenyi | Jiangxi | 3.39 (2.36, 4.43) | 3.62 (0.23, 7.13) | 5.04 (-2.07, 12.67) | -2.69 (-10.31, 5.59) |
| Fuliang | Jiangxi | 3.50 (2.63, 4.39) | 1.85 (-3.68, 7.71) | 6.48 (-0.18, 13.59) | 1.43 (-4.67, 7.92) |
| Gan | Jiangxi | 3.41 (2.60, 4.22) | 1.78 (-2.41, 6.15) | 5.47 (-0.51, 11.80) | 1.15 (-3.40, 5.92) |
| Gaoan | Jiangxi | 4.01 (3.06, 4.98) | 1.35 (-0.89, 3.65) | 4.53 (-1.17, 10.56) | 6.34 (1.28, 11.65) |
| Guangchang | Jiangxi | 3.59 (2.67, 4.51) | 0.91 (-4.86, 7.02) | 4.49 (-1.21, 10.51) | 1.91 (-3.02, 7.08) |
| Guangfeng | Jiangxi | 2.71 (1.86, 3.56) | 0.93 (-2.89, 4.91) | 4.43 (-1.30, 10.50) | 2.85 (-2.28, 8.25) |
| Guangxin | Jiangxi | 4.09 (3.45, 4.73) | 1.10 (-3.43, 5.84) | 4.40 (1.58, 7.31) | 4.51 (0.70, 8.46) |
| Guixi | Jiangxi | 2.51 (1.53, 3.50) | 0.74 (-4.11, 5.83) | 5.37 (-3.15, 14.63) | 2.10 (-5.09, 9.83) |
| Hengfeng | Jiangxi | 3.16 (2.51, 3.82) | 5.13 (1.36, 9.05) | 4.08 (-2.10, 10.65) | 1.46 (-3.99, 7.22) |
| Huichang | Jiangxi | 3.40 (2.56, 4.25) | 2.20 (-4.22, 9.06) | 3.05 (-0.92, 7.17) | 2.19 (-1.87, 6.41) |
| Hukou | Jiangxi | 4.94 (4.37, 5.50) | 3.40 (-0.29, 7.22) | 4.93 (0.12, 9.98) | 6.91 (2.17, 11.88) |
| Jian | Jiangxi | 3.88 (3.00, 4.76) | 1.36 (-3.21, 6.15) | 4.54 (-2.59, 12.20) | 2.32 (-2.88, 7.81) |
| Jingan | Jiangxi | 2.01 (1.18, 2.85) | 0.95 (-2.00, 3.98) | 2.64 (-2.94, 8.55) | 1.21 (-4.19, 6.91) |
| Jinggangshan | Jiangxi | 2.13 (1.15, 3.11) | -0.67 (-5.34, 4.23) | 3.78 (-2.84, 10.86) | -0.89 (-7.68, 6.40) |
| Jinxi | Jiangxi | 2.28 (1.77, 2.79) | 2.38 (0.25, 4.56) | 1.10 (-2.41, 4.73) | 1.52 (-3.83, 7.17) |
| Jinxian | Jiangxi | 2.27 (1.43, 3.11) | 1.80 (-4.46, 8.47) | 3.12 (-3.33, 9.99) | -0.26 (-6.75, 6.69) |
| Jishui | Jiangxi | 3.25 (2.48, 4.02) | 1.18 (-4.17, 6.83) | 4.60 (-0.66, 10.13) | 1.21 (-4.02, 6.73) |
| Le'an | Jiangxi | 2.51 (1.64, 3.39) | 1.41 (-5.14, 8.42) | 1.20 (-3.20, 5.81) | 1.31 (-3.12, 5.94) |
| Leping | Jiangxi | 2.46 (1.86, 3.07) | 1.65 (-1.16, 4.53) | 2.82 (-2.12, 8.02) | 1.71 (-3.55, 7.25) |
| Lianhua | Jiangxi | 2.25 (1.39, 3.11) | 0.19 (-5.20, 5.90) | 2.94 (-3.40, 9.70) | 2.21 (-2.02, 6.63) |
| Lichuan | Jiangxi | 2.80 (2.11, 3.49) | 1.90 (-3.29, 7.36) | 2.59 (-3.08, 8.60) | 2.17 (-1.78, 6.28) |
| Longnan | Jiangxi | 3.80 (2.90, 4.71) | 3.59 (-1.94, 9.43) | 5.56 (-2.26, 14.00) | 3.07 (-1.39, 7.73) |
| Lushan | Jiangxi | 3.48 (2.05, 4.92) | -0.56 (-5.95, 5.14) | -0.28 (-16.05, 18.44) | 0.54 (-5.04, 6.43) |
| Luxi | Jiangxi | 2.48 (1.55, 3.42) | 0.99 (-2.96, 5.09) | 4.56 (-0.55, 9.93) | -2.33 (-9.47, 5.37) |
| Nanchang | Jiangxi | 6.41 (4.11, 8.77) | 0.40 (-4.05, 5.06) | 10.37 (-5.64, 29.09) | 13.61 (5.80, 21.99) |
| Nancheng | Jiangxi | 2.68 (1.94, 3.42) | 0.85 (-2.90, 4.74) | 3.23 (-2.87, 9.72) | 1.16 (-2.52, 4.97) |
| Nanfeng | Jiangxi | 3.26 (2.39, 4.14) | 1.52 (-3.74, 7.07) | 4.18 (-1.40, 10.07) | 1.61 (-1.88, 5.22) |
| Nankang | Jiangxi | 3.88 (2.95, 4.82) | 2.80 (-5.07, 11.33) | 2.98 (1.41, 4.57) | 2.29 (-3.47, 8.39) |
| Ningdou | Jiangxi | 2.64 (1.82, 3.46) | 0.67 (-4.24, 5.84) | 3.45 (-1.03, 8.12) | 3.19 (-0.18, 6.68) |
| Pengze | Jiangxi | 3.67 (2.44, 4.91) | 0.30 (-4.09, 4.89) | 3.86 (-1.68, 9.72) | 3.67 (-2.48, 10.21) |
| Poyang | Jiangxi | 2.46 (1.71, 3.22) | 0.50 (-4.72, 6.00) | 2.42 (-0.27, 5.17) | 2.84 (-1.03, 6.87) |
| Quannan | Jiangxi | 2.63 (1.47, 3.81) | -0.51 (-5.40, 4.64) | 2.41 (-2.94, 8.07) | 5.63 (-0.51, 12.14) |
| Ruichang | Jiangxi | 4.00 (2.85, 5.16) | -0.87 (-6.73, 5.37) | 7.10 (0.15, 14.54) | 2.66 (-2.29, 7.87) |
| Ruijin | Jiangxi | 3.13 (2.44, 3.81) | 1.88 (-2.51, 6.48) | 4.90 (-0.26, 10.33) | 1.92 (-2.77, 6.84) |
| Shanggao | Jiangxi | 3.05 (1.99, 4.12) | -0.33 (-4.29, 3.80) | 3.68 (-3.53, 11.43) | 3.42 (-2.36, 9.53) |
| Shangli | Jiangxi | 3.03 (2.37, 3.69) | 0.33 (-3.57, 4.40) | 4.90 (0.55, 9.44) | 1.67 (-1.93, 5.40) |
| Shangyou | Jiangxi | 2.83 (1.86, 3.82) | 1.23 (-5.07, 7.95) | 2.35 (-3.91, 9.02) | 3.46 (-0.94, 8.06) |
| Shicheng | Jiangxi | 3.25 (2.47, 4.04) | 2.68 (-0.92, 6.40) | 3.09 (-0.96, 7.30) | 3.32 (-1.63, 8.52) |
| Suichuan | Jiangxi | 2.89 (2.21, 3.57) | 1.38 (-5.03, 8.24) | 3.01 (-1.65, 7.90) | 3.09 (-0.81, 7.15) |
| Taihe | Jiangxi | 3.02 (2.17, 3.88) | -0.19 (-4.82, 4.65) | 3.54 (-0.64, 7.90) | 2.81 (-2.18, 8.05) |
| Tonggu | Jiangxi | 2.68 (1.74, 3.64) | 0.18 (-4.16, 4.71) | 3.44 (-3.27, 10.61) | 2.13 (-3.11, 7.64) |
| Wanan | Jiangxi | 2.79 (1.93, 3.67) | 0.29 (-4.91, 5.77) | 3.83 (-1.86, 9.84) | 0.55 (-4.79, 6.18) |
| Wannian | Jiangxi | 2.85 (2.05, 3.65) | 1.16 (-3.06, 5.56) | 3.52 (-1.20, 8.46) | 1.77 (-3.49, 7.31) |
| Wanzai | Jiangxi | 2.79 (2.00, 3.59) | 0.77 (-3.44, 5.16) | 3.98 (-0.98, 9.18) | 2.38 (-4.35, 9.59) |
| Wuning | Jiangxi | 2.94 (1.89, 4.01) | 0.49 (-5.10, 6.42) | 2.72 (-3.26, 9.07) | 2.40 (-2.83, 7.90) |
| Wuyuan | Jiangxi | 2.49 (1.57, 3.41) | 1.14 (-1.33, 3.68) | 3.69 (-2.05, 9.77) | 1.34 (-4.01, 6.99) |
| Xiajiang | Jiangxi | 2.46 (1.61, 3.32) | 0.26 (-3.27, 3.93) | 4.03 (-2.27, 10.75) | -0.95 (-6.70, 5.16) |
| Xinfeng | Jiangxi | 3.41 (2.53, 4.28) | 1.73 (-4.43, 8.29) | 3.45 (-2.06, 9.27) | 2.48 (-0.30, 5.33) |
| Xingan | Jiangxi | 2.89 (2.12, 3.67) | 1.22 (-2.54, 5.14) | 4.03 (-1.40, 9.75) | 1.94 (-4.11, 8.37) |
| Xingguo | Jiangxi | 3.04 (2.32, 3.77) | 1.83 (-4.22, 8.26) | 2.69 (-1.73, 7.31) | 3.29 (-0.67, 7.40) |
| Xinjian | Jiangxi | 4.24 (2.65, 5.86) | 0.59 (-2.85, 4.15) | 2.95 (-6.33, 13.15) | 10.31 (0.64, 20.91) |
| Xiushui | Jiangxi | 2.51 (1.72, 3.31) | 1.22 (-5.47, 8.38) | 2.64 (-2.21, 7.73) | 3.17 (-1.13, 7.66) |
| Xunwu | Jiangxi | 2.90 (1.91, 3.91) | 1.39 (-3.77, 6.82) | 2.43 (-3.21, 8.40) | 2.70 (-2.29, 7.95) |
| Yanshan | Jiangxi | 3.09 (2.25, 3.93) | 1.09 (-4.54, 7.04) | 3.32 (-0.90, 7.72) | 2.19 (-2.13, 6.70) |
| Yifeng | Jiangxi | 3.07 (2.23, 3.92) | 0.07 (-3.95, 4.25) | 3.81 (-0.91, 8.75) | 2.38 (-3.58, 8.70) |
| Yihuang | Jiangxi | 2.40 (1.60, 3.20) | 1.05 (-5.67, 8.23) | 2.70 (-3.41, 9.19) | 2.26 (-2.66, 7.44) |
| Yiyang | Jiangxi | 2.52 (1.69, 3.35) | 1.14 (-3.41, 5.90) | 2.64 (-3.85, 9.57) | 2.61 (-1.57, 6.96) |
| Yongfeng | Jiangxi | 3.48 (2.60, 4.37) | 1.15 (-3.92, 6.49) | 4.74 (-2.68, 12.74) | 2.04 (-3.35, 7.73) |
| Yongxin | Jiangxi | 2.56 (1.70, 3.44) | 0.64 (-5.72, 7.44) | 2.70 (-2.40, 8.06) | 1.44 (-3.81, 6.99) |
| Yongxiu | Jiangxi | 2.71 (1.84, 3.60) | 2.26 (-1.34, 6.00) | 3.19 (-2.69, 9.42) | 3.41 (-2.94, 10.18) |
| Yudou | Jiangxi | 3.37 (2.61, 4.14) | 1.52 (-3.52, 6.83) | 3.76 (-1.46, 9.25) | 2.91 (-0.54, 6.48) |
| Yugan | Jiangxi | 2.78 (1.67, 3.89) | 0.53 (-5.53, 6.99) | 2.22 (-1.10, 5.65) | 6.92 (-1.52, 16.09) |
| Yujiang | Jiangxi | 3.88 (3.01, 4.76) | 2.61 (-2.69, 8.19) | 4.39 (-1.52, 10.66) | 1.77 (-4.35, 8.29) |
| Yushan | Jiangxi | 3.19 (2.46, 3.93) | 1.41 (-3.33, 6.39) | 3.33 (0.18, 6.58) | 3.85 (-0.61, 8.51) |
| Zhangshu | Jiangxi | 4.36 (3.34, 5.38) | 2.59 (-0.06, 5.31) | 8.52 (-1.41, 19.45) | 1.22 (-4.52, 7.31) |
| Zixi | Jiangxi | 2.40 (1.64, 3.16) | 1.21 (-5.23, 8.09) | 2.46 (-2.89, 8.10) | 1.99 (-2.85, 7.06) |
| Antu | Jilin | 1.22 (0.42, 2.03) | 0.94 (-3.50, 5.57) | 3.45 (-0.93, 8.03) | -3.98 (-6.59, -1.30) |
| Changbai | Jilin | 1.40 (0.45, 2.37) | -0.34 (-2.97, 2.36) | 2.87 (-3.66, 9.86) | -3.21 (-11.01, 5.27) |
| Changling | Jilin | 3.44 (1.75, 5.15) | 3.82 (-5.63, 14.21) | 5.54 (-2.56, 14.31) | -7.04 (-15.84, 2.67) |
| Daan | Jilin | 1.62 (0.56, 2.69) | -0.81 (-6.59, 5.33) | 4.07 (0.77, 7.49) | -5.15 (-10.25, 0.24) |
| Dehui | Jilin | 2.22 (1.12, 3.34) | 0.84 (-7.04, 9.39) | 4.44 (-1.98, 11.28) | -2.16 (-10.13, 6.52) |
| Dongfeng | Jilin | 2.52 (1.48, 3.57) | 0.73 (-4.52, 6.26) | 5.07 (2.81, 7.38) | -4.13 (-9.24, 1.28) |
| Dongliao | Jilin | 2.16 (0.91, 3.41) | 2.41 (-2.40, 7.46) | 3.41 (-0.73, 7.72) | -6.19 (-14.64, 3.10) |
| Dunhua | Jilin | 0.58 (-0.31, 1.49) | 0.59 (-3.00, 4.32) | 3.16 (-1.20, 7.72) | -5.59 (-9.31, -1.72) |
| Fusong | Jilin | 1.88 (0.70, 3.07) | -1.32 (-3.48, 0.88) | 5.23 (2.05, 8.50) | -4.42 (-12.97, 4.97) |
| Fuyu | Jilin | 2.33 (0.76, 3.94) | 6.40 (-2.08, 15.61) | 3.53 (-2.52, 9.95) | -6.74 (-11.74, -1.46) |
| Gongzhuling | Jilin | 2.46 (1.60, 3.32) | 1.69 (-7.55, 11.85) | 3.01 (-1.93, 8.21) | -0.87 (-5.46, 3.94) |
| Helong | Jilin | 1.17 (0.13, 2.23) | 0.98 (-3.65, 5.82) | 3.30 (0.19, 6.49) | -6.79 (-8.53, -5.03) |
| Huadian | Jilin | 0.64 (-1.09, 2.40) | 4.89 (-0.34, 10.39) | 2.53 (-4.22, 9.74) | -8.74 (-13.92, -3.24) |
| Huinan | Jilin | 0.61 (-0.18, 1.39) | 0.77 (-4.71, 6.56) | 1.17 (-4.39, 7.06) | -2.04 (-5.52, 1.57) |
| Hunchun | Jilin | 1.92 (0.63, 3.23) | 3.65 (-0.24, 7.69) | 6.66 (-0.34, 14.16) | -5.57 (-13.19, 2.71) |
| Jian | Jilin | 1.10 (-0.09, 2.31) | 5.54 (-0.16, 11.56) | 1.09 (-6.94, 9.82) | -5.65 (-11.47, 0.55) |
| Jiangyuan | Jilin | 1.21 (-0.31, 2.74) | 3.68 (-5.14, 13.32) | 1.48 (-3.11, 6.29) | -2.53 (-19.19, 17.58) |
| Jiaohe | Jilin | 1.42 (0.39, 2.47) | 3.07 (-1.38, 7.73) | 3.11 (-2.11, 8.61) | -5.44 (-10.34, -0.27) |
| Jingyu | Jilin | 2.06 (1.06, 3.07) | 4.21 (1.09, 7.42) | 3.21 (-4.22, 11.21) | -4.23 (-9.00, 0.78) |
| Jiutai | Jilin | 2.41 (1.13, 3.71) | 1.77 (-8.84, 13.61) | 2.34 (-2.51, 7.44) | -1.79 (-13.62, 11.65) |
| Linjiang | Jilin | 1.78 (0.87, 2.70) | 1.58 (-1.77, 5.05) | 4.51 (-1.22, 10.58) | -3.59 (-9.49, 2.69) |
| Lishu | Jilin | 1.75 (0.48, 3.03) | 4.03 (-6.42, 15.66) | -0.57 (-10.64, 10.64) | -0.77 (-5.30, 3.97) |
| Liuhe | Jilin | 1.64 (0.65, 2.65) | 5.05 (-3.37, 14.20) | 0.53 (-4.60, 5.93) | -3.52 (-7.17, 0.27) |
| Longjing | Jilin | 2.39 (1.18, 3.62) | 1.05 (-4.26, 6.64) | 3.94 (-0.15, 8.19) | -3.33 (-15.06, 10.02) |
| Meihekou | Jilin | 1.94 (0.89, 3.00) | 1.83 (-6.03, 10.34) | 1.69 (-5.50, 9.42) | 2.20 (-6.40, 11.58) |
| Nongan | Jilin | 2.09 (1.12, 3.07) | 2.01 (-5.74, 10.39) | 4.39 (2.57, 6.24) | -2.24 (-10.29, 6.53) |
| Panshi | Jilin | -1.73 (-2.69, -0.77) | 3.91 (2.01, 5.86) | -1.75 (-8.20, 5.15) | -5.57 (-10.63, -0.22) |
| Qian Gorlos | Jilin | -1.57 (-3.39, 0.28) | 8.18 (-0.89, 18.09) | -2.50 (-9.41, 4.94) | -7.81 (-13.91, -1.28) |
| Qianan | Jilin | 2.25 (-0.26, 4.83) | 8.44 (4.80, 12.21) | 6.48 (0.88, 12.38) | -14.04 (-23.18, -3.83) |
| Shuangliao | Jilin | 1.35 (-0.21, 2.94) | 3.39 (-3.52, 10.79) | 5.14 (-2.00, 12.80) | -7.75 (-11.33, -4.04) |
| Shulan | Jilin | 1.35 (0.64, 2.06) | 1.94 (-3.10, 7.25) | 2.06 (0.04, 4.12) | -3.16 (-7.79, 1.71) |
| Taonan | Jilin | 3.06 (1.30, 4.84) | -2.59 (-18.84, 16.9) | 6.37 (1.44, 11.53) | -5.01 (-10.25, 0.53) |
| Tonghua | Jilin | 1.97 (0.35, 3.62) | 6.24 (1.03, 11.72) | 2.82 (-4.21, 10.36) | -8.66 (-15.52, -1.25) |
| Tongyu | Jilin | 2.75 (1.21, 4.30) | -1.04 (-8.29, 6.79) | 6.48 (0.28, 13.07) | -6.32 (-11.72, -0.58) |
| Tumen | Jilin | -0.29 (-1.26, 0.68) | -4.10 (-9.29, 1.40) | 2.37 (-3.05, 8.11) | -5.23 (-9.23, -1.05) |
| Wangqing | Jilin | 1.56 (0.82, 2.31) | 1.42 (-2.84, 5.87) | 3.43 (-1.39, 8.49) | -2.74 (-6.50, 1.17) |
| Yanji | Jilin | -0.65 (-1.56, 0.27) | -3.68 (-7.50, 0.30) | -0.26 (-8.02, 8.14) | -1.33 (-4.91, 2.39) |
| Yitong | Jilin | 1.43 (0.47, 2.39) | 4.15 (-3.75, 12.69) | 2.10 (-2.47, 6.88) | -4.17 (-4.99, -3.34) |
| Yongji | Jilin | 1.26 (0.41, 2.11) | 1.72 (-5.09, 9.01) | 1.53 (-2.18, 5.40) | -3.96 (-8.50, 0.80) |
| Yushu | Jilin | 1.65 (0.51, 2.81) | 1.34 (-9.41, 13.35) | 2.47 (-3.73, 9.07) | -3.62 (-11.24, 4.64) |
| Zhenlai | Jilin | 2.07 (0.38, 3.79) | 2.74 (-4.67, 10.73) | 2.44 (-7.79, 13.80) | -8.27 (-14.63, -1.44) |
| Beipiao | Liaoning | 1.18 (-0.05, 2.43) | 3.84 (-0.16, 7.99) | 2.44 (-2.64, 7.79) | -2.53 (-7.42, 2.61) |
| Beizhen | Liaoning | 1.10 (0.43, 1.77) | -1.52 (-8.38, 5.85) | 1.94 (-0.31, 4.23) | 0.45 (-4.65, 5.82) |
| Benxi | Liaoning | 0.13 (-1.03, 1.31) | -0.76 (-7.78, 6.79) | 2.90 (-2.03, 8.07) | -5.30 (-13.59, 3.79) |
| Changhai | Liaoning | 1.14 (0.01, 2.28) | 0.62 (-3.50, 4.91) | 2.87 (-5.73, 12.25) | -3.95 (-13.76, 6.98) |
| Changtu | Liaoning | 0.97 (-0.02, 1.96) | 3.04 (-5.80, 12.70) | -0.74 (-6.01, 4.83) | -0.58 (-4.85, 3.88) |
| Chaoyang | Liaoning | 1.48 (0.25, 2.72) | 6.10 (0.70, 11.80) | 1.84 (-2.06, 5.89) | -1.20 (-5.52, 3.31) |
| Dashiqiao | Liaoning | -1.78 (-3.07, -0.47) | 0.12 (-6.08, 6.73) | -0.29 (-14.7, 16.55) | -0.94 (-6.41, 4.86) |
| Dawa | Liaoning | 5.18 (3.08, 7.32) | 3.24 (-10.07, 18.52) | 3.04 (-13.59, 22.88) | -0.37 (-3.15, 2.48) |
| Dengta | Liaoning | 2.88 (1.04, 4.76) | 2.51 (-7.80, 13.97) | 10.11 (-1.90, 23.59) | -1.44 (-9.93, 7.86) |
| Diaobingshan | Liaoning | -1.64 (-3.33, 0.09) | 0.84 (-12.89, 16.74) | -8.15 (-19.03, 4.20) | 5.96 (-0.47, 12.81) |
| Donggang | Liaoning | -0.60 (-1.65, 0.46) | 1.50 (-1.69, 4.81) | -1.15 (-8.82, 7.16) | -2.70 (-7.49, 2.33) |
| Faku | Liaoning | 1.65 (-0.18, 3.51) | 6.78 (-0.72, 14.86) | 5.03 (-2.62, 13.29) | -7.13 (-14.00, 0.30) |
| Fengcheng | Liaoning | 0.61 (-0.81, 2.04) | 3.22 (-1.93, 8.64) | 1.13 (-8.59, 11.89) | -3.26 (-9.94, 3.92) |
| Fushun | Liaoning | -0.45 (-2.40, 1.54) | 3.48 (0.86, 6.16) | 4.83 (-1.74, 11.85) | -9.08 (-20.50, 3.98) |
| Fuxin | Liaoning | 1.95 (1.07, 2.84) | 0.45 (-4.73, 5.92) | 3.01 (-2.65, 9.00) | 0.19 (-3.21, 3.70) |
| Gaizhou | Liaoning | 1.89 (0.89, 2.91) | 4.06 (-7.73, 17.36) | 1.09 (-3.88, 6.31) | 0.24 (-3.36, 3.98) |
| Haicheng | Liaoning | 0.49 (-0.48, 1.46) | 0.20 (-6.39, 7.26) | 2.50 (-4.95, 10.55) | -1.21 (-6.56, 4.46) |
| Heishan | Liaoning | 1.06 (0.22, 1.89) | 0.91 (-6.94, 9.44) | 2.21 (-1.26, 5.81) | -1.60 (-6.93, 4.04) |
| Huanren | Liaoning | 0.95 (-0.21, 2.12) | 2.57 (-2.77, 8.19) | 2.42 (-4.73, 10.11) | -5.44 (-11.18, 0.67) |
| Jianchang | Liaoning | 0.93 (0.26, 1.60) | 3.11 (-3.04, 9.66) | -1.04 (-5.36, 3.47) | -0.52 (-2.17, 1.16) |
| Jianping | Liaoning | 0.88 (-0.24, 2.00) | 3.91 (1.57, 6.31) | 1.55 (-3.97, 7.39) | 0.26 (-7.29, 8.42) |
| Kaiyuan | Liaoning | -1.17 (-3.11, 0.80) | 7.98 (1.62, 14.73) | -7.63 (-14.51, -0.19) | 0.71 (-6.00, 7.91) |
| Kangping | Liaoning | 1.60 (-0.12, 3.35) | 6.85 (1.45, 12.54) | 4.87 (-4.49, 15.14) | -4.98 (-12.08, 2.69) |
| Kazuo | Liaoning | 0.47 (-0.54, 1.49) | 6.37 (1.81, 11.14) | -2.10 (-8.91, 5.22) | 1.23 (-3.18, 5.85) |
| Kuandian | Liaoning | -0.69 (-1.38, 0.02) | 1.29 (-0.91, 3.54) | -0.60 (-4.93, 3.93) | -0.54 (-9.37, 9.16) |
| Liaoyang | Liaoning | 0.17 (-1.08, 1.43) | 0.42 (-6.46, 7.80) | 5.08 (-3.74, 14.72) | -3.90 (-12.39, 5.42) |
| Liaozhong | Liaoning | 0.70 (-0.94, 2.37) | 4.68 (-8.86, 20.23) | 1.22 (-4.82, 7.65) | -2.23 (-10.43, 6.72) |
| Linghai | Liaoning | 0.55 (-0.58, 1.70) | 2.91 (-3.72, 10.01) | 3.11 (-1.60, 8.05) | -3.09 (-7.93, 2.00) |
| Lingyuan | Liaoning | 0.19 (-0.52, 0.90) | 2.40 (-2.65, 7.70) | -0.35 (-7.65, 7.54) | -0.50 (-3.98, 3.11) |
| Panshan | Liaoning | 3.61 (2.39, 4.85) | 4.42 (-0.88, 10.01) | 3.44 (-8.86, 17.41) | 4.85 (-0.26, 10.23) |
| Pulandian | Liaoning | -0.32 (-1.43, 0.80) | 1.04 (-6.15, 8.79) | 1.95 (-5.77, 10.30) | -5.38 (-10.68, 0.23) |
| Qingyuan | Liaoning | 0.78 (-0.65, 2.22) | 3.83 (-1.08, 8.98) | 3.38 (-1.75, 8.79) | -5.85 (-14.20, 3.31) |
| Suizhong | Liaoning | 1.06 (0.37, 1.76) | -1.89 (-4.80, 1.10) | 2.46 (-2.54, 7.70) | 3.29 (1.90, 4.70) |
| Taian | Liaoning | 0.46 (-0.94, 1.87) | 1.28 (-7.86, 11.32) | 2.81 (-3.39, 9.42) | -3.66 (-12.79, 6.43) |
| Tieling | Liaoning | 0.22 (-3.00, 3.55) | 13.44 (2.18, 25.94) | -7.71 (-19.38, 5.64) | -0.83 (-5.39, 3.96) |
| Wafangdian | Liaoning | 1.92 (0.59, 3.28) | 2.77 (-0.92, 6.59) | 5.93 (0.04, 12.17) | -5.45 (-10.24, -0.41) |
| Xifeng | Liaoning | 0.88 (0.29, 1.47) | 1.88 (-4.77, 8.99) | 1.16 (-2.13, 4.56) | -0.24 (-5.11, 4.89) |
| Xinbin | Liaoning | 0.54 (-0.76, 1.85) | 1.18 (-3.51, 6.09) | 3.92 (-2.03, 10.22) | -6.14 (-14.17, 2.65) |
| Xingcheng | Liaoning | 2.83 (1.48, 4.21) | -1.58 (-12.32, 10.49) | 1.36 (-6.60, 9.99) | 0.75 (-1.77, 3.33) |
| Xinmin | Liaoning | 1.48 (-0.39, 3.39) | 1.41 (-13.80, 19.32) | 5.19 (-0.80, 11.53) | -4.98 (-13.26, 4.08) |
| Xiuyan | Liaoning | 0.09 (-0.83, 1.01) | 1.00 (-4.97, 7.35) | 1.69 (-4.08, 7.81) | -2.68 (-9.89, 5.11) |
| Yi | Liaoning | 1.79 (0.86, 2.73) | 0.25 (-9.07, 10.52) | 2.21 (0.11, 4.35) | -0.99 (-5.93, 4.20) |
| Zhangwu | Liaoning | 1.91 (0.74, 3.10) | -2.62 (-11.74, 7.45) | 4.08 (-0.86, 9.27) | -1.22 (-5.49, 3.25) |
| Zhuanghe | Liaoning | 1.11 (0.04, 2.20) | 2.63 (-0.76, 6.14) | 2.96 (-5.21, 11.84) | -2.68 (-7.20, 2.05) |
| Haiyuan | Ningxia | 7.30 (6.00, 8.61) | 5.07 (-7.75, 19.65) | 6.00 (-3.48, 16.40) | 10.27 (1.11, 20.26) |
| Helan | Ningxia | 1.75 (-0.10, 3.63) | -2.17 (-11.46, 8.11) | 2.30 (-18.07, 27.73) | -1.89 (-11.01, 8.18) |
| Jingyuan | Ningxia | 3.55 (2.54, 4.57) | 1.16 (-7.24, 10.32) | 0.85 (-7.20, 9.59) | 3.78 (1.21, 6.41) |
| Lingwu | Ningxia | 8.36 (6.27, 10.49) | 16.35 (3.22, 31.14) | 6.49 (-2.66, 16.50) | 0.42 (-10.06, 12.12) |
| Longde | Ningxia | 5.71 (4.63, 6.80) | 2.10 (-5.77, 10.63) | 5.30 (-0.69, 11.65) | 6.60 (-0.40, 14.10) |
| Pengyang | Ningxia | 7.14 (5.96, 8.33) | 2.51 (-9.52, 16.15) | 7.34 (1.98, 12.99) | 6.01 (-1.20, 13.75) |
| Pingluo | Ningxia | 2.41 (1.47, 3.36) | 4.85 (-3.82, 14.30) | 2.92 (-3.88, 10.19) | 2.02 (-5.46, 10.08) |
| Qingtongxia | Ningxia | -0.72 (-2.60, 1.18) | -6.64 (-13.34, 0.58) | 3.76 (-14.73, 26.26) | -5.38 (-12.43, 2.24) |
| Tongxin | Ningxia | 8.30 (6.94, 9.68) | 3.27 (-3.88, 10.95) | 5.63 (-5.81, 18.45) | 13.10 (4.54, 22.36) |
| Xiji | Ningxia | 5.38 (4.27, 6.49) | 1.40 (-7.37, 10.99) | 2.54 (-1.08, 6.30) | 10.62 (4.59, 16.99) |
| Yanchi | Ningxia | 8.21 (6.92, 9.52) | 6.59 (-3.15, 17.30) | 9.41 (0.19, 19.47) | 3.93 (-7.41, 16.66) |
| Yongning | Ningxia | 4.81 (3.40, 6.24) | 3.12 (-4.13, 10.91) | 4.89 (-2.89, 13.31) | -2.00 (-10.02, 6.75) |
| Zhongning | Ningxia | 5.00 (3.64, 6.39) | 2.35 (-4.43, 9.62) | 4.75 (-4.78, 15.23) | 1.47 (-6.56, 10.20) |
| Banma | Qinghai | 1.20 (-0.16, 2.58) | -1.88 (-6.23, 2.68) | 0.94 (-7.34, 9.96) | 2.25 (-4.22, 9.15) |
| Chengduo | Qinghai | 4.22 (2.16, 6.32) | -0.10 (-8.81, 9.45) | -3.99 (-18.07, 12.52) | -4.45 (-14.02, 6.18) |
| Dari | Qinghai | 2.54 (1.34, 3.75) | -2.98 (-9.81, 4.38) | 3.03 (-3.38, 9.87) | 4.05 (-0.85, 9.20) |
| Datong | Qinghai | 0.47 (-0.24, 1.19) | -3.42 (-7.43, 0.76) | 0.45 (-4.52, 5.66) | -0.80 (-5.35, 3.97) |
| Delingha | Qinghai | 5.82 (4.23, 7.43) | 2.76 (-3.91, 9.89) | 2.36 (-4.60, 9.82) | 8.42 (-8.37, 28.28) |
| Doulan | Qinghai | 9.02 (6.99, 11.09) | 0.94 (-3.63, 5.74) | 17.04 (9.27, 25.36) | -0.14 (-12.83, 14.40) |
| Gande | Qinghai | -0.12 (-1.78, 1.58) | -2.65 (-7.64, 2.62) | -1.28 (-6.32, 4.03) | 6.14 (2.37, 10.05) |
| Gangcha | Qinghai | 4.10 (2.39, 5.83) | -1.97 (-10.33, 7.17) | 8.32 (-8.08, 27.65) | -0.85 (-9.06, 8.10) |
| Geermu | Qinghai | 6.81 (3.88, 9.82) | 15.1 (-5.64, 40.4) | 16.58 (1.20, 34.31) | -3.78 (-14.80, 8.67) |
| Gonghe | Qinghai | 3.30 (1.20, 5.44) | -2.12 (-13.54, 10.8) | 8.10 (1.44, 15.19) | -9.29 (-19.06, 1.65) |
| Guide | Qinghai | 4.87 (3.42, 6.34) | 1.14 (-5.54, 8.30) | 10.14 (-4.81, 27.43) | 3.47 (-6.50, 14.50) |
| Guinan | Qinghai | 3.72 (2.49, 4.97) | 4.98 (-0.09, 10.30) | 1.19 (-8.48, 11.87) | 4.56 (-3.88, 13.74) |
| Haiyan | Qinghai | 0.26 (-3.61, 4.28) | 29.02 (1.88, 63.38) | 10.51 (-2.63, 25.43) | -13.41 (-21.85, -4.07) |
| Henan | Qinghai | 2.14 (0.71, 3.59) | -1.33 (-6.78, 4.44) | 5.02 (-4.68, 15.70) | 0.62 (-8.71, 10.90) |
| Hualong | Qinghai | 2.15 (1.24, 3.07) | -0.01 (-7.13, 7.67) | 5.62 (-2.65, 14.59) | 3.32 (0.63, 6.08) |
| Huangyuan | Qinghai | 2.51 (1.95, 3.08) | 2.84 (0.00, 5.76) | 0.90 (-0.67, 2.49) | 1.69 (-4.41, 8.17) |
| Huangzhong | Qinghai | 4.32 (2.84, 5.82) | 9.7 (1.08, 19.06) | 2.75 (-8.05, 14.83) | 2.47 (-1.38, 6.47) |
| Huzhu | Qinghai | 4.32 (3.57, 5.07) | 3.84 (-3.25, 11.44) | 6.17 (0.89, 11.72) | 1.62 (-2.92, 6.38) |
| Jianzha | Qinghai | -0.13 (-1.13, 0.89) | -4.73 (-10.98, 1.96) | -1.39 (-8.46, 6.23) | 1.76 (-3.29, 7.07) |
| Jiuzhi | Qinghai | -0.53 (-1.99, 0.95) | -3.75 (-10.16, 3.12) | -1.51 (-7.77, 5.17) | 3.12 (-1.28, 7.71) |
| Ledou | Qinghai | 5.40 (4.48, 6.33) | 3.51 (-2.50, 9.88) | 4.78 (-6.59, 17.52) | 4.78 (0.15, 9.62) |
| Maduo | Qinghai | 2.60 (0.42, 4.83) | -4.71 (-7.87, -1.44) | 7.98 (-10.88, 30.83) | -0.72 (-11.45, 11.32) |
| Maqin | Qinghai | 1.34 (0.19, 2.50) | 1.71 (-4.95, 8.83) | 2.55 (-4.18, 9.76) | -1.50 (-7.13, 4.48) |
| Menyuan | Qinghai | 3.07 (1.61, 4.56) | -2.96 (-11.90, 6.90) | 6.23 (-1.69, 14.79) | -0.81 (-7.43, 6.28) |
| Minhe | Qinghai | 4.36 (3.64, 5.09) | 4.06 (-1.48, 9.90) | 3.40 (-3.29, 10.56) | 7.89 (5.21, 10.65) |
| Nangqian | Qinghai | 2.83 (0.26, 5.47) | 1.79 (-6.70, 11.05) | -10.43 (-33.51, 20.67) | 1.64 (-4.28, 7.93) |
| Pingan | Qinghai | 5.41 (3.95, 6.89) | 9.14 (-0.04, 19.15) | 10.68 (-3.06, 26.36) | 2.36 (-6.79, 12.40) |
| Qilian | Qinghai | 2.09 (0.47, 3.73) | -2.66 (-9.20, 4.36) | 7.34 (-0.84, 16.19) | -7.54 (-14.03, -0.56) |
| Qumalai | Qinghai | 0.57 (-2.13, 3.33) | 1.49 (-9.97, 14.41) | -10.93 (-36.37, 24.70) | -2.80 (-11.69, 6.99) |
| Tianjun | Qinghai | -1.89 (-8.32, 5.00) | 27.03 (9.63, 47.20) | 4.63 (-38.24, 77.25) | 7.58 (-0.67, 16.51) |
| Tongde | Qinghai | 3.50 (2.01, 5.02) | 2.42 (-4.49, 9.83) | 3.03 (-5.51, 12.34) | 7.84 (-5.41, 22.95) |
| Tongren | Qinghai | 4.00 (1.78, 6.26) | 0.16 (-5.57, 6.24) | 12.17 (-5.18, 32.70) | 12.55 (-7.89, 37.53) |
| Wulan | Qinghai | -1.95 (-6.26, 2.56) | 25.20 (6.43, 47.28) | -16.83 (-29.89, -1.33) | 7.97 (-1.17, 17.96) |
| Xinghai | Qinghai | 3.67 (2.51, 4.84) | 6.51 (1.32, 11.96) | 5.90 (2.03, 9.92) | -2.12 (-13.14, 10.31) |
| Xunhua | Qinghai | 3.60 (2.10, 5.12) | 0.31 (-12.26, 14.68) | 5.04 (-4.90, 16.03) | 2.29 (-3.71, 8.67) |
| Yushu | Qinghai | 1.77 (-0.88, 4.48) | -3.44 (-23.57, 22.01) | -8.94 (-26.54, 12.87) | 1.87 (-9.37, 14.50) |
| Zaduo | Qinghai | 1.94 (0.06, 3.85) | -0.21 (-7.36, 7.50) | -0.78 (-20.46, 23.76) | -4.68 (-14.33, 6.05) |
| Zeku | Qinghai | 3.21 (2.00, 4.44) | 0.61 (-6.36, 8.10) | 1.03 (-4.95, 7.38) | 2.63 (-3.62, 9.27) |
| Zhiduo | Qinghai | 2.76 (-0.96, 6.62) | -2.83 (-8.43, 3.12) | -16.28 (-42.36, 21.61) | -3.14 (-16.48, 12.33) |
| Ansai | Shaanxi | 2.36 (-0.01, 4.78) | 15.71 (-1.40, 35.8) | 5.98 (-0.85, 13.28) | -4.24 (-12.42, 4.71) |
| Baihe | Shaanxi | 4.45 (3.55, 5.36) | 3.34 (-3.10, 10.21) | 6.65 (2.21, 11.28) | 3.32 (-5.73, 13.25) |
| Baishui | Shaanxi | 3.32 (2.63, 4.02) | 0.65 (-5.00, 6.64) | 1.31 (-4.25, 7.19) | 3.95 (0.90, 7.09) |
| Binzhou | Shaanxi | 5.53 (4.24, 6.82) | 10.2 (5.54, 15.07) | 7.31 (-1.05, 16.38) | 1.51 (-7.01, 10.81) |
| Changwu | Shaanxi | 5.92 (4.73, 7.12) | 6.06 (-2.17, 14.98) | 2.59 (-7.45, 13.73) | 1.93 (-5.36, 9.79) |
| Chengcheng | Shaanxi | 1.29 (0.57, 2.01) | 4.09 (-1.64, 10.16) | 1.21 (-4.31, 7.05) | -0.68 (-6.02, 4.96) |
| Chenggu | Shaanxi | 3.96 (2.97, 4.96) | 1.36 (-3.66, 6.64) | 6.59 (-1.12, 14.90) | 1.68 (-4.28, 8.00) |
| Chunhua | Shaanxi | 2.96 (2.15, 3.79) | 3.52 (0.71, 6.41) | 3.80 (-2.09, 10.05) | -0.63 (-7.91, 7.22) |
| Dali | Shaanxi | 2.39 (1.49, 3.31) | -1.62 (-8.86, 6.19) | 0.84 (-2.26, 4.04) | 3.21 (-4.22, 11.22) |
| Danfeng | Shaanxi | 3.64 (2.72, 4.57) | 1.62 (-4.39, 8.00) | 5.10 (-0.39, 10.90) | -0.56 (-6.07, 5.29) |
| Dingbian | Shaanxi | 6.95 (3.68, 10.34) | 20.99 (13.13, 29.40) | 12.61 (3.73, 22.25) | -1.62 (-7.27, 4.37) |
| Feng | Shaanxi | 4.69 (1.78, 7.69) | 6.92 (1.22, 12.94) | 10.94 (2.92, 19.58) | -13.63 (-28.77, 4.72) |
| Fengxiang | Shaanxi | 2.75 (1.86, 3.64) | -0.85 (-7.53, 6.31) | 3.43 (-3.87, 11.28) | 1.01 (-4.19, 6.48) |
| Foping | Shaanxi | 1.92 (0.78, 3.07) | -1.56 (-6.33, 3.45) | 3.60 (-2.48, 10.05) | 0.09 (-5.01, 5.46) |
| Fu | Shaanxi | 3.95 (3.16, 4.75) | 1.28 (-6.11, 9.26) | 4.99 (-0.51, 10.80) | 3.36 (-1.22, 8.15) |
| Fufeng | Shaanxi | 2.66 (2.08, 3.25) | 2.88 (-0.04, 5.88) | 0.86 (-5.77, 7.95) | 2.97 (-1.22, 7.34) |
| Fugu | Shaanxi | 8.96 (5.40, 12.65) | 19.20 (4.80, 35.57) | 7.81 (0.36, 15.82) | 0.41 (-13.39, 16.41) |
| Fuping | Shaanxi | 3.30 (2.41, 4.20) | 1.41 (-5.06, 8.31) | 1.45 (-7.32, 11.05) | 4.55 (0.97, 8.27) |
| Ganquan | Shaanxi | 0.69 (-0.46, 1.85) | 1.44 (0.47, 2.41) | 2.13 (-7.70, 13.01) | 2.86 (-8.03, 15.05) |
| Gaoling | Shaanxi | 5.05 (2.82, 7.33) | -1.77 (-15.69, 14.44) | 6.62 (-15.45, 34.45) | 2.67 (0.67, 4.72) |
| Hancheng | Shaanxi | 3.12 (1.52, 4.74) | -4.59 (-14.60, 6.60) | 8.11 (-0.94, 17.98) | 0.70 (-8.00, 10.22) |
| Hanyin | Shaanxi | 4.36 (3.50, 5.23) | 2.33 (-4.21, 9.33) | 4.77 (-2.27, 12.31) | 3.23 (-3.54, 10.49) |
| Hengshan | Shaanxi | 6.32 (4.29, 8.39) | 16.83 (12.60, 21.22) | 6.84 (-3.05, 17.75) | 7.56 (-2.12, 18.19) |
| Heyang | Shaanxi | 3.49 (2.73, 4.26) | 6.87 (1.62, 12.39) | 2.36 (-3.95, 9.09) | 1.81 (-3.18, 7.05) |
| Huangling | Shaanxi | 3.07 (1.99, 4.15) | 5.56 (-1.05, 12.62) | 4.58 (-4.23, 14.22) | 4.32 (-2.52, 11.63) |
| Huanglong | Shaanxi | 2.25 (1.25, 3.26) | 1.03 (-3.64, 5.93) | 2.68 (-2.60, 8.24) | 1.10 (-4.26, 6.76) |
| Huayin | Shaanxi | 2.03 (0.95, 3.12) | 0.67 (-8.87, 11.22) | -1.11 (-10.24, 8.95) | 0.64 (-5.82, 7.55) |
| Huazhou | Shaanxi | 1.68 (0.89, 2.47) | 1.94 (-5.38, 9.84) | 0.65 (-7.04, 8.97) | 1.00 (-2.96, 5.11) |
| Huyi | Shaanxi | 3.57 (2.12, 5.04) | -3.21 (-11.25, 5.55) | 5.21 (-7.84, 20.10) | 2.98 (-1.43, 7.60) |
| Jia | Shaanxi | 5.80 (4.50, 7.11) | 5.82 (-5.82, 18.90) | 8.18 (4.22, 12.30) | 6.78 (-8.09, 24.04) |
| Jingbian | Shaanxi | -0.96 (-2.42, 0.54) | 3.15 (-5.64, 12.77) | 7.05 (-2.28, 17.26) | -2.18 (-13.01, 10.01) |
| Jingyang | Shaanxi | 5.10 (3.96, 6.26) | 2.88 (-3.05, 9.18) | 8.62 (-4.55, 23.62) | 1.72 (-3.11, 6.79) |
| Langao | Shaanxi | 3.25 (2.26, 4.25) | 1.61 (-4.42, 8.03) | 5.49 (-0.15, 11.45) | -0.01 (-6.34, 6.75) |
| Lantian | Shaanxi | 3.72 (2.91, 4.53) | 3.79 (-4.27, 12.53) | 1.46 (-4.63, 7.94) | 5.07 (-0.99, 11.5) |
| Linyou | Shaanxi | 8.22 (6.55, 9.93) | 2.00 (-0.98, 5.08) | 16.36 (10.53, 22.50) | 4.34 (-9.31, 20.04) |
| Liquan | Shaanxi | 2.63 (1.73, 3.54) | -1.02 (-9.16, 7.85) | 2.38 (-3.20, 8.28) | 1.57 (-5.21, 8.84) |
| Liuba | Shaanxi | 2.19 (1.31, 3.07) | 0.37 (-3.16, 4.03) | 3.29 (-1.00, 7.76) | -1.31 (-6.47, 4.15) |
| Long | Shaanxi | 3.57 (2.61, 4.54) | 1.29 (-3.43, 6.23) | 5.19 (-2.25, 13.19) | 1.77 (-4.70, 8.67) |
| Lueyang | Shaanxi | 1.56 (0.62, 2.51) | -1.52 (-5.77, 2.91) | 2.46 (-3.81, 9.14) | -0.20 (-7.94, 8.19) |
| Luochuan | Shaanxi | 1.08 (-0.32, 2.51) | 2.86 (-2.83, 8.87) | 8.42 (-1.23, 19.01) | 0.99 (-6.15, 8.68) |
| Luonan | Shaanxi | 3.23 (2.43, 4.02) | 2.60 (-3.07, 8.59) | 3.19 (-3.69, 10.56) | 1.01 (-4.17, 6.47) |
| Mei | Shaanxi | 3.87 (2.97, 4.77) | 1.25 (-3.97, 6.76) | 5.72 (-2.73, 14.91) | 2.61 (-3.00, 8.54) |
| Mian | Shaanxi | 3.62 (2.95, 4.29) | 2.11 (-2.89, 7.36) | 4.21 (-3.34, 12.34) | 3.64 (-0.60, 8.06) |
| Mizhi | Shaanxi | 3.82 (2.29, 5.37) | 6.41 (-3.04, 16.79) | 3.16 (-8.32, 16.07) | 1.42 (-6.64, 10.17) |
| Nanzheng | Shaanxi | 3.53 (2.67, 4.38) | 1.42 (-4.43, 7.63) | 4.37 (-5.34, 15.08) | 2.92 (-2.58, 8.73) |
| Ningqiang | Shaanxi | 3.80 (3.11, 4.50) | 2.85 (-2.35, 8.32) | 5.02 (0.66, 9.58) | 2.33 (-3.79, 8.83) |
| Ningshan | Shaanxi | 3.15 (2.37, 3.95) | 3.01 (-1.27, 7.49) | 4.30 (-0.64, 9.49) | 0.13 (-5.75, 6.38) |
| Pingli | Shaanxi | 4.51 (3.52, 5.52) | 3.42 (-2.81, 10.04) | 6.53 (-0.16, 13.68) | 1.98 (-5.71, 10.29) |
| Pucheng | Shaanxi | 3.12 (2.19, 4.05) | 0.23 (-2.55, 3.09) | -0.68 (-9.83, 9.39) | 2.63 (-1.72, 7.19) |
| Qian | Shaanxi | 2.85 (1.99, 3.72) | 1.17 (-4.49, 7.17) | 0.96 (-7.59, 10.29) | 1.66 (-5.04, 8.82) |
| Qianyang | Shaanxi | 3.83 (2.93, 4.74) | 3.31 (-2.63, 9.61) | 4.29 (-3.25, 12.42) | 2.97 (-2.93, 9.23) |
| Qingjian | Shaanxi | 5.83 (3.35, 8.36) | 12.99 (1.43, 25.87) | 8.03 (0.94, 15.62) | -2.37 (-18.19, 16.52) |
| Qishan | Shaanxi | 2.80 (1.93, 3.67) | 0.97 (-6.33, 8.83) | -0.63 (-7.65, 6.92) | 2.13 (-2.46, 6.94) |
| Sanyuan | Shaanxi | 3.09 (2.27, 3.93) | 3.78 (-1.74, 9.60) | 3.21 (-6.46, 13.89) | 1.55 (-4.24, 7.69) |
| Shangnan | Shaanxi | 3.40 (1.51, 5.33) | 12.42 (-7.75, 37.01) | 4.85 (-0.81, 10.84) | -0.52 (-7.50, 6.98) |
| Shanyang | Shaanxi | 4.22 (3.44, 5.00) | 3.10 (-2.66, 9.20) | 5.10 (0.72, 9.67) | 1.39 (-4.68, 7.86) |
| Shenmu | Shaanxi | 6.80 (4.29, 9.38) | 19.28 (14.43, 24.35) | 7.30 (-1.64, 17.05) | 2.17 (-11.1, 17.41) |
| Shiquan | Shaanxi | 3.94 (2.91, 4.98) | 2.67 (-2.22, 7.81) | 4.22 (-2.92, 11.89) | 1.23 (-5.45, 8.38) |
| Suide | Shaanxi | 6.22 (5.33, 7.12) | 6.22 (2.26, 10.33) | 6.57 (-0.19, 13.78) | 2.34 (-3.65, 8.70) |
| Taibai | Shaanxi | 1.79 (1.12, 2.47) | 2.37 (-4.32, 9.54) | 2.07 (-1.69, 5.97) | -0.48 (-4.51, 3.71) |
| Tongguan | Shaanxi | 3.17 (2.10, 4.25) | 1.77 (-5.34, 9.41) | 0.99 (-5.25, 7.65) | -1.09 (-8.37, 6.77) |
| Wubao | Shaanxi | 4.65 (3.39, 5.93) | 7.58 (-5.74, 22.77) | 6.51 (-1.40, 15.05) | 5.06 (-3.71, 14.63) |
| Wugong | Shaanxi | 3.36 (2.40, 4.33) | 0.02 (-8.23, 9.00) | 4.94 (-4.99, 15.90) | 3.05 (-0.46, 6.68) |
| Wuqi | Shaanxi | 0.49 (-2.80, 3.90) | 22.88 (14.09, 32.34) | 2.59 (-6.81, 12.94) | -2.52 (-10.72, 6.45) |
| Xingping | Shaanxi | 3.25 (1.92, 4.60) | -0.52 (-6.55, 5.90) | 1.58 (-12.93, 18.52) | 2.71 (-0.76, 6.30) |
| Xixiang | Shaanxi | 3.46 (2.56, 4.37) | 2.94 (-1.59, 7.68) | 4.96 (-2.43, 12.92) | -0.21 (-8.53, 8.86) |
| Xunyang | Shaanxi | 4.14 (3.16, 5.12) | 2.85 (-1.92, 7.85) | 5.54 (-0.53, 11.97) | 1.33 (-6.22, 9.49) |
| Xunyi | Shaanxi | 3.29 (2.10, 4.49) | 3.83 (-2.09, 10.12) | 6.87 (1.30, 12.74) | -2.56 (-11.15, 6.85) |
| Yanchang | Shaanxi | 5.67 (4.44, 6.91) | 7.94 (4.40, 11.59) | 8.68 (0.63, 17.37) | -0.12 (-11.14, 12.26) |
| Yanchuan | Shaanxi | -1.24 (-3.74, 1.33) | -14.63 (-33.37, 9.37) | 6.75 (4.37, 9.19) | 4.43 (-5.03, 14.83) |
| Yang | Shaanxi | 3.00 (2.27, 3.73) | 1.56 (-4.73, 8.27) | 3.18 (-3.38, 10.18) | 2.01 (-3.78, 8.15) |
| Yichuan | Shaanxi | 4.25 (3.46, 5.04) | 4.47 (-2.86, 12.35) | 3.94 (-0.85, 8.97) | 1.36 (-5.02, 8.17) |
| Yijun | Shaanxi | 3.37 (2.42, 4.32) | 3.19 (-1.95, 8.60) | 5.74 (0.32, 11.45) | -1.17 (-8.95, 7.29) |
| Yongshou | Shaanxi | 4.04 (3.12, 4.98) | 1.75 (-2.95, 6.68) | 2.96 (-4.59, 11.11) | 3.84 (-4.27, 12.63) |
| Zhashui | Shaanxi | 3.51 (2.60, 4.44) | 3.93 (-1.68, 9.87) | 4.47 (-2.55, 11.99) | -1.40 (-7.48, 5.08) |
| Zhenan | Shaanxi | 3.26 (2.42, 4.11) | 1.95 (-3.41, 7.60) | 4.74 (0.10, 9.59) | 0.45 (-6.82, 8.28) |
| Zhenping | Shaanxi | 3.05 (2.19, 3.91) | 2.62 (-4.96, 10.8) | 2.28 (-2.66, 7.47) | 2.96 (-4.49, 11.00) |
| Zhenba | Shaanxi | 3.51 (2.65, 4.37) | 2.95 (-2.17, 8.35) | 5.03 (-0.30, 10.64) | 2.09 (-3.88, 8.43) |
| Zhidan | Shaanxi | -0.78 (-3.46, 1.97) | 14.72 (-3.19, 35.93) | -0.23 (-11.67, 12.68) | -4.92 (-12.97, 3.89) |
| Zhouzhi | Shaanxi | 3.53 (2.78, 4.28) | 1.56 (-4.29, 7.78) | 5.69 (0.65, 10.97) | 3.08 (-3.02, 9.56) |
| Zichang | Shaanxi | 3.92 (2.78, 5.08) | 5.46 (0.90, 10.23) | 5.50 (-1.92, 13.49) | 4.28 (-6.68, 16.52) |
| Ziyang | Shaanxi | 3.78 (2.93, 4.65) | 2.42 (-4.14, 9.43) | 5.35 (0.51, 10.43) | 2.37 (-4.05, 9.21) |
| Zizhou | Shaanxi | 5.73 (3.32, 8.20) | 12.48 (0.61, 25.75) | 7.80 (0.06, 16.15) | 0.05 (-14.45, 17.00) |
| Anqiu | Shandong | 0.88 (0.13, 1.63) | -0.88 (-8.57, 7.46) | 0.41 (-4.02, 5.05) | 2.35 (-2.84, 7.81) |
| Boxing | Shandong | 2.69 (1.67, 3.71) | -1.33 (-12.36, 11.10) | 3.49 (-1.63, 8.89) | 2.36 (-3.27, 8.32) |
| Cao | Shandong | 2.44 (1.91, 2.98) | 1.47 (0.18, 2.77) | 0.65 (-1.89, 3.25) | 4.15 (0.44, 7.99) |
| Changdao | Shandong | 0.73 (-0.86, 2.35) | 6.48 (-3.67, 17.70) | -5.75 (-17.25, 7.34) | 5.10 (-4.17, 15.25) |
| Changle | Shandong | 2.35 (1.45, 3.25) | 0.55 (-5.50, 6.99) | -1.57 (-8.46, 5.85) | 1.31 (-3.44, 6.28) |
| Changyi | Shandong | 1.70 (0.65, 2.76) | 1.40 (-1.07, 3.93) | 0.01 (-7.26, 7.85) | 4.30 (-1.38, 10.31) |
| Chengwu | Shandong | 2.17 (1.53, 2.80) | 2.41 (-2.72, 7.81) | -0.41 (-5.08, 4.49) | 1.48 (-3.65, 6.89) |
| Chiping | Shandong | 0.98 (-0.04, 2.02) | 0.18 (-5.82, 6.56) | 3.85 (-1.98, 10.03) | -1.27 (-7.38, 5.25) |
| Dan | Shandong | 1.55 (0.43, 2.69) | 1.73 (-5.90, 9.99) | -4.51 (-9.92, 1.22) | 5.13 (0.12, 10.39) |
| Dingtao | Shandong | 3.47 (2.66, 4.29) | 1.80 (-6.38, 10.69) | 0.11 (-4.60, 5.06) | 4.84 (0.88, 8.95) |
| Donga | Shandong | 1.67 (0.81, 2.53) | 0.78 (-7.38, 9.65) | 3.60 (-1.84, 9.34) | -1.35 (-6.24, 3.80) |
| Dongming | Shandong | 3.41 (2.48, 4.36) | -0.72 (-7.05, 6.05) | 7.77 (3.30, 12.43) | 3.15 (-1.42, 7.94) |
| Dongping | Shandong | 1.28 (0.51, 2.05) | 1.01 (-3.64, 5.89) | 2.12 (-0.77, 5.09) | -2.96 (-9.66, 4.23) |
| Fei | Shandong | 1.68 (0.63, 2.74) | 0.61 (-6.07, 7.77) | 1.04 (-6.00, 8.59) | 3.70 (-1.97, 9.71) |
| Feicheng | Shandong | -0.18 (-0.93, 0.57) | -2.40 (-7.77, 3.28) | 0.69 (-5.01, 6.72) | -0.40 (-6.50, 6.09) |
| Gaomi | Shandong | -0.24 (-1.14, 0.67) | -1.72 (-11.26, 8.84) | -2.17 (-7.81, 3.81) | 0.69 (-4.70, 6.39) |
| Gaoqing | Shandong | 2.19 (1.01, 3.39) | -2.45 (-13.31, 9.76) | 2.43 (-4.50, 9.87) | 0.63 (-7.10, 9.00) |
| Gaotang | Shandong | -0.04 (-1.46, 1.39) | -4.63 (-13.11, 4.69) | 3.92 (-4.87, 13.52) | -3.87 (-11.43, 4.33) |
| Guan | Shandong | 2.75 (2.00, 3.51) | 2.86 (-3.73, 9.90) | 4.04 (-1.00, 9.34) | 1.08 (-3.36, 5.71) |
| Guangrao | Shandong | 1.49 (0.51, 2.49) | -0.68 (-8.94, 8.32) | 1.60 (-6.43, 10.32) | -1.84 (-8.33, 5.12) |
| Haiyang | Shandong | 0.57 (-1.19, 2.36) | -1.40 (-22.19, 24.95) | 0.45 (-11.37, 13.86) | 2.74 (-4.09, 10.06) |
| Huantai | Shandong | 0.18 (-1.61, 2.00) | -10.53 (-13.77, -7.17) | 0.07 (-8.94, 9.98) | 5.09 (0.42, 9.98) |
| Huimin | Shandong | 2.50 (1.94, 3.07) | 1.53 (-3.04, 6.32) | 2.97 (1.19, 4.79) | 0.64 (-4.97, 6.58) |
| Jiaozhou | Shandong | 0.83 (-0.26, 1.93) | -0.80 (-7.50, 6.39) | 1.66 (-9.52, 14.22) | 1.63 (-4.34, 7.96) |
| Jiaxiang | Shandong | 2.37 (1.76, 2.98) | 0.78 (-3.61, 5.36) | 1.09 (-2.73, 5.05) | 3.59 (-0.82, 8.18) |
| Jimo | Shandong | 0.06 (-1.64, 1.78) | -4.42 (-14.73, 7.15) | 2.22 (-6.52, 11.79) | 2.34 (-1.65, 6.48) |
| Jinxiang | Shandong | 1.74 (1.16, 2.33) | 1.34 (-2.79, 5.66) | 0.84 (-5.38, 7.47) | 2.27 (-1.89, 6.59) |
| Jiyang | Shandong | 1.92 (1.16, 2.70) | 2.48 (-3.34, 8.65) | 1.21 (-4.90, 7.71) | 1.45 (-4.80, 8.11) |
| Ju | Shandong | 1.97 (1.23, 2.70) | 1.70 (-4.97, 8.85) | 0.23 (-3.78, 4.40) | 3.33 (-1.56, 8.45) |
| Juancheng | Shandong | 1.92 (1.26, 2.59) | 1.38 (-5.45, 8.70) | 0.78 (-2.15, 3.80) | 2.39 (-0.39, 5.25) |
| Junan | Shandong | 3.12 (1.90, 4.36) | -2.32 (-6.70, 2.27) | 5.08 (-2.65, 13.41) | 4.98 (-0.25, 10.48) |
| Juye | Shandong | 1.68 (1.09, 2.26) | 0.37 (-5.46, 6.56) | 0.85 (-2.51, 4.33) | 2.32 (-1.79, 6.60) |
| Kenli | Shandong | 1.75 (0.31, 3.21) | 2.24 (-0.69, 5.27) | 5.25 (-2.12, 13.18) | -6.74 (-17.12, 4.94) |
| Laixi | Shandong | -0.22 (-1.20, 0.77) | -4.81 (-7.49, -2.04) | 3.73 (-2.40, 10.26) | -0.32 (-6.54, 6.30) |
| Laiyang | Shandong | -0.62 (-1.43, 0.20) | -2.72 (-5.60, 0.26) | -1.11 (-3.96, 1.83) | 1.70 (-2.56, 6.15) |
| Laizhou | Shandong | 0.55 (-0.30, 1.41) | -0.06 (-6.32, 6.61) | 2.17 (-3.16, 7.79) | -3.35 (-9.73, 3.49) |
| Lanling | Shandong | 2.27 (1.43, 3.12) | 1.18 (-2.89, 5.42) | 3.35 (-2.30, 9.33) | 2.09 (-2.65, 7.06) |
| Leling | Shandong | 2.20 (1.54, 2.87) | 2.02 (-5.94, 10.67) | 1.62 (-1.73, 5.09) | 3.43 (-0.06, 7.04) |
| Liangshan | Shandong | 1.20 (0.38, 2.02) | 2.75 (-3.88, 9.84) | -1.08 (-5.16, 3.18) | 2.67 (-2.38, 7.98) |
| Lijin | Shandong | 1.87 (0.81, 2.95) | -2.40 (-9.27, 4.99) | 5.32 (-1.70, 12.83) | -0.04 (-8.32, 9.00) |
| Lingcheng | Shandong | 2.03 (1.05, 3.01) | 0.29 (-8.11, 9.46) | 3.67 (-2.87, 10.64) | 2.84 (-1.83, 7.73) |
| Linqing | Shandong | 0.54 (-0.49, 1.58) | -3.43 (-11.14, 4.95) | 1.23 (-4.99, 7.87) | 0.37 (-4.39, 5.36) |
| Linqu | Shandong | 1.37 (0.71, 2.04) | -0.39 (-4.58, 3.99) | -1.53 (-5.10, 2.17) | 4.06 (0.46, 7.79) |
| Linshu | Shandong | 0.69 (-0.43, 1.82) | -1.69 (-10.63, 8.14) | 1.25 (-4.36, 7.19) | 2.50 (-2.46, 7.71) |
| Linyi | Shandong | 0.68 (-0.40, 1.77) | -5.31 (-13.59, 3.77) | 1.85 (-4.82, 8.99) | 0.96 (-3.39, 5.51) |
| Longkou | Shandong | -2.66 (-4.10, -1.19) | -9.00 (-18.53, 1.64) | -3.34 (-9.50, 3.24) | -0.09 (-5.81, 5.98) |
| Mengyin | Shandong | 0.91 (-0.06, 1.90) | 1.30 (-2.75, 5.50) | 2.72 (-2.60, 8.33) | -0.36 (-6.51, 6.20) |
| Ningjin | Shandong | 1.15 (0.28, 2.02) | -2.39 (-6.08, 1.44) | 2.11 (-3.03, 7.52) | 2.29 (-3.00, 7.86) |
| Ningyang | Shandong | 1.38 (0.66, 2.11) | 1.66 (-3.53, 7.13) | 1.58 (-3.74, 7.20) | -0.08 (-6.68, 6.98) |
| Penglai | Shandong | 1.61 (0.53, 2.69) | -0.81 (-4.86, 3.41) | 4.98 (-5.45, 16.56) | -1.02 (-9.57, 8.34) |
| Pingdu | Shandong | 0.44 (-0.53, 1.42) | -1.01 (-7.27, 5.67) | 3.45 (-2.84, 10.16) | -0.29 (-6.06, 5.83) |
| Pingyi | Shandong | 1.56 (0.55, 2.58) | -0.01 (-5.05, 5.30) | 2.01 (-3.11, 7.41) | 1.84 (-4.28, 8.34) |
| Pingyin | Shandong | 1.99 (0.86, 3.13) | 3.56 (-5.45, 13.42) | 3.02 (-4.11, 10.69) | 1.67 (-4.98, 8.79) |
| Pingyuan | Shandong | 1.00 (0.17, 1.84) | -0.47 (-4.41, 3.63) | 2.43 (-2.62, 7.73) | 1.71 (-2.63, 6.24) |
| Qihe | Shandong | 4.00 (3.11, 4.90) | 0.77 (-6.80, 8.96) | 6.72 (-1.14, 15.20) | 4.47 (1.51, 7.51) |
| Qingyun | Shandong | 1.23 (0.55, 1.91) | 2.62 (0.11, 5.21) | 0.95 (-5.69, 8.05) | 0.12 (-3.90, 4.31) |
| Qingzhou | Shandong | 0.91 (0.02, 1.81) | -0.83 (-7.02, 5.77) | -2.16 (-10.62, 7.11) | 2.00 (-3.73, 8.07) |
| Qixia | Shandong | -0.04 (-1.06, 0.99) | -3.97 (-10.31, 2.81) | 0.39 (-6.57, 7.86) | 1.01 (-4.26, 6.58) |
| Qufu | Shandong | 0.39 (-0.77, 1.55) | 0.07 (-13.13, 15.27) | -3.55 (-9.91, 3.25) | 1.44 (-4.89, 8.20) |
| Rongcheng | Shandong | -0.87 (-2.00, 0.28) | -2.17 (-5.59, 1.37) | -3.04 (-13.12, 8.21) | 0.51 (-7.59, 9.33) |
| Rushan | Shandong | -3.15 (-4.57, -1.70) | -3.46 (-7.96, 1.27) | -4.02 (-14.24, 7.41) | -4.82 (-15.56, 7.28) |
| Shanghe | Shandong | 1.70 (0.88, 2.53) | -1.40 (-9.81, 7.81) | 0.03 (-1.25, 1.32) | 2.14 (-3.08, 7.64) |
| Shen | Shandong | 2.21 (1.53, 2.89) | 1.56 (-5.70, 9.37) | 2.94 (-1.30, 7.35) | 1.19 (-3.74, 6.36) |
| Shouguang | Shandong | 1.08 (-0.08, 2.26) | -3.04 (-13.75, 9.00) | 0.84 (-8.26, 10.86) | 1.38 (-3.27, 6.26) |
| Sishui | Shandong | 1.54 (0.84, 2.25) | 4.02 (1.01, 7.12) | -0.76 (-5.66, 4.39) | 1.10 (-4.41, 6.94) |
| Tancheng | Shandong | 1.43 (0.39, 2.48) | -0.95 (-6.88, 5.36) | 1.23 (-5.37, 8.30) | 3.48 (-0.02, 7.10) |
| Tengzhou | Shandong | 0.28 (-0.52, 1.09) | -2.56 (-7.91, 3.10) | 0.63 (-3.65, 5.11) | 1.35 (-2.79, 5.67) |
| Weishan | Shandong | 2.14 (1.30, 2.99) | 1.94 (-1.97, 6.01) | 3.71 (-4.71, 12.87) | 1.20 (-5.31, 8.15) |
| Wendeng | Shandong | -2.28 (-3.65, -0.88) | -3.58 (-9.01, 2.17) | -2.08 (-13.49, 10.84) | -3.68 (-12.75, 6.33) |
| Wenshang | Shandong | 2.26 (1.64, 2.89) | 2.48 (-4.24, 9.67) | 2.31 (-2.96, 7.88) | 0.69 (-2.63, 4.13) |
| Wucheng | Shandong | 0.38 (-0.73, 1.51) | 1.62 (-8.87, 13.31) | -0.95 (-8.60, 7.34) | 2.87 (-2.11, 8.10) |
| Wudi | Shandong | 2.57 (1.98, 3.16) | 2.61 (0.59, 4.68) | 2.10 (-2.45, 6.87) | 2.72 (-3.04, 8.82) |
| Wulian | Shandong | 0.05 (-1.18, 1.29) | -2.97 (-5.20, -0.69) | 2.11 (-4.55, 9.24) | -0.13 (-7.21, 7.50) |
| Xiajin | Shandong | 0.21 (-0.39, 0.81) | 0.08 (-5.10, 5.55) | 0.40 (-2.88, 3.79) | 0.53 (-3.49, 4.71) |
| Xintai | Shandong | -0.51 (-1.32, 0.31) | -3.13 (-8.21, 2.23) | -0.51 (-6.46, 5.81) | -1.36 (-7.95, 5.71) |
| Yanggu | Shandong | 2.75 (1.96, 3.54) | 0.82 (-5.23, 7.26) | 4.55 (0.39, 8.88) | 2.22 (-1.95, 6.57) |
| Yangxin | Shandong | 2.98 (2.31, 3.66) | 4.00 (-2.41, 10.84) | 1.00 (-3.78, 6.02) | 3.97 (-0.11, 8.22) |
| Yanzhou | Shandong | -1.07 (-2.16, 0.03) | -5.00 (-14.25, 5.25) | 1.02 (-5.05, 7.48) | -2.69 (-8.45, 3.44) |
| Yinan | Shandong | 2.14 (1.15, 3.13) | -0.17 (-4.77, 4.66) | 1.98 (-2.64, 6.82) | 2.47 (-2.27, 7.45) |
| Yishui | Shandong | 1.41 (0.61, 2.23) | -1.41 (-6.61, 4.07) | -0.96 (-6.19, 4.56) | 0.88 (-4.71, 6.80) |
| Yiyuan | Shandong | 1.78 (0.89, 2.68) | 0.86 (-6.06, 8.29) | 3.12 (-3.15, 9.80) | 0.16 (-5.63, 6.30) |
| Yucheng | Shandong | -0.04 (-0.59, 0.51) | -1.89 (-6.20, 2.62) | -0.43 (-0.98, 0.12) | -0.28 (-4.95, 4.62) |
| Yuncheng | Shandong | 2.98 (2.23, 3.74) | 1.62 (-6.50, 10.44) | 2.55 (-1.08, 6.32) | 3.96 (-0.34, 8.44) |
| Yutai | Shandong | 1.29 (0.43, 2.15) | -0.12 (-6.85, 7.09) | 2.64 (-3.70, 9.40) | 1.11 (-3.67, 6.12) |
| Zhangqiu | Shandong | 1.38 (-0.04, 2.82) | -6.57 (-13.76, 1.23) | 3.26 (-6.00, 13.44) | 3.04 (-1.30, 7.58) |
| Zhanhua | Shandong | 1.95 (1.08, 2.84) | 1.25 (-6.02, 9.07) | 1.41 (0.29, 2.55) | -1.70 (-10.46, 7.93) |
| Zhaoyuan | Shandong | -0.04 (-0.87, 0.79) | 0.94 (-3.64, 5.75) | 0.48 (-6.90, 8.44) | -1.46 (-9.55, 7.35) |
| Zhucheng | Shandong | -0.28 (-1.30, 0.76) | -0.73 (-8.14, 7.28) | -0.29 (-8.54, 8.71) | 0.25 (-5.75, 6.63) |
| Zoucheng | Shandong | -0.51 (-1.40, 0.39) | -2.42 (-8.87, 4.48) | -1.68 (-9.16, 6.41) | 0.11 (-5.88, 6.49) |
| Zouping | Shandong | -1.33 (-2.58, -0.06) | -8.43 (-14.98, -1.38) | -0.42 (-8.58, 8.46) | -4.38 (-11.69, 3.54) |
| Anze | Shanxi | 1.55 (0.72, 2.38) | 4.09 (-3.34, 12.1) | 0.64 (-5.36, 7.02) | 1.27 (-5.34, 8.34) |
| Baode | Shanxi | 3.10 (1.84, 4.36) | 3.74 (-3.91, 12.01) | 6.69 (-1.44, 15.50) | -0.87 (-9.39, 8.45) |
| Dai | Shanxi | 2.42 (1.23, 3.63) | 6.42 (-1.75, 15.26) | 2.27 (-3.57, 8.45) | 0.65 (-5.83, 7.56) |
| Daning | Shanxi | 0.59 (-0.51, 1.70) | -1.15 (-8.17, 6.39) | 1.90 (-4.11, 8.28) | 3.49 (-3.62, 11.13) |
| Dingxiang | Shanxi | 1.27 (0.69, 1.86) | 0.67 (-3.29, 4.79) | -0.03 (-5.79, 6.09) | 2.15 (-3.01, 7.58) |
| Fangshan | Shanxi | 3.86 (3.12, 4.60) | 6.43 (0.07, 13.19) | 2.49 (0.38, 4.65) | 5.84 (-0.20, 12.25) |
| Fanzhi | Shanxi | 2.79 (2.27, 3.31) | 1.68 (-1.01, 4.44) | 3.57 (0.31, 6.93) | 2.30 (-3.54, 8.50) |
| Fenxi | Shanxi | -1.56 (-3.84, 0.78) | -16.02 (-23.8, -7.44) | 2.70 (-0.78, 6.30) | 0.92 (-5.79, 8.11) |
| Fenyang | Shanxi | -1.61 (-3.39, 0.21) | -12.56 (-19.93, -4.51) | -4.62 (-12.22, 3.64) | 4.94 (-0.17, 10.32) |
| Fushan | Shanxi | 0.57 (-0.72, 1.88) | -5.78 (-9.09, -2.35) | 7.89 (3.37, 12.62) | -2.55 (-9.80, 5.27) |
| Gaoping | Shanxi | 0.98 (0.17, 1.80) | 2.55 (-4.10, 9.66) | 1.28 (-7.65, 11.07) | 1.34 (-1.86, 4.63) |
| Gu | Shanxi | -1.91 (-2.61, -1.21) | 0.03 (-6.14, 6.62) | -3.54 (-7.29, 0.36) | -2.99 (-9.67, 4.17) |
| Guangling | Shanxi | 1.53 (0.56, 2.51) | -1.38 (-9.10, 7.00) | 1.90 (-2.43, 6.41) | 4.24 (2.30, 6.23) |
| Gujiao | Shanxi | -2.98 (-4.90, -1.02) | -13.04 (-20.12, -5.34) | -2.24 (-8.91, 4.91) | 1.10 (-3.00, 5.38) |
| Hejin | Shanxi | -4.34 (-6.63, -1.99) | -15.11 (-25.22, -3.64) | -7.07 (-24.39, 14.23) | 2.75 (0.14, 5.44) |
| Hequ | Shanxi | 1.84 (0.55, 3.15) | -4.48 (-10.19, 1.61) | 6.27 (-1.48, 14.64) | 1.36 (-7.38, 10.93) |
| Heshun | Shanxi | 3.20 (2.54, 3.86) | 2.63 (-0.27, 5.62) | 5.52 (2.39, 8.74) | -0.06 (-5.83, 6.06) |
| Hongtong | Shanxi | -0.54 (-2.56, 1.52) | -11.19 (-27.45, 8.72) | 3.18 (-6.06, 13.33) | 1.02 (-2.57, 4.75) |
| Houma | Shanxi | -1.61 (-3.55, 0.37) | -6.04 (-15.58, 4.56) | -1.11 (-12.81, 12.15) | 5.50 (3.37, 7.68) |
| Huairen | Shanxi | 2.81 (0.99, 4.65) | 1.51 (-5.80, 9.39) | 5.39 (-11.47, 25.46) | 0.07 (-5.34, 5.79) |
| Huguan | Shanxi | -0.23 (-1.88, 1.45) | -9.44 (-13.68, -5.00) | 1.26 (-5.05, 8.00) | 4.03 (-1.20, 9.54) |
| Hunyuan | Shanxi | 2.94 (2.14, 3.75) | 1.96 (-4.88, 9.29) | 2.26 (-4.18, 9.13) | 2.24 (-3.31, 8.11) |
| Huozhou | Shanxi | -1.46 (-2.82, -0.08) | -7.63 (-20.97, 7.97) | 1.03 (-6.97, 9.73) | 0.05 (-3.92, 4.19) |
| Ji | Shanxi | 1.50 (0.49, 2.51) | -2.94 (-9.25, 3.80) | 3.64 (-0.59, 8.05) | -1.10 (-6.67, 4.81) |
| Jiaocheng | Shanxi | -0.42 (-1.26, 0.42) | -2.19 (-4.18, -0.15) | -2.93 (-12.24, 7.38) | 2.14 (-2.91, 7.45) |
| Jiaokou | Shanxi | -1.15 (-3.29, 1.05) | -13.48 (-21.43, -4.72) | 6.92 (-1.51, 16.07) | -0.37 (-9.97, 10.25) |
| Jiexiu | Shanxi | -1.51 (-3.55, 0.57) | -7.35 (-25.10, 14.62) | -3.18 (-22.19, 20.46) | 3.20 (0.27, 6.23) |
| Jingle | Shanxi | 3.00 (2.11, 3.90) | -0.10 (-3.91, 3.86) | 6.49 (4.25, 8.78) | 2.42 (-2.84, 7.96) |
| Jishan | Shanxi | 0.97 (-0.28, 2.24) | -6.58 (-14.10, 1.61) | 1.16 (-6.68, 9.65) | 1.62 (-2.06, 5.44) |
| Lan | Shanxi | 4.57 (3.49, 5.66) | 0.64 (-5.25, 6.90) | 9.51 (1.90, 17.69) | 1.77 (-3.06, 6.83) |
| Licheng | Shanxi | 0.51 (-0.32, 1.35) | -2.85 (-8.68, 3.35) | 3.59 (-1.21, 8.62) | -0.38 (-6.19, 5.80) |
| Lin | Shanxi | 5.08 (4.24, 5.93) | 5.9 (-0.84, 13.09) | 4.24 (-2.34, 11.27) | 7.09 (0.52, 14.10) |
| Lingchuan | Shanxi | 1.98 (1.07, 2.91) | 0.04 (-7.52, 8.21) | 0.90 (-4.22, 6.29) | 1.27 (-3.40, 6.16) |
| Lingqiu | Shanxi | 0.26 (-0.58, 1.11) | -4.19 (-7.81, -0.42) | -2.33 (-7.44, 3.06) | 3.35 (-0.91, 7.80) |
| Lingshi | Shanxi | 1.47 (0.26, 2.69) | 0.32 (-10.64, 12.62) | 3.08 (-5.05, 11.92) | -0.20 (-10.38, 11.13) |
| Linyi | Shanxi | 3.03 (2.27, 3.80) | -0.50 (-5.75, 5.05) | 3.67 (-0.69, 8.22) | 0.05 (-4.37, 4.68) |
| Liulin | Shanxi | 1.08 (-1.81, 4.05) | 13.32 (4.64, 22.72) | -3.56 (-19.03, 14.88) | 4.03 (-3.14, 11.73) |
| Loufan | Shanxi | 0.96 (-0.56, 2.50) | -9.04 (-12.59, -5.34) | 5.61 (-0.95, 12.61) | 3.28 (-4.24, 11.38) |
| Lucheng | Shanxi | -2.65 (-5.10, -0.14) | -14.08 (-25.46, -0.96) | 2.49 (-7.83, 13.97) | 5.56 (3.06, 8.12) |
| Ningwu | Shanxi | 2.75 (1.46, 4.06) | -1.39 (-5.22, 2.60) | 8.79 (-0.30, 18.71) | 1.60 (-5.90, 9.69) |
| Pianguan | Shanxi | 1.63 (0.77, 2.51) | -0.61 (-4.17, 3.09) | 3.63 (-5.73, 13.92) | 4.09 (-1.28, 9.75) |
| Pingding | Shanxi | 1.29 (0.29, 2.30) | 0.35 (-8.01, 9.48) | 3.50 (-6.65, 14.76) | 1.66 (-1.73, 5.16) |
| Pinglu | Shanxi | 0.46 (-0.98, 1.92) | -5.95 (-18.64, 8.72) | 0.40 (-4.04, 5.05) | -0.41 (-3.89, 3.20) |
| Pingshun | Shanxi | 2.34 (1.58, 3.10) | 1.41 (-2.71, 5.69) | 4.59 (-0.83, 10.30) | -0.14 (-5.42, 5.45) |
| Pingyao | Shanxi | 0.06 (-1.23, 1.37) | -2.34 (-12.24, 8.66) | 0.90 (-11.27, 14.75) | 2.89 (-1.83, 7.84) |
| Pu | Shanxi | 0.20 (-2.95, 3.46) | -20.13 (-32.32, -5.76) | 7.08 (1.44, 13.04) | 2.97 (-5.43, 12.12) |
| Qi | Shanxi | 1.39 (0.49, 2.30) | -2.65 (-6.53, 1.39) | 3.15 (-4.34, 11.24) | 2.28 (-2.31, 7.08) |
| Qin | Shanxi | 1.94 (1.13, 2.75) | -0.58 (-5.48, 4.57) | 1.29 (-1.96, 4.65) | -0.26 (-6.39, 6.26) |
| Qingxu | Shanxi | 1.05 (-0.68, 2.80) | -3.79 (-13.21, 6.65) | 2.55 (-4.21, 9.79) | 8.63 (4.96, 12.43) |
| Qinshui | Shanxi | 2.72 (1.49, 3.97) | 7.89 (-2.82, 19.78) | 6.37 (2.99, 9.87) | -1.85 (-8.22, 4.96) |
| Qinyuan | Shanxi | 2.54 (1.48, 3.61) | 4.03 (-6.16, 15.34) | 0.78 (-3.04, 4.74) | 2.11 (-9.18, 14.8) |
| Quwo | Shanxi | -0.45 (-2.15, 1.27) | -6.54 (-22.56, 12.78) | 2.47 (-7.92, 14.02) | 2.56 (-0.69, 5.92) |
| Ruicheng | Shanxi | 1.37 (0.71, 2.03) | 1.15 (-1.45, 3.82) | 1.33 (-2.57, 5.39) | 0.42 (-2.95, 3.91) |
| Shangdang | Shanxi | 1.70 (0.03, 3.40) | 4.55 (-12.64, 25.13) | 2.65 (-10.93, 18.30) | 4.58 (0.56, 8.77) |
| Shanyin | Shanxi | 3.16 (1.18, 5.19) | 12.84 (6.17, 19.94) | 1.81 (-10.18, 15.41) | 2.66 (-4.86, 10.78) |
| Shenchi | Shanxi | 4.12 (2.56, 5.71) | -1.96 (-10.44, 7.31) | 8.63 (-7.10, 27.01) | 5.87 (-1.20, 13.45) |
| Shilou | Shanxi | 3.84 (3.19, 4.49) | 5.75 (2.33, 9.28) | 3.92 (1.39, 6.52) | 8.17 (4.86, 11.59) |
| Shouyang | Shanxi | 2.68 (1.57, 3.81) | 7.88 (5.15, 10.67) | 2.47 (-1.51, 6.62) | -0.79 (-8.21, 7.23) |
| Taigu | Shanxi | 1.85 (0.56, 3.16) | -3.42 (-13.79, 8.20) | 5.42 (0.60, 10.47) | 0.72 (-2.51, 4.07) |
| Tianzhen | Shanxi | 3.43 (2.73, 4.13) | 0.19 (-3.94, 4.50) | 3.10 (0.51, 5.75) | 3.36 (-0.56, 7.44) |
| Tunliu | Shanxi | 1.23 (0.01, 2.47) | 0.28 (-12.04, 14.32) | 5.40 (-4.09, 15.84) | 2.01 (-4.54, 9.02) |
| Wanrong | Shanxi | 2.91 (2.12, 3.70) | 0.33 (-8.57, 10.09) | 1.41 (-1.70, 4.62) | 1.03 (-2.36, 4.54) |
| Wenshui | Shanxi | -0.46 (-1.87, 0.97) | -2.46 (-17.03, 14.67) | -2.52 (-14.28, 10.86) | 1.20 (-4.08, 6.78) |
| Wenxi | Shanxi | 0.30 (-0.77, 1.38) | -3.21 (-6.23, -0.10) | -2.55 (-8.42, 3.71) | 5.49 (0.43, 10.79) |
| Wutai | Shanxi | 2.18 (1.60, 2.76) | 1.31 (-2.96, 5.76) | 3.80 (-0.11, 7.86) | 0.12 (-2.78, 3.10) |
| Wuxiang | Shanxi | 1.09 (0.25, 1.94) | 1.05 (-5.81, 8.41) | 2.07 (-3.76, 8.25) | -1.29 (-7.63, 5.49) |
| Wuzhai | Shanxi | 3.77 (2.86, 4.69) | -1.19 (-7.24, 5.25) | 3.74 (-1.17, 8.90) | 7.04 (1.72, 12.64) |
| Xi | Shanxi | 1.60 (0.69, 2.51) | -0.94 (-4.78, 3.06) | 4.58 (2.04, 7.19) | 1.03 (-7.31, 10.12) |
| Xia | Shanxi | 2.42 (1.61, 3.23) | 0.41 (-8.88, 10.64) | 1.60 (-2.05, 5.37) | 0.89 (-2.70, 4.63) |
| Xiangfen | Shanxi | -1.66 (-4.01, 0.75) | -15.62 (-27.45, -1.86) | 4.94 (-2.58, 13.04) | 1.56 (-1.69, 4.91) |
| Xiangning | Shanxi | 1.51 (0.50, 2.53) | -2.98 (-6.95, 1.17) | 5.60 (0.63, 10.81) | 2.44 (-3.67, 8.94) |
| Xiangyuan | Shanxi | -0.70 (-1.82, 0.43) | 1.95 (-6.69, 11.40) | -3.99 (-9.54, 1.90) | 4.10 (-1.64, 10.18) |
| Xiaoyi | Shanxi | -2.85 (-4.86, -0.78) | -12.82 (-25.85, 2.48) | -3.39 (-20.52, 17.45) | -3.29 (-12.42, 6.78) |
| Xing | Shanxi | 6.48 (4.95, 8.03) | 1.92 (-7.87, 12.74) | 5.60 (-0.91, 12.54) | 6.52 (-3.22, 17.23) |
| Xinjiang | Shanxi | 1.63 (0.22, 3.07) | -6.12 (-16.53, 5.60) | -0.53 (-10.86, 10.99) | 0.91 (-2.63, 4.57) |
| Xiyang | Shanxi | 2.81 (2.19, 3.43) | 3.32 (0.27, 6.47) | 3.73 (0.11, 7.49) | 2.24 (-3.78, 8.64) |
| Yangcheng | Shanxi | 1.21 (0.47, 1.96) | 0.77 (-2.57, 4.22) | 1.17 (-5.75, 8.59) | 0.10 (-6.38, 7.02) |
[truncated: 53,707 more chars]
